# Chemical generation of checkpoint inhibitory T cell engagers for the treatment of cancer

**DOI:** 10.1038/s41557-023-01280-4

**Published:** 2023-07-24

**Authors:** Peter A. Szijj, Melissa A. Gray, Mikaela K. Ribi, Calise Bahou, João C. F. Nogueira, Carolyn R. Bertozzi, Vijay Chudasama

**Affiliations:** 1 Department of Chemistry, University College London, London, UK; 2 Department of Chemistry, Sarafan ChEM-H, and Howard Hughes Medical Institute, Stanford University, Stanford, CA USA

**Keywords:** Chemical modification, Protein design

## Abstract

Bispecific T cell engagers (BiTEs), a subset of bispecific antibodies (bsAbs), can promote a targeted cancer cell’s death by bringing it close to a cytotoxic T cell. Checkpoint inhibitory T cell engagers (CiTEs) comprise a BiTE core with an added immunomodulatory protein, which serves to reverse cancer-cell immune-dampening strategies, improving efficacy. So far, protein engineering has been the main approach to generate bsAbs and CiTEs, but improved chemical methods for their generation have recently been developed. Homogeneous fragment-based bsAbs constructed from fragment antigen-binding regions (Fabs) can be generated using click chemistry. Here we describe a chemical method to generate biotin-functionalized three-protein conjugates, which include two CiTE molecules, one containing an anti-PD-1 Fab and the other containing an immunomodulatory enzyme, *Salmonella typhimurium* sialidase. The CiTEs’ efficacy was shown to be superior to that of the simpler BiTE scaffold, with the sialidase-containing CiTE inducing substantially enhanced T cell-mediated cytotoxicity in vitro. The chemical method described here, more generally, enables the generation of multi-protein constructs with further biological applications.

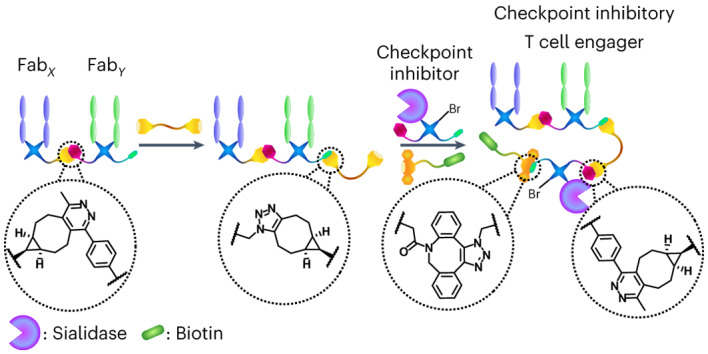

## Abstract (web summary)

Three-protein conjugates, which have so far been produced using protein-engineering strategies, can now be generated using a chemical approach that enables the addition of small-molecule functionality. Checkpoint inhibitory T cell engagers (CiTEs) were assembled and shown to have enhanced in vitro potency compared to a traditional T cell engager.

## Main

There are now five examples of bispecific antibodies (bsAbs) as anticancer therapeutics on the market, three of which have been approved by the US Food and Drug Administration and European Medicines Agency since 2021^1–3^. These bsAbs can simultaneously bind to two distinct antigenic epitopes, which can facilitate downstream biology that monospecific antibodies are not capable of performing^4^. Through the generation of multi-protein conjugates, especially with the option to attach small-molecule functionalities, further advanced mechanisms of action can be accessed. A promising class of such molecules combines T cell re-directing bsAb technology with immunomodulating proteins for additional therapeutic benefit^5^. Here we report a chemical method to generate functionalized three-protein conjugates and test their efficacy in vitro.

So far, genetic and protein engineering to generate fused amino-acid sequences, which can then be expressed, has been the standard approach for generating bsAbs. However, the field of protein bioconjugation (that is, how to attach small molecules to proteins) has afforded chemical methods for bsAb production, which can offer benefits over expression-based methods as they conceptually offer greater modularity, speed and potentially inherent handles for further functionalization, such as bsAb-drug or bsAb-fluorophore conjugates. For a more comprehensive overview of the subject of chemical bsAb synthesis, the readers are referred to two recent reviews on the topic^6,7^.

Re-bridging the solvent-accessible interchain disulfide bonds of antibodies or their antigen-binding fragments (Fabs) affords site-selective homogeneous bsAb formation. Generating homogeneous and well-defined bsAbs is possible through disulfide re-bridging because the natural abundance of cysteine is low^8^, and most antibodies contain four readily accessible disulfides, with Fabs containing only one. The early chemical tools include a PEG with two bis-sulfones at either end to generate Fab–PEG–Fab^9^. Maleimide molecules with leaving groups on each double bond—termed next-generation maleimides (NGMs)—have been used to synthesize a range of constructs (Fab–ScFv, albumin–Fab and (ScFv)_3_)^10,11^, and combining the NGM platform with strain-promoted azide–alkyne cycloaddition (SPAAC) click chemistry has been used to generate Fab–Fab^12^ and full-length IgG2–IgG2^13^ (IgG, immunoglobulin G). These methods were useful but were limited by long reaction times, poor yields and the inability for further functionalization.

**Fig. 1:**
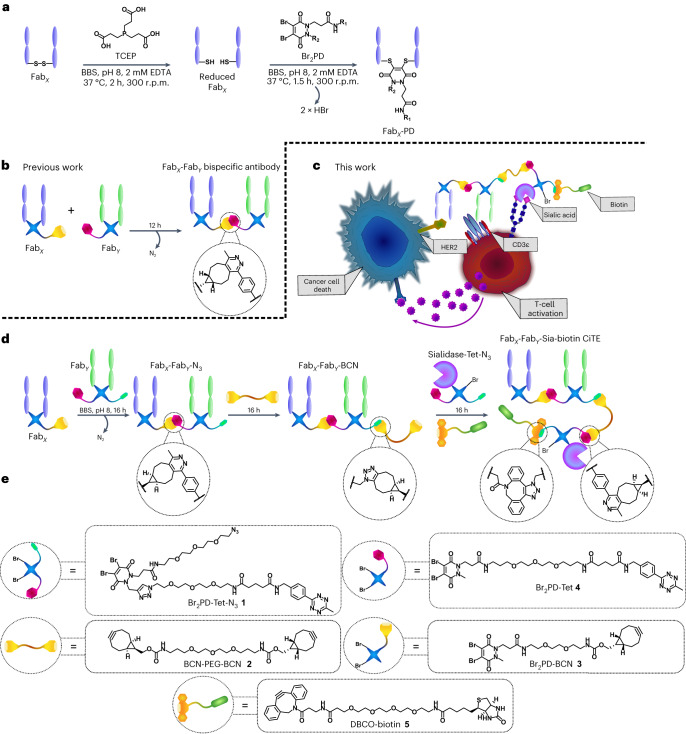
CiTE generation and proposed underlying biological mechanism. **a**, The pyridazinedione method for the generation of functionalized Fabs. The Fab is first reduced with TCEP to liberate the cysteines of the single interchain disulfide bond. The reduced Fab is then reacted with the Br_2_PD of choice, via an addition-elimination mechanism whereby the thiols sequentially displace each Br atom to generate a stable covalent linkage between the heavy and light chains of the protein. BBS, Borate buffered saline. **b**, The previously developed method for the generation of bsAbs with pyridazinediones by means of SPIEDAC click chemistry. **c**, Proposed mechanism of action of a sialidase-containing CiTE. The CiTE binds to a target cancer cell through HER2-engagement and to a T cell through the CD3 co-receptor, crosslinking the two cells. The sialidase enzyme removes sialic acid from both target and effector (T) cell to enhance immune activation, leading to more potent T cell-mediated cytotoxicity. The CiTE is functionalized with a biotin molecule to help imaging and/or purification. **d**, The method developed in this manuscript for the generation of functionalized three-protein CiTE constructs. **e**, The pyridazinediones and other small molecules used in this work (Br_2_PD–Tet–N_3_ **1**, BCN–PEG–BCN linker **2**, Br_2_PD–BCN **3**, Br_2_PD–Tet **4** and DBCO–biotin **5**) for the biotinylation of the constructs.

Recently, a rapid and modular click chemistry-based method for the construction of homogeneous bispecific antibody conjugates was developed that has the ability to add further functionality to the bsAb^14^. The method was based on the dibromopyridazinedione (Br_2_PD) scaffold (Fig. 1a)^15–19^, in which the interchain disulfide of a Fab is reduced with TCEP (tris(2-carboxyethyl)phosphine) and reacted with a Br_2_PD molecule, leading to two sequential addition-elimination reactions where each Br atom is displaced by the S atom of one of the cysteine residues. This leads to a 2-carbon covalent linkage between the heavy and light chains of the Fab, which is stable in blood serum^17^. This method was employed to re-bridge the disulfide bonds of Fabs and functionalize the protein with bioorthogonal click handles (strained alkyne and tetrazine). These click-enabled Fabs could react with each other through the strain-promoted inverse electron-demand Diels–Alder cycloaddition (SPIEDAC) reaction to generate a bsAb construct where the two proteins are linked by a flexible PEG-containing tether (Fig. 1b). As pyridazinediones contain two N atoms in the ring, a second functional handle could be introduced. This was demonstrated with the attachment of two distinct fluorescent dyes to the bsAb via Cu-catalysed azide–alkyne cycloaddition^14^. As Cu is difficult to remove and also toxic, developing a method where both click reactions are Cu-free would be desirable for the production of a three-protein conjugate.

Recently, a Cu-free pyridazinedione-based approach was used to generate IgG-like bsAbs—SynAbs (synthetic antibodies). The Fc modality of an anti-CD20 mAb (rituximab) was modified with either strained alkyne (BCN, bicyclo[6.1.0]non-4-yne) or tetrazine click handles, and reacted sequentially with Fab species, each harbouring a complementary click handle, to form mono- or bispecific SynAbs^20^. This strategy was an iteration of the previously described pyridazinedione-based method for bsAb generation that additionally allowed for the introduction of Fc-mediated functionality, such as half-life extension or effector function. These SynAbs were, however, not functionalized with additional small molecules. Importantly, the strategy employed to generate SynAbs was predicated on using an Fc as the core of the three-protein construct, limiting utility to IgG-like species. Thus a new method needed to be developed to allow for the generation of three-protein constructs with a wider selection of constituent proteins, that is, not limited to two Fabs and one Fc. We thus established a chemical method for three-protein conjugate synthesis, suitable for attachment of an additional checkpoint inhibitory modality to a T cell-engager core.

The class of functional molecules we generate here can be termed ‘checkpoint inhibitory T cell engagers’ (CiTEs)^21^. CiTEs combine the cytotoxic ability of bispecific T cell engagers (BiTEs; note, in this Article we use the term BiTE in the broader sense to encompass all bispecific T cell engager formats)^22,23^ with a checkpoint inhibitory modality to further enhance T cell activation and thus efficacy. Limited examples of such three- or four-protein conjugates, generated through protein engineering, have been reported in the context of immunotherapy^5^.

In the field of T cell redirection it has been shown that blockade of the PD-1/PD-L1 immune checkpoint is synergistic with BiTE treatment^24^. Target cells not constitutively expressing PD-1 can upregulate this immunosuppressive protein following the addition of BiTEs. Based on these observations, the generation of fusion proteins with an anti-CD33 BiTE core combined with a PD-L1-blocking antibody fragment (or the low-affinity extracellular portion of the PD-1 protein) were generated^21^. The authors of the work dubbed these molecules checkpoint inhibitory T cell engagers (CiTEs). This study provided an elegant and promising new strategy for combining checkpoint blockade with immune cell redirection.

However, these three-protein conjugate platforms do not incorporate an Fc fragment or similar half-life-extending functionality, and might require other approaches to improve pharmacokinetics^21^. Many parameters have to also be considered for their construction (for example, the cancer target, binding affinities, the immune checkpoint pathway to modulate, potential side effects caused by immune-cell activation, half-life, tumour penetration and Fc-mediated effector function or lack thereof), such that the addition of small molecules to either modulate function, provide theranostic capabilities or just as tools to allow for monitoring of the biodistribution of these species could prove beneficial. Therefore, a modular chemical method that can rapidly produce conjugates from a pool of components for initial testing would be advantageous^5^. Additionally, few CiTEs have been described in the literature and there are thus many combinations of checkpoint inhibitor and BiTE that are yet to be explored. Among these is the checkpoint inhibitory enzyme *Salmonella typhimurium* (ST) sialidase (Sia), which has been studied recently in combination with antibody-mediated targeting^25^.

Thus, we set out to develop a chemical method for the attachment of an additional (checkpoint inhibitory) protein to a BiTE core (Fig. 1c). The method developed allowed for the introduction of small-molecule functionality to these three-protein constructs (in the form of a biotin molecule to assist imaging, in this case). This work thus explores the bioorthogonal Cu-free chemical construction of functionalized bsAbs, followed by the generation of functionalized bsAb-enzyme and trispecific antibody conjugates. So far, to the best of our knowledge, only IgG-like complexes composed of three different proteins have been assembled via chemical means^20^. Finally, to showcase the functionality of these constructs, and to demonstrate that a key characteristic of the method described herein is its modularity, functional CiTE molecules were generated. In addition to an anti-HER2/anti-CD3 BiTE core, these constructs incorporated either an anti-PD-1 Fab or a checkpoint inhibitory enzyme (sialidase)^25^. The biological activities of these were then explored in vitro in a co-culture assay between cancer cells and T cells (Fig. 1d).

## Results and discussion

### Chemical CiTE construct generation

Multiple methods were trialled to generate the desired three-protein CiTEs, as described in this Article and in the Supplementary Information. The initial strategy, relying on the conversion of a bsAb–N_3_ into a bsAb–PDBr_2_ through SPAAC click with a bicyclononyne (BCN) strained alkyne-functionalized pyridazinedione molecule, followed by addition of reduced Fab or ST sialidase (expressed with an SLCTPSRGS amino-acid sequence at the C terminus to introduce a solvent-accessible cysteine)^25^ to react with the pyridazinedione molecule on the bsAb, met with some success. It was, however, hard to reproduce due to competing side reactions, which made the process less reliable. We discuss these initial results in detail in the Supplementary Information. The subsequently developed method, which will be detailed here, relied on the SPIEDAC reaction between tetrazine and BCN strained alkyne to achieve all protein–protein linkages. As this reaction was previously shown to work well for bispecific formation^14^, it was envisaged that it would be optimal for the installation of the third protein (sialidase **6** or Fab_PD-1_ **7**).

The plan thus involved initially generating a bispecific Fab–Fab construct bearing an azide handle. This Fab_*X*_–Fab_*Y*_–N_3_ construct would then be converted to Fab_*X*_–Fab_*Y*_–BCN via reaction with BCN–PEG–BCN **2** (in tenfold excess to limit crosslinking). This Fab_*X*_–Fab_*Y*_–BCN could then be reacted with Sia–Tet–N_3_ **8** or Fab_PD-1_–Tet–N_3_ **9** and DBCO–biotin **5** in situ, to add the enzyme or third Fab (via tetrazine–BCN click), and a biotin molecule (via azide–DBCO click) to further aid in purification or imaging. The enzymatic generation of the Fab moieties from the corresponding full antibodies is discussed in detail in the Supplementary Information.

**Fig. 2:**
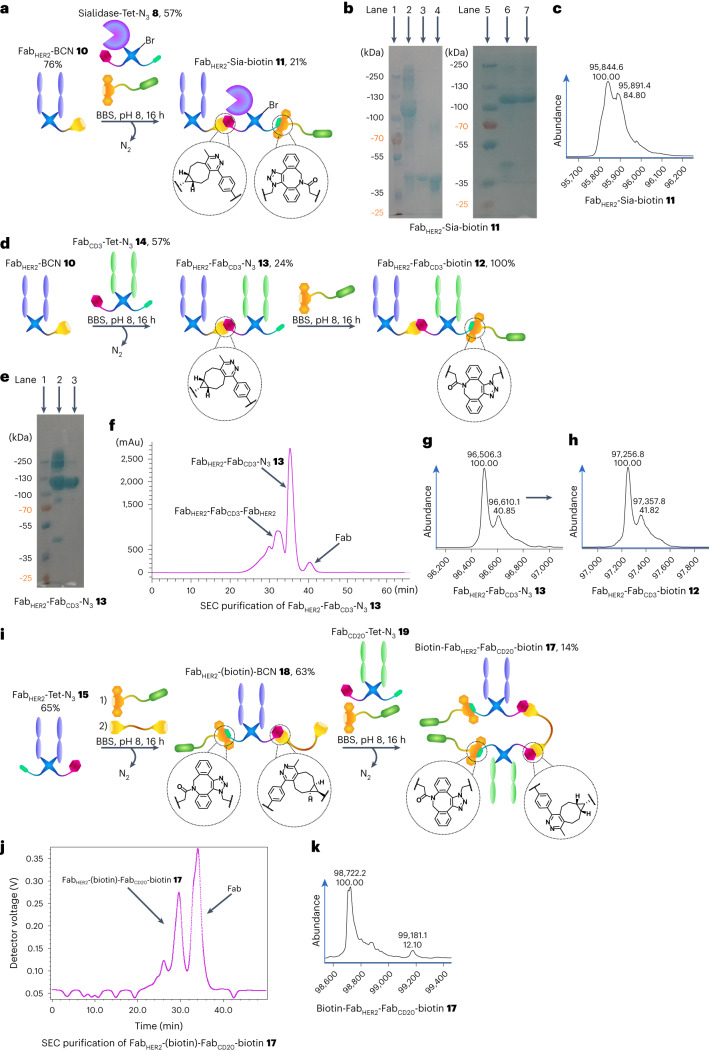
Generation of biotinylated bsAbs and the Fab_HER2_–sialidase conjugate **11** with pyridazinediones. **a**, Generation of Fab_HER2_–Sia–biotin **11**. Fab_HER2_–BCN **10** was reacted with Sia–Tet–N_3_ **8** and DBCO–biotin **5** to generate Fab_HER2_–Sia–biotin **11** after monomeric avidin agarose purification. **b**, SDS–PAGE analysis of Fab_HER2_–Sia–biotin **11**. Lanes 1 and 5: ladder. Lane 2: crude Fab_HER2_–Sia–biotin **11**. Lane 3: Fab_HER2_. Lane 4: Sia–Tet–N_3_ **8**. Lane 6: non-bound fraction of monomeric avidin agarose purification. Lane 7: bound fraction of purification; Fab_HER2_–Sia–biotin **11**. **c**, LC-MS analysis of Fab_HER2_–Sia–biotin **11**. Expected mass: 95,873 Da. Observed mass: 95,845 Da and 95,891 Da (*Δ* = 46 Da, formic acid, MS adduct). **d**, Generation of Fab_HER2_–Fab_CD3_–biotin **12**. Fab_HER2_–BCN **10** was reacted with Fab_CD3_–Tet–N_3_ **14** to form Fab_HER2_–Fab_CD3_–N_3_ **13**. This construct was then reacted with DBCO–biotin **5** to generate Fab_HER2_–Fab_CD3_–biotin **12** after SEC purification. **e**, SDS–PAGE analysis of Fab_HER2_–Fab_CD3_–N_3_ **13**. Lane 1: ladder. Lane 2: crude Fab_HER2_–Fab_CD3_–N_3_ **13**. Lane 3: purified Fab_HER2_–Fab_CD3_–N_3_ **13**. **f**, Ultraviolet (UV) trace of SEC purification of Fab_HER2_–Fab_CD3_–N_3_ **13**. **g**, LC-MS analysis of Fab_HER2_–Fab_CD3_–N_3_ **13**. Expected mass: 96,496 Da. Observed mass: 96,506 Da. **h**, LC-MS analysis of Fab_HER2_–Fab_CD3_–biotin **12**. Expected mass: 97,246 Da. Observed mass: 97,257 Da. **i**, Generation of Fab_HER2_–(biotin)–Fab_CD20_–biotin **17**. Fab_HER2_–Tet–N_3_ **15** was reacted with DBCO–biotin **5** for 1 h to afford Fab_HER2_–Tet–biotin **16**, followed by in situ addition of BCN–PEG–BCN **2** to generate Fab_HER2_–(biotin)–BCN **18** over a further 15 h. After removal of excess small molecule, this was reacted with Fab_CD20_–Tet–N_3_ **19** and DBCO–biotin **5** in situ to generate Fab_HER2_–(biotin)–Fab_CD20_–biotin **17** after SEC purification. **j**, UV trace of SEC purification of Fab_HER2_–(biotin)–Fab_CD20_–biotin **17**. **k**, LC-MS analysis of Fab_HER2_–(biotin)–Fab_CD20_–biotin **17**. Expected mass: 98,734 Da. Observed mass: 98,722 Da and 99,181 Da (biotin–Fab_HER2_–Fab_HER2_–biotin **20**, expected mass: 99,193 Da). Generation of most Fab conjugates was carried out two or three times, yielding similar results. Each protein–protein construct was generated a single time unless otherwise stated.

Initially, Fab_HER2_–BCN **10** was reacted sequentially in a one-pot reaction with Sia–Tet–N_3_ **8** and DBCO–biotin **5** (Fig. 2a) to assess the orthogonality of the tetrazine–BCN and DBCO–azide clicks, as well as to test the stability of the sialidase enzyme **6** under the reaction conditions. As the enzyme was previously found to be acid-sensitive, the click reaction was carried out at pH 7 (PBS) instead of pH 5 (acetate). The reaction proceeded well, generating Fab_HER2_–Sia–biotin **11** (Fig. 2b). After monomeric avidin agarose purification, clean Fab_HER2_–Sia–biotin **11** was isolated (21% yield), with the purity confirmed by liquid-chromatography mass spectrometry (LC-MS; Fig. 2b) and sodium dodecyl sulfate polyacrylamide gel electrophoresis (SDS–PAGE; Fig. 2c). Complete LC-MS spectra of the isolated constructs discussed in this manuscript are provided in the Supplementary Information. Additionally, a Fab_HER2_–Fab_CD3_–biotin **12** BiTE bsAb was synthesized (Fig. 2d). Initially, Fab_HER2_–Fab_CD3_–N_3_ bsAb **13** (24% yield) was constructed, then, after size exclusion chromatography (SEC) purification (Fig. 2f), this was reacted with DBCO–biotin to yield the biotinylated construct Fab_HER2_–Fab_CD3_–biotin **12** (100% yield). The purity of the constructs was confirmed by SDS–PAGE (Fig. 2e) and LC-MS (Fig. 2g,h). Please note that the MS spectra for all constructs containing Fab_CD3_ contain an additional peak at ~+110 Da. We believe this is due to papain cutting mAb_CD3_ (OKT3) at either end of an asparagine residue, leading to two Fab_CD3_ species. This is explained in more detail in the Supplementary Information. As this variation is in the hinge region, no impact on binding affinity is expected.

As the bsAb produced by this method had an azide handle, it had to be converted to either a tetrazine or BCN to enable a tetrazine–BCN click to install the final protein. In this way we could ensure that all protein–protein attachment steps would be driven by the extremely fast BCN–tetrazine IEDDA click, shown to be the best reaction to overcome the steric hindrance that makes the coupling of such large molecules difficult^26^. To this end, BCN–PEG–BCN molecule **2** was synthesized (details are provided in the Supplementary Information) to enable the conversion of bsAb–N_3_ into bsAb–BCN.

To test the BCN–PEG–BCN molecule **2** and attempt the construction of a dually modified bsAb with Cu-free click chemistry, the synthesis of Fab_HER2_–(biotin)–Fab_CD20_–biotin **17** was carried out (Fig. 2i). Fab_HER2_–Tet–N_3_ **15** was reacted with DBCO–biotin **5** followed by BCN–PEG–BCN **2** sequentially, to generate Fab_HER2_–(biotin)–BCN **18** (63% yield). This was then further reacted with Fab_CD20_–Tet–N_3_ **19** and DBCO–biotin **5** in situ to yield Fab_HER2_–(biotin)–Fab_CD20_–biotin **17** (14% yield) after SEC purification (Fig. 2j). The purity of the construct was assessed via LC-MS (Fig. 2k). About 10% Fab_HER2_–(biotin)–Fab_HER2_–biotin **20** impurity was observed, stemming from unwanted dimerization during the BCN–PEG–BCN **2**-addition step of the reaction. This could be mitigated by further reducing the concentration of the reaction and increasing the equivalents of BCN–PEG–BCN **2**. Unfortunately, the solubility of BCN–PEG–BCN **2** in water was suboptimal, and thus required careful monitoring to ensure that the compound did not precipitate out of solution. This is not a major limitation when low equivalents are sufficient, but in this case where controlling a competing side reaction depends on a large excess of the molecule, it is a concern. Here, two biotin molecules were installed into the construct, but as they were added at different stages, two distinct cargo molecules could just as easily have been added. Thus, a method for the Cu-free dual modification of a chemically constructed bsAb has been developed.

**Fig. 3:**
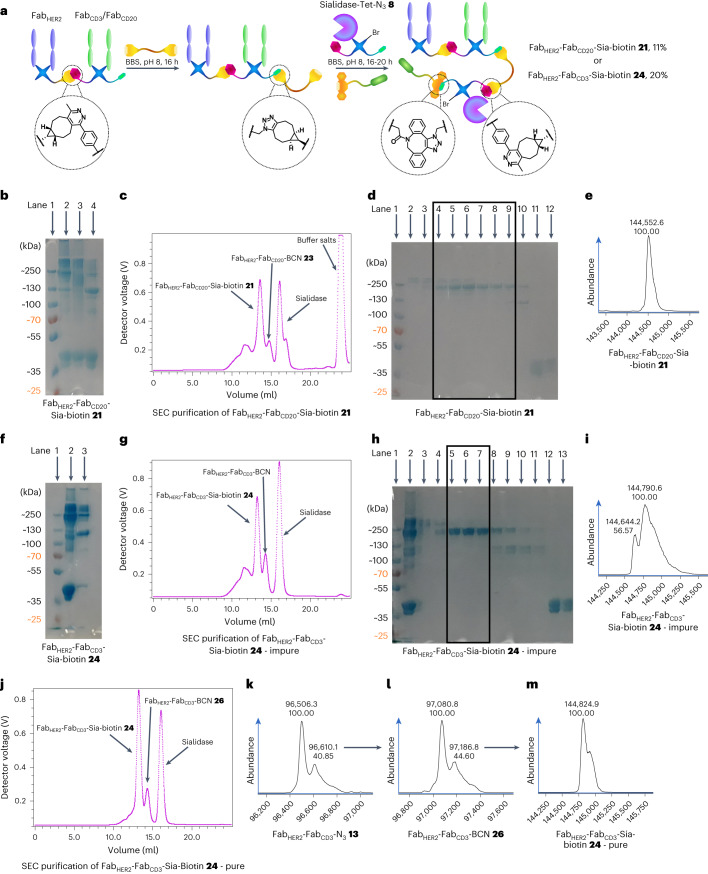
Synthesis of bsAb–Sia conjugates: Fab_HER2_–Fab_CD20_–Sia–biotin **21** and Fab_HER2_–Fab_CD3_–Sia–biotin CiTE **24**. **a**, Method for the synthesis of bsAb–Sia conjugates. Fab_*X*_–Fab_*Y*_–N_3_ is prepared as outlined before. This is then either SEC-purified (for maximum final purity) or taken forward without purification to be reacted with BCN–PEG–BCN **2** to generate Fab_*X*_–Fab_*Y*_–BCN. Sia–Tet–N_3_ **8** and DBCO–biotin **5** are then added and reacted in situ to form Fab_*X*_–Fab_*Y*_–Sia–biotin, which is then isolated after SEC purification. **b**, SDS–PAGE of Fab_HER2_–Fab_CD20_–Sia–biotin **21** formation. Lane 1: ladder. Lane 2: Fab_HER2_–Fab_CD20_–Sia–biotin **21** heated at 95 °C for 5 min. Lane 3: unheated Fab_HER2_–Fab_CD20_–Sia–biotin **21**. Lane 4: Fab_HER2_–Fab_CD20_–N_3_ **22** + Sia–Tet–N_3_ **8** (no BCN–PEG–BCN **2** was added, thus no reaction was possible). **c**, UV trace of SEC purification of Fab_HER2_–Fab_CD20_–Sia–biotin **21**. **d**, SDS–PAGE of SEC purification of Fab_HER2_–Fab_CD20_–Sia–biotin **21**. Lane 1: ladder. Lanes 2–3: aggregates. Lanes 4–9: Fab_HER2_–Fab_CD20_–Sia–biotin **21**. Lane 10: Fab_HER2_–Fab_CD20_–BCN **23**. Lanes 11–12: Sia–Tet–N_3_ **8**. **e**, LC-MS analysis of Fab_HER2_–Fab_CD20_–Sia–biotin **21**. Expected mass: 144,532 Da. Observed mass: 144,553 Da. **f**, SDS–PAGE of Fab_HER2_–Fab_CD3_–Sia–biotin CiTE **24** formation. Lane 1: ladder. Lane 2: crude Fab_HER2_–Fab_CD3_–Sia–biotin CiTE **24**. Lane 3: crude Fab_HER2_–Fab_CD3_–N_3_ **13**. **g**, UV trace of SEC purification of Fab_HER2_–Fab_CD3_–Sia–biotin CiTE **24**. **h**, SDS–PAGE of SEC purification of Fab_HER2_–Fab_CD3_–Sia–biotin CiTE **24**. Lane 1: ladder. Lane 2: crude Fab_HER2_–Fab_CD3_–Sia–biotin **24**. Lane 3–4: aggregates. Lanes 5–7: purified Fab_HER2_–Fab_CD3_–Sia–biotin CiTE **24** (+ Fab_HER2_–Fab_CD3_–Fab_HER2_ **25** impurity). Lanes 8–11: Fab_HER2_–Fab_CD3_–N_3_ **13**. Lanes 12–13: Sia–Tet–N_3_ **8**. **i**, LC-MS analysis of impure Fab_HER2_–Fab_CD3_–Sia–biotin CiTE **24**. Expected mass: 144,799 Da. Observed mass: 144,791 and 144,644 Da (Fab_HER2_–Fab_CD3_–Fab_HER2_ trispecific antibody **25** impurity, expected mass: 144,632 Da). **j**, UV trace of SEC purification of Fab_HER2_–Fab_CD3_–Sia–biotin CiTE **24** generated from SEC-purified Fab_HER2_–Fab_CD3_–N_3_ **13**. **k**, LC-MS analysis of Fab_HER2_–Fab_CD3_–N_3_ **13**. Expected mass: 96,493 Da. Observed mass: 96,506 Da. **l**, LC-MS analysis of Fab_HER2_–Fab_CD3_–BCN **26**. Expected mass: 97,065 Da. Observed mass: 97,081 Da. **m**, LC-MS analysis of pure Fab_HER2_–Fab_CD3_–Sia–biotin CiTE **24**. Expected mass: 144,799 Da. Observed mass: 144,825 Da. Generation of most Fab conjugates was carried out two or three times, yielding similar results. Each protein–protein construct was generated a single time unless otherwise stated.

With these encouraging preliminary results obtained, the generation of a Fab_HER2_–Fab_CD20_–Sia–biotin species **21** was attempted (Fig. 3a). SDS–PAGE analysis showed that the bsAb formation proceeded well and, after Fab_HER2_–Fab_CD20_–N_3_ **22** was reacted with BCN–PEG–BCN **2** (and excess small molecule removed after 6 h), Sia–Tet–N_3_ **8** and DBCO–biotin **5** addition led to consumption of Fab_HER2_–Fab_CD20_–BCN **23** and the appearance of a larger band (Fig. 3b). Interestingly, vigorous denaturing conditions (95 °C, 5 min) were required to increase the resolution of the gel. SEC purification showed that >80% conversion to the Fab_HER2_–Fab_CD20_–Sia–biotin **21** construct (11% yield from Fab_HER2_–BCN **10**) was achieved (Fig. 3c), which was encouraging compared to the best previous conversion of <50% (as detailed in the Supplementary Information). SDS–PAGE (Fig. 3d) and LC-MS analysis (Fig. 3e) confirmed the purity of the sample.

Following these encouraging results, the generation of Fab_HER2_–Fab_CD3_–Sia–biotin **24** was attempted via the same strategy. Unfortunately, in this case, bispecific formation also led to a notable amount of undesired Fab_HER2_–Fab_CD3_–Fab_HER2_ trispecific antibody **25**, as shown by SDS–PAGE (Fig. 3f). Although not impacting further reactions, as it is of a similar size to Fab_HER2_–Fab_CD3_–Sia–biotin **24**, SEC purification would not be able to separate them. As expected, addition of Sia–Tet–N_3_ **8** and DBCO–biotin **5** led to substantial consumption of Fab_HER2_–Fab_CD3_–BCN **26** (Fig. 3f), and SEC purification confirmed good conversion (~70%) of bsAb to product **24** (Fig. 3g). SDS–PAGE (Fig. 3h) and LC-MS analysis (Fig. 3i) confirmed formation of the product, although with ~15% Fab_HER2_–Fab_CD3_–Fab_HER2_ **25** impurity arising from the bsAb-formation step of the reaction, as discussed. This issue could be alleviated by either controlling the equivalents of Fabs to minimize the formation of trispecific antibody or scaling up the reaction and purifying the bsAb–N_3_ **13** by SEC before subsequent reactions. Alternatively, a dual purification approach with protein A and monomeric avidin agarose resin could be carried out, which should leave only species that contain both Fab_HER2_ (binds protein A) and Sia–biotin (binds avidin).

To address the purity issues of the final construct, the synthesis was repeated, this time using SEC-purified Fab_HER2_–Fab_CD3_–N_3_ **13** as described above (Fig. 2d–g). The portion of Fab_HER2_–Fab_CD3_–N_3_ **13** that was not biotinylated before was now treated with BCN–PEG–BCN **2** over 6 h. After removal of excess small molecule, the purity of the sample was confirmed by LC-MS (Fig. 3l), then Sia–Tet–N_3_ **8** and DBCO–biotin **5** were added, and the mixture was incubated for 20 h at 22 °C. After this time, the sample was SEC-purified (Fig. 3j) and subsequently the purity was confirmed by LC-MS analysis (Fig. 3m). Gratifyingly, clean Fab_HER2_–Fab_CD3_–Sia–biotin **24** (20% yield from bsAb–N_3_ **13**) was obtained.

**Fig. 4:**
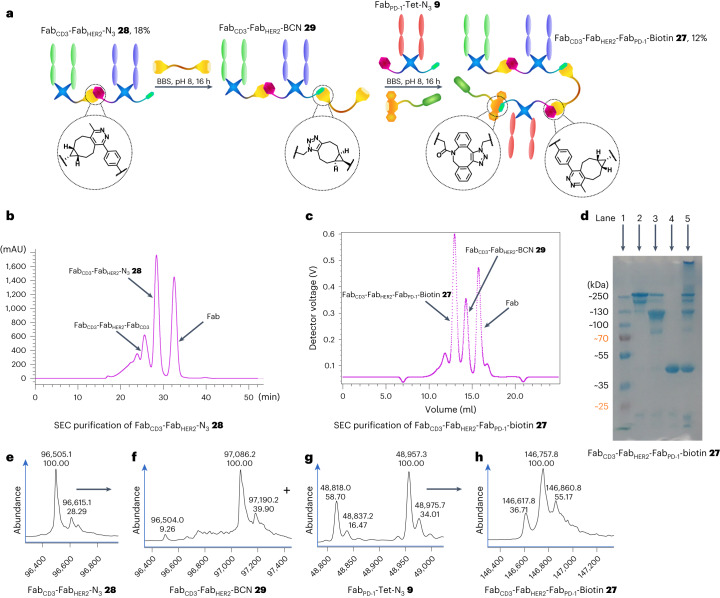
Synthesis of Fab_CD3_–Fab_HER2_–Fab_PD-1_–biotin CiTE **27**. **a**, Method for the synthesis of Fab_CD3_–Fab_HER2_–Fab_PD-1_–biotin CiTE **27**. Fab_CD3_–Fab_HER2_–N_3_ **28** was prepared as outlined before. This was then SEC-purified and reacted with BCN–PEG–BCN **2** to generate Fab_CD3_–Fab_HER2_–BCN **29**. Fab_PD-1_–Tet-N_3_ **9** and DBCO–biotin **5** were then added and reacted in situ to form Fab_CD3_–Fab_HER2_–Fab_PD-1_–biotin CiTE **27**, which was then isolated after SEC purification. **b**, UV trace of SEC purification of Fab_CD3_–Fab_HER2_–N_3_ **28**. **c**, UV trace of SEC purification of Fab_CD3_–Fab_HER2_–Fab_PD-1_–biotin CiTE **27**. **d**, SDS–PAGE analysis of Fab_CD3_–Fab_HER2_–Fab_PD-1_–biotin CiTE **27**. Lane 1: ladder. Lane 2: purified Fab_CD3_–Fab_HER2_–Fab_PD-1_–biotin CiTE **27**. Lane 3: left-over bsAb (Fab_CD3_–Fab_HER2_–BCN **29**) after SEC. Lane 4: left-over Fab (Fab_PD-1_–Tet–N_3_ **9**) after SEC. Lane 5: crude Fab_CD3_–Fab_HER2_–Fab_PD-1_–biotin CiTE **27** formation reaction. **e**, LC-MS analysis of Fab_CD3_–Fab_HER2_–N_3_ **28**. Expected mass: 96,496 Da and 96,610 Da. Observed mass: 96,505 Da and 96,615 Da. **f**, LC-MS analysis of Fab_CD3_–Fab_HER2_–BCN **29**. Expected mass: 97,068 Da and 97,182 Da. Observed mass: 97,086 and 97,190 Da. **g**, LC-MS analysis of Fab_PD-1_–Tet–N_3_ **9**. Expected mass: 48,820 Da and 48,959 Da. Observed mass: 48,818 Da and 48,957 Da. **h**, LC-MS analysis of Fab_CD3_–Fab_HER2_–Fab_PD-1_–biotin CiTE **27**. Expected mass: 146,610 Da, 146,749 Da and 146,858 Da. Observed mass: 146,618 Da, 146,758 Da and 146,861 Da. Generation of most Fab conjugates was carried out two or three times, yielding similar results. Each protein–protein construct was generated a single time unless otherwise stated.

To further demonstrate the modularity of the three-protein conjugation approach developed here and to generate an additional useful construct, the synthesis of a Fab_CD3_–Fab_HER2_–Fab_PD-1_–biotin CiTE **27** was attempted (Fig. 4a). The synthesis of a Fab_CD3_–Fab_HER2_–N_3_ bsAb **28** was carried out as before, although with the positions of the Fab_CD3_ and Fab_HER2_ arms swapped to showcase the flexibility of the strategy and investigate the effect of Fab placement within the construct on biological function. Following SEC purification (Fig. 4b), the purity of the construct was determined by means of SDS–PAGE (Fig. 4d) and LC-MS (Fig. 4e) analysis. The bsAb–N_3_ **28** was converted to Fab_CD3_–Fab_HER2_–BCN **29** with BCN–PEG–BCN **2**, as before (Fig. 4f). After removal of small molecule, Fab_PD-1_–Tet–N_3_ **9** (Fig. 4g) and DBCO–biotin **5** were added to form Fab_CD3_–Fab_HER2_–Fab_PD-1_–biotin CiTE **27** after SEC purification (12% yield from bsAb–N_3_ **28**, Fig. 4c). The purity of the construct was analysed via SDS–PAGE (Fig. 4d) and LC-MS (Fig. 4h). The SDS–PAGE analysis showed an additional fainter band beneath the main band, and the LC-MS spectrum (see Supplementary Information for the complete spectrum) contained additional peaks at lower masses in the raw data, in addition to the expected mass envelope. However, the deconvoluted spectrum showed primarily the expected masses (with the three major peaks arising from one-amino-acid variations in the precursor Fabs as discussed in the Supplementary Information). As the LC-MS suggests that there are no other major species in the 100–150 kDa range, we tentatively propose that the additional band in the SDS–PAGE could be due to incomplete denaturation of the construct or some other SDS–PAGE-derived artefact. The LC-MS raw data do show some smaller contaminant species, although these may be overrepresented as larger proteins (such as CiTE **27**) tend to ionize worse than smaller proteins under LC-MS conditions. This is further corroborated by the SDS–PAGE, which shows only very minor bands at a low molecular weight (Fig. 4d). Furthermore, the SEC UV trace of the purification also suggests a relatively homogeneous product by size, as the corresponding peak is narrow, with no visible shoulders (Fig. 4c). However, it must be noted that we cannot with confidence claim that CiTE **27** is completely pure. We have, however, demonstrated that the method can produce completely pure products, as is seen in the case of CiTE **24**. As this is a proof-of-concept work, we focused on rapid publication even with the caveats pertaining to the purity of CiTE **27**.

### Biological evaluation of CiTE constructs

**Fig. 5:**
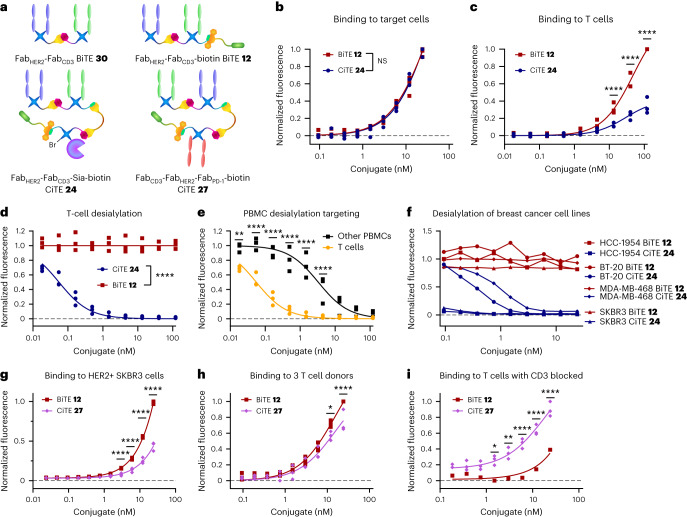
Biological testing of CiTE constructs **24** and **27**. **a**, Structures of the constructs used in the assay. **b**, Binding of Fab_HER2_–Fab_CD3_–Sia–biotin CiTE **24** and Fab_HER2_–Fab_CD3_–biotin BiTE **12** to HER2^+^ cancer cell lines (SKBR3, HCC1954, BT-20) detected by flow cytometry, normalized to maximum binding. NS, not significant. **c**, Binding of Fab_HER2_–Fab_CD3_–Sia–biotin CiTE **24** and Fab_HER2_–Fab_CD3_–biotin BiTE **12** to T cells from three donors detected by flow cytometry, normalized to maximum binding for each donor. **d**, Desialylation of T cells from three donors by Fab_HER2_–Fab_CD3_–Sia–biotin CiTE **24** and Fab_HER2_–Fab_CD3_–biotin BiTE **12**, normalized to untreated. **e**, Desialylation of T cells and PBMCs from three donors by Fab_HER2_–Fab_CD3_–Sia–biotin CiTE **24**, normalized to untreated. **f**, Desialylation of cancer cells by Fab_HER2_–Fab_CD3_–Sia–biotin CiTE **24**, normalized to untreated. **g**, Binding of Fab_CD3_–Fab_HER2_–Fab_PD-1_–biotin CiTE **27** and Fab_HER2_–Fab_CD3_–biotin BiTE **12** to the HER2^+^ SKBR3 cancer cell line detected by flow cytometry, normalized to maximum binding. **h**, Binding of Fab_CD3_–Fab_HER2_–Fab_PD-1_–biotin CiTE **27** and Fab_HER2_–Fab_CD3_–biotin BiTE **12** to T cells from three donors detected by flow cytometry, normalized to maximum binding for each donor. **i**, Binding of Fab_CD3_–Fab_HER2_–Fab_PD-1_–biotin CiTE **27** and Fab_HER2_–Fab_CD3_–biotin BiTE **12** to T cells from one donor, after CD3 blockade, detected by flow cytometry, normalized to maximum binding. Data are represented as individual data points from three replicates (except in **f** and for BiTE **12** binding in **i**, which are single data points without replicates). Statistical analysis was carried out with a two-way analysis of variance (ANOVA) followed by a post hoc Šídák’s multiple comparisons test with multiplicity-adjusted *P* values with *α* = 0.05. *P < 0.05, **P < 0.01, ***P < 0.001, ****P < 0.0001. For **a** and **c**, the differences were not significant and significant (****) at all concentrations, respectively. Curves are fitted with nonlinear regression with the following models: one site -- specific binding (**b**,**c**,**h**); [Inhibitor] versus response (three parameters) (**d**,**e**); [Agonist] versus response (three parameters) (**g**,**i**). List of *P* values for CiTE **24** versus BiTE **12** in **b**: 0.9985 (at 0.094 nM), >0.9999 (at 0.188 nM), 0.9860 (at 0.375 nM), >0.9999 (at 0.75 nM), 0.9316 (at 1.5 nM), >0.9999 (at 3 nM), 0.9303 (at 6 nM), 0.6189 (at 12 nM) and >0.9999 (at 24 nM). List of *P* values for CiTE **24** versus BiTE **12** in **c**: >0.9999 (at 0.018 nM), >0.9999 (at 0.055 nM), >0.9999 (at 0.165 nM), >0.9999 (at 0.494 nM), 0.9936 (at 1.48 nM), >0.2870 (at 4.44 nM), <0.0001 (at 13.3 nM), <0.0001 (at 40 nM) and <0.0001 (at 120 nM). List of *P* values for CiTE **24** versus BiTE **12** in **d**: <0.0001 (at 0.018 nM), <0.0001 (at 0.055 nM), <0.0001 (at 0.165 nM), <0.0001 (at 0.494 nM), <0.0001 (at 1.48 nM), <0.0001 (at 4.44 nM), <0.0001 (at 13.3 nM), <0.0001 (at 40 nM) and <0.0001 (at 120 nM). List of *P* values for T cell versus PBMC in **e**: 0.0044 (at 0.018 nM), <0.0001 (at 0.055 nM), <0.0001 (at 0.165 nM), <0.0001 (at 0.494 nM), <0.0001 (at 1.48 nM), <0.0001 (at 4.44 nM), 0.2744 (at 13.3 nM), 0.9392 (at 40 nM) and 0.9959 (at 120 nM). List of *P* values for CiTE **27** versus BiTE **12** in **g**: >0.9999 (at 0.023 nM), >0.9999 (at 0.047 nM), >0.9999 (at 0.094 nM), >0.9999 (at 0.188 nM), 0.9982 (at 0.375 nM), 0.7945 (at 0.75 nM), 0.1950 (at 1.5 nM), <0.0001 (at 3 nM), <0.0001 (at 6 nM), <0.0001 (at 12 nM) and <0.0001 (at 24 nM). List of *P* values for CiTE **27** versus BiTE **12** in **h**: >0.9999 (at 0 nM), >0.9999 (at 0.094 nM), 0.9991 (at 0.188 nM), 0.9998 (at 0.375 nM), 0.9987 (at 0.75 nM), 0.9756 (at 1.5 nM), 0.9834 (at 3 nM), 0.5546 (at 6 nM), 0.0116 (at 12 nM) and <0.0001 (at 24 nM). List of *P* values for CiTE **27** versus BiTE **12** in **i**: 0.9123 (at 0 nM), 0.6874 (at 0.188 nM), 0.8176 (at 0.375 nM), 0.3741 (at 0.75 nM), 0.0169 (at 1.5 nM), 0.0028 (at 3 nM), <0.0001 (at 6 nM), <0.0001 (at 12 nM) and <0.0001 (at 24 nM). Source data

With the CiTE constructs prepared, their biological activity was evaluated. Initially, the binding of Fab_HER2_–Fab_CD3_–Sia–biotin CiTE **24** (note, for all biological assays, pure CiTE **24** was used) to HER2^+^ cancer cells (SKBR3, HCC1954, BT-20) was measured via flow cytometry and shown to be not significantly different from the binding of Fab_HER2_–Fab_CD3_–biotin BiTE **12** to these cells (Fig. 5b). Next the binding assay was repeated on T cells, and here it was shown that the CD3 binding of CiTE **24** was significantly lower than that of BiTE **12** (Fig. 5c). We postulate that this may be due to the placement of the Fab_CD3_ moiety, as it is sandwiched between the other two protein components. This decreased binding is, however, not necessarily a drawback. In fact, weaker binding to T cells compared to HER2^+^ target cells could lead to better tumour-specificity and localization, and thus less systemic immune activation, lowering the risk of associated side effects such as cytokine release syndrome^27^. It was thus established that the two Fab components of CiTE **24** retained their biological activity (as it pertains to binding), so next the activity of the sialidase enzyme component was investigated. T cells or peripheral blood mononuclear cells (PBMCs) were incubated with CiTE **24** and BiTE **12**, and the cell-surface sialic acid content was measured. Although BiTE **12**, as expected, exhibited no sialidase activity (as it lacks the enzyme), CiTE **24** showed significant desialylation, with activity on T cells being more than an order of magnitude higher than off-target desialylation on other PBMCs (not expressing CD3; Fig. 5d,e). It is worth noting that visualization of the binding of CiTE **24** and BiTE **12** was carried out by incubation with a streptavidin Alexa Fluor 647 conjugate, thus also confirming that the biotin molecule attached to the constructs retained its binding to streptavidin, and showing why the capacity of the method for functionalization of these protein–protein constructs is beneficial. The desialylation of breast cancer cell lines (HCC1954, BT-20, MDA-MB-468 and SKBR3) by CiTE **24** was then investigated (Fig. 5f). BiTE **12** again exhibited no activity, whereas desialylation by CiTE **24** was dependent on HER2 expression, as HER2^hi^ cells (HCC1954 and SKBR3) were desialylated at lower concentrations than HER2^lo^ cells (BT-20, MDA-MB-468). The components of CiTE **24** (Fab_HER2_, Fab_CD3_, ST sialidase) thus all retained their relevant biological activity, despite the numerous enzymatic and chemical transformations carried out during construct assembly.

This testing of components was then carried out on Fab_CD3_–Fab_HER2_–Fab_PD-1_–biotin CiTE **27**. Although CiTE **27** showed binding to HER2^+^ target cells (SKBR3), it was significantly weaker than that of Fab_HER2_–Fab_CD3_–biotin BiTE **12**, corroborating the theory that the Fab sandwiched in the middle of the construct has lower binding strength, presumably due to the steric hindrance of the other two proteins on either side of it (Fig. 5g). Certainly, weaker target binding is not desirable in this case, so, in the future, a Fab_HER2_–Fab_CD3_–Fab_PD-1_–biotin CiTE would be a better candidate, with higher HER2 binding but the aforementioned lower (and beneficial) CD3 binding. Additionally, a Fab_PD-L1_ moiety would also be a more suitable way of targeting the PD-1/PD-L1 checkpoint, as PD-L1 is expressed on target cells, and PD-1 on effector cells; ideally effector-cell binding would only occur in the tumour environment. Unfortunately, our efforts to obtain clean PD-L1 Fab were unsuccessful, which is why Fab_PD-1_ was our protein of choice. The binding of CiTE **27** to T cells was also compared to that of BiTE **12**, and it was found that CiTE **27** bound T cells significantly more weakly at higher concentrations than BiTE **12** (Fig. 5h). However, this decrease in T cell binding was clearly less pronounced than in the case of CiTE **24**. This increased T cell binding of CiTE **27** compared to CiTE **24** may have been due to PD-1 binding, or the change in connectivity (Fab_CD3_ now being on the outside of the construct rather than in the middle) or a combination of both. Indeed, to investigate the PD-1 binding of CiTE **27**, T cells were pre-incubated with anti-CD3 mAbs, followed by incubation with varying concentrations of CiTE **27** or BiTE **12** (Fig. 5i). The binding of BiTE **12** clearly decreased comparatively and was found to be significantly lower than CiTE **27** under these conditions, suggesting that Fab_CD3_–Fab_HER2_–Fab_PD-1_–biotin CiTE **27** was indeed capable of binding to PD-1. Again, all binding studies here were carried out with the aid of a dye-tagged streptavidin, showing that the biotin molecule attached to CiTE **27** provided an important advantage for ease of analysis. The components of CiTE **27** (Fab_HER2_, Fab_CD3_ and Fab_PD-1_) thus also retained their binding activity, at least to an extent.

Finally, a T cell/HER2^+^ MDA-MB-231 cell line co-culture cell-kill assay was carried out to observe whether any efficacy increase can be attributed to the CiTE molecules compared to a conventional BiTE. The constructs were expected to bind to the HER2 receptor on the target cells and the CD3 receptor on the T cells (as shown above), re-directing the immune cells and leading to T cell-mediated cytotoxicity and death of the target cells. Furthermore, the effect of the checkpoint inhibitory modalities of the CiTEs (sialidase enzyme and Fab_PD-1_, respectively) could be investigated, that is, whether the CiTEs show enhanced cytotoxicity due to enhanced T cell activation promoted by checkpoint inhibition.

**Fig. 6:**
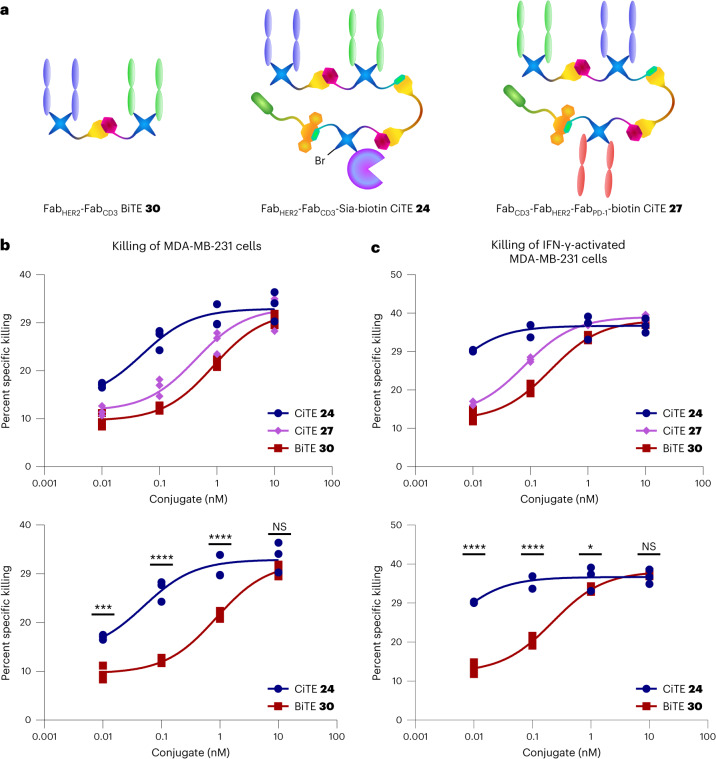
Cytotoxicity assay of Fab_HER2_–Fab_CD3_–Sia–biotin CiTE **24** and Fab_CD3_–Fab_HER2_–Fab_PD-1_–biotin CiTE **27**. **a**, Structures of the constructs used in the assay. **b**, Cytotoxicity assay of Fab_HER2_–Fab_CD3_–Sia–biotin CiTE **24** and Fab_CD3_–Fab_HER2_–Fab_PD-1_–biotin CiTE **27**. MDA-MB-231 cells were co-cultured with T cells from a single donor (E:T ratio of 2:1) and treated with 0.01–10 nM CiTE **24**, CiTE **27** or BiTE **30**. MDA-MB-231 viability was assessed 24 h following treatment via lactate dehydrogenase (LDH) assay. **c**, Cytotoxicity assay of Fab_HER2_–Fab_CD3_–Sia–biotin CiTE **24** and Fab_CD3_–Fab_HER2_–Fab_PD-1_–biotin CiTE **27**. MDA-MB-231 cells, pre-incubated with IFN-γ to induce PD-L1 expression, were co-cultured with T cells from a single donor (E:T ratio of 2:1) and treated with 0.01–10 nM CiTE **24**, CiTE **27** or BiTE **30**. MDA-MB-231 viability was assessed 24 h following treatment via LDH assay. Statistical analysis was carried out with a two-way ANOVA followed by a post hoc Tukey’s multiple comparisons test with multiplicity-adjusted *P* values with *α* = 0.05. *P < 0.05, **P < 0.01, ***P < 0.001, ****P < 0.0001. List of *P* values for CiTE **24** versus BiTE **30** in **b**: 0.0004 (at 0.01 nM), <0.0001 (at 0.1 nM), <0.0001 (at 1 nM) and 0.1791 (at 10 nM). List of *P* values for CiTE **24** versus BiTE **30** in **c**: <0.0001 (at 0.01 nM), <0.0001 (at 0.1 nM), 0.0333 (at 1 nM) and 0.7435 (at 10 nM). Data are represented as individual data points, from three replicates. Curves are fitted with nonlinear regression with the following model: [Agonist] versus response (three parameters). See Supplementary Information for the ANOVA table and comparisons between CiTE **27** and BiTE **30**, and CiTE **24** and CiTE **27**. Source data

Here, a non-biotinylated Fab_HER2_–Fab_CD3_ BiTE **30** (Supplementary Information provides synthesis details) was used to conserve biotinylated Fab_HER2_–Fab_CD3_–biotin BiTE **12** for studies where the biotin would be important for the visualization of binding. HER2^+^ MDA-MB-231 cells were either untreated or incubated with interferon gamma (IFN-γ) to induce PD-L1 expression. They were then co-cultured with T cells (effector:target (E:T) ratio of 2:1) and incubated with a range of concentrations of Fab_HER2_–Fab_CD3_ BiTE **30**, Fab_HER2_–Fab_CD3_–Sia–biotin CiTE **24** or Fab_CD3_–Fab_HER2_–Fab_PD-1_–biotin CiTE **27**. In the case of both IFN-γ-treated and untreated cells, both CiTEs, as a trend, showed greater cytotoxicity than BiTE **30** in the concentration range 0.01–1 nM (Fig. 6b,c). This increased efficacy is in line with the findings on the previously reported engineered anti-CD33/anti-CD3/PD-1_ex_ CiTE^21^. Another general trend was the higher cytotoxicity observed in the case of IFN-γ-treated MDA-MB-231 cells.

PD-1 blocking CiTE **27** was slightly more potent at lower concentrations than BiTE **30**, especially when the MDA-MB-231 cells were treated with IFN-γ to induce PD-L1 expression (Supplementary Information). However, sialidase-containing CiTE **24** was significantly more active at lower concentrations than either CiTE **27** or BiTE **30**, suggesting that, under these conditions, desialylation is synergistic with T cell engagement, and more so than PD-1/PD-L1 checkpoint blockade (Fig. 6b,c; the Supplementary Information provides additional comparisons). The catalytic activity of sialidase enzyme contrasted with the stoichiometric nature of PD-1 blockade could perhaps play a role in how active CiTE **24** was at low concentrations compared to CiTE **27**. Indeed, the cancer-cell and T cell desialylation data discussed previously (Fig. 5d,f) suggest that all sialic acid is removed by between 0.1 nM and 1 nM CiTE **24**, facilitating T cell-mediated cytotoxicity.

## Conclusions and outlook

In summary, a method has been developed for the chemical generation of functionalized three-protein constructs. CiTE molecules with either an ST sialidase enzyme for removal of immunosuppressive sialic acid glycans from target and effector cells^25^ or with an anti-PD-1 Fab checkpoint inhibitor^21^ attached were synthesized along with relevant controls. The syntheses were carried out via tetrazine–BCN SPIEDAC click chemistry for protein–protein conjugation, and each CiTE had a biotin small molecule also conjugated via SPAAC for imaging and/or purification. These CiTE molecules were then tested for their biological activity. Owing to its modularity, this method could be applied to the generation of a variety of three-protein constructs. BiTEs could be conjugated to different checkpoint inhibitors (for example, CTLA-4 or ICOS) or cytokines (for example, interleukin-2)^28^, the selectivity of the construct could be improved by targeting two separate tumour-associated receptors in addition to CD3, or target-independent immune activators could be developed to reactivate exhausted T cells, regardless of cancer indication^29^. The method has the added flexibility of an inherent handle for the attachment of small molecules, such as biotin, fluorophores, cytotoxins, half-life extenders or activity-masking moieties^30,31^. However, the constructs described here do not possess an Fc moiety and would be expected to have a shorter half-life in vivo due to a lack of FcRn-mediated recycling^32^. If this reduced half-life is shown to be detrimental in vivo, the strategy would need to be adapted to incorporate an Fc (for example, via a SynAb-checkpoint-inhibitor conjugate^20^) or other half-life extender, such as albumin^33^ or an albumin-binding motif^34^. The strategy also, to some extent, enables control over the binding profile of the constructs, as it seems that the Fab moiety sandwiched in the middle of the three-protein species has reduced ability to bind its target, presumably due to steric hindrance. This could be exploited to minimize unwanted binding, and thus potentially reduce side effects. The method is also rapid (the conjugates can be prepared starting from mAbs within a 5–7-day timescale) and modular (works with most mAbs and cysteine-mutant proteins). It could thus be very useful in hit identification, where a large number of constructs with various protein combinations are generated from a pool of biomacromolecules, for example, in a 96-well plate. These crude constructs could then be screened for biological activity and the most promising hits scaled up for further testing. The scalability and developability of this strategy should, however, be investigated, as, based on current information, it is hard to judge whether it would be feasible to make the shift to large-scale industrial production. That being said, we do not see any inherent reason it could not, provided the process can be streamlined to minimize protein loss during purification steps, as the chemical reactions themselves all proceed with excellent conversions.

The generated constructs, Fab_HER2_–Fab_CD3_–Sia–biotin CiTE **24** and Fab_CD3_–Fab_HER2_–Fab_PD-1_–biotin CiTE **27**, along with simpler two-protein BiTE constructs had their biological activities investigated. The constituent parts were shown to retain their biological function (although binding was impaired in some cases). The CiTEs were then shown to be significantly more effective than the corresponding BiTE **30** at promoting T cell-mediated HER2^+^ cell death. Although the increase in the efficacy of PD-1-blocking CiTE **27** was perhaps not astounding in its magnitude, there was significant benefit in adding the checkpoint inhibitory modality to a BiTE scaffold, even under these relatively unoptimized conditions. The sialidase-containing CiTE **24**, however, had robustly increased cytotoxic activity (by about an order of magnitude) at lower concentrations than BiTE **30**. Carrying out more in-depth biological assays (including in vivo assays and testing different HER2^+^ cancer-cell lines) was beyond the scope of this chemistry-focused project. However, as other groups have demonstrated the synergy between checkpoint inhibition and T cell engagement^21^, we believe this exciting angle of immunomodulation should be explored further, especially as this work goes on to show that sialic-acid removal is synergistic with BiTE treatment in vitro. Investigating such a three-protein CiTE in vivo will be important to understand whether it is beneficial over administering the checkpoint inhibitor and the BiTE separately, as unwanted off-site checkpoint inhibitor-mediated immune activation can be minimized. Furthermore, we hope we have provided a method that can be applied to generate further functionalized three-protein constructs. We also hope we have demonstrated the power of bioorthogonal chemical strategies for protein–protein conjugation. This area of research has been gaining momentum recently^6,7^, but there is much untapped potential that is still waiting to be uncovered.

### Reporting summary

Further information on research design is available in the Nature Portfolio Reporting Summary linked to this Article.

## Online content

Any methods, additional references, Nature Portfolio reporting summaries, source data, extended data, supplementary information, acknowledgements, peer review information; details of author contributions and competing interests; and statements of data and code availability are available at 10.1038/s41557-023-01280-4.

### Supplementary information

Supplementary Information Scheme 1, Figs. 1–5, Tables 1–3, discussion, experimental section, NMR spectra, LC-MS spectra.

Reporting Summary

### Source data

Source Data Fig. 5 .csv files containing the raw data for each section of Fig. 5. Prism (.pzfx) files containing the raw data and statistical analyses for each section of Fig. 5.

Source Data Fig. 6 .csv files containing the raw data for each section of Fig. 6. Prism (.pzfx) files containing the raw data and statistical analyses for each section of Fig. 6.

## Data Availability

The detailed procedures required to duplicate this work are available in the Supplementary Information along with full LC-MS and NMR spectra where appropriate. The numerical data from the in vitro cell assays are available as .csv and Prism files. Any additional data or unique materials (through a materials transfer agreement) are available from the corresponding authors on reasonable request. Source data are provided with this paper.

## References

[1] Gera N (2022). The evolution of bispecific antibodies. Expert Opin. Biol. Ther..

[2] Budde LE (2022). Safety and efficacy of mosunetuzumab, a bispecific antibody, in patients with relapsed or refractory follicular lymphoma: a single-arm, multicentre, phase 2 study. Lancet Oncol..

[3] Hong Y, Nam SM, Moon A (2023). Antibody–drug conjugates and bispecific antibodies targeting cancers: applications of click chemistry. Arch. Pharm. Res..

[4] Husain B, Ellerman D (2018). Expanding the boundaries of biotherapeutics with bispecific antibodies. BioDrugs.

[5] Thoreau F, Chudasama V (2022). Enabling the next steps in cancer immunotherapy: from antibody-based bispecifics to multispecifics, with an evolving role for bioconjugation chemistry. RSC Chem. Biol..

[6] Szijj P, Chudasama V (2021). The renaissance of chemically generated bispecific antibodies. Nat. Rev. Chem..

[7] Taylor RJ, Geeson MB, Journeaux T, Bernardes GJL (2022). Chemical and enzymatic methods for post-translational protein-protein conjugation. J. Am. Chem. Soc..

[8] Moura A, Savageau MA, Alves R (2013). Relative amino acid composition signatures of organisms and environments. PLoS ONE.

[9] Khalili H (2013). Fab-PEG-Fab as a potential antibody mimetic. Bioconjug. Chem..

[10] Hull EA (2014). Homogeneous bispecifics by disulfide bridging. Bioconjug. Chem..

[11] Forte N (2018). Tuning the hydrolytic stability of next generation maleimide cross-linkers enables access to albumin-antibody fragment conjugates and tri-scFvs. Bioconjug. Chem..

[12] Patterson JT (2017). PSMA-targeted bispecific Fab conjugates that engage T cells. Bioorg. Med. Chem. Lett..

[13] Patterson JT (2017). Chemically generated IgG2 bispecific antibodies through disulfide bridging. Bioorg. Med. Chem. Lett..

[14] Maruani A (2020). A plug-and-play approach for the de novo generation of dually functionalized bispecifics. Bioconjug. Chem..

[15] Lee MTW, Maruani A, Chudasama V (2016). The use of 3,6-pyridazinediones in organic synthesis and chemical biology. J. Chem. Res..

[16] Bahou C (2018). Highly homogeneous antibody modification through optimisation of the synthesis and conjugation of functionalised dibromopyridazinediones. Org. Biomol. Chem..

[17] Robinson E (2017). Pyridazinediones deliver potent, stable, targeted and efficacious antibody-drug conjugates (ADCs) with a controlled loading of 4 drugs per antibody. RSC Adv..

[18] Lee MTW, Maruani A, Baker JR, Caddick S, Chudasama V (2016). Next-generation disulfide stapling: reduction and functional re-bridging all in one. Chem. Sci..

[19] Maruani A (2015). A mild TCEP-based *para*-azidobenzyl cleavage strategy to transform reversible cysteine thiol labelling reagents into irreversible conjugates. Chem. Commun..

[20] Thoreau F (2023). Modular chemical construction of IgG-like mono- and bispecific synthetic antibodies (SynAbs). ACS Cent. Sci.

[21] Herrmann M (2018). Bifunctional PD-1 × αCD3 × αCD33 fusion protein reverses adaptive immune escape in acute myeloid leukemia. Blood.

[22] Rader C (2020). Bispecific antibodies in cancer immunotherapy. Curr. Opin. Biotechnol..

[23] Bukhari A, Lee ST (2019). Blinatumomab: a novel therapy for the treatment of non-Hodgkin’s lymphoma. Expert Rev. Hematol..

[24] Krupka C (2016). Blockade of the PD-1/PD-L1 axis augments lysis of AML cells by the CD33/CD3 BiTE antibody construct AMG 330: reversing a T-cell-induced immune escape mechanism. Leukemia.

[25] Gray MA (2020). Targeted glycan degradation potentiates the anticancer immune response in vivo. Nat. Chem. Biol..

[26] Baalmann M (2020). A bioorthogonal click chemistry toolbox for targeted synthesis of branched and well-defined protein-protein conjugates. Angew. Chem. Int. Ed..

[27] Strohl WR, Naso M (2019). Bispecific T-cell redirection versus chimeric antigen receptor (CAR)-T cells as approaches to kill cancer cells. Antibodies.

[28] Neri D (2019). Antibody-cytokine fusions: versatile products for the modulation of anticancer immunity. Cancer Immunol. Res..

[29] Edgar LJ (2021). Sialic acid ligands of CD28 suppress costimulation of T cells. ACS Cent. Sci..

[30] Autio KA, Boni V, Humphrey RW, Naing A (2020). Probody therapeutics: an emerging class of therapies designed to enhance on-target effects with reduced off-tumor toxicity for use in immuno-oncology. Clin. Cancer Res..

[31] Lucchi R, Bentanachs J, Oller-Salvia B (2021). The masking game: design of activatable antibodies and mimetics for selective therapeutics and cell control. ACS Cent. Sci..

[32] Ward ES, Ober RJ (2018). Targeting FcRn to generate antibody-based therapeutics. Trends Pharmacol. Sci..

[33] Mandrup OA (2021). Programmable half-life and anti-tumour effects of bispecific T-cell engager-albumin fusions with tuned FcRn affinity. Commun. Biol..

[34] Liu L (2017). Albumin binding domain fusing R/K-X-X-R/K sequence for enhancing tumor delivery of doxorubicin. Mol. Pharm..

