# Supplementary material for: Chemical generation of checkpoint inhibitory T cell engagers for the treatment of cancer
Source: Nat Chem. Author manuscript; Available in PMC 2024 Feb 16. (PMC10624612; doi:10.1038/s41557-023-01280-4)
Supplement: supplementary information [file NIHMS1922194-supplement-supplementary_information.pdf]

nature chemistry

Article https://doi.org/10.1038/s41557-023-01280-4

Chemical generation of checkpoint inhibitory T cell engagers for the treatment of cancer

In the format provided by the authors and unedited

1. Table of contents **1**

2. Foreword **2**

3. Synthesis of BCN-PEG-BCN 2 .... **2**

4. Generation of protein fragments via enzymatic digestion **2**

5. Generation of model Fab_{HER2}-Fab_{CD3} BiTE 30 .... **5**

6. Synthesis of a model trispecific antibody, Fab_{HER2}-Fab_{CD20}-Fab_{EGFR} tsAb S33 .... **7**

7. Construction of improved bispecific T cell engager three-protein constructs **8**

8. Experimental Section **12**

8.2. Synthetic chemistry section 12

8.3. Chemical biology section 25

8.4. Cell Biology Section 36

9. NMR spectra **42**

10. LC-MS spectra **58**

11. References **96**

2. Foreword

This document will start by detailing the synthesis of BCN-PEG-BCN **2** molecule, followed by a section on the generation of the Fab modalities required for three-protein assembly. Next, the initial experiments on three-protein assembly will be detailed. These sections were written manuscript-style, however, as they detail proof-of-concept and optimization steps, or starting material-generation steps, it was felt that including them in the manuscript would be detrimental to the cohesion of the paper and increase the length and complexity for not enough tangible benefit. However, the authors believe that there is merit in reporting these steps in detail here as they are either key for the successful reproduction of this work or provide an interesting alternative method for the generation three-protein constructs with chemistry, even if the method reported in the manuscript was found to be superior.

Finally, the experimental section will describe the methods employed for the generation of the data presented here and in the manuscript.

3. Synthesis of BCN-PEG-BCN **2**

To aid in the modular generation of three-protein constructs, the synthesis of a BCN-PEG-BCN **2** molecule was carried out by reacting 2.1 eq. BCN-NHS carbonate with a bis-amine PEG in DCM in the presence of base (Scheme 1). After column purification, compound **2** was isolated in 42% yield.

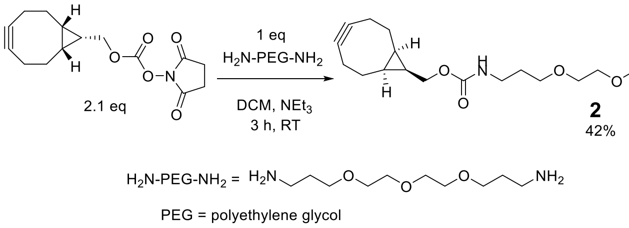

Scheme 1 | **Synthesis of BCN-PEG-BCN.**
The synthesis of BCN-PEG-BCN **2**.

4. Generation of protein fragments via enzymatic digestion

To be able to assemble multi-protein constructs, the generation of antibody antigen-binding fragment (Fab) building blocks had to be carried out. The commercially available parent monoclonal antibodies (mAbs) were subjected to enzymatic digestion, usually *via* the papaya cysteine protease, papain. The generation of these Fabs will be detailed in the sections below.

An anti-EGFR mAb (Cetuximab), an anti-PD-1 mAb, an anti-human ICOS mAb and an anti-CTLA-4 mAb were digested to the corresponding Fab_{EGFR} **S17**, Fab_{PD-1} **7**, Fab_{ICOS} **S20** and Fab_{CTLA-4} **S19**, respectively, *via* the standard antibody digestion protocol (Figure 1/A) and after protein A purification the purities were confirmed by SDS-PAGE (Figure 1/D,H) and LC-MS (Figure 1/J,N-P). The digestion of an anti-PD-L1 mAb

was also carried out, but the resulting Fab_{PD-L1} and Fc_{PD-L1} species could not be separated *via* protein A, protein A/G or protein L purification (data not shown).

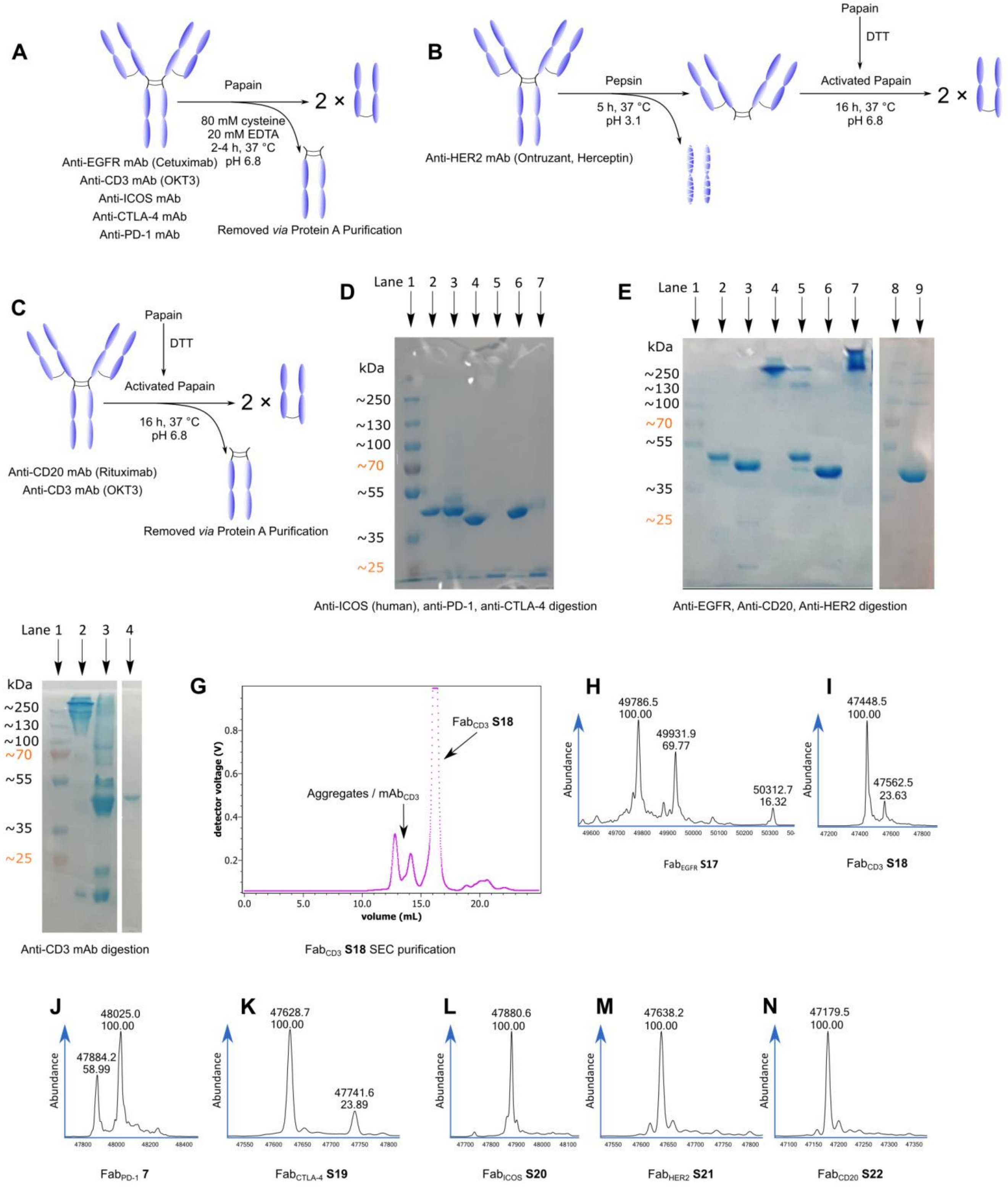

Figure 1 | **Enzymatic digestion of monoclonal antibodies (mAbs) to their corresponding fragments antigen binding (Fabs).**

A | Standard mAb digestion protocol used in the case of anti-EGFR mAb (Cetuximab), anti-CD3 mAb (OKT3), anti-human ICOS mAb, anti-CTLA-4 mAb, anti-PD-1 mAb. Papain, activated with 80 mM cysteine *in situ,* was used to generate Fab and Fc fragments of the parent mAb. These were then separated by

protein A purification. **B |** Digestion protocol used in the case of anti-CD20 mAb (Rituximab) and initially in the case of anti-CD3 mAb (OKT3). Subsequently the protocol outlined in Figure 1/A was used for anti-CD3 digestion. Papain, pre-activated with 10 mM DTT, was used to generate Fab and Fc fragments of the parent mAb. These were then separated by protein A purification. **C |** Protocol used for digestion of anti-HER2 mAb (Trastuzumab, Herceptin, Ontruzant). The Fc fragment was removed by pepsin digestion as the Fab of Trastuzumab binds protein A, thus making it impossible to separate the Fab and Fc that way. After the generation of $F(ab')_2$ fragments, pre-activated papain (with DTT) was used to remove the hinge region and furnish clean Fab_{HER2} **S21**. **D |** SDS-PAGE of Fab_{ICOS} **S20**, Fab_{PD-1} **7**, and Fab_{CTLA-4} **S19** digestion. Lane 1: Ladder. Lane 2: Fab_{ICOS} **S20**. Lane 3: Fc_{ICOS}. Lane 4: Fab_{PD-1} **7**. Lane 5: Fc_{PD-1}. Lane 6: Fab_{CTLA-4} **S19**. Lane 7: Fc_{CTLA-4}**. E |** SDS-PAGE of Fab_{HER2} **S21**, Fab_{CD20} **S22**, Fab_{EGFR} **S17** digestion. Lane 1 & 8: Ladder. Lane 2: Fc_{EGFR}. Lane 3: Fab_{EGFR} **S17**. Lane 4: mAb_{EGFR} (Cetuximab). Lane 5: Fc_{CD20} **S23**. Lane 6: Fab_{CD20} **S22**. Lane 7: mAb_{CD20} (Rituximab). Lane 9: Fab_{HER2} **S21**. Lanes 1-7 are from a different gel from lanes 8 & 9. **F |** SDS-PAGE of digestion and purification of Fab_{CD3} **S18**. SEC purification afforded clean Fab_{CD3} **S18**. Lane 1: Ladder. Lane 2: mAb_{CD3}. Lane 3: Crude digested mAb_{CD3} (OKT3). Lane 4: SEC purified Fab_{CD3} **S18**. All lanes are from the same gel, with irrelevant lanes removed from in between lanes 3 & 4. **G |** SEC UV trace of purification of Fab_{CD3} **S18**. **H |** LC-MS analysis of Fab_{EGFR} **S17**. Expected mass: 49788 and 49933 Da (glycoforms). Observed Mass: 49786 and 49933 Da (Δ = 147 Da, Fucose). **I |** LC-MS analysis of Fab_{CD3} **S18**. Observed mass: 47449 Da and 47563 Da (Δ = 114 Da, Asn). **J |** LC-MS analysis of Fab_{PD-1} **7**. Observed mass: 47884 Da and 48025 Da (Δ = 141 Da, His/Phe). **K |** LC-MS analysis of Fab_{CTLA-4} **S19**. Observed mass: 47629 Da and 47742 Da (Δ = 113 Da, Ile/Leu). **L |** LC-MS analysis of Fab_{ICOS} **S20**. Observed mass: 47881 Da. **M |** LC-MS analysis of Fab_{HER2} **S21**. Expected mass: 47638 Da. Observed mass: 47639 Da. **N |** LC-MS analysis of Fab_{CD20} **S22**. Expected mass: 47181 Da. Observed mass: 47180 Da. **| Relevant amino acid residue masses. |** Isoleucine/Leucine: 113.08 Da, Asparagine: 114.04 Da, Aspartic acid: 115.02 Da, Histidine: 137.06 Da, Phenylalanine: 147.07 Da. **Relevant glycan residue masses. |** Fucose: 146.06 Da. **|** Generation of most Fabs was carried out at least 2-3 times yielding similar results.

Anti-HER2 mAb (Herceptin or Ontruzant) was pre-digested with pepsin to remove the Fc region and then digested with pre-activated immobilized papain (with DTT) to afford the Fab fragment (Fab_{HER2} **S21**) in 32% yield, according to a previously published protocol (Figure 1/B).[1] This was necessary, as protein A binds to Fab_{HER2} **S21** in addition to Fc_{HER2}. Anti-CD20 mAb (Rituximab) was digested with pre-activated immobilized papain (with DTT) and then then purified with a protein A column, to yield anti-CD20 Fab (Fab_{CD20} **S22**) and anti-CD20 Fc (Fc_{CD20} **S23**, Figure 1/C). The purity of the Fabs and Fc_{CD20} **S23** was confirmed by SDS-PAGE (Figure 1/H) and LC-MS (Figure 1/Q-S).

Anti-CD mAb (OKT3) antibody was initially digested as mAb_{CD20} was with pre-activated papain, except the reaction was allowed to progress over 30 h (Figure 1/C), and instead of protein A purification, SEC purification was used (Figure 1/I). The purity of Fab_{CD3} **S18** was confirmed by SDS-PAGE (Figure 1/G) and LC-MS (Figure 1/M). Later the digestion was also repeated with the standard digestion protocol followed by protein A purification and was shown to yield similar results, over a shorter timeframe, thus for future digestions this protocol was used (Figure 1/A).

5. Generation of model Fab_{HER2}-Fab_{CD3} BiTE **30**

With the Fabs having now been produced, and the small molecules necessary already available from previous work,[2–4] the synthesis of a model BiTE was attempted from Fab_{HER2} **S21** and Fab_{CD3} **S18**. This would be the 1st BiTE prepared via the pyridazinedione (PD) method and would also serve as a suitable control for the three-protein CiTEs. Fab_{HER2} **S21** and Fab_{CD3} **S18** were reduced with TCEP, 20 and 60 eq., respectively, over 2 h at pH 8, 37 °C. After removal of excess TCEP (ultrafiltration), Br_2PD-BCN **3** was added to the reduced Fab_{HER2} **S21** solution and Br_2PD-Tet **S9** to the reduced Fab_{CD3} **S18** solution. The reaction was carried out over 90 min at pH 8, 37 °C (Figure 2/A). After removal of small molecule (ultrafiltration), the PD-modified, click-enabled Fab_{HER2}-BCN **10** and Fab_{CD3}-Tet **S29** were acquired. These were then reacted with each other in a 1:1.2 ratio (Figure 2/C) in acetate buffer (pH = 5) over 16 h at 30 °C. SDS-PAGE showed formation of a band of the expected size (Figure 2/D). N.B., antibody species consistently migrate slower on SDS-PAGE gels than the ladder would indicate, thus the band appearing at ~130 kDa according to the ladder, is actually the bsAb, which is ~97 kDa. Similarly, mAbs usually appear at ~250 kDa. Presumably this is due to incomplete denaturation, perhaps as a result of the disulfide bonds keeping the structure intact to some degree. SEC purification was then carried out to furnish clean Fab_{HER2}-Fab_{CD3} BiTE **30** (Figure 2/E) as confirmed by LC-MS analysis (Figure 2/F).

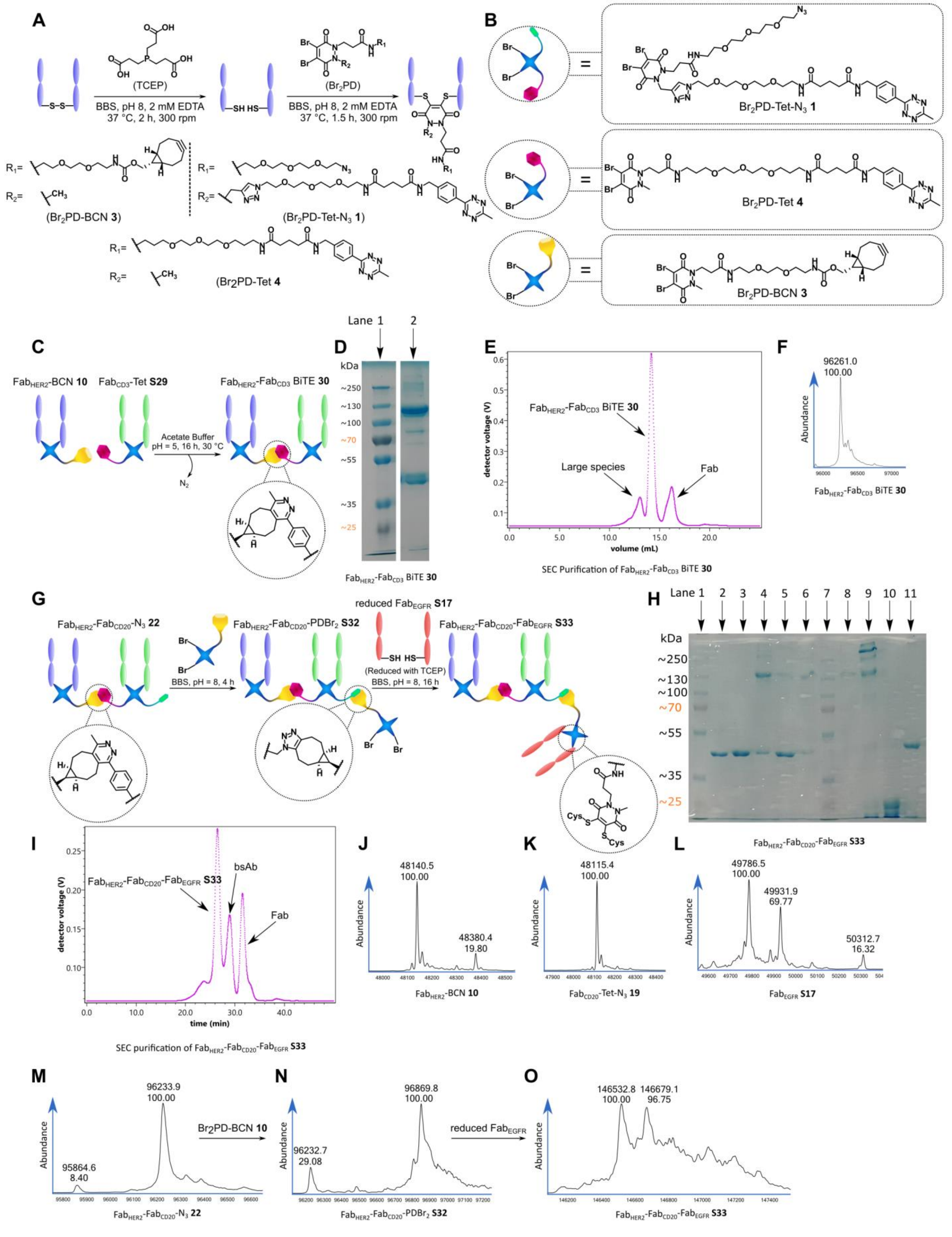

Figure 2 | **Generation of a bispecific T cell-engager (BiTE) Fab_{HER2}-Fab_{CD3} 30 and a trispecific antibody Fab_{HER2}-Fab_{CD20}-Fab_{EGFR} tsAb S33.**

A | Method for modifying Fabs with dibromo pyridazinediones (Br_2PDs). The Fab is reduced with TCEP (20-60 eq.). After removal of excess TCEP the Br_2PD is added (5-20 eq.) to form the Fab-PD conjugate. This way different click handles, or combinations thereof can be installed on the protein (e.g., bicyclononyne (BCN), tetrazine, or tetrazine and azide both).[2,3] **B |** The PDs used for the generation of bispecific and trispecific antibodies; Br_2PD-Tet **4**, Br_2PD-BCN **3**, and Br_2PD-Tet-N_3 1. **C |** Method for generating Fab_{HER2}-Fab_{CD3} **30** with PDs. Fab_{HER2}-BCN 10 and Fab_{CD3}-Tet **S29** are reacted with each other in a 1:1.2 ratio to form Fab_{HER2}-Fab_{CD3} **30** after SEC purification. **D |** SDS-PAGE of formation of Fab_{HER2}-Fab_{CD3} BiTE **30**. **Lane 1:** Ladder. **Lane 2:** Crude Fab_{HER2}-Fab_{CD3} BiTE **30**. Both lanes are from the same gel, with irrelevant lanes removed from in between lanes 1 & 2. **E |** UV trace of SEC purification of Fab_{HER2}-Fab_{CD3} BiTE **30**. **F |** LC-MS analysis of Fab_{HER2}-Fab_{CD3} BiTE **30**. Expected mass: 96256 Da. Observed mass: 96261 Da. **G |** Method for generating trispecific antibodies with PDs. Fab_{HER2}-BCN 10 and Fab_{CD20}-Tet-N_3 19 were reacted with each other in a 1:1.4 ratio to form Fab_{HER2}-Fab_{CD20}-N_3 **22**. This was purified by protein A purification and subsequently reacted with Br_2PD-BCN **3** to from Fab_{HER2}-Fab_{CD20}-$PDBr_2$ **S32**. To this was then added reduced Fab_{EGFR} **S17** to generate Fab_{HER2}-Fab_{CD20}-Fab_{EGFR} **S33** after SEC purification. **H |** SDS-PAGE of formation of Fab_{HER2}-Fab_{CD20}-Fab_{EGFR} trispecific antibody (tsAb) **S33**. **Lanes 1 & 7:** Ladder. **Lane 2:** Fab_{CD20} **S22**. **Lane 3:** Fab_{HER2} **S21**. **Lane 4:** Crude Fab_{HER2}-Fab_{CD20}-N_3 bsAb **22**. **Lane 5:** Fab_{CD20}-Tet-N_3 19 recovered after protein A purification. **Lane 6:** Purified Fab_{HER2}-Fab_{CD20}-N_3 bsAb **22**. **Lane 8:** Fab_{HER2}-Fab_{CD20}-$PDBr_2$ bsAb **S32**. **Lane 9:** Crude Fab_{HER2}-Fab_{CD20}-Fab_{EGFR} tsAb **S33**. **Lane 10:** Reduced Fab_{EGFR} **S17**. **Lane 11:** Fab_{EGFR} **S17**. **I |** UV trace of SEC purification of Fab_{HER2}-Fab_{CD20}-Fab_{EGFR} tsAb **S33**. **J |** LC MS analysis of Fab_{HER2}-BCN 10. Expected mass: 48140 Da. Observed mass: 48141 Da. **K |** LC-MS analysis of Fab_{CD20}-Tet-N_3 19. Expected mass: 48816 Da. Observed: 48115 Da. **L |** LC-MS analysis of Fab_{EGFR} **S17**. Expected mass: 49788 and 49933 Da (glycoforms). Observed mass: 49786 and 49933 Da (Δ = 147 Da, Fucose). **M |** LC-MS analysis of Fab_{HER2}-Fab_{CD20}-N_3 bsAb **22**. Expected mass: 96229 Da. Observed mass: 96234 Da. **N |** LC-MS analysis of Fab_{HER2}-Fab_{CD20}-$PDBr_2$ bsAb **S32**. Expected mass: 96891 Da. Observed mass: 96870 Da and 96234 Da (Fab_{HER2}-Fab_{CD20}-N_3 **22** starting material). **O |** LC-MS analysis of Fab_{HER2}-Fab_{CD20}-Fab_{EGFR} tsAb **S33**. Expected mass: 146512 Da and 146659 Da. Observed mass: 146530 Da and 146677 Da. **|** Generation of most Fab conjugates was carried out 2-3 times yielding similar results. Each protein–protein construct was generated a single time unless otherwise stated.

6. Synthesis of a model trispecific antibody, Fab_{HER2}-Fab_{CD20}-Fab_{EGFR} tsAb **S33**

Next the formation of a trispecific antibody, as a model three-protein construct, was attempted. First, Fab_{HER2}-BCN **10** and Fab_{CD20}-Tet-N_3 **19** were prepared. These were then combined in a 1:1.4 (Fab_{HER2}:Fab_{CD20}) ratio and incubated for 16 h at 22 °C and pH 5. The crude product was then purified by protein A to remove excess Fab_{CD20} **S22**, as Fab_{HER2} **S21** binds protein A, while Fab_{CD20} **S22** does not. The purity of the resulting Fab_{HER2}-Fab_{CD20}-N_3 **13** was confirmed by LC-MS (Figure 2/M). Next, 10 eq. of Br_2PD-BCN **3** was added to click to Fab_{HER2}-Fab_{CD20}-N_3 **13** to prepare Fab_{HER2}-Fab_{CD20}-$PDBr_2$ **S32** over 4 h at pH 8. After this time, the excess small molecule was removed, and the resulting construct analysed by LC-MS (Figure 2/N). Reduced Fab_{EGFR} **S17** was then added, and the reaction incubated at 37 °C, pH 8 overnight to yield crude Fab_{HER2}-Fab_{CD20}-Fab_{EGFR} trispecific antibody (tsAb) **S33** (Figure 2/G). Formation of a ~150 kDa species was confirmed by SDS-PAGE (Figure 2/H). This was then subjected to SEC purification (Figure 2/I)

to yield purified Fab_{HER2}-Fab_{CD20}-Fab_{EGFR} tsAb **S33**, the mass of which was confirmed by LC-MS analysis (Figure 2/O).

7. Construction of improved bispecific T cell engager three-protein constructs

With a method for the generation of a trispecific antibody (tsAb) in hand, it was decided to try and synthesize a more therapeutically relevant set of molecules. To this end, a bispecific T cell engager (BiTE)[5] platform comprised of the Fab arm of an anti-CD3 antibody (clone: OKT3, for engaging and activating T cells) **S18** and the Fab arm of an anti-HER2 antibody (Trastuzumab, for directing the immune cells to the cancer target) **S21** was generated with an azide functionality available for further attachment. To this bsAb, initially the attachment of the enzyme *Salmonella typhimurium* Sialidase (ST Sia) **6**, and later of an anti-PD-1 Fab **7** was attempted, to generate checkpoint inhibitory T cell-engagers (CiTEs). Sialidase removes sialic acid from the surface of cancer and immune cells, removing an immune checkpoint, leading to increased T cell activation,[6,7] and hopefully improved BiTE efficacy. Similarly, an anti-PD-1 Fab would block the PD-1/PD-L1 immune checkpoint thus acting as a checkpoint-inhibitor, hopefully leading to increased target cell-killing.[8] The generation of these three-protein conjugate CiTEs along with suitable controls will be detailed in the following sections.

Fab_{CD3} **S18** and ST Sia **6** were reduced with TCEP (20 eq., 2 h) and reacted with Br_2PD-BCN **3** (10 eq., 90 min). Both reactions proceeded well as analysed by SDS-PAGE (Figure 3/C) and LC-MS data showed clean conversion of both sialidase **6** and Fab_{CD3} **S18** to their respective conjugates Sia-BCN **S28** and Fab_{CD3}-BCN **S30** (Figure 3/D, E).

With these encouraging results in hand, the generation of a Fab_{CD20}-Fab_{HER2}-sialidase construct **S37** was attempted as a test reaction (and future negative control) for a Fab_{CD3}-Fab_{HER2}-sialidase construct *via* a modified protocol of the trispecific generation detailed before (Figure 3/A). Fab_{HER2}-Tet-N_3 **15** and Fab_{CD20}-BCN **S25** were prepared and reacted with each other at pH 5 overnight to produce Fab_{CD20}-Fab_{HER2}-N_3 bispecific antibody **S31**. This was then reacted with Br_2PD-BCN **3** and after removal of small molecules, with sialidase (pre-reduced with TCEP) at pH 8 overnight. After the reaction was shown to have not gone to completion, a further 1 eq. of Br_2PD-BCN **3** was added and the reaction incubated overnight once more, and then SDS-PAGE analysis was performed and suggested formation of the final construct (Figure 3/I).

After this time, the mixture was purified by SEC. The excess sialidase **6**, left-over Fab and Fab_{CD20}-Fab_{HER2}-N_3 bispecific antibody **S31** separated well from the larger constructs, but unfortunately among the larger species separation was not very good (SDS-PAGE: Figure 3/J, SEC UV trace: Figure 3/K). Nonetheless, LC-MS analysis of the largest fraction showed the expected mass of Fab_{CD20}-Fab_{HER2}-Sia_2 **S36**, although due to a combination of low concentration and the construct not ionising well on the MS, the data was rather noisy (Figure 3/L).

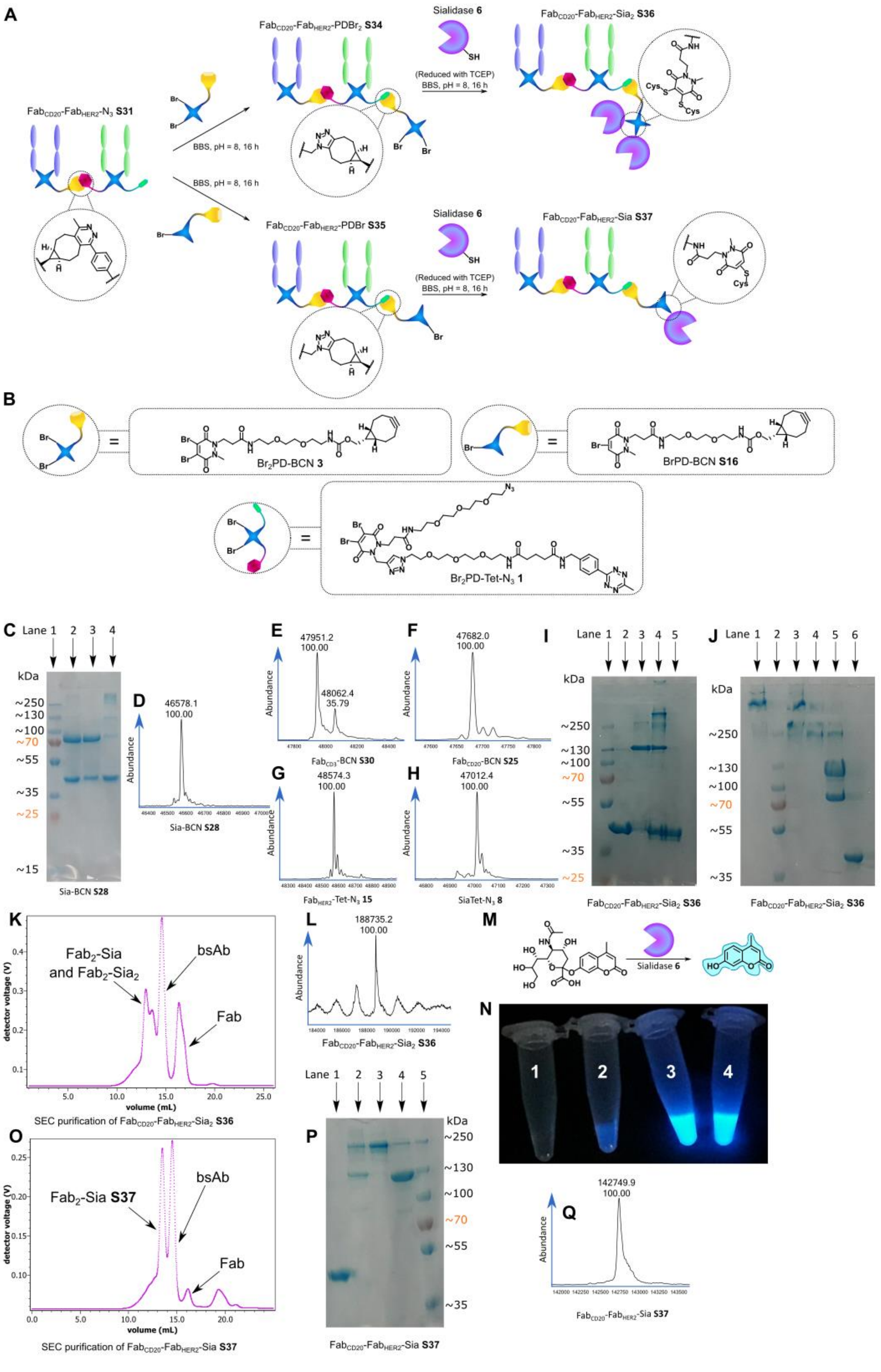
A
Fab$_{CD20}$-Fab$_{HER2}$-N$_3$ S31
BBS, pH = 8, 16 h
Fab$_{CD20}$-Fab$_{HER2}$-PDBr$_2$ S34
Fab$_{CD20}$-Fab$_{HER2}$-PDBr S35
Sialidase 6
(Reduced with TCEP)
BBS, pH = 8, 16 h
Fab$_{CD20}$-Fab$_{HER2}$-Sia$_2$ S36
Fab$_{CD20}$-Fab$_{HER2}$-Sia S37
B
Br$_2$PD-BCN 3
BrPD-BCN S16
Br$_2$PD-Tet-N$_3$ 1
C
Lane 1 2 3 4
kDa
~250 ~130 ~100 ~70 ~55 ~35 ~25 ~15
Sia-BCN S28
D
46578.1
100.00
Abundance
Sia-BCN S28
E
47951.2
100.00
48062.4
35.79
Fab$_{CD3}$-BCN S30
F
47682.0
100.00
Fab$_{CD20}$-BCN S25
G
48574.3
100.00
Fab$_{HER2}$-Tet-N$_3$ 15
H
47012.4
100.00
SiaTet-N$_3$ 8
I
Lane 1 2 3 4 5
kDa
~250 ~130 ~100 ~70 ~55 ~35 ~25
Fab$_{CD20}$-Fab$_{HER2}$-Sia$_2$ S36
J
Lane 1 2 3 4 5 6
kDa
~250 ~130 ~100 ~70 ~55 ~35
Fab$_{CD20}$-Fab$_{HER2}$-Sia$_2$ S36
K
Fab$_2$-Sia and Fab$_2$-Sia$_2$
bsAb
Fab
detector voltage (V)
volume (mL)
SEC purification of Fab$_{CD20}$-Fab$_{HER2}$-Sia$_2$ S36
L
188735.2
100.00
Abundance
Fab$_{CD20}$-Fab$_{HER2}$-Sia$_2$ S36
M
Sialidase 6
N
1 2 3 4
O
Fab$_2$-Sia S37
bsAb
Fab
detector voltage (V)
volume (mL)
SEC purification of Fab$_{CD20}$-Fab$_{HER2}$-Sia S37
P
Lane 1 2 3 4 5
kDa
~250 ~130 ~100 ~70 ~55 ~35
Fab$_{CD20}$-Fab$_{HER2}$-Sia S37
Q
142749.9
100.00
Abundance
Fab$_{CD20}$-Fab$_{HER2}$-Sia S37

Figure 3 | **Initial experiments towards generating a Fab_X-Fab_Y-Sialidase conjugate.**
A | Initial strategies for generating Fab_{CD20}-Fab_{HER2}-$(Sia)_n$ conjugates. A Fab_{CD20}-Fab_{HER2}-N_3 bsAb **S31** was generated as before (see Figure 2/G-O, albeit with the positions of Fab_{CD20} **S22** and Fab_{HER2} **S21** in the construct swapped). This bsAb was then reacted with either Br_2PD-BCN **3** as before (see Figure 2/G-O) or with BrPD-BCN **S16**. The resulting Fab_{CD20}-Fab_{HER2}-$PDBr_2$ **S34** or Fab_{CD20}-Fab_{HER2}-PDBr **S35** was then reacted with reduced *Salmonella typhimurium* Sialidase enzyme cysteine mutant **6** (ST Sia) to generate Fab_{CD20}-Fab_{HER2}-Sia_2 **S36** or Fab_{CD20}-Fab_{HER2}-Sia **S37**, respectively. **B |** The PDs used for the modification of sialidase and the initial attempts at the construction of a Fab_{CD20}-Fab_{HER2}-$(Sia)_n$ conjugates; Br_2PD-BCN **3**, Br_2PD-Tet-N_3 **1** and BrPD-BCN6 **S16**. **C |** SDS-PAGE of reaction of sialidase with Br_2PD-BCN **3**. **Lane 1:** Ladder. **Lane 2:** Native ST sialidase **6**. **Lane 3:** Native sialidase **6** + Br_2PD-BCN **3**. **Lane 4:** Reduced sialidase **6** (*via* TCEP) + Br_2PD-BCN **3**. **D |** LC-MS analysis of Sia-BCN **S28**. Expected mass: 46578 Da. Observed mass: 46578 Da. **E |** LC-MS analysis of Fab_{CD3}-BCN **S30**. Expected mass: 47948 Da. Observed mass: 47951 Da. **F |** LC-MS analysis of Fab_{CD20}-BCN **S25**. Expected mass: 47683 Da. Observed mass: 47682 Da. **G |** LC-MS analysis of Fab_{HER2}-Tet-N_3 **15**. Expected mass: 48574 Da. Observed mass: 48574 Da. **H |** LC-MS analysis of Sia-Tet-N_3 **8**. Expected mass: 47012 Da. Observed mass: 47012 Da. **I |** SDS-PAGE of Fab_{CD20}-Fab_{HER2}-Sia_2 **S36** formation. **Lane 1:** Ladder. **Lane 2:** Fab_{HER2} **S21**. **Lane 3** Fab_{CD20}-Fab_{HER2}-$PDBr_2$ **S34**. **Lane 4:** Fab_{CD20}-Fab_{HER2}-Sia_2 **S36**. **Lane 5:** Reduced sialidase **6**. **J |** SDS-PAGE after SEC purification of Fab_{CD20}-Fab_{HER2}-Sia_2 **S36**. **Lane 1**: Largest fraction + TCEP. **Lane 2:** Ladder. **Lane 3:** Largest fraction, Fab_{CD20}-Fab_{HER2}-Sia_2 **S36**. **Lane 4:** Fab_{CD20}-Fab_{HER2}-Sia. **Lane 5:** Fab_{CD20}-Fab_{HER2}-N_3 **S31** and sialidase dimer. **Lane 6:** Excess sialidase **6**. **K |** SEC UV-trace of Fab_{CD20}-Fab_{HER2}-Sia_2 **S36** formation reaction. **L |** LC-MS analysis of Fab_{CD20}-Fab_{HER2}-Sia_2 **S36**. Expected mass: 188720 Da. Observed mass: 188735 Da. **M |** The 4-methylumbelli-feryl N-acetyl-α-D-neuraminic acid (MUNANA) assay to assess sialidase **6** activity. The enzymatic activity of sialidase **6** cleaves MUNANA and releases fluorescent 4-methylumbelliferone. **N |** The MUNANA assay carried out on Fab_{CD20}-Fab_{HER2}-Sia_2 **S36**. Eppendorf vials were photographed under a UV lamp (365 nm). **Vial 1:** PBS. **Vial 2:** A solution of MUNANA in PBS. **Vial 3:** A solution of MUNANA and ST sialidase **6** in PBS. **Vial 4:** A solution of MUNANA and Fab_{CD20}-Fab_{HER2}-Sia_2 **S36** in PBS. **O |** SEC UV-trace of Fab_{CD20}-Fab_{HER2}-Sia **S37** formation reaction. **P |** SDS-PAGE of SEC purified Fab_{CD20}-Fab_{HER2}-Sia **S37**. **Lane 1:** Fab_{HER2} **S21**. **Lane 2:** Crude Fab_{CD20}-Fab_{HER2}-Sia **S37**. **Lane 3:** Purified Fab_{CD20}-Fab_{HER2}-Sia **S37**. **Lane 4:** Recovered Fab_{CD20}-Fab_{HER2}-PDBr **S35**. **Lane 5:** Ladder. **Q |** LC-MS analysis of Fab_{CD20}-Fab_{HER2}-Sia **S37**. Expected mass: 142725 Da. Observed mass: 142750 Da. **|** Generation of most Fab conjugates have been carried out 2-3 times yielding similar results. Each protein–protein construct was generated a single time unless otherwise stated.

With Fab_{CD20}-Fab_{HER2}-Sia_2 **S36** obtained, the impact of the procedure on the activity of the enzyme was tested with a MUNANA assay. MUNANA contains a 4-methylumbelliferone moiety masked by a glycan that sialidase can cleave off releasing the UV-active chromophore (Figure 3/M). By eye, no difference in fluorescence intensity could be observed when adding MUNANA to Fab_{CD20}-Fab_{HER2}-Sia_2 **S36** or an equivalent concentration of sialidase, whilst the controls, MUNANA in PBS or just PBS alone, showed markedly less or no fluorescence, respectively (Figure 3/N). Of course, this experiment was no substitute for a quantitative assay, but it did suggest that the enzyme retained (at least some of) its biological activity.

As mentioned above, separation between larger species was difficult, and furthermore it was hypothesized that having an additional sialidase on the construct would not improve efficacy but might increase off-target effects. To address these issues a new synthesis was attempted where instead of Br_2PD-BCN **3**, BrPD-BCN **S16** was clicked to bsAb-N_3 to make diaddition of sialidase **6** mechanistically impossible (Figure 3/A). To further ease purification, after generation of bsAb-PDBr, the construct was subjected to protein A purification (to remove excess Fab_{CD20} **S22** which would not be bound), but instead of eluting the construct, subsequent reaction steps were performed with the construct bound to the protein A resin. Thus, after sialidase **6** addition and incubation, unreacted sialidase **6** could just be washed from the column before the final construct was eluted under acidic conditions. This strategy showed a marked improvement in both conversion of the bsAb to the final construct (as only the Fab_{CD20}-Fab_{HER2}-Sia **S37** was produced), and in the ease of SEC purification (Figure 3/O). SDS-PAGE (Figure 3/P) and LC-MS (Figure 3/Q) analysis confirmed the purity of the product.

Unfortunately, it was found that these results were hard to reproduce, due to issues with side-reactions and purification. The postulated side-reactions include an internal lysine on the bsAb-PDBr displacing the bromine atom on the PD and thus competing with reduced sialidase **6** (Sia-SH) addition, as evidenced by the loss of a Br-mass bsAb-PDBr (data not shown), from re-oxidation of Sia-SH **6** to Sia-S-S-Sia, and background reaction between the PD portion of Fab_{CD20}-Fab_{HER2}-PDBr **S35** and TCEP (in case it was added to limit Sia-SH **6** re-oxidation). The low conversion (<50%) probably arising due to these side-reactions limited the efficiency of purification leading to low yields after SEC. To circumvent these issues, a more elegant method was proposed as detailed in the main text along with suitable biological tests of the generated constructs.

8.2. Synthetic chemistry section

8.2.1. General experimental details for synthetic chemistry

Chemicals were purchased from Sigma-Aldrich, Santa Cruz Biotechnology, or AlfaAesar, and were used as received unless otherwise stated. Solvents were used as supplied. Where described below, petrol refers to petroleum ether (b.p. 40-60 °C). All reactions were monitored by thin-layer chromatography (TLC) on pre-coated silica gel plates. Flash column chromatography was carried out with either pre-loaded Biotage® SNAP column chromatography cartridges or pre-loaded GraceResolv™ flash cartridges on a Biotage® Isolera Spektra One flash chromatography system. All reaction mixtures were stirred magnetically unless stated otherwise. Al reactions involving moisture sensitive compounds or procedures were carried out in flame-dried flask under an atmosphere of argon. Room temperature (RT) is defined as 16-23 °C. Reactions at 0 °C were cooled with an ice/water bath. Removal of solvent and concentration *in vacuo* was carried out on a Büchi rotary evaporator followed by evaporation under high vacuum.

^{1}H NMR spectra were obtained at 400, 500, 600 or 700 MHz. ^{13}C NMR spectra were obtained at 100, 125, 150 or 175 MHz. All results were obtained using Bruker NMR instruments, the models are as follows: Avance Neo 700, Avance III 600, Avance 500, Avance III 400. The chemical shifts (δ) for ^{1}H and ^{13}C are quoted relative to residual signals of the solvent on the ppm scale. ^{1}H NMR peaks are reported as singlet (s), doublet (d), triplet (t), quint. (quintet), m (multiplet), br. (broad), dd (doublet of doublet). Coupling constants (J values) are reported in Hertz (Hz) and are H-H coupling constants unless otherwise stated. In the case of amide rotamers, and when possible, only the major rotamer has been assigned for chemical shifts, and areas underneath all rotameric peaks have been considered for integration calculations. Peak assignments were carried out with the aid of ^{1}H COSY and ^{1}H–^{13}C HSQC experiments where necessary. NMR analysis was carried out with MestReNova, version 6. Infrared spectra were obtained on a Perkin Elmer Spectrum 100 FTIR Spectrometer operating in ATR mode. For synthetic products mass spectra were obtained from the UCL mass spectrometry service on a Thermo Orbitrap Exactive Plus (ESI) mass spectrometer.

8.2.2. Compound **S1**, *tert*-butyl (4-cyanobenzyl)carbamate[9]

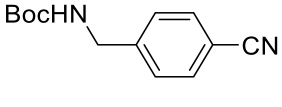

To a stirring solution of NaOH (3.6 g, 89.1 mmol) and di-*tert*-butyl dicarbonate (7.1 g, 32.6 mmol) in H_2O (30 mL) was added, at room temperature, a pre-dissolved solution of 4-(aminomethyl)benzonitrile (5.0 g, 29.7 mmol) in H_2O (30 mL). The mixture was stirred for 16 h, after which time a white precipitate had formed. The mixture was filtered, the solid washed with H_2O (100 mL), and the resulting solid dried under vacuum to yield compound **S1** as a white solid (6.1 g, 26.2 mmol, 88%). ^{1}H NMR (400 MHz, $CDCl_3$) δ 7.62 (d, *J* = 8.3, 2H), 7.38 (d, *J* = 8.3, 2H), 4.96 (br. s, 1H), 4.37 (d, *J* = 5.9 Hz, 2H), 1.46 (s, 9H); ^{13}C NMR (100 MHz,

$CDCl_3$) δ 144.7 (C), 132.5 (C), 127.9 (C), 118.8 (C), 111.1 (C), 80.1 (C), 44.3 (CH_2), 28.4 (CH_3); IR (solid) 3350, 2974, 2927, 2226, 1692 cm^{-1}.

8.2.3. Compound **S2**, *tert*-butyl (4-(6-methyl-1,2,4,5-tetrazin-3-yl)benzyl)carbamate[10]

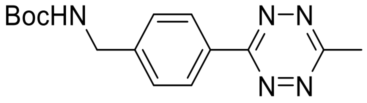

The following procedure was adapted from work by Lang *et al.*[9] To a stirring suspension of *tert*-butyl carbamate **S1** (3.0 g, 12.9 mmol), acetonitrile (6.72 mL, 12.9 mmol), and $Zn(OTf)_2$ (2.34 g, 6.46 mmol) in 1,4-dioxane (6 mL) was added, at room temperature, hydrazine hydrate (80% w/w, 39.5 mL, 646 mmol). The reaction was heated to 65 °C and stirred for 72 h. After this time, the reaction was cooled to room temperature and diluted with EtOAc (50 mL). The mixture was washed with 1 M HCl (50 mL), and the aqueous phase extracted with EtOAc (2 × 30 mL). The organic phase was dried ($MgSO_4$), filtered and solvent was then removed *in vacuo*. The resulting crude residue was dissolved in a mixture of DCM and acetic acid (1:1, 200 mL), and to this was added $NaNO_2$ (17.8 g, 258 mmol) slowly over a period of 15 min, during which time the reaction turned bright red. The reaction was then diluted with DCM (200 mL), washed with sodium bicarbonate (sat. aq., 200 mL) and the aqueous phase extracted with DCM (2 × 100 mL). The organic phases were combined and then dried ($MgSO_4$), filtered and the solvent was then removed *in vacuo*. The resulting residue was purified by flash column chromatography (20% EtOAc/petrol) to yield tetrazine **S2** as a pink solid (1.07 g, 3.55 mmol, 28%). ^1H NMR (400 MHz, $CDCl_3$) δ 8.55 (d, *J* = 8.4 Hz, 2H), 7.50 (d, *J* = 8.3 Hz, 2H), 4.97 (br. s, 1H), 4.44 (d, *J* = 5.8 Hz, 2H), 3.09 (s, 3H), 1.48 (s, 9H); ^{13}C NMR (100 MHz, $CDCl_3$) δ 167.3 (C), 164.0 (C), 144.0 (C), 130.1 (C), 128.3 (C), 128.1 (C), 80.1 (C), 28.5 (CH_2), 21.1 (CH_3); IR (solid) 3339, 2974, 2928, 1696, 1516 cm^{-1}.

8.2.4. Compound **S3**, 5-((4-(6-methyl-1,2,4,5-tetrazin-3-yl)benzyl)amino)-5-oxopentanoic acid[11]

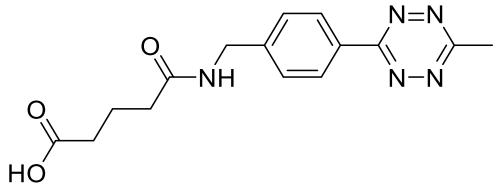

Tert-butyl (4-(6-methyl-1,2,4,5-tetrazin-3-yl)benzyl)carbamate **S2** (800 mg, 2.65 mmol) was dissolved in a mixture of TFA and DCM (1:4, 20 mL) and the solution was stirred at room temperature for 2 h. The solvent was then removed *in vacuo* and the mixture re-dissolved in THF (50 mL). To this solution was added glutaric anhydride (605 mg, 5.31 mmol) and the mixture stirred at 55 °C for 16 h. The solvent was removed *in vacuo* and the mixture re-dissolved in sat. aq. K_2CO_3 solution (100 mL). The mixture was then acidified with 15% HCl aq. solution until the mixture stopped producing $CO_{2(g)}$ on acid. The mixture was then extracted with EtOAc (3 × 50 mL), and the combined organic phases washed with H_2O (4 × 30 mL) and

brine (30 mL), and then dried ($MgSO_4$). Any precipitate formed during extraction was re-dissolved in sat. aq. K_2CO_3 solution (30 mL) and the work-up was repeated on this solution and the dried organic phases were combined, filtered and the solvent removed *in vacuo* to yield compound **S3** as a purple powder (691 mg, 2.2 mmol, 83%) without further purification. ^{1}H NMR (400 MHz, $CDCl_3$) δ 8.41 (d, *J* = 8.4, 2H), 7.51 (d, *J* = 8.5 Hz, 2H), 4.38 (d, *J* = 6.0 Hz, 2H), 4.27 (q, *J* = 7.4 Hz, 4H), 2.98 (s, 3H), 1.76 (quint., *J* = 7.4 Hz, 2 H); ^{13}C NMR (100 MHz, $CDCl_3$) δ 174.2 (C), 171.9 (C), 167.1 (C), 163.2 (C), 144.5 (C), 130.4 (C), 128.0 (C), 127.5 (C), 41.9 (CH_2), 34.4 (CH_2), 33.0 (CH_2), 20.8 (CH_3), 20.7 (CH_2); IR (thin film) 3271, 3025, 2973, 2923, 2880, 1694, 1630, 1523 cm^{-1}.

8.2.1. Compound **S4**, *N*^{1}-(3-(2-(2-(3-aminopropoxy)ethoxy)ethoxy)propyl)-*N*^{5}-(4-(6-methyl-1,2,4,5-tetrazin-3-yl)benzyl)glutaramide[12]

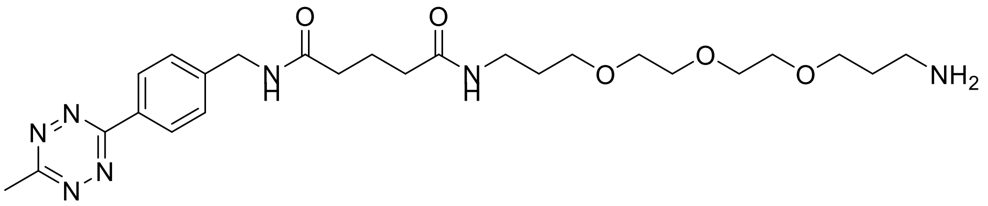

To a solution of *O,O'*-bis(3-aminopropyl)diethylene glycol (280 mg, 1.27 mmol) in DCM (5 mL) was added slowly, at room temperature, a solution of 5-((4-(6-methyl-1,2,4,5-tetrazin-3-yl)benzyl)amino)-5-oxopentanoic acid **S3** (200 mg, 0.63 mmol), HATU (240 mg, 0.63 mmol), and NEt_3 (64 mg, 0.63 mmol) in DCM (5 mL). The resulting solution was stirred at room temperature for 16 h. The solvent was then removed *in vacuo*, and the mixture re-dissolved in 1 M HCl solution (20 mL) and washed with DCM (3 × 20 mL) to remove unreacted 5-((4-(6-methyl-1,2,4,5-tetrazin-3-yl)benzyl)amino)-5-oxopentanoic acid **S3**. The aqueous phase was then basified with sat. aq. K_2CO_3 solution until $CO_{2(g)}$ evolution stopped, and then extracted with DCM (3 × 20 mL). The combined organic phases were extracted with 1 M HCl solution (20 mL). The aqueous phase was basified with sat. aq. K_2CO_3 solution until $CO_{2(g)}$ evolution stopped, and then extracted with DCM (3 × 20 mL). The combined organic phases were washed with brine (20 mL), dried ($MgSO_4$), filtered and the solvent removed *in vacuo*. The crude residue was purified by flash column chromatography (10–30% MeOH in DCM) to afford compound **S4** (47.6 mg, 0.09 mmol, 15%) as a purple oil. ^{1}H NMR (600 MHz, $CDCl_3$) δ 8.49 (d, *J* = 8.4 Hz, 2H), 8.35 (t, *J* = 5.8 Hz, 1H), 7.57 (d, *J* = 8.5 Hz, 2H), 6.67 (t, *J* = 6.1 Hz, 1H), 4.52 (d, *J* = 6.2 Hz, 2H), 3.71 (t, *J* = 5.3 Hz, 2H), 3.65–3.61 (m, 4H), 3.61–3.57 (m, 4H), 3.50 (t, *J* = 5.6 Hz, 2H), 3.32 (q, *J* = 6.8 Hz, 2H), 3.19–3.15 (m, 2H), 3.08 (s, 3H), 2.40 (t, *J* = 7.5 Hz, 2H), 2.30 (t, *J* = 6.9 Hz, 2H), 2.03–1.96 (m, 4H), 1.77 (quint., *J* = 6 Hz, 2H); ^{13}C NMR (150 MHz, $CDCl_3$) δ 174.0 (C), 173.3 (C), 167.2 (C), 164.2 (C), 144.6 (C), 130.4 (C), 128.8 (CH), 128.1 (CH), 70.9 (CH_2), 70.7 (CH_2), 69.8 (CH_2), 69.7 (CH_2), 69.6 (CH_2), 68.3 (CH_2), 43.0 (CH_2), 40.7 (CH_2), 36.5 (CH_2), 35.4 (CH_2), 35.1 (CH_2), 30.0 (CH_2), 26.1 (CH_2), 21.9 (CH_2), 21.3 (CH_3). IR (thin film) 3302, 2945, 2831, 1642, 1630, 1542 cm^{-1}.

8.2.2. Compound **S5**, *N*[1]-(2-(2-(2-(2-azidoethoxy)ethoxy)ethoxy)ethyl)-*N*[5]-(4-(6-methyl-1,2,4,5-tetrazin-3-yl)benzyl)glutaramide[3]

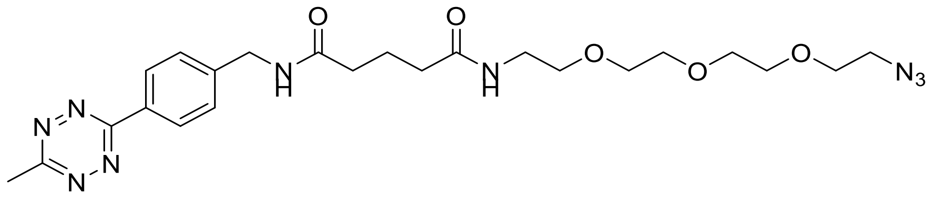

To a solution of 5-((4-(6-methyl-1,2,4,5-tetrazin-3-yl)benzyl)amino)-5-oxopentanoic acid **S3** (200 mg, 0.63 mmol) in DCM (5 mL) was added, at room temperature, HATU (240 mg, 0.63 mmol), and NEt_3 (87.8 µL, 0.63 mmol), and the reaction stirred for 5 min. Subsequently, to this solution was added, at room temperature, a solution of 2-(2-(2-(2-azidoethoxy)ethoxy)ethoxy)ethan-1-amine (387.5 µL, 1.96 mmol) in DCM (5 mL), and the resulting solution was stirred at room temperature for 16 h. The reaction was then diluted with EtOAc (25 mL) and H_2O (25 mL) and the phases separated. The aqueous phase was extracted with EtOAc (3 × 25 mL) and the combined organic phases were washed with H_2O (3 × 25 mL), brine (20 mL), dried ($MgSO_4$), filtered and the solvent removed *in vacuo*. The crude residue was purified by flash column chromatography (0-10% MeOH in DCM) to afford *N*[1]-(2-(2-(2-(2-azidoethoxy)ethoxy)ethoxy)ethyl)-*N*[5]-(4-(6-methyl-1,2,4,5-tetrazin-3-yl)benzyl)glutaramide **S5** (209.5 mg, 0.51 mmol, 64%) as a purple solid. ^{1}H NMR (400 MHz, $CDCl_3$) δ 8.55 (d, *J* = 8.4 Hz, 2H), 7.51 (d, *J* = 8.5 Hz, 2H), 6.61 (br. s, 1H), 6.16 (br. s, 1H), 4.55 (d, *J* = 6.0 Hz, 2H), 3.68–3.59 (m, 10 H), 3.56–3.52 (m, 2H), 3.45–3.36 (m, 4H), 3.09 (s, 3H), 2.35 (t, *J* = 7.1 Hz, 2H), 2.26 (t, *J* = 6.9 Hz, 2H), 2.01 (quint., *J* = 6.9 Hz, 2 H); ^{13}C NMR (100 MHz, $CDCl_3$) δ 172.7 × 2 (C), 167.3 (C), 164.0 (C), 143.6 (C), 131.0 (C), 128.6 (CH), 128.3 (CH), 70.8 (CH_2), 70.7 (CH_2), 70.6 (CH_2), 70.3 (CH_2), 70.1(CH_2), 69.8 (CH_2), 50.7 (CH_2), 43.3 (CH_2), 39.3 (CH_2), 35.3 (CH_2), 35.2 (CH_2), 22.0 (CH_2), 21.2 (CH_3). IR (solid) 3298, 3076, 2868, 2101, 1637, 1541 cm^{-1}. HRMS (ESI) calcd for $C_{23}H_{34}N_9O_5$ $[M+H]^+$ 516.2677; observed 516.2677.

8.2.3. Compound **S6**, di-*tert*-butyl 1-methylhydrazine-1,2-dicarboxylate[2]

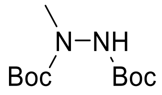

To a solution of methyl hydrazine (1.14 mL, 21.7 mmol) in propan-2-ol (16 mL), was added drop-wise over 30 min a solution of di-*tert*-butyl dicarbonate (11.4 g, 52.1 mmol, pre-dissolved in DCM (12 mL)). The reaction was then stirred at 21 °C for 16 h. After this time, the solvents were removed *in vacuo* and the crude residue purified by flash column chromatography (0% to 15% EtOAc/petrol) to afford di-*tert*-butyl-1-methylhydrazine-1,2-dicarboxylate **S6** (4.67 g, 19.1 mmol, 88%) as a white solid. ^{1}H NMR (400 MHz, $CDCl_3$, rotamers) δ 6.41–6.15 (m, 1H) 3.10 (s, 3H), 1.46–1.45 (m, 18H); ^{13}C NMR (150 MHz, $CDCl_3$, rotamers) δ 155.9 (C), 81.3 (C), 37.6 (CH_3), 28.3 (CH_3); IR (solid) 3299, 2974, 2929, 1703 cm^{-1}.

8.2.4. Compound **S7**, di-*tert*-butyl 1-(3-(*tert*-butoxy)-3-oxopropyl)-2-methylhydrazine-1,2-dicarboxylate[2]

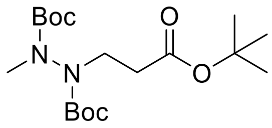

To a solution of di-*tert*-butyl 1-methylhydrazine-1,2-dicarboxylate **S6** (3.00 g, 12.2 mmol) in *tert*-butanol (15 mL) was added 10% aq. NaOH (0.5 mL) and the reaction mixture stirred at 21 °C for 10 min. After this time, *tert*-butyl acrylate (5.31 mL, 36.6 mmol) was added to the solution and the reaction mixture was heated at 60 °C for 24 h. Following this, the solvent was removed *in vacuo* and the crude residue was dissolved in EtOAc (150 mL) and washed with water (3 × 50 mL). The organic layer was then dried ($MgSO_4$), filtered and the solvent removed *in vacuo*. Purification of the crude residue by flash column chromatography (0% to 20% EtOAc/petrol) afforded di-*tert*-butyl-1-(3-(*tert*-butoxy)-3-oxopropyl)-2-methylhydrazine-1,2-dicarboxylate **S7** (3.33 g, 8.91 mmol, 73%) as a clear oil. ^{1}H NMR (600 MHz, $CDCl_3$, rotamers) δ 3.82–3.47 (m, 2H), 3.03–2.94 (m, 3H), 2.47 (t, *J* = 7.1 Hz, 2H), 1.48–1.37 (m, 27H); ^{13}C NMR (150 MHz, $CDCl_3$, rotamers) δ 171.0 (C), 155.4 (C), 154.4 (C), 81.0 (C), 44.6 (CH_3), 36.6 (CH_2), 34.1 (CH_2), 28.3 (CH_3); IR (thin film) 2974, 2931, 1709 cm^{-1}.

8.2.5. Compound **S8**, 3-(4,5-dibromo-2-methyl-3,6-dioxo-3,6-dihydropyridazin-1(2*H*)-yl)propanoic acid[2]

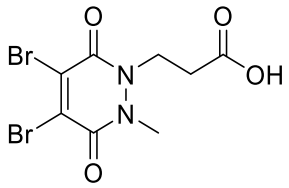

Dibromomaleic anhydride (4.00 g; 14.61 mmol) was dissolved in AcOH (80 mL) and heated under reflux for 30 min. To this solution was added di-*tert*-butyl 1-(3-(*tert*-butoxy)-3-oxopropyl)-2-methylhydrazine-1,2-dicarboxylate **S7** (4.77 g; 12.75 mmol) and the reaction heated under reflux for a further 4 h. The solvent was removed *in vacuo* by co-evaporation with toluene and the crude residue purified by flash column chromatography (50-100% EtOAc 1% AcOH/petrol) to yield 3-(4,5-dibromo-2-methyl-3,6-dioxo-3,6-dihydropyridazin-1(2*H*)-yl)propanoic acid **S8** (3.22 g, 9.0 mmol, 71%) as a yellow powder. ^{1}H NMR (700 MHz, $CDCl_3$) δ 4.41 (t, *J* = 7.1 Hz, 2H), 3.69 (s, 3H), 2.77 (t, *J* = 7.1 Hz, 2H); ^{13}C NMR (150 MHz, $CDCl_3$) δ 173.9 (C), 153.5 (C), 153.2 (C), 136.4 (C), 135.7 (C), 43.5 (C), 35.3 (C), 31.2 (C); IR (thin film) 3300-2700, 2924, 1726, 1617, 1570, 1442, 1396 cm^{-1}.

8.2.6. Compound **S9**, 2,5-dioxopyrrolidin-1-yl 3-(4,5-dibromo-2-methyl-3,6-dioxo-3,6-dihydropyridazin-1(2*H*)-yl)propanoate[2]

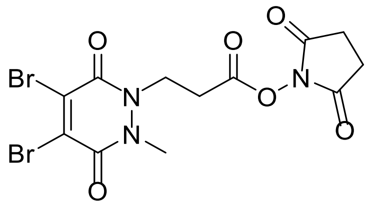

To a solution of 3-(4,5-dibromo-2-methyl-3,6-dioxo-3,6-dihydropyridazin-1(2*H*)-yl)propanoic acid **S8** (2.00 g; 5.62 mmol) in dry THF (40 mL) at 0 °C was added DCC (1.28 g; 6.2 mmol). The solution was stirred at 0 °C for 30 min. Following this, was added NHS (718 mg, 6.2 mmol) and the reaction stirred at room temperature for a further 16 h. The solvent was removed *in vacuo* and the crude residue purified by flash column chromatography (20-100% EtOAc/petrol) to yield pyridazinedione **S9** (796 mg, 1.76 mmol, 31%) as a white powder. ^{1}H NMR (700 MHZ, $CDCl_3$) δ 4.48 (t, J= 6.9, 2H), 3.68 (s, 3H), 3.10 (t, *J* = 6.9, 2H), 2.85 (br. s, 4H); ^{13}C NMR (175 MHz, $CDCl_3$) δ 168.7 (C), 166.0 (C), 153.3 (C), 153.1 (C), 136.9 (C), 135.5 (C), 43.5 (CH_2), 35.3 (CH_3), 30.5 (CH_2), 25.7 (CH_2); IR (thin film) 2927, 2851, 1733, 1632, 1571, 1203 cm^{-1}.

8.2.7. Compound **3**, ((1R,8S,9s)-bicyclo[6.1.0]non-4-yn-9-yl)methyl (2-(2-(2-(3-(4,5-dibromo-2-methyl-3,6-dioxo-3,6-dihydropyridazin-1*(2H)-yl*)propanamido)ethoxy)ethoxy)ethyl)carbamate[2]

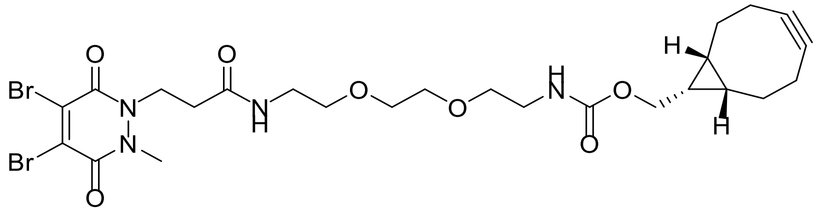

To a solution of 2,5-dioxopyrrolidin-1-yl 3-(4,5-dibromo-2-methyl-3,6-dioxo-3,6- dihydropyridazin-1(2*H*)-yl) propanoate **S9** (100.0 mg, 0.221 mmol, pre-dissolved in MeCN (10 mL)), was added ((1R,8S,9s)-bicyclo[6.1.0]non-4-yn-9-yl)methyl (2-(2-(2-aminoethoxy)ethoxy)ethyl)carbamate (160.9 mg, 0.243 mmol) and the reaction mixture was stirred at 21 °C for 16 h. After this time, the reaction was concentrated *in vacuo* and the crude residue dissolved in $CHCl_3$ (50 mL) and washed with water (2 × 30 mL) and saturated aq. K_2CO_3 (30 mL). The organic layer was then dried ($MgSO_4$) and concentrated *in vacuo.* Purification of the crude residue by flash column chromatography (0% to 10% MeOH/EtOAc) afforded ((1*R*,8*S*,9*S*)-Bicyclo[6.1.0] non-4-yn-9-yl)methyl (2-(2-(2-(3-(4,5-dibromo-2-methyl-3,6-dioxo-3,6-dihydropyridazin- 1(2*H*)-yl)propanamido)ethoxy)ethoxy)ethyl) carbamate **3** (105.0 mg, 0.168 mmol, 72%) as a yellow oil: ^{1}H NMR (600 MHz, $CDCl_3$, rotamers) δ 7.84 (s, 0.3H), 6.38 (s, 0.7H), 5.78 (s, 0.3H), 5.24 (s, 0.7H), 4.44 (t, *J* = 6.6 Hz, 2H), 4.14–4.12 (m, 2H), 3.73–3.71 (m, 3H), 3.60–3.57 (m, 6H), 3.53–3.52 (m, 2H), 3.45–3.43 (m, 2H), 3.39–3.35 (m, 2H), 2.62 (t, *J* = 6.6 Hz, 2H), 2.29–2.20 (m, 6H), 1.61–1.57 (m, 2H), 1.35–1.32 (m, 1H), 0.96–0.94 (m, 2H); ^{13}C NMR (150 MHz, CDCl3, rotamers) δ 169.1 (C), 156.9 (C), 153.1 (C), 153.0 (C), 136.4 (C), 135.5 (C), 98.9 (C), 70.4 (CH_2), 70.3 (CH_2), 69.7 (CH_2), 63.0 (CH_2), 44.6 (CH_2), 40.8 (CH_2), 39.5 (CH_2), 35.1 (CH_3), 34.1 (CH_2), 29.3 (CH_2), 29.2 (CH_2), 21.6 (CH_2), 20.2 (CH_2), 17.9 (CH), 14.3 (CH); IR (thin film) 3329, 2920, 2858, 1708, 1630, 1572, 1534 cm^{-1}; LRMS (ESI), 687 (50, $[M^{81}Br^{81}Br+Na]^+$) 685 (100, $[M^{79}Br^{81}Br+Na]^+$), 683 (50, $[M^{79}Br^{79}Br+Na]^+$), 663 (60, $[M^{79}Br^{81}Br+H]^+$); HRMS (ESI) calcd for $C_{25}H_{35}Br_2N_4O_7$ $[M^{79}Br^{81}Br+H]^+$ 663.0847; observed 663.0846.

N.B. This batch of molecule **3** was made previously in our lab, but we have chosen to include the experimental details here for ease of reference. Please see cited paper for spectral data.[2]

8.2.1. Compound **4**, *N*[1]-(17-(4,5-dibromo-2-methyl-3,6-dioxo-3,6-dihydropyridazin-1*(2H)-yl*)-15-oxo-4,7,10-trioxa-14-azaheptadecyl)-*N*[5]-(4-(6-methyl-1,2,4,5-tetrazin-3-yl)benzyl)glutaramide[12]

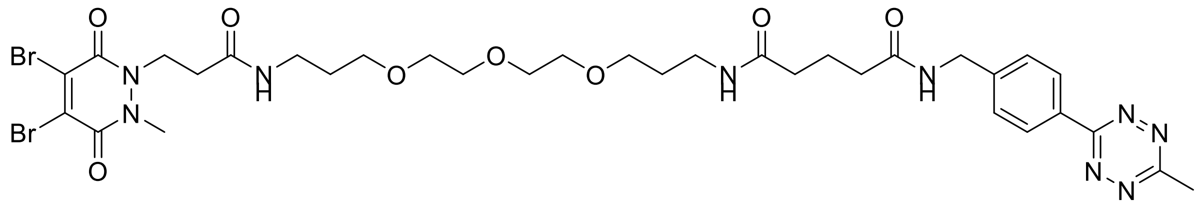

To a solution of 2,5-dioxopyrrolidin-1-yl 3-(4,5-dibromo-2-methyl-3,6-dioxo-3,6-dihydropyridazin-1(2*H*)-yl) propanoate **S9** (61.2 mg, 140.0 µmol) in DCM (5 mL), was added *N*[1]-(3-(2-(2-(3-aminopropoxy)ethoxy)ethoxy)propyl)-*N*[5]-(4-(6-methyl-1,2,4,5-tetrazin-3-yl)benzyl)glutaramide **S4** (27.6 mg, 58.0 µmol) and NEt_3 (7.4 µL, 5.4 mg, 53.0 µmol), and the reaction mixture was stirred at room temperature for 3 h. After this time, the reaction was concentrated *in vacuo* and the crude residue dissolved in $CHCl_3$ (25 mL) and washed with water (2 × 15 mL) and sat. aq. K_2CO_3 (15 mL). The organic layer was then dried ($MgSO_4$), filtered and the solvent removed *in vacuo* . Purification of the crude residue by flash column chromatography (5% to 20% MeOH/EtOAc) afforded compound **4** (15.8 mg, 18.0 µmol, 32%) as a purple oil. ^{1}H NMR (600 MHz, $CDCl_3$) δ 8.53 (d, *J* = 8.4 Hz, 2H), 7.50 (d, *J* = 8.5 Hz, 2H), 7.04 (t, *J* = 5.2 Hz, 1H), 6.80 (t, *J* = 5.8 Hz, 1H), 6.46 (t, *J* = 5.3 Hz, 1H), 4.55 (d, *J* = 6.0 Hz, 2H), 4.39 (t, *J* = 6.9 Hz, 2H), 3.69 (s, 3H), 3.63–3.50 (m, 12H), 3.36–3.26 (m, 4H), 3.09 (s, 3H), 2.80 (d, *J* = 4.8 Hz, 1H), 2.56 (t, *J* = 6.9 Hz, 2H), 2.36 (t, *J* = 7.3 Hz, 2H), 2.26 (t, *J* = 6.9 Hz, 2H), 1.99 (app. quint., *J* = 7.1 Hz, 2H), 1.78–1.69 (m, 4H); ^{13}C NMR (150 MHz, $CDCl_3$) δ 173.0 (C), 172.8 (C), 169.2 (C), 167.4 (C), 164.0 (C), 153.1 (C), 152.9 (C), 143.6 (C), 136.4 (C), 135.4 (C), 131.0 (C), 128.6 (2×CH), 128.3 (2×CH), 70.6 (2×CH_2), 70.1 (2×CH_2), 70.0 (2×CH_2), 44.6 (CH_2), 43.3 (CH_2), 38.2 (CH_2), 38.0 (CH_2), 35.6 (CH_2), 35.4 (CH_2), 35.2 (CH_3), 34.1 (CH_2), 29.1 (CH_2), 28.9 (CH_2), 22.2 (CH_2), 21.3 (CH_3); IR (thin film) 3310, 2923, 2851, 1734, 1631, 1543 cm^{-1}; LRMS (ESI). 858 (50, $[M^{81}Br^{81}Br+H]^+$), 856 (100, $[M^{79}Br^{81}Br+H]^+$), 854 (50, $[M^{79}Br^{79}Br+H]^+$); HRMS (ESI) calcd for $C_{33}H_{46}Br_2N_9O_8Na$ $[M^{79}Br^{81}Br+Na]^+$ 878.1630; observed 878.1639.

8.2.2. Compound **S10**, di-*tert*-butyl 1-(prop-2-yn-1-yl)hydrazine-1,2-dicarboxylate[13]

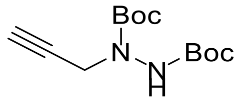

To a solution of di-*tert*-butyl hydrazine-1,2-dicarboxylate (3.00 g, 12.9 mmol) in a mixture of toluene (15 mL) and 5% aq. NaOH (15 mL) were added tetra-*n*-butylammonium bromide (104 mg, 0.32 mmol) and propargyl bromide (5.76 g, 38.7 mmol). The reaction mixture was stirred at 20 °C for 16 h. After this time, H_2O (20 mL) was added, and the mixture was extracted with EtOAc (3 × 30 mL). The combined organic layers were washed with brine (30 mL), dried ($MgSO_4$), filtered and the solvent removed *in vacuo*. Purification by flash column chromatography (20 % EtOAc/petrol) yielded di-*tert*-butyl 1-(prop-2-yn-1-yl)hydrazine-1,2-dicarboxylate **S10** (2.04 g, 7.56 mmol, 59%) as a white solid. ^{1}H NMR (400 MHz, CDCl3,

rotamers) δ 6.48 (br. s, 0.7H), 6.17 (br. s, 0.2H), 4.27 (s, 2H), 2.24 (t, J = 2.4 Hz, 1H), 1.47 (s, 18H); ^{13}C NMR (125 MHz, $CDCl_3$, rotamers) δ 154.7 (C), 82.1 (C), 81.6 (C), 78.8 (C), 72.1 (CH), 39.3 (CH_2), 28.3 (CH_3), 28.3 (CH_3); IR (solid) 3310, 3290, 2982, 1729, 1688, 1512 cm^{-1}.

8.2.3. Compound **S11**, di-*tert*-butyl 1-(3-(*tert*-butoxy)-3-oxopropyl)-2-(prop-2-yn-1-yl)hydrazine-1,2-dicarboxylate[12]

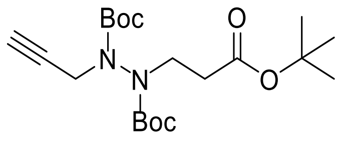

To a solution of di-*tert*-butyl 1-(prop-2-yn-1-yl)hydrazine-1,2-dicarboxylate **S10** (1.5 g, 5.55 mmol) in *tert*-BuOH (10 mL) and 5% NaOH solution (0.5 mL) was added *tert*-butyl acrylate (3.22 mL, 22.2 mmol) and the reaction mixture was heated at 60 °C for 24 h. Following this, the solvent was removed *in vacuo* and the crude residue was dissolved in EtOAc (75 mL) and washed with H_2O (3 × 25 mL). The organic layer was then dried ($MgSO_4$), filtered and the solvent removed *in vacuo* to afford di-*tert*-butyl 1-(3-(*tert*-butoxy)-3-oxopropyl)-2-(prop-2-yn-1-yl)hydrazine-1,2-dicarboxylate **S11** (1.84 g, 4.6 mmol, 83%) as a clear oil. ^{1}H NMR (400 MHz, $CDCl_3$, rotamers) δ 4.69–3.58 (m, 2H), 3.85–3.65 (m, 2H), 2.69–2.60 (m, 2H), 2.27 (t, J = 2.5 Hz, 1H), 1.60–1.38 (m, 27H); ^{13}C NMR (100 MHz, $CDCl_3$, rotamers) δ 171.0 (C), 154.5 (C), 154.3 (C), 82.0 (C), 81.4 (C), 80.7 (C), 78.4 (C), 73.0 (CH), 46.1 (CH_2), 39.4 (CH_2), 34.2 (CH_2), 28.3 (CH_3), 28.2 (CH_3); IR (thin film) 3262, 2978, 1709 cm^{-1}.

8.2.4. Compound **S12**, 3-(4,5-dibromo-3,6-dioxo-2-(prop-2-yn-1-yl)-3,6-dihydropyridazin-1(2*H*)-yl)propanoic acid[12]

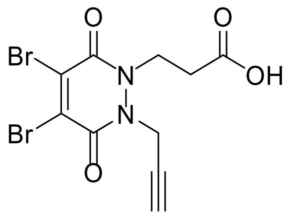

Dibromomaleic anhydride (1.13 g, 4.14 mmol) was dissolved in AcOH (30 mL) and heated under reflux for 30 min. To this solution was added di-*tert*-butyl 1-(3-(*tert*-butoxy)-3-oxopropyl)-2-(prop-2-yn-1-yl)hydrazine-1,2-dicarboxylate **S11** (1.5 g, 3.76 mmol) and the reaction heated under reflux for a further 4 h. The solvent was removed *in vacuo* by co-evaporation with toluene and the crude residue was purified by flash column chromatography (25-100% (99% EtOAc, 1% AcOH)/petrol) to yield 3-(4,5-dibromo-3,6-dioxo-2-(prop-2-yn-1-yl)-3,6-dihydropyridazin-1(2*H*)-yl)propanoic acid **S12** (926 mg, 2.44 mmol, 65%) as a yellow powder. ^{1}H NMR (400 MHz, DMSO-d_6) δ 12.48 (br. s, 1 H), 4.91 (d, J = 2.4 Hz, 2H), 4.27 (t, J = 7.4 Hz, 2H), 3.53 (t, J = 2.4 Hz, 1H), 2.66 (t, J = 7.4 Hz, 2H); ^{13}C NMR (100 MHz, DMSO-d_6) δ 171.9 (C), 153.4 (C),

153.0 (C), 136.2 (C), 135.1 (C), 77.2 (C), 76.7 (CH), 43.4 (CH_2), 37.6 (CH_2), 31.5 (CH_2); IR (solid) 3216, 2979, 2121 (small), 1725, 1660, 1620, 1577 cm^{-1}.

8.2.5. Compound **S13**, 3-(4,5-dibromo-2-((1-(1-(4-(6-methyl-1,2,4,5-tetrazin-3-yl)phenyl)-3,7-dioxo-11,14,17-trioxa-2,8-diazanonadecan-19-yl)-1*H*-1,2,3-triazol-4-yl)methyl)-3,6-dioxo-3,6-dihydropyridazin-1(2*H*)-yl)propanoic acid[3]

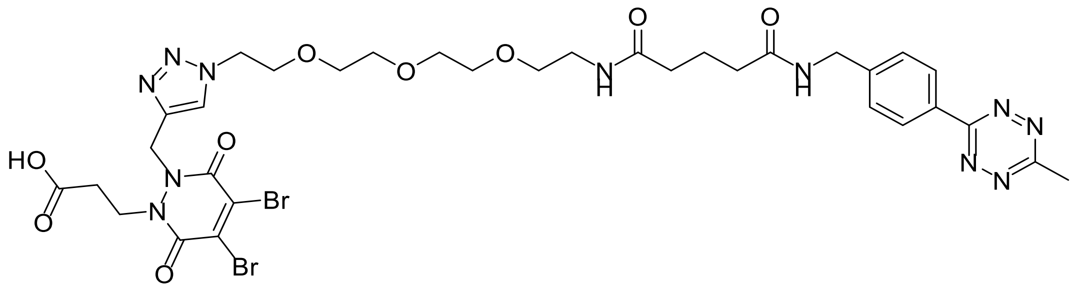

To a solution of *N*[1]-(2-(2-(2-(2-azidoethoxy)ethoxy)ethoxy)ethyl)-*N*[5]-(4-(6-methyl-1,2,4,5-tetrazin-3-yl)benzyl)glutaramide **S5** (50.0 mg, 97.0 µmol) and 3-(4,5-dibromo-3,6-dioxo-2-(prop-2-yn-1-yl)-3,6-dihydropyridazin-1(2*H*)-yl)propanoic acid **S12** (44.3 mg, 116.6 µmol) in THF (10 mL) at 21 °C was added DIPEA (16.8 µL, 97.0 µmol) and CuI (9.4 mg, 48.5 µmol) and the mixture stirred at 21 °C for 5 h. The mixture was then filtered, and the solvent removed *in vacuo*. The mixture was dissolved in H_2O (10 mL) and basified with sat. aq. $NaHCO_3$ (10 mL), and then washed with DCM (3 × 10 mL). The organic phase was discarded, and DCM (10 mL) was added to the aqueous phase. The aqueous phase was then acidified with 4 M HCl until the evolution of $CO_{2(g)}$ stopped and the purple product mostly moved to the organic phase. The aqueous phase was then extracted with further DCM (3 × 10 mL). The combined organic phases were washed with brine, dried ($MgSO_4$), filtered and the solvent removed *in vacuo*. The crude residue was purified by flash column chromatography (0-20% (99% MeOH, 1% AcOH)/DCM) to yield 3-(4,5-dibromo-2-((1-(1-(4-(6-methyl-1,2,4,5-tetrazin-3-yl)phenyl)-3,7-dioxo-11,14,17-trioxa-2,8-diazanonadecan-19-yl)-1*H*-1,2,3-triazol-4-yl)methyl)-3,6-dioxo-3,6-dihydropyridazin-1(2*H*)-yl)propanoic acid **S13** (67.2 mg, 75.0 µmol, 77%) as a purple solid: ^{1}H NMR (400 MHz, DMSO-d_6) δ 8.47 (s, 1H) 8.42 (d, *J* = 8.3 Hz, 2H), 8.10 (s, 1H), 7.88 (s, 1H), 7.51 (d, *J* = 8.3 Hz, 2H), 5.35 (br. s, 2H), 4.50 (t, *J* = 5.1 Hz, 2H), 4.38 (d, *J* = 6.0 Hz, 2H), 4.30 (s 2H), 3.78 (t, *J* = 5.3 Hz, 2H), 3.51–3.43 (m, 10 H), 3.39 (t, *J* = 6.0 Hz, 2H), 3.18 (q, *J* = 5.9 Hz, 2H), 2.99 (s, 3H), 2.18 (t, *J* = 7.5 Hz, 2H), 2.10 (t, *J* = 7.4 Hz, 2H), 1.76 (quint., *J* = 7.7 Hz, 2H); ^{13}C NMR (125 MHz, DMSO-d_6) δ 172.0 (C), 171.8 (C), 167.1 (C), 163.2 (C), 153.5 (C), 152.9 (C), 144.6 (C), 135.9 (C), 135.2 (C), 130.4 (C), 128.1 (CH), 127.5 (CH), 124.5 (CH), 69.7 (CH_2), 69.6 × 2 (CH_2), 69.2 (CH_2), 68.6 (CH_2), 54.9 (CH_2), 49.6 (CH_2), 41.8 (CH_2), 34.8 (CH_2), 34.7 (CH_2), 21.5 (CH_2), 20.9 (CH_3). IR (solid) 3335, 2924, 1721, 1630, 1545 cm^{-1}. HRMS (ESI) calcd for $C_{33}H_{42}Br_2N_{11}O_9$ $[M^{79}Br^{81}Br+H]^+$ 896.1435; observed 896.1503.

8.2.6. Compound **1,** N^1-(2-(2-(2-(2-(4-((2-(1-azido-13-oxo-3,6,9-trioxa-12-azapentadecan-15-yl)-4,5-dibromo-3,6-dioxo-3,6-dihydropyridazin-1(2*H*)-yl)methyl)-1H-1,2,3-triazol-1-yl)ethoxy)ethoxy)ethoxy)ethyl)-N^5-(4-(6-methyl-1,2,4,5-tetrazin-3-yl)benzyl)glutaramide[3]

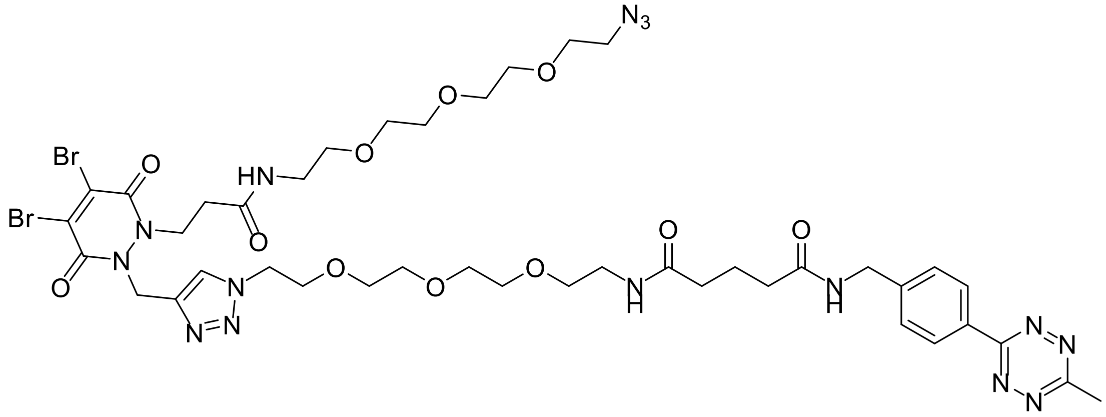

To a solution of 3-(4,5-dibromo-2-((1-(1-(4-(6-methyl-1,2,4,5-tetrazin-3-yl)phenyl)-3,7-dioxo-11,14,17-trioxa-2,8-diazanonadecan-19-yl)-1*H*-1,2,3-triazol-4-yl)methyl)-3,6-dioxo-3,6-dihydropyridazin-1(2*H*)-yl)propanoic acid **S13** (40 mg, 45 µmol) in DCM (2.5 mL) was added, at 21 °C, HATU (28.1 mg, 74 µmol), and DIPEA (5.78 mg, 45 µmol), and the reaction stirred for 5 min. Subsequently, to this solution was added at 21 °C a solution of 2-(2-(2-(2-azidoethoxy)ethoxy)ethoxy)ethan-1-amine (14.6 mg, 67 µmol) in DCM (2.5 mL), and the resulting solution was stirred at room temperature for 16 h. After this time, the reaction was diluted with EtOAc (10 mL) and washed with sat. aq. $NaHCO_3$ (3 × 10 mL), 1 M HCl (3 × 10 mL), H_2O (10 mL), brine (10 mL), dried ($MgSO_4$), filtered and the solvent removed *in vacuo*. The crude residue was purified by flash column chromatography (0-10% MeOH in DCM) to afford N^1-(2-(2-(2-(2-(4-((2-(1-azido-13-oxo-3,6,9-trioxa-12-azapentadecan-15-yl)-4,5-dibromo-3,6-dioxo-3,6-dihydropyridazin-1(2*H*)-yl)methyl)-1H-1,2,3-triazol-1-yl)ethoxy)ethoxy)ethoxy)ethyl)-N^5-(4-(6-methyl-1,2,4,5-tetrazin-3-yl)benzyl)glutaramide **1** (16.7 mg, 15 µmol, 34%) as a purple solid. ^{1}H NMR (500 MHz, $CDCl_3$) δ 8.52 (d, *J* = 8.4 Hz, 2H), 7.84 (s, 1H), 7.50 (d, *J* = 8.5 Hz, 2H), 6.74 (br. s, 1H), 6.65 (br. s, 1H), 6.43 (br. s, 1H), 4.65 (t, *J* = 6.8 Hz, 2H), 4.54 (d, *J* = 5.9 Hz, 2H), 4.50 (t, *J* = 4.9 Hz, 2H), 3.85 (t, *J* = 5.1 Hz, 2H), 3.70–3.50 (m, 24 H), 3.41 (td, *J* = 10.3, 5.1 Hz, 6H), 3.09 (s, 3H), 2.66 (t, *J* = 6.8 Hz, 2H), 2.38 (t, *J* = 7.0 Hz, 2H), 2.35 (t, *J* = 7.1 Hz, 2H), 2.01 (quint., *J* = 7.0 Hz, 2 H); ^{13}C NMR (125 MHz, $CDCl_3$) δ 172.9 (C), 172.8 (C), 169.5 (C), 167.4 (C), 164.0 (C), 153.4 (C), 153.0 (C), 143.7 (C), 141.0 (C), 136.5 (C), 135.7 (C), 131.0 (C), 128.6 (CH), 128.3 (CH), 125.1 (CH), 70.8 (CH_2), 70.7 × 2 (CH_2), 70.6 × 3 (CH_2), 70.4 (CH_2), 70.3 (CH_2), 70.1(CH_2), 69.9 (CH_2), 69.6 (CH_2), 69.3 (CH_2), 50.8 (CH_2), 50.5 (CH_2), 45.2 (CH_2), 43.3 (CH_2), 42.7 (CH_2), 39.5 (CH_2), 39.4 (CH_2), 35.5 (CH_2), 35.3 (CH_2), 34.0 (CH_2), 22.0 (CH_2), 21.3 (CH_3). IR (solid) 3306, 2919, 2101, 1722, 1634, 1543 cm^{-1}. HRMS (ESI) calcd for $C_{41}H_{58}Br_2N_{15}O_{11}$ [$M^{79}Br^{81}Br+H]^+$ 1096.2708; observed 1096.2782.

8.2.7. Compound **2**, bis(((1*R*,8*S*,9s)-bicyclo[6.1.0]non-4-yn-9-yl)methyl) (((oxybis(ethane-2,1-diyl))bis(oxy))bis(propane-3,1-diyl))dicarbamate

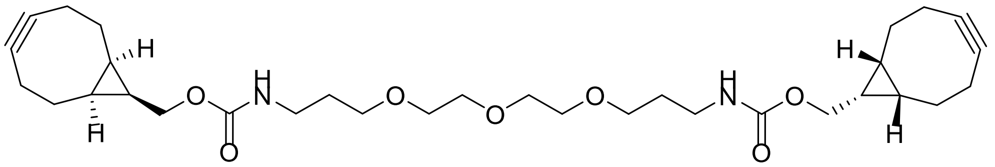

To a solution of 3,3'-((oxybis(ethane-2,1-diyl))bis(oxy))bis(propan-1-amine) (21.6 mg, 21.5 µL, 0.098 mmol) and NEt_3 (28.6 mg, 40.5 µL, 0.392 mmol) in DCM (4 mL) was added at room temperature ((1R,8S,9s)-bicyclo[6.1.0]non-4-yn-9-yl)methyl (2,5-dioxopyrrolidin-1-yl) carbonate (60 mg, 0.206 mmol), and the solution stirred at room temperature under an inert atmosphere for 3 h. After this time, the solvent was removed *in vacuo*, and the crude residue purified by flash column chromatography (0-100% EtOAc/cyclohexane) to afford bis(((1*R*,8*S*,9s)-bicyclo[6.1.0]non-4-yn-9-yl)methyl) (((oxybis(ethane-2,1-diyl))bis(oxy))bis(propane-3,1-diyl))dicarbamate **2** (23.4 mg, 40.9 µmol, 42%) as a yellow oil:
^{1}H NMR (700 MHz, $CDCl_3$) δ 5.17 (br. s, 2H), 4.14 (d, *J* = 7.9 Hz, 4H), 3.67–3.63 (m, 4H), 3.62–3.59 (m, 4H), 3.56 (t, *J* = 5.8 Hz, 4H), 3.32–3.26 (m, 4H), 2.33–2.17 (m, 12H), 1.78 (quint., *J* = 6.3 Hz, 4H), 1.38–1.32 (m, 2H), 0.94 (t, *J* = 9.7 Hz, 4 H); ^{13}C NMR (175 MHz, $CDCl_3$) δ 156.9 (C), 99.0 (C), 70.7 (CH_2), 70.4 (CH_2), 69.7 (CH_2), 62.7 (CH_2), 45.3 (CH), 39.2 (CH_2), 29.6 (CH_2), 29.2 (CH_2), 21.6 (CH_2), 20.2 (CH_2), 18.0 (CH). IR (thin film) 3331, 2917, 2866, 1694, 1522, 1243 cm^{-1}. HRMS (ESI) calcd for $C_{32}H_{48}N_2O_7$ $[M+H]^+$ 573.3462; observed 573.3533.

8.2.8. Compound **S14,** 3-(Bromo-2-methyl-3,6-dioxo-3,6-dihydropyridazin-1(2*H*)-yl)propanoic acid[4]

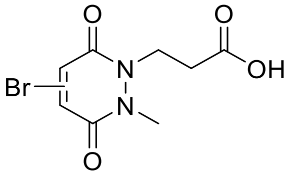

To a solution of di-*tert*-butyl-1-(3-(*tert*-butoxy)-3-oxopropyl)-2-methylhydrazine-1,2-dicarboxylate **S7** (1.50 g, 4.01 mmol) in AcOH (20 mL) was added bromomaleic anhydride (0.41 mL, 4.41 mmol) and the reaction heated under reflux for 4 h. After this time, the reaction mixture was concentrated *in vacuo* with toluene co-evaporation (3 × 30 mL, as an azeotrope). The crude residue was then purified by flash column chromatography (0% to 10% MeOH/EtOAc (1% AcOH)) to afford an inseparable mixture of regioisomers 3-(4-bromo-2-methyl-3,6-dioxo-3,6-dihydropyridazin-1(2*H*)-yl)propanoic acid **S14** and 3-(5-bromo-2-methyl-3,6-dioxo-3,6-dihydropyridazin-1(2*H*)-yl)propanoic acid **S14** (804 mg, 2.90 mmol, 72%) as a white solid. m.p. 142–145 °C. ^{1}H NMR (600 MHz, DMSO, regioisomers (1:1)) δ 7.59 (s, 1H), 7.58 (s, 1H), 4.28 (t, *J* = 7.4 Hz, 2H), 4.21 (t, *J* = 7.4 Hz, 2H), 3.58 (s, 3H), 3.50 (s, 3H), 2.62–2.57 (m, 4H). ^{13}C NMR (150 MHz,

DMSO-d_6, regioisomers (1:1)) δ 172.0 (C), 171.9 (C), 155.4 (C), 155.1 (C), 153.5 (C), 153.2 (C), 135.8 (CH), 135.5 (CH), 132.8 (C), 132.3 (C) 42.7 (CH_2), 41.5 (CH_2), 34.2 (CH_3), 32.9 (CH_3), 31.8 (CH_2). IR (solid) 3058, 1722, 1619 cm^{-1}. LRMS (ESI) 277 (100, $[M^{79}Br+H]^+$), 279 (95, $[M^{81}Br+H]^+$), HRMS (ESI) calcd for $C_8H_{10}BrN_2O_4$ $[M^{79}Br+H]$+ 276.9818; observed 276.9820.

N.B. This batch of molecule **S14** was made previously in our lab, but we have chosen to include the experimental details here for ease of reference. Please see cited paper for spectral data.[4]

8.2.9. Compound **S15,** 2,5-Dioxopyrrolidin-1-yl 3-(4-bromo-2-methyl-3,6-dioxo-3,6-dihydropyridazin-1(2*H*)-yl)propanoate[4]

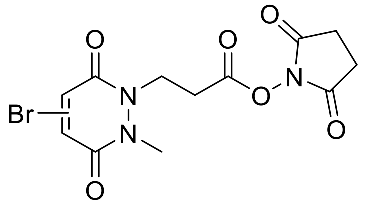

A solution of 3-(bromo-2-methyl-3,6-dioxo-3,6-dihydropyridazin-1(2*H*)-yl) propanoic acid **S14** (1.20 g, 4.33 mmol), in THF (10 mL) was cooled to 0 °C and was added N,N′-dicyclohexylcarbodiimide (1.0 g, 4.85 mmol). The homogenous solution was then stirred at 0 °C for 30 min. After this time, was added N-hydroxysuccinimide (535 mg, 4.67 mmol) and the reaction was stirred at 21 °C for a further 16 h. The newly formed heterogenous mixture was then filtered and the filtrate concentrated in vacuo. Purification of the crude residue by flash column chromatography (50% to 100% EtOAc/petrol) afforded an inseparable mixture of regioisomers 2,5-dioxopyrrolidin-1-yl 3-(4-bromo-2-methyl-3,6- dioxo-3,6-dihydropyridazin-1(2*H*)-yl)propanoate **S15** and 2,5-dioxopyrrolidin-1-yl 3-(5-bromo-2-methyl3,6-dioxo-3,6-dihydropyridazin-1(2*H*)-yl)propanoate **S15** (1.0 g, 2.69 mmol, 62%) as a white powder. m.p. 140-145 °C. ^{1}H NMR (500 MHz, DMSO-d_6, regioisomers (1:1)) δ 7.34 (s, 1H), 7.31 (s, 1H), 4.42 (t, J = 7.2 Hz, 2H), 4.36 (t, J = 7.2 Hz, 2H), 3.62 (s, 3H), 3.55 (s, 3H), 3.06–3.01 (m, 4H), 2.79 (s, 8H). ^{13}C NMR (125 MHz, DMSO, regioisomers (1:1)) δ 170.0 (C), 166.7 (C), 155.4 (C), 155.2 (C), 153.5 (C), 153.3 (C), 135.9 (CH), 135.4 (CH), 133.0 (C), 132.1 (C), 41.7 (CH_2), 40.6 (CH_2), 34.3 (CH_3), 33.0 (CH_3), 28.5 (CH_2), 25.4 (CH_2). IR (solid) 2944, 1808, 1778, 1731, 1632, 1596 cm^{-1}. LRMS (ESI) 374 (100, $[M^{79}Br+H]^+$), 376 (95, $[M^{81}Br+H]^+$), HRMS (ESI) calcd for $C_{12}H_{12}BrN_3O_6$ $[M^{79}Br+H]^+$ 373.9988; observed 373.9979.

N.B. This batch of molecule **S15** was made previously in our lab, but we have chosen to include the experimental details here for ease of reference. Please see cited paper for spectral data.[4]

8.2.10. Compound **S16**, ((1*R*,8*S*,9*S*)-Bicyclo[6.1.0]non-4-yn-9-yl)methyl (2-(2-(2-(3-(4,5-dibromo-2-methyl-3,6-dioxo-3,6- dihydropyridazin-1(2*H*)-yl)propanamido)ethoxy)ethoxy)ethyl) carbamate[4]

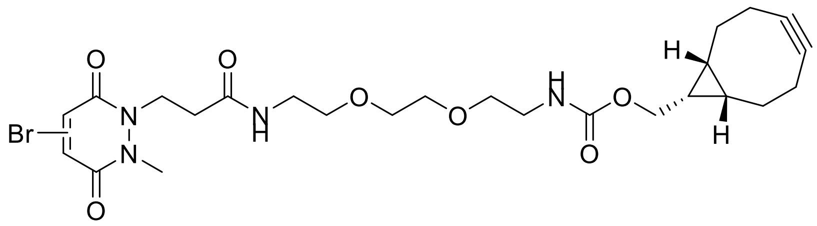

To a solution of 2,5-dioxopyrrolidin-1-yl 3-(bromo-2-methyl-3,6-dioxo-3,6-dihydropyridazin1(2H)yl) propanoate **S15** (50 mg, 0.110 mmol, pre-dissolved in MeCN (10 mL)), was added N- [(1R,8S,9S)-bicyclo[6.1.0]non-4-yn-9-ylmethyloxycarbonyl]-1,8-diamino-3,6-dioxaoctane (31 mg, 0.122 mmol) and the reaction mixture was stirred at 21 °C for 16 h. to afford an inseparable mixture of regioisomers ((1R,8S,9s)-bicyclo[6.1.0]non-4-yn-9-yl)methyl (2-(2-(2-(3-(5-bromo-2-methyl-3,6-dioxo-3,6-dihydropyridazin-1(2*H*)-yl)propanamido)ethoxy)ethoxy)ethyl)carbamate **S16** and ((1R,8S,9s)-bicyclo[6.1.0]non-4-yn-9-yl)methyl (2-(2-(2-(3-(4-bromo-2-methyl-3,6-dioxo-3,6-dihydropyridazin-1(2*H*)-yl)propanamido)ethoxy)ethoxy)ethyl)carbamate **S16** (42 mg, 0.07 mmol, 52%) as a yellow oil: ^{1}H NMR (600 MHz, CDCl3, regioisomers (1:1)) δ 7.86 (s, 0.5H), 7.80 (s, 0.5H), 7.38-7.36 (m, 2H), 6.42-6.39 (m, 1H), 5.81-5.78 (m, 1H), 5.33 (s, 0.5H), 5.27 (m, 0.5H), 4.44 (t, J = 7.0 Hz, 2H), 4.37 (t, J = 7.0 Hz, 2H), 4.18-4.15 (m, 4H), 3.73-3.67 (m, 3H), 3.66- 3.36 (m, 27H), 2.63-2.61 (m, 4H), 2.32-2.21 (m, 12H), 1.65-1.52 (m, 4H), 1.42-1.31 (m, 2H), 0.96- 0.93 (m, 4H). ^{13}C NMR (150 MHz, $CDCl_3$, regioisomers (1:1)) δ 171.5 (C),168.9 (C), 156.9 (C), 155.9 (C), 139.4 (C), 136.1 (C), 135.7 (C), 98.9 (C), 70.7 (CH_2), 70.3 (CH_2), 69.7 (CH_2), 62.3 (CH_2), 60.6 (CH_2), 44.2 (CH_2), 43.4 (CH_2), 40.8 (CH_2), 39.5 (CH_2), 34.7 (CH_3), 34.0 (CH_2), 33.4 (CH_2), 33.1 (CH_2), 29.2 (CH_2), 21.6 (CH_2), 21.0 (CH_2), 20.2 (CH_2), 17.9 (CH), 14.3 (CH). IR (thin film) 3331, 2989, 2857, 1715, 1645, 1572, 1534 cm^{-1}. LRMS (ESI) 583 (95, $[M^{79}Br+H]^+$), 585 (100, $[M^{81}Br+H]^+$), HRMS (ESI) calcd for $C_{25}H_{35}BrN_4O_7$ $[M^{79}Br+H]^+$ 583.1714; observed 583.1763.

N.B. This batch of molecule **S16** was made previously in our lab, but we have chosen to include the experimental details here for ease of reference. Please see cited paper for spectral data.[4]

8.3. Chemical biology section

8.3.1. General experimental details for chemical biology

Conjugation experiments were carried out in standard 1.5 mL Eppendorf tubes. All buffer solutions were prepared with double-deionised water and filter-sterilized for long-term storage. BBS refers to borate buffered saline (25 mM borate, 25 mM NaCl, pH 8.0, 2 mM EDTA), 5 × BBS refers to borate buffered saline (125 mM borate, 125 mM NaCl, pH 8.0, 10 mM EDTA) and PBS refers to phosphate buffered saline (10 mM phosphate, 2.7 mM KCl, 137 mM NaCl, pH 7.4) unless otherwise stated. Buffer exchange/ultrafiltration

was carried out using sample concentrators (Sartorius Stedim, Vivaspin, MWCO 3, 5 or 10 kDa) or desalting columns (Zeba™ Spin, ThermoFisher Scientific, 7k MWCO). In case where all traces of small molecule had to be removed (for the synthesis of protein-protein conjugates *via* click chemistry) the concentration of small molecule in the sample was diluted to 1 in 10 by Vivaspin, then the remainder removed by Zeba Spin; this was a crucial step for good conversion. Cetuximab (anti-EGFR), rituximab (anti-CD20), ontruzant (anti-HER2) and Herceptin (anti-HER2) were purchased from UCLH. Anti-ICOS (C398.4A) antibody was purchased from BioLegend. Anti-PD-1 (J116) and anti-CTLA4 (BN13) antibodies were purchased from BioXCell. Anti-CD3 (OKT3) antibody was purchased from either BioLegend or BioXCell. *Salmonella typhimurium* (ST) sialidase cysteine mutant **6** was expressed as described in a previous publication.[6] For protein A purification Pierce™ Protein A IgG binding Buffer was used. Cetuximab (anti-EGFR), anti-CD3 (OKT3) anti-CTLA4, anti-PD-1 and anti-ICOS antibody were digested using a standard antibody digestion protocol.[12,14] Purification by size exclusion chromatography (SEC) was carried out on an Agilent 1100 HPLC system (column: Superdex 200 increase, 10/300 GL) with a MALS system attached (Optilab T-rEX, Dawn8$^+$ Heleos, Wyatt Technology), equilibrated in PBS pH 7.5 with 0.05% NaN_3 at the ISMB biophysics institute. Detection was by absorption at 280 nm.

8.3.2. Protein LC-MS

Protein conjugates were prepared for analysis by desalting into distilled water (Zeba™ Spin, ThermoFisher Scientific, 7k MWCO) to achieve approximate concentrations of 4-5 µM (1.0 mg × mL^{-1}) and submitted to the UCL Chemistry Mass Spectrometry Facility at the Chemistry Department, UCL, for analysis on the Agilent 6510 QTOF LC-MS system (Agilent, UK). 10-20 µL of each sample was injected onto a PLRP-S, 1000 Å, 8 µM, 150 mM × 2.1 mM column (Agilent, UK), which was maintained at 60 °C. The separation was achieved using mobile phase A (5% MeCN in 0.1% formic acid) and B (95% MeCN, 5% water 0.1% formic acid) using a gradient elution (Table 1). The column effluent was continuously electrosprayed into the capillary ESI source of the Agilent 6510 QTOF mass spectrometer and ESI mass spectra were acquired in positive electrospray ionisation (ESI) mode using the m/z range 1,000−8,000 in profile mode with Quad AMU set to 500. The raw data was converted to zero charge mass spectra using a maximum entropy deconvolution algorithm, over the appropriate regions as identified *via* the LC TIC trace, with the software, MassHunter (version B.07.00). The region of the LC TIC trace from which the raw data was extracted is shown in the upper left corner of the raw data and deconvoluted spectra.

Table 1 | **Gradient for LC-MS elution.**

System used: Agilent 6510 QTOF LC-MS (Agilent, UK). Column: PLRP-S, 1000 Å, 8 µM, 150 mM × 2.1 mM (Agilent, UK). Fa stands for formic acid.

Time (min)	%A (H_2O 0.1% Fa)	%B (MeCN 0.1% FA)
0	85	15
2	85	15
3	68	32
4	68	32
14	50	50
18	5	95
20	5	95
22	85	15
25	85	15

8.3.3. SDS-PAGE gels

Non-reducing glycine-SDS-PAGE with 10%, 12% or 15% acrylamide gels (based on protein size: 10% for 45-150 kDa, 12% for 40-90 kDa and 15% for 25-45 kDa) were performed following standard lab procedures. A 6% stacking gel was used and a broad-range MW marker (10–250 kDa, PageRuler™ Plus Pre-stained Protein Ladder, Thermo Scientific™) was co-run to estimate protein masses. Samples (6-10 µL at ~10 µM protein) were mixed with loading buffer (1-2 µL, composition for 6 × SDS: 1 g SDS, 3 mL glycerol, 6 mL 0.5 M Tris buffer pH 6.8, 2 mg bromophenol blue in 10 mL) and heated at 75 °C for 3 min (or 95 °C for 5 min in case of Fab_2-Sia-Biotin constructs). 6-8 µL of the samples was loaded into each well. The gels were run at 80 V for 15 min and 160 V for 40-60 min in 1 × SDS running buffer. The gels were stained with Coomassie Blue dye, and destained with distilled water under microwave irradiation.

8.3.4. UV-Vis spectroscopy

UV-Vis spectroscopy was used to determine the concentrations of protein constructs using a NanoDrop™ One microvolume UV-Vis spectrophotometer operating at RT. Sample buffer was used as blank for baseline correction. Extinction coefficients (Ɛ) for proteins and small molecules (at 280 nm) are listed below (Table 2). Where possible ε_{280} of proteins was determined from amino acid sequence information with tools such as ProtParam (https://web.expasy.org/protparam/). Where necessary, in lieu of sequence information, the generic value of 70000 $M^{-1}cm^{-1}$ was used for Fabs. The ε_{280} of the protein was totalled with that of the expected small molecule adducts to calculate the total Ɛ of the construct at 280 nm ($\Sigma\varepsilon_{280}$). The concentration (c) of the construct was determined by the following equation:

$$c = \frac{A_{280}}{\Sigma\varepsilon_{280}}$$

Table 2 **| Extinction coefficient (Ɛ) values for proteins and small molecules at 280 nm.**

**Protein**	**ε_{280} ($M^{-1}cm^{-1}$)**	**Small molecule**	**ε_{280} ($M^{-1}cm^{-1}$)**
Fab_{HER2} **S21**	71905	Br_2PD-BCN **3**	2275
Fab_{CD20} **S22**	82905	BrPD-BCN **S16**	2275
Fab_{EGFR} **S17**	72900	Br_2PD-Tet **4**	28340
$Fab_{Generic}$	70000	Br_2PD-Tet-N_3 **1**	28340
Sia **6**	57090		
$mAb_{Generic}$	220000		

8.3.5. Standard Antibody Digestion Protocol

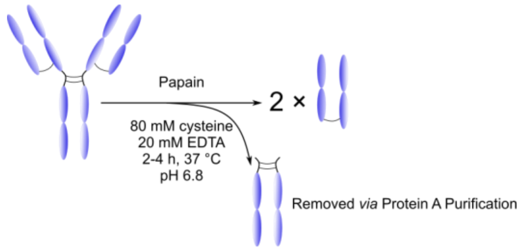

The standard antibody digestion protocol was used for the digestion of mAb_{EGFR} (Cetuximab), mAb_{CD3}, mAb_{ICOS}, mAb_{CTLA-4}, mAb_{PD-L1} and mAb_{PD-1}.

Full antibody (1-20 mg, 10 mg/mL) was buffer exchanged into digest buffer (20 mM NaH_2PO_4, 10 mM EDTA, 80 mM cysteine·HCl, pH 7.0). Immobilized papain (papain/antibody ratio 1:40 w/w) was washed three times with digest buffer and full antibody solution added. The reaction was incubated at 37 °C for 2-4 h under constant agitation (1100 rpm). The resin was separated from the digest using a filter column and washed with Pierce™ Protein A Binding Buffer three times. The digest was combined with the washes and the buffer was exchanged completely for Pierce™ Protein A Binding Buffer and the volume adjusted to 1.5 mL. The Fab and Fc were then separated by protein A purification. Representative yields: 28% Fab_{EGFR} **S17**, 71% Fab_{CD3} **S18**, 60% Fab_{PD-1} **7**, 60% Fab_{CTLA-4} **S19**, 30% Fab_{ICOS} **S20**.

8.3.6. Protein A purification

The sample was applied to a NAb protein A column (Thermo Scientific) and incubated at RT with end-over-end mixing for 10 min. The non-bound Fab fraction was eluted four times with Pierce™ Protein A Binding Buffer. The bound Fc fraction was eluted four times with Pierce™ IgG elution buffer or 0.1 M glycine buffer (pH 2.5), which was neutralised with 10% (V/V) of a 1.5 M Tris base, pH 8.8 solution. The Fab and Fc solutions were buffer exchanged into BBS.

8.3.7. Monomeric avidin agarose purification

A monomeric avidin agarose column (800 + 600 + 400 µL of Pierce™ Monomeric Avidin Agarose 50% aqueous slurry in a 0.8 mL Pierce™ Centrifuge Column, spun after each addition to remove liquid and thus pack column tightly) was washed with PBS (400 µL × 2), followed by PBS (400 µL × 22 mM biotin, pH 7.4)

to block all biotin binding sites. Then the reversible binding sites were re-generated by washing the column with elution buffer (400 µL × 4, glycine buffer pH 2.5). The column was equilibrated with PBS (400 µL × 2) and then 400 µL of sample was applied and incubated at RT with end-over-end mixing for 10 min. The non-bound fraction was eluted six times with PBS (400 µL × 6). The bound fraction was eluted six times with PBS (400 µL × 6, 10 mM phosphate, 2.7 mM KCl, 137 mM NaCl, 2 mM biotin, pH 7.4).

8.3.8. Digestion of anti-HER2 mAb (Herceptin and Ontruzant)

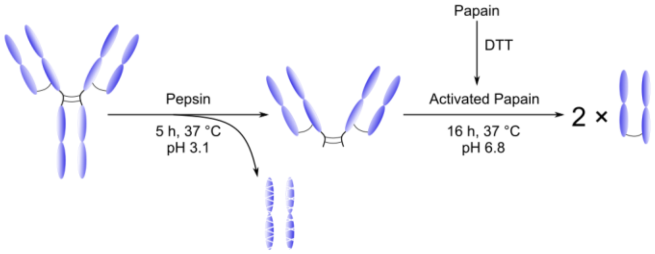

The mAb sample was buffer exchanged into sodium pepsin digest buffer (20 mM NaOAc, pH 3.1). Immobilized pepsin (732 µL) was washed 4 times with pepsin digest buffer and the mAb solution (1 mL, 107 µM) was added to this. The mixture was incubated for 5 h at 37 °C under constant agitation (1100 rpm). The resin was separated from the digest using a filter column and washed 3 times with papain digest buffer (50 mM sodium phosphate, 150 mM NaCl, 1 mM EDTA, pH 6.8). The digest was combined with the washes and the volume adjusted to 0.5 mL.

Immobilized papain (1.22 mL, 0.25 mg/mL) was activated with 10 mM DTT (in papain digest buffer) with constant agitation (1100 rpm) for 90 min at 37 °C. The resin was washed 4 times with papain digest buffer (without DTT) and the 0.5 mL of $F(ab')_2$ solution was added. The mixture was incubated for 24 h at 37 °C under constant agitation (1100 rpm). The resin was separated from the digest using a filter column, washed 3 times with PBS and the digest combined with the washes. The buffer was exchanged completely for PBS (10 mM phosphate, 2.7 mM KCl, 137 mM NaCl, pH 7.4), and the volume adjusted to 0.5 mL. Representative yield: 56% Fab_{HER2} **S21**.

8.3.9. Digestion of anti-CD20 mAb (Rituximab)

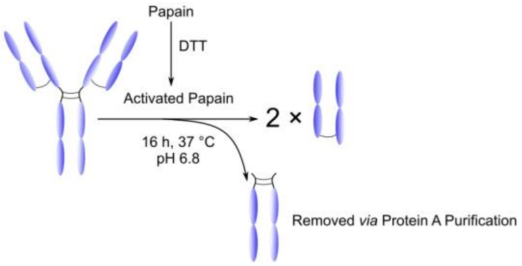

Immobilised papain (0.3 mL, 0.25 mg/mL) was activated with 10 mm DTT in papain digest buffer (50 mM phosphate, 1 mM EDTA, 150 mM NaCl, pH 6.8) under constant agitation (1100 rpm) for 1 h at 25 °C. The resin was washed with papain digest buffer (without DTT) four times and rituximab (3 mg in 0.5 mL of papain digest buffer) was added. The mixture was incubated for 16 h at 37 °C under constant agitation (1100 rpm). Then the resin was separated from the digest using a filter column and washed with Pierce™

Protein A Binding Buffer three times. The digest was combined with the washes and the buffer was exchanged completely for Pierce™ Protein A Binding Buffer and the volume adjusted to 1.5 mL. The Fab and Fc were then separated by protein A purification. Representative yields: 43% Fab_{CD20} **S22**, 31% Fc_{CD20} **S23**.

8.3.10. Reduction of Fab or sialidase **6**

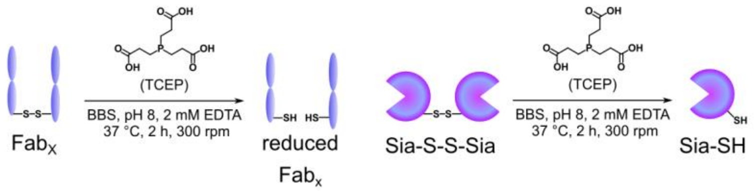

A solution of 20 mM TCEP was prepared by dissolving TCEP-HCl (15 mg) in 5 × BBS (2.6 mL). Fab, or sialidase **6** solution (10 µM, 200 µL) was prepared in BBS and 5 × BBS was added (100 µL), followed by addition of 5-60 equivalents of TCEP (20 mM in 5 × BBS, 0.5-6 µL) was added. The mixture was incubated for 120 min at 37 °C under constant agitation (300 rpm). The buffer was then exchanged for BBS. NB.: The 5 × BBS was employed to maintain the pH of the solution at 8, as TCEP-HCl is strongly acidic. TCEP-HCl equivalents used for reduction: 20 eq. for Fab_{EGFR} **S17**, 60 eq. for Fab_{CD3} **S18**, 60 eq. for $Fab_{PD\text{-}1}$ **7**, 20 eq. for Fab_{HER2} **S21** and 20 eq. for Fab_{CD20} **S22.**

8.3.11. Re-bridging of Fab or modification of sialidase **6** with pyridazinediones

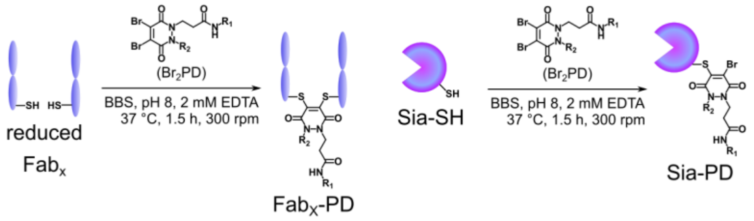

To a solution of reduced Fab_X, or sialidase **6** (100 µL, 20 µM) in BBS was added 5-20 equivalents of pyridazinedione Br_2PD-BCN **3** or Br_2PD-Tet-N_3 **1** (0.5-2 µL, 20 mM in DMSO) and the mixture incubated at 37 °C with constant agitation (300 RPM) over 90-120 min. The purity of the sample was assessed by non-reducing SDS-PAGE and high-resolution LC-MS. Representative yields: 57% Sia-Tet-N_3 **8**, 77% $Fab_{PD\text{-}1}$-Tet-N_3 **9**, 76% Fab_{HER2}-BCN **10**, 57% Fab_{CD3}-Tet-N_3 **14**, 65% Fab_{HER2}-Tet-N_3 **15**, 65% Fab_{EGFR}-Tet **S24**, 52% Fab_{CD20}-BCN **S25**, 58% Fab_{CD20}-Tet-N_3 **19**, 56% Fab_{HER2}-Tet **S26**, 43% Fab_{CD20}-Tet **S27**. Sia-BCN **S28**, Fab_{CD3}-Tet **S29** and Fab_{CD3}-BCN **S30** were also prepared in this manner.

8.3.12. General procedure for the preparation of Fab–Fab conjugates *via* SPIEDAC

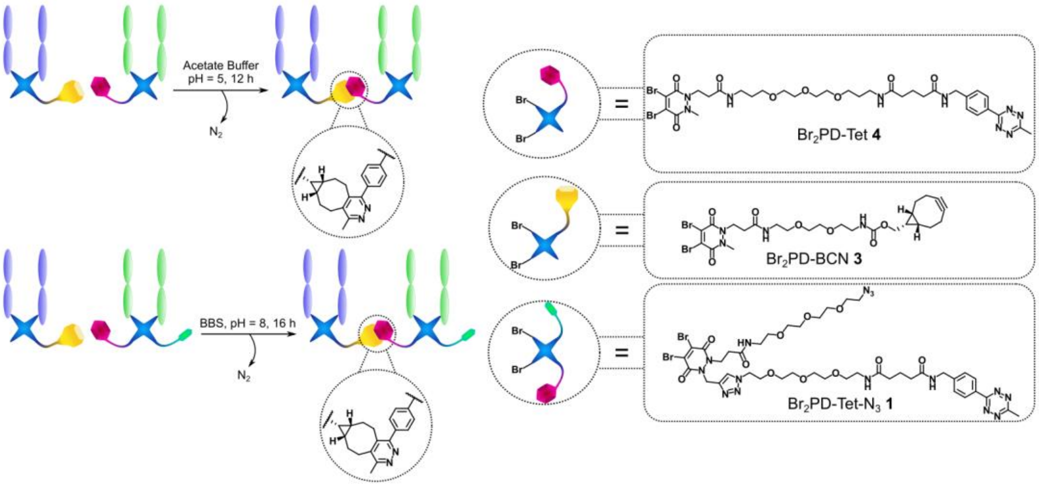

To a solution of Fab_X-BCN (50 µL, 200 µM, 10 nmol, 1 eq.) in acetate buffer (20 mM, pH 5.5) or BBS was added Fab_Y-Tetrazine or N_3-Fab_Y-Tetrazine (75 µL, 200 µM, 14 nmol, 1.4 eq.) and the reaction mixture was incubated at 30 °C or 37 °C for 16 h under an Argon atmosphere. After this time, the mixture was purified by protein A purification (if $Fab_X = Fab_{HER2}$) or SEC purification or taken forward without further purification. Representative yields: 24% Fab_{HER2}-Fab_{CD3}-N_3 **13**, 18% Fab_{CD3}-Fab_{HER2}-N_3 **28**, 11% Fab_{HER2}-Fab_{CD3} **30**. Fab_{HER2}-Fab_{CD20}-N_3 **22** and Fab_{CD20}-Fab_{HER2}-N_3 **S31** were also prepared in this manner.

N.B. initially acetate buffer was used, but after it was shown that the reaction worked in BBS at 37 °C, that protocol was used for convenience.

8.3.13. General procedure for preparation of trispecifics antibody Fab_{HER2}-Fab_{CD20}-Fab_{EGFR} **S33**

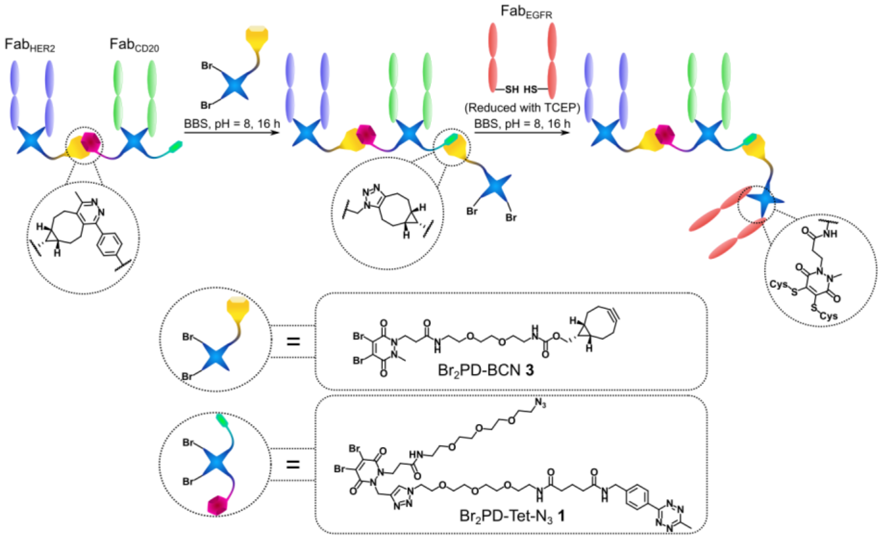

To a solution of Fab_{HER2}-Fab_{CD20}-N_3 **22** (50 µL, 200 µM, 10 nmol, 1 eq.) in BBS was added Br_2PD-BCN **3** (5 µL, 20 mM in DMSO) and the mixture incubated at 30 °C for 5 h to yield Fab_{HER2}-Fab_{CD20}-$PDBr_2$ **S32**. The excess Br_2PD-BCN **3** was removed by buffer exchange into BBS. To this solution was added reduced Fab_{EGFR} (100 µL, 200 µM, 20 nmol, 2 eq.), and the reaction mixture was incubated at 37 °C for 16 h. After this time, the mixture was purified by SEC. Representative yield: 12% Fab_{HER2}-Fab_{CD20}-Fab_{EGFR} **S33** (from Fab_{HER2}-Fab_{CD20}-$PDBr_2$ **S32**).

8.3.14. Preparation of bispecific-sialidase constructs; Fab_{CD20}-Fab_{HER2}-Sia_2 **S36** and Fab_{CD20}-Fab_{HER2}-Sia **S37**

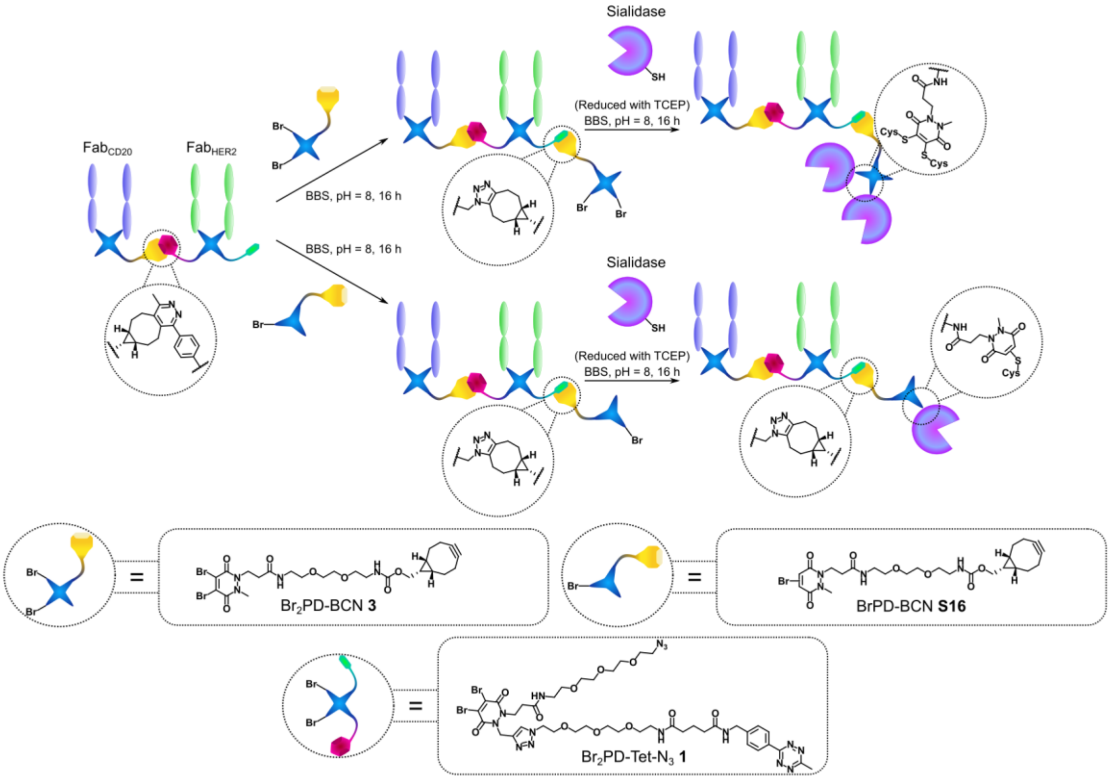

To a solution of Fab_{CD20}-Fab_{HER2}-N_3 **S31** (300 µL, 33.3 µM, 10 nmol, 1 eq.) in protein A binding buffer on a protein A column was added Br_2PD-BCN **3** or BrPD-BCN **S16** (5 µL, 20 mM in DMSO) and the mixture incubated at 30 °C for 16 h. After this time the excess Br_2PD-BCN **3** or BrPD-BCN **S16** was eluted (3 × 400 µL protein A binding buffer). To the Fab_{CD20}-Fab_{HER2}-$PDBr_2$ **S34** or Fab_{CD20}-Fab_{HER2}-PDBr **S35** conjugate on the resin, was added reduced sialidase (400 µL, 50 µM, 20 nmol, 2 eq.) in protein A binding buffer, and the reaction mixture was incubated at 30 °C for 20 h. After this time, the excess sialidase was washed off (3 x 400 µL protein A binding buffer) and the bound fraction eluted (3 × 400 µL, 0.1 M glycine buffer pH 2.5, which was neutralised after elution with 10% (V/V) of a 1.5 M Tris, pH 8.8 solution). The eluted fractions were then combined, and buffer exchanged into PBS (10 mM phosphate, 2.7 mM KCl, 137 mM NaCl, pH 7.0, 1 × Zeba spin). The mixture was then purified by SEC. Representative yield: 6% Fab_{CD20}-Fab_{HER2}-Sia_2 **S36** (from Fab_{CD20}-Fab_{HER2}-N_3 **S31**), 4% Fab_{CD20}-Fab_{HER2}-Sia **S37** (from Fab_{HER2}-BCN 10).

8.3.15. Preparation of the Fab_{HER2}–Sia–biotin conjugate **11** *via* SPIEDAC and SPAAC

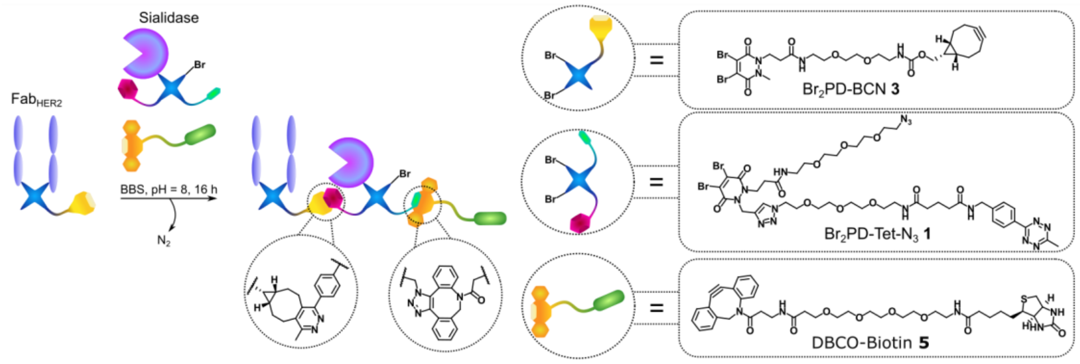

To a solution of Fab_{HER2}-BCN **10** (95 µL, 116 µM, 11.0 nmol, 1 eq.) in PBS (10 mM phosphate, 2.7 mM KCl, 137 mM NaCl, pH 7.0) was added Sia-Tet-N_3 **8** (87.6 µL, 168 µM, 14.7 nmol, 1.34 eq.) and DBCO-PEG_4-biotin **5** (3.7 µL, 20 mM in DMSO, 74 nmol, 5 eq.) and the reaction mixture was incubated at 37 °C for 16 h under an Argon atmosphere. After this time, the mixture was purified by monomeric avidin agarose purification. Yield: 21% Fab_{HER2}-Sia-biotin **11** (from Fab_{HER2}-BCN **10**).

8.3.1. Preparation of Fab_{HER2}-Fab_{CD3}-Biotin **12** *via* SPAAC

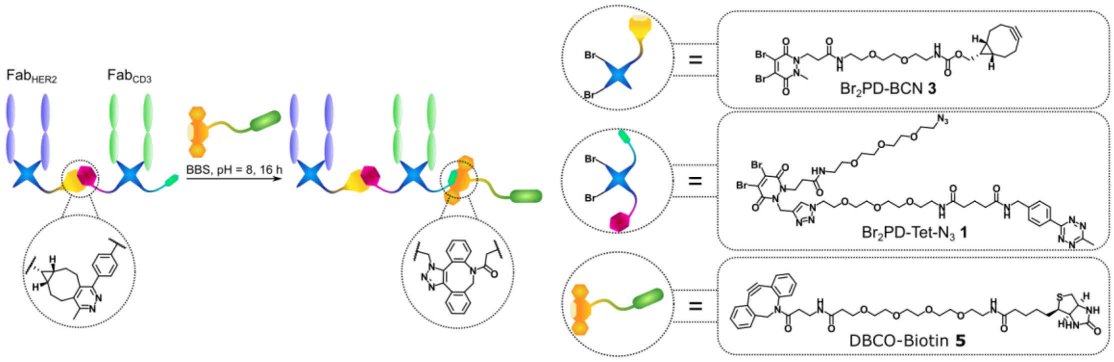

To a solution of purified Fab_{HER2}-Fab_{CD3}-N_3 **13** (100 µL, 15.6 µM, 1.56 nmol, 1 eq.) in BBS was added DBCO-PEG_4-biotin **5** (0.8 µL, 20 mM in DMSO, 15.6 nmol, 10 eq.) and the reaction mixture was incubated at 37 °C for 16 h under an Argon atmosphere. After this time the excess small molecule was removed by buffer exchange into BBS (1 × Zeba spin) to yield: 100% Fab_{HER2}-Fab_{CD3}-Biotin **12** (from purified Fab_{HER2}-Fab_{CD3}-N_3 **13**).

8.3.2. Preparation of the Fab_{HER2}-(biotin)-Fab_{CD20}-biotin conjugate 17 *via* SPIEDAC and SPAAC

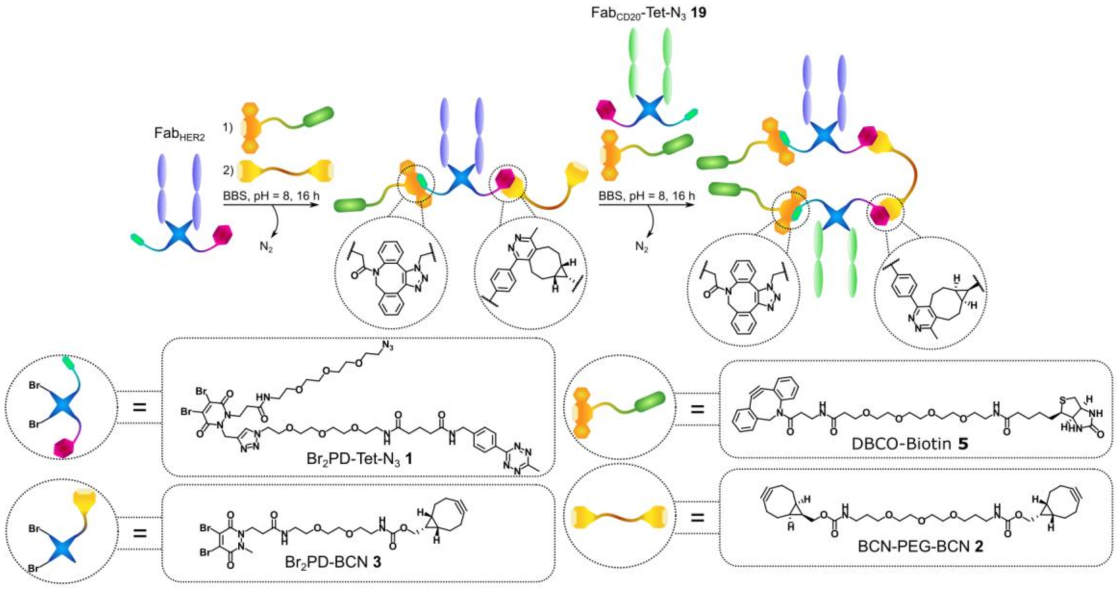

To a solution of Fab_{HER2}-Tet-N_3 **15** (92 µL, 130 µM, 12.0 nmol, 1 eq.) in BBS was added DBCO-PEG_4-Biotin **5** (6 µL, 20 mM in DMSO, 120 nmol, 10 eq.) and the reaction mixture was incubated at 37 °C for 1 h to generate Fab_{HER2}-Tet-Biotin **16**.

After this time, a dilute solution of BCN-PEG-BCN **2** was prepared by slowly adding BCN-PEG-BCN **2** (60 µL, 4 mM in DMSO, 240 nmol, 20 eq.) to BBS (400 µL) to avoid precipitation. To this solution was added the reaction mixture containing Fab_{HER2}-Tet-Biotin **16** and the reaction mixture was incubated at 37 °C for 5 h and then at 0 °C for 11 h under an Argon atmosphere to yield, after removal of excess small molecule (1 × Viva spin, 1 × Zeba spin), Fab_{HER2}-BCN-Biotin **18** (125 µL, 60 µM, 7.5 nmol, 63%).

To this solution was added Fab_{CD20}-Tet-N_3 **19** (105 µL, 51 µM, 5.4 nmol, 0.7 eq.) and DBCO-PEG_4-biotin **5** (4 µL, 20 mM in DMSO, 80 nmol, 15 eq.), and the reaction mixture was incubated at 37 °C for 16 h under an Argon atmosphere. After this time, the mixture was purified by SEC. Yield: 14% Fab_{HER2}-(biotin)-Fab_{CD20}-biotin **17** (from Fab_{CD20}-Tet-N_3 **19**).

8.3.3. Preparation of Fab_X-Fab_Y-Sia-Biotin species

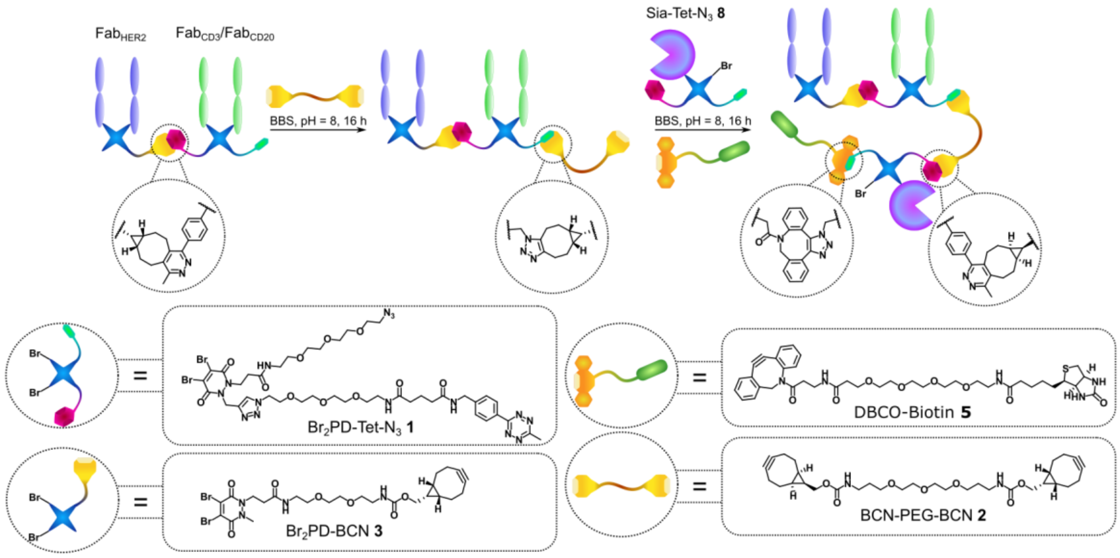

To a solution of Fab_{HER2}-Fab_{CD20}-N_3 **22** or Fab_{HER2}-Fab_{CD3}-N_3 **13** (100 µL, 163 µM, 16.3 nmol, 1 eq.) in acetate buffer (0.1 M, pH 5.0) was added BCN-PEG-BCN **2** (8.14 µL, 20 mM in DMSO, 163 nmol, 10 eq.) and the mixture incubated at 30 °C for 6 h under an Argon atmosphere to generate Fab_{HER2}-Fab_{CD20}-BCN **23** or Fab_{HER2}-Fab_{CD3}-BCN **26**, respectively. After this time the excess small molecule was removed by buffer exchange into PBS (10 mM phosphate, 2.7 mM KCl, 137 mM NaCl, pH 7.0, 1 × Viva Spin, 1 x Zeba spin), followed by addition of Sia-Tet-N_3 **8** (100 µL, 255 µM, 25.5 nmol, 1.56 eq.) in PBS pH 7 and DBCO-PEG_4-biotin **5** (6.38 µL, 20 mM in DMSO, 128 nmol, 7.9 eq.), and the reaction mixture was incubated at 22 °C for 16 h under an Argon atmosphere. After this time the excess small molecule was removed by buffer exchange into PBS (10 mM phosphate, 2.7 mM KCl, 137 mM NaCl, pH 7.0, 1 × Zeba spin) and the mixture was purified by SEC. Representative yields: 11% Fab_{HER2}-Fab_{CD20}-Sia-Biotin **21** (from Fab_{HER2}-BCN **10**), 6% Fab_{HER2}-Fab_{CD3}-Sia-Biotin **24** (from Fab_{HER2}-BCN), 20% Fab_{HER2}-Fab_{CD3}-Sia-Biotin **24** (from purified Fab_{HER2}-Fab_{CD3}-N_3 **13**).

8.3.4. Preparation of Fab_{CD3}-Fab_{HER2}-Fab_{PD-1}-Biotin CiTE **27**

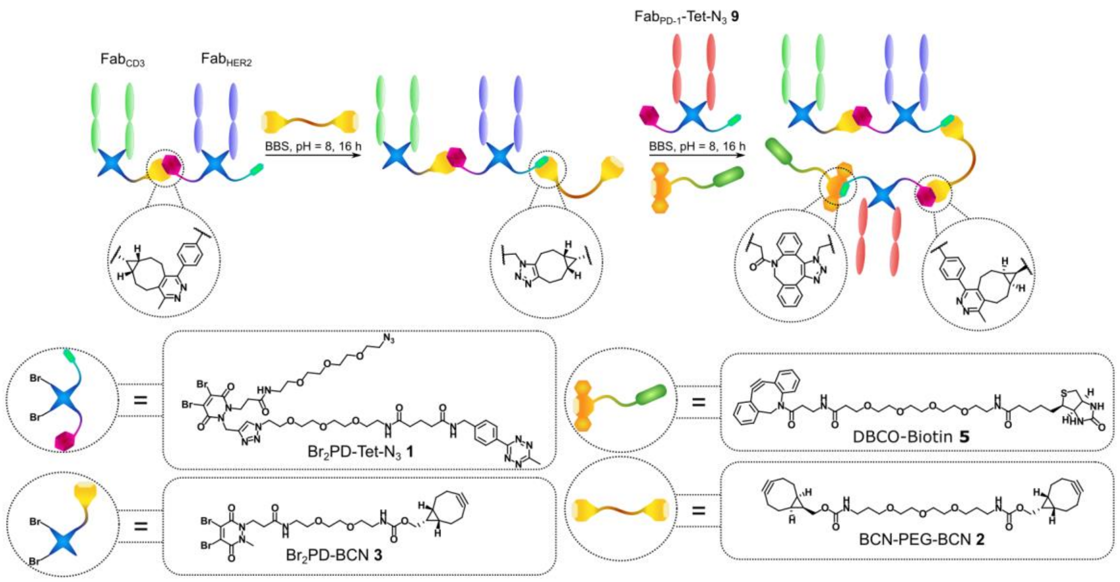

To BBS 350 µL (25 mM borate, 25 mM NaCl, pH 8.0, 2 mM EDTA) was added dropwise BCN-PEG-BCN **2** (50 µL, 4.2 mM in DMSO, 212 nmol, 20 eq.). To this solution was added Fab_{CD3}-Fab_{HER2}-N_3 **28** (115 µL, 61.6 µM, 7.1 nmol, 1 eq.) in BBS and the mixture incubated at 37 °C for 6 h. After this time, the excess small molecule was removed by buffer exchange into BBS (1 × Viva Spin, 1 x Zeba spin). To the resulting solution of Fab_{CD3}-Fab_{HER2}-BCN **29** (120 µL, 40.1 µM, 4.8 nmol) was added of Fab_{PD-1}-Tet-N_3 **9** (120 µL, 54.9 µM, 6.6 nmol, 1.34 eq.) in BBS and DBCO-PEG_4-biotin **5** (2.5 µL, 20 mM in DMSO, 33 nmol, 6.7 eq.), and the reaction mixture was incubated at 37 °C for 20 h under an Argon atmosphere. After this time the excess small molecule was removed by buffer exchange into PBS (1 × Zeba spin), and the mixture was purified by SEC. Representative yield: 12% Fab_{CD3}-Fab_{HER2}-Fab_{PD-1}-Biotin **27** (from Fab_{CD3}-Fab_{HER2}-N_3 **28**).

8.4. Cell Biology Section

8.4.1. Cell lines

SKBR3 (HTB-30™), HCC-1954 (CRL-2338™), BT-20 (HTB-19™), MDA-MB-468 (HTB-132™) and MDA-MB-231 (HTB-26™) cell lines were purchased from American Type Culture Collection and cultured in filtered Dulbecco's Modified Eagle's Medium/Nutrient Mixture F-12 Ham media with 10% heat-inactivated FBS and no added antibiotics or cultured as suggested. Cultures were grown in T25 and T75 flasks and maintained at 37 °C with 5% CO_2. Where required, cells were induced for the expression of human PD-L1 by incubation with interferon gamma (IFN-γ) (PeproTech 300-02) at 100 ng/mL for 48 h and lifted with Enzyme Free Cell Dissociation Solution PBS Based (MilliporeSigma S-014-M) before flow cytometry and cytotoxicity assays.

8.4.2. Human blood

Leukoreduction system (LRS) chambers were obtained from healthy anonymous human donors who gave informed consent at the Stanford Blood Center. Tier 1 characteristics of human biospecimens reported according to BRISQ guidelines[15] as follows. Biospecimen type: white blood cell concentrate of TrimaAccel®

LRS chamber recovered after Plateletpheresis procedure. Product contains PBMCs, red blood cells, plasma, and negligible amount of anticoagulant (ACD-A). Anatomical or collection site: vein (venipuncture). Biospecimen disease status and clinical characteristics of patients: healthy donors, not routinely tested for infectious disease markers. Vital state: alive. Collection mechanism and parameters: blood draw and LRS filtration for platelet donation. Platelets are collected by TrimaAccel® machine, using a 17 gauge needle for the stick. RBCs were returned to the donor. Mechanism of stabilization: anticoagulant ACD-A. Type of long-term preservation: peripheral blood mononuclear cells (PBMCs) were separated from the LRS chambers using dentistry gradient separation with Ficoll-Paque (GE Healthcare Life Sciences), biospecimens were frozen in FBS + 10% DMSO in liquid nitrogen. Constitution and concentration of fixative/preservation solution: heat inactivated foetal bovine serum (FBS) with 10% DMSO solution and frozen at -80 °C in an insulated cooler before being transferred to liquid nitrogen for long-term storage. Storage and shipping temperatures: LRS chambers were stored in a cooler for transport to the lab. Isolated PBMCs were stored in liquid nitrogen vapor (-196 °C). Storage duration: <1 day in LRS chamber. Duration varied as the units were collected at different times and sent to the lab in batches throughout the day, usually around 3-5 hours including drive time from the draw location to the lab. <2 years for frozen isolated PBMCs. Composition assessment and selection: none.

8.4.3. Binding and Desialylation with Flow Cytometry

Flow Cytometry experiments were performed on a MACSQuant® Analyzer 10 Flow Cytometer (Miltenyi Biotec) and analyzed using FlowJo, version 10.8.1. Cells were stained with either Zombie NIR (Biolegend 423106) or Zombie Violet (Biolegend 423113) Fixable Viability Kits according to manufacturer protocols and fixed with 4% Paraformaldehyde (Ted Pella 18505) prior to analysis. Washing and staining were performed in PBS with 0.5% BSA. Binding was determined by incubating the constructs with 100,000 cells for 30 min at 4 °C, followed by incubating with Streptavidin Alexa Fluor™ 647 conjugate (ThermoFisher S21374, 1:2000 dilution) for 30 min at 4 °C. Desialylation activity was determined by incubating cells for 30 min at 37 °C with the constructs, then detecting binding with a 1:1 molar mixture of recombinant Human Siglec-9 Fc (R&D Systems 1139-SL-050, 2 µg/mL) and rabbit IgG Alexa Flour 488-conjugated antibody (R&D Systems IC1051G, 1:375 dilution). Data points were normalized to the maximum mean fluorescence intensity.

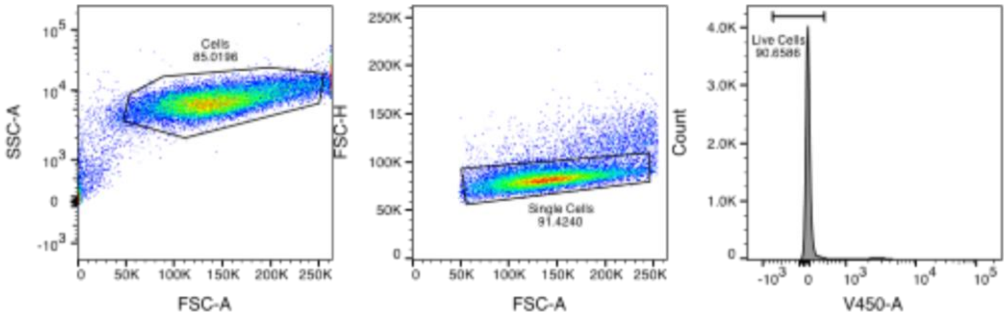

Figure 4 **| Representative flow cytometry gating.**

| Gating was performed using FlowJo software to eliminate debris (forward versus side scatter (FSC/SSC)) to analyze single cells (FSC-A/FSC-H), and to analyze live cells with either Zombie NIR (Biolegend 423106) or Zombie Violet (Biolegend 423113) Fixable Viability Kits.

8.4.4. T-cell isolation procedure and activation

LRS chambers were obtained from healthy human donors from the Stanford Blood Bank. Peripheral blood mononuclear cells (PBMC) were separated from the chambers using dentistry gradient separation with Ficoll-Paque (GE Healthcare Life Sciences). T cells were isolated using immunomagnetic negative selection EasySep™ Human T Cell Isolation Kit StemCell (STEMCELL Technologies, 17951) followed by activation for 5 days with human T-Activator CD3/CD28 Dynabeads™ (ThermoFisher, 11131D) and 30 IU/mL recombinant human Interleukin-2 (IL-2) (PeproTech, 200-02). For binding and desialylation experiments T cells from a separate donor were used for each replicate. For the cytotoxicity assay T cells from a single donor were used for all replicates.

8.4.5. Cytotoxicity assay

BiTE induced cellular toxicity was determined by lactate dehydrogenase (LDH) release with CyQUANT LDH Cytotoxicity Assay (ThermoFisher, C20300). 20,000 activated T cells were incubated with 10,000 target cells in the presence of constructs at various concentrations in 96-well plates. After 24 h at 37 °C, the supernatant from the co-incubation was measured using SpectraMax i3x (Molecular Devices) and SoftMax Pro 6.4.2. Specific killing was determined following the manufacturer's protocol.

8.4.6. Statistical analysis

Statistical analysis carried out with two-way ANOVA followed by post-hoc Tukey’s or Šídák's multiple comparisons tests with multiplicity-adjusted P values with $\alpha = 0.05$ in GraphPad Prism version 9. *$P < 0.05$, **$P < 0.01$, ***$P < 0.001$, ****$P < 0.0001$. Curves fitted with non-linear regression in GraphPad Prism version 9 with the following models: [Agonist] vs response (three parameters), One-site – Specific

binding, [Inhibitor] vs response (three parameters), [Agonist] vs response (three parameters). Best model identified with Prism's method-comparison function for each dataset.

Table 3 | **Two-way ANOVA table for Cytotoxicity assay of Fab_{HER2}-Fab_{CD3}-Sia-Biotin CiTE 24 and Fab_{CD3}-Fab_{HER2}-$Fab_{PD\text{-}1}$-Biotin CiTE 27.**

A

Two-way ANOVA for BiTE vs CiTE cell-kill assay on MDA-MB-231 cells				Ordinary	
Alpha	0.05				
Source of Variation	% of total variation	P value	P value summary	Significant?	
Interaction	4.377	0.0023	**	Yes	
Concentration	74.92	<0.0001	****	Yes	
Construct	17.08	<0.0001	****	Yes	
ANOVA table	SS	DF	MS	F (DFn, DFd)	P value
Interaction	115.9	6	19.31	F (6, 24) = 4.828	P=0.0023
Concentration	1983	3	661	F (3, 24) = 165.3	P<0.0001
Construct	452	2	223	F (2, 24) = 56.51	P<0.0001
Residual	95.99	24	3.999		
Data summary					
Number of columns (Construct)	3				
Number of rows (Concentration)	4				
Number of values	36				

B

Two-way ANOVA for BiTE vs CiTE cell-kill assay on MDA-MB-231 cells activated with INF-γ				Ordinary	
Alpha	0.05				
Source of Variation	% of total variation	P value	P value summary	Significant?	
Interaction	15.89	<0.0001	****	Yes	
Concentration	65.62	<0.0001	****	Yes	
Construct	16.8	<0.0001	****	Yes	
ANOVA table	SS	DF	MS	F (DFn, DFd)	P value
Interaction	429.2	6	71.69	F (6, 24) = 37.50	P<0.0001
Concentration	1776	3	592	F (3, 24) = 299.7	P<0.0001
Construct	454.7	2	226.3	F (2, 24) = 118.9	P<0.0001
Residual	45.88	24	1.912		
Data summary					
Number of columns (Construct)	3				
Number of rows (Concentration)	4				
Number of values	36				

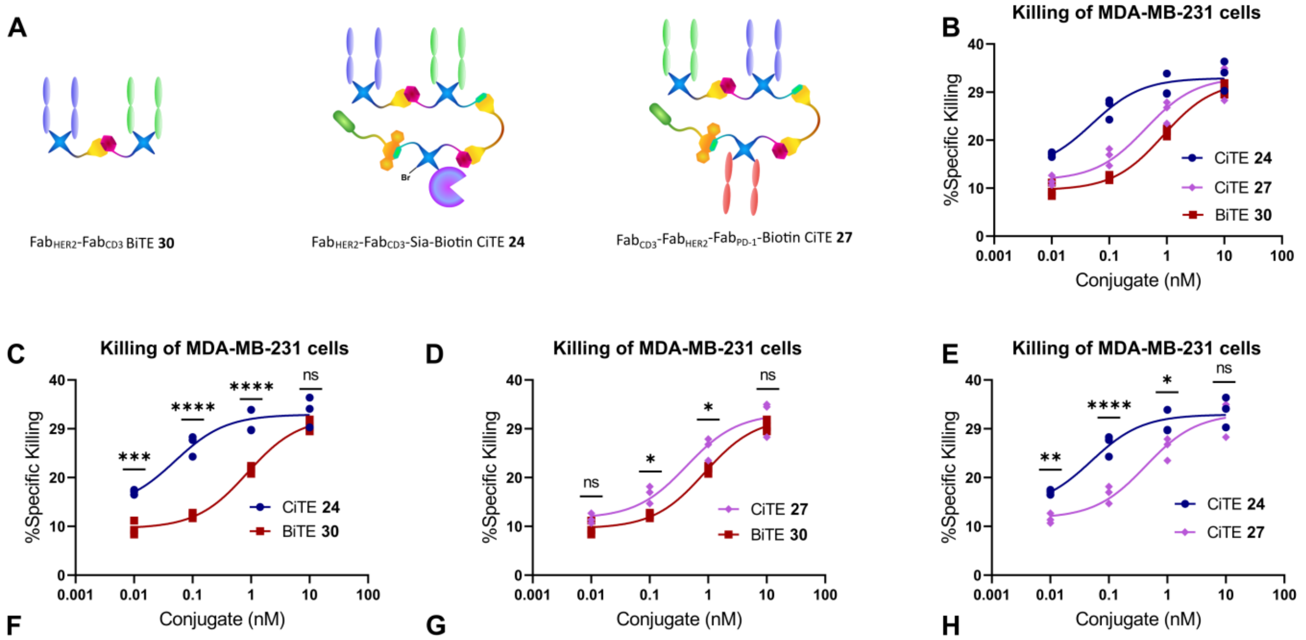

F

Post-hoc multiple comparisons table for BiTE vs CiTE cell-kill assay on MDA-MB-231 cells		
No induction of MDA-MB-231 PD-L1 expression		
BiTE **30** vs CiTE **24**		
Concentration	Tukey's adjusted P value	Significance
0.01 nM	0.0004	***
0.1 nM	<0.0001	****
1 nM	<0.0001	****
10 nM	0.1791	ns

G

Post-hoc multiple comparisons table for BiTE vs CiTE cell-kill assay on MDA-MB-231 cells		
No induction of MDA-MB-231 PD-L1 expression		
BiTE **30** vs CiTE **27**		
Concentration	Tukey's adjusted P value	Significance
0.01 nM	0.4621	ns
0.1 nM	0.0342	*
1 nM	0.0342	*
10 nM	0.4505	ns

H

Post-hoc multiple comparisons table for BiTE vs CiTE cell-kill assay on MDA-MB-231 cells		
No induction of MDA-MB-231 PD-L1 expression		
CiTE **24** vs CiTE **27**		
Concentration	Tukey's adjusted P value	Significance
0.01 nM	0.0076	**
0.1 nM	<0.0001	****
1 nM	0.013	*
10 nM	0.8148	ns

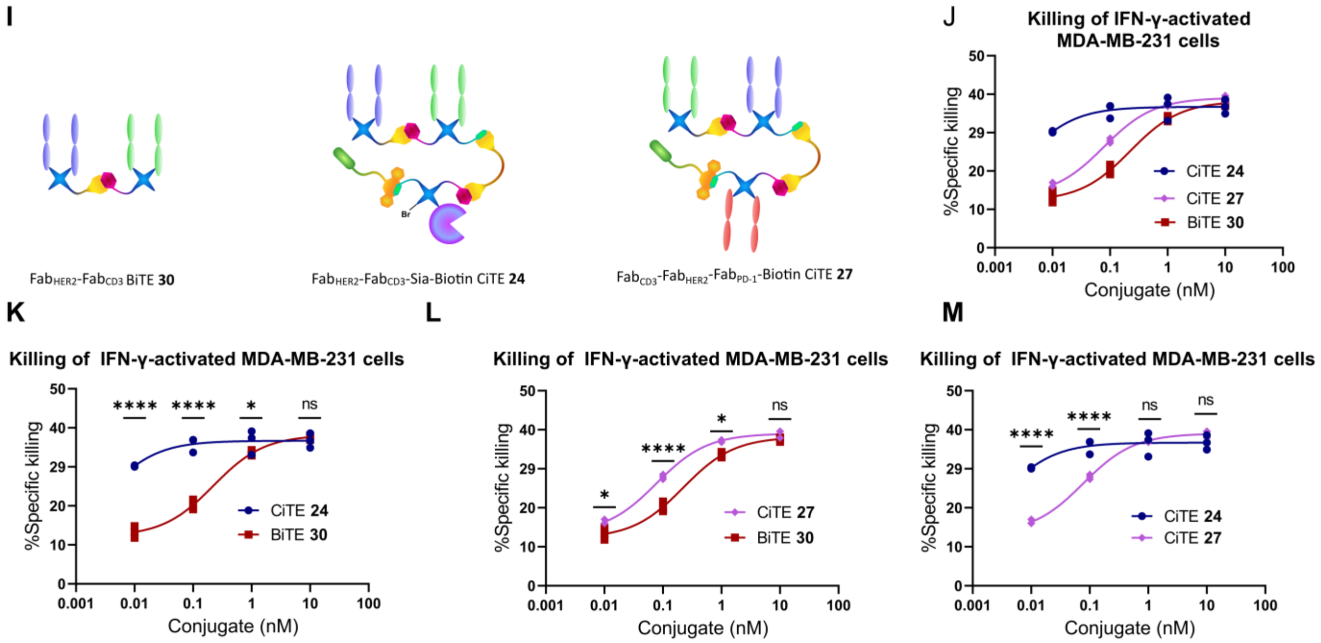

N

Post-hoc multiple comparisons table for BiTE vs CiTE cell-kill assay on MDA-MB-231 cells		
MDA-MB-231 PD-L1 expression induced with INF-γ		
BiTE **30** vs CiTE **24**		
Concentration	Tukey's adjusted P value	Significance
0.01 nM	<0.0001	****
0.1 nM	<0.0001	****
1 nM	0.0333	*
10 nM	0.7435	ns

O

Post-hoc multiple comparisons table for BiTE vs CiTE cell-kill assay on MDA-MB-231 cells		
MDA-MB-231 PD-L1 expression induced with INF-γ		
BiTE **30** vs CiTE **27**		
Concentration	Tukey's adjusted P value	Significance
0.01 nM	0.0333	*
0.1 nM	<0.0001	****
1 nM	0.0131	*
10 nM	0.4255	ns

P

Post-hoc multiple comparisons table for BiTE vs CiTE cell-kill assay on MDA-MB-231 cells		
MDA-MB-231 PD-L1 expression induced with INF-γ		
CiTE **24** vs CiTE **27**		
Concentration	Tukey's adjusted P value	Significance
0.01 nM	<0.0001	****
0.1 nM	<0.0001	****
1 nM	0.9105	ns
10 nM	0.132	ns

Figure 5 **| Cytotoxicity assay of Fab$_{HER2}$-Fab$_{CD3}$-Sia-Biotin CiTE 24 and Fab$_{CD3}$-Fab$_{HER2}$-Fab$_{PD-1}$-Biotin CiTE 27. A & I |** Structures of constructs used in assay. **B |** Cytotoxicity assay of Fab$_{HER2}$-Fab$_{CD3}$-Sia-Biotin CiTE **24** and Fab$_{CD3}$-Fab$_{HER2}$-Fab$_{PD-1}$-Biotin CiTE **27**. MDA-MB-231 cells were co-cultured with T cells (E:T ratio of 2:1) and treated with 0.01 – 10 nM of CiTE **24**, CiTE **27** or BiTE 30. MDA-MB-231 viability was assessed 24 h following treatment *via* LDH assay. **C & F |** Comparison of cytotoxicity of CiTE **24** and BiTE **30** on MDA-MB-231 cells. **D & G |** Comparison of cytotoxicity of CiTE **27** and BiTE 30 on MDA-MB-231 cells. **E & H |** Comparison of cytotoxicity of CiTE **27** and CiTE **24** on MDA-MB-231 cells. **J |** Cytotoxicity assay of Fab$_{HER2}$-Fab$_{CD3}$-Sia-Biotin CiTE **24** and Fab$_{CD3}$-Fab$_{HER2}$-Fab$_{PD-1}$-Biotin CiTE **27**. MDA-MB-231 cells, pre-incubated with IFN-γ to induce PD-L1 expression, were co-cultured with T cells (E:T ratio of 2:1) and treated with 0.01 – 10 nM of CiTE **24**, CiTE **27** or BiTE **30**. MDA-MB-231 viability was assessed 24 h following treatment *via* LDH assay. **K & N |** Comparison of cytotoxicity of CiTE **24** and BiTE **30**, with IFN-γ-activated MDA-MB-231 cells. **L & O |** Comparison of cytotoxicity of CiTE **27** and BiTE **30,** with IFN-γ-activated MDA-MB-231 cells. **M & P |** Comparison of cytotoxicity of CiTE **27** and CiTE **24,** with IFN-γ-activated MDA-MB-231 cells. **|** Statistical analysis carried out with two-way ANOVA followed by post-hoc Tukey's multiple comparisons test with multiplicity-adjusted P values with $\alpha = 0.05$. *P < 0.05, **P < 0.01, ***P < 0.001, ****P <0.0001. Data represented as individual datapoints, from three replicates. Curves fitted with non-linear regression with the following model: [Agonist] vs response (three parameters).

9. NMR spectra

9.2. Compound **S1**, tert-butyl (4-cyanobenzyl)carbamate[9]

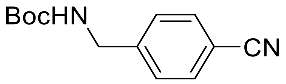

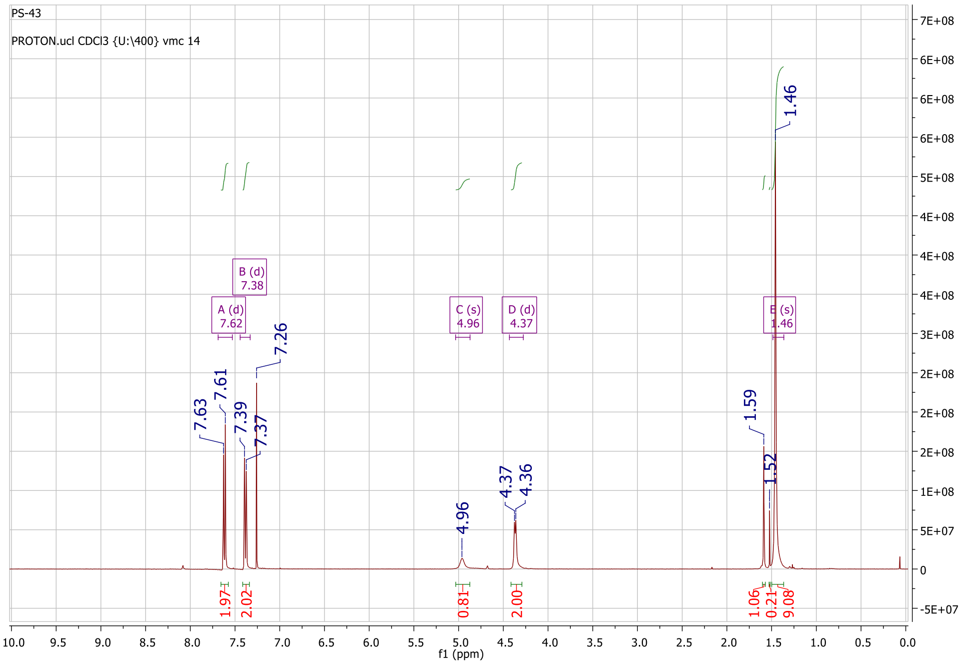

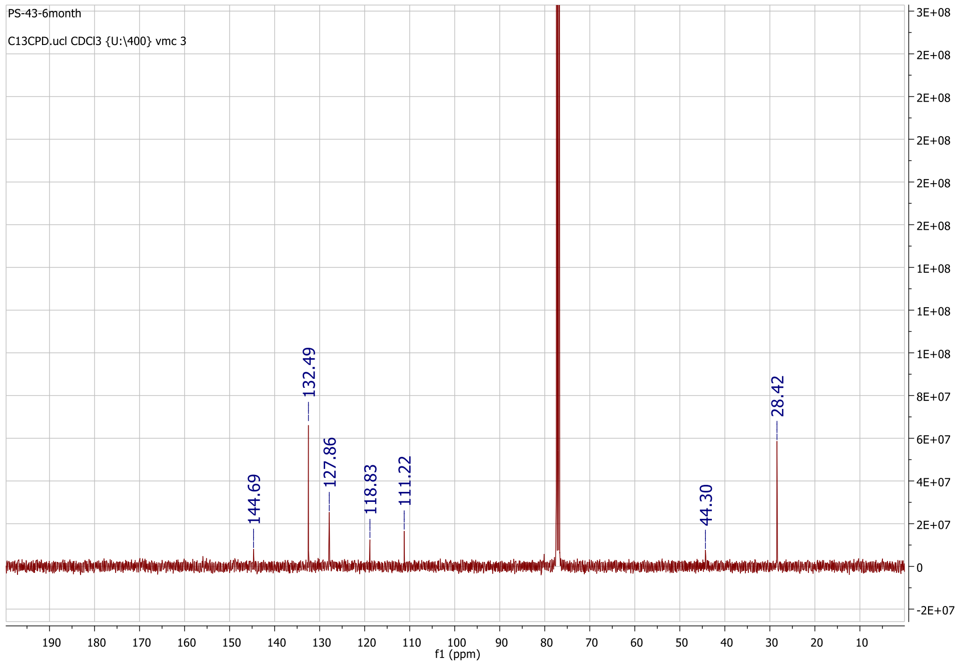

9.3. Compound **S2**, *tert*-butyl (4-(6-methyl-1,2,4,5-tetrazin-3-yl)benzyl)carbamate[9]

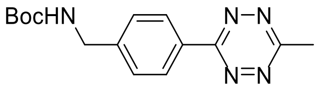

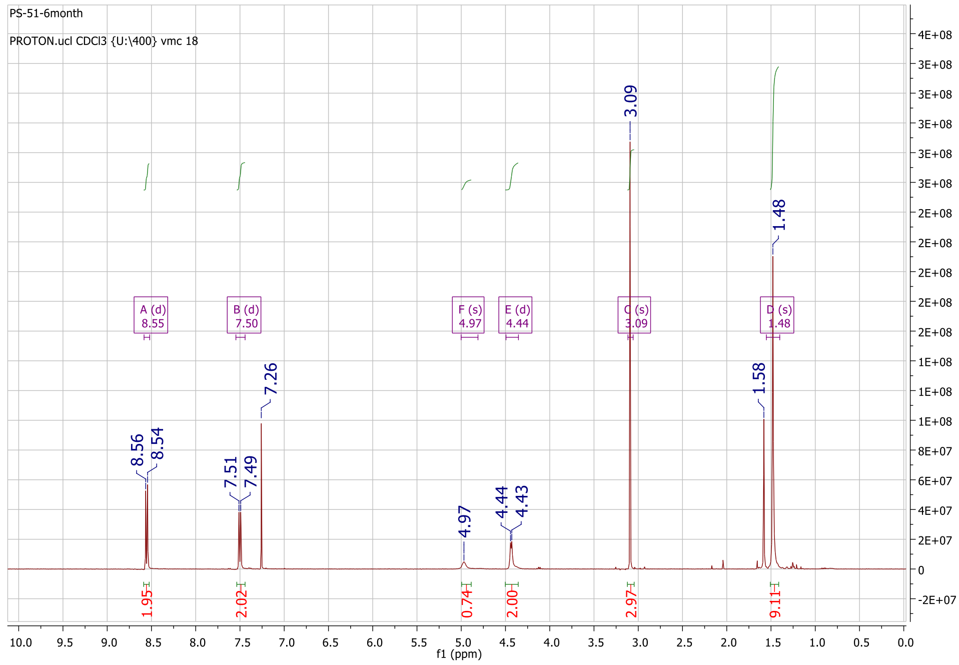

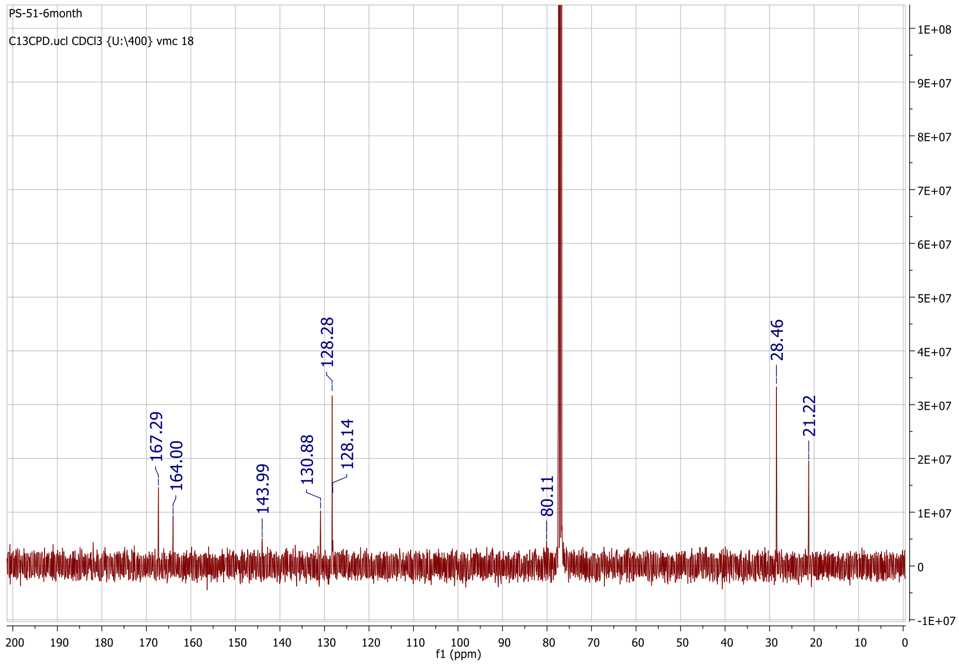

9.4. Compound **S3**, 5-((4-(6-methyl-1,2,4,5-tetrazin-3-yl)benzyl)amino)-5-oxopentanoic acid[11]

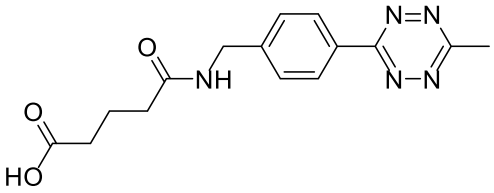

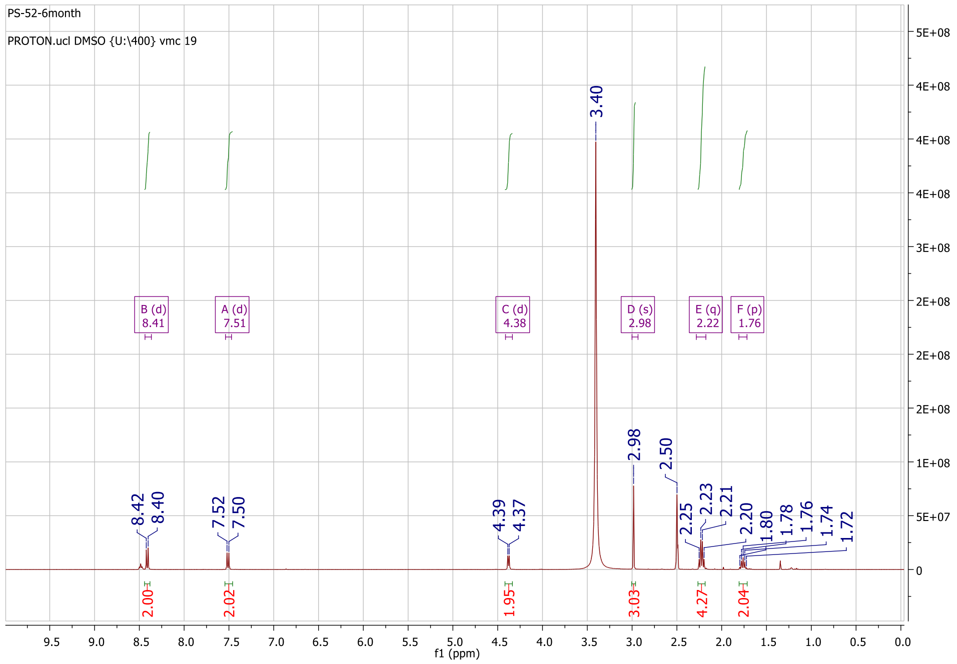

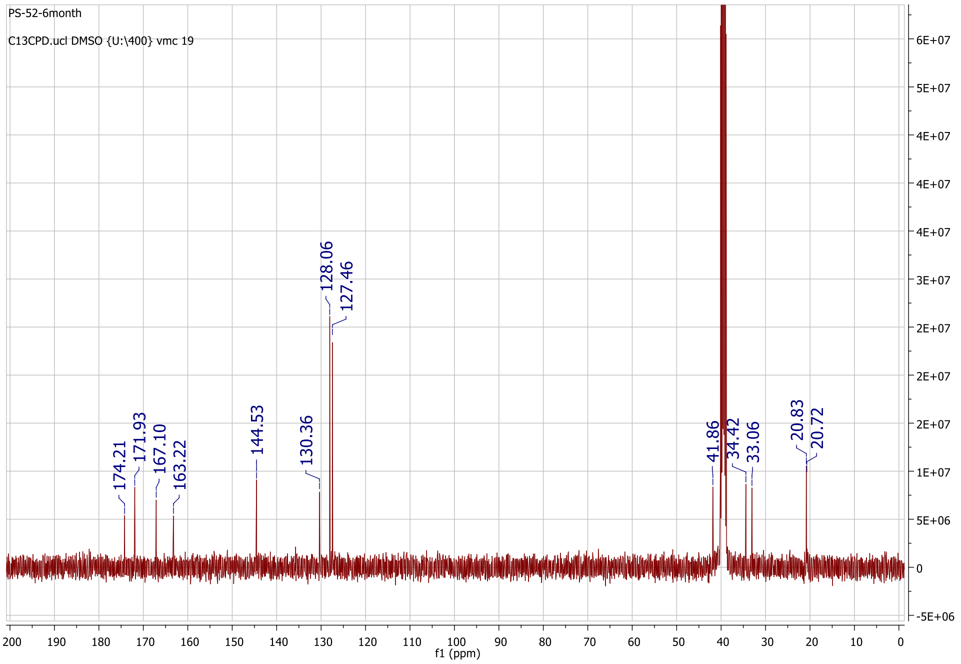

9.5. Compound **S4**, *N*[1]-(3-(2-(2-(3-aminopropoxy)ethoxy)ethoxy)propyl)-*N*[5]-(4-(6-methyl-1,2,4,5-tetrazin-3-yl)benzyl)glutaramide[12]

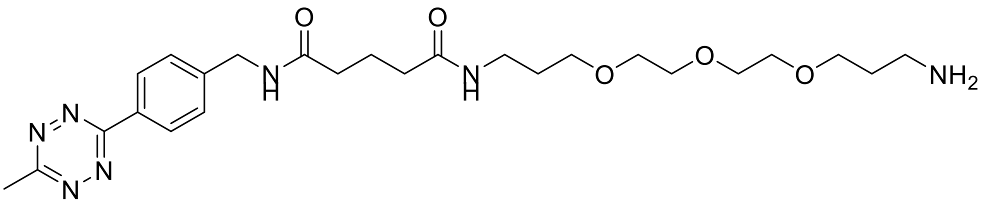

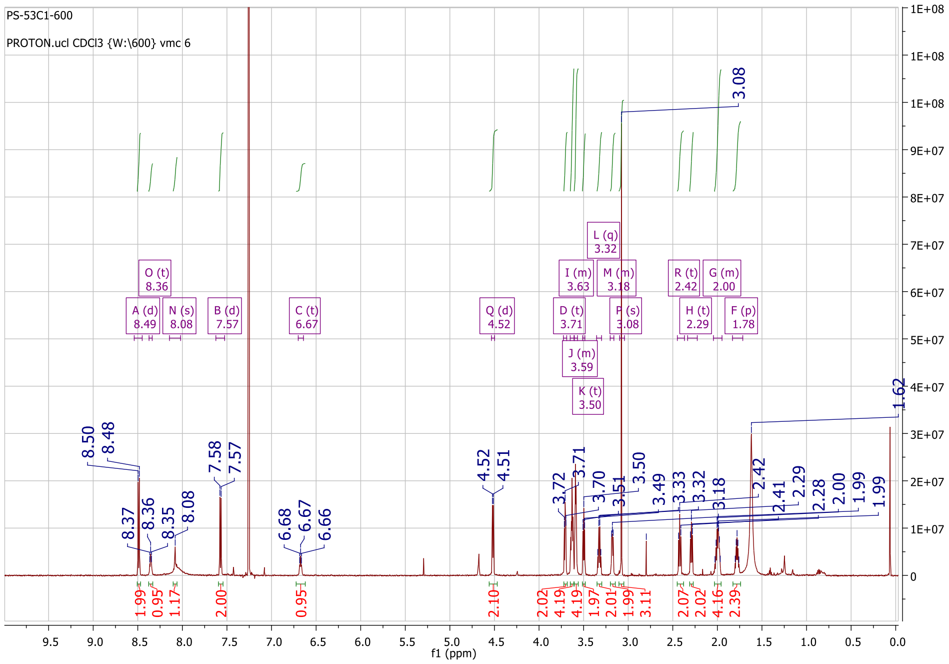

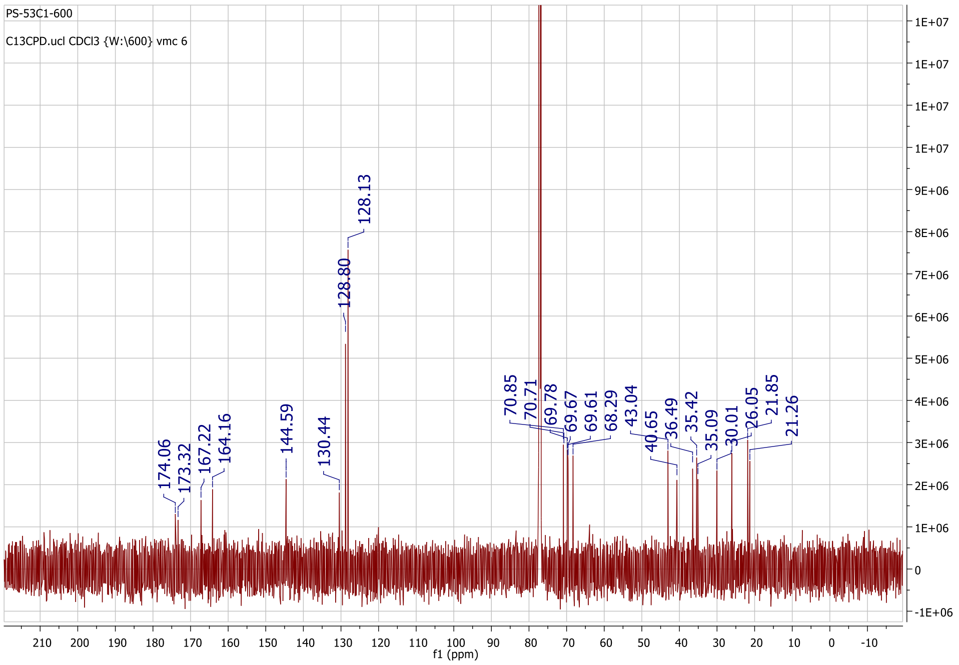

9.6. Compound **S5**, *N*[1]-(2-(2-(2-(2-azidoethoxy)ethoxy)ethoxy)ethyl)-*N*[5]-(4-(6-methyl-1,2,4,5-tetrazin-3-yl)benzyl)glutaramide[3]

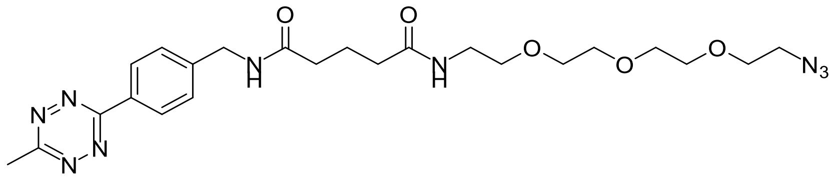

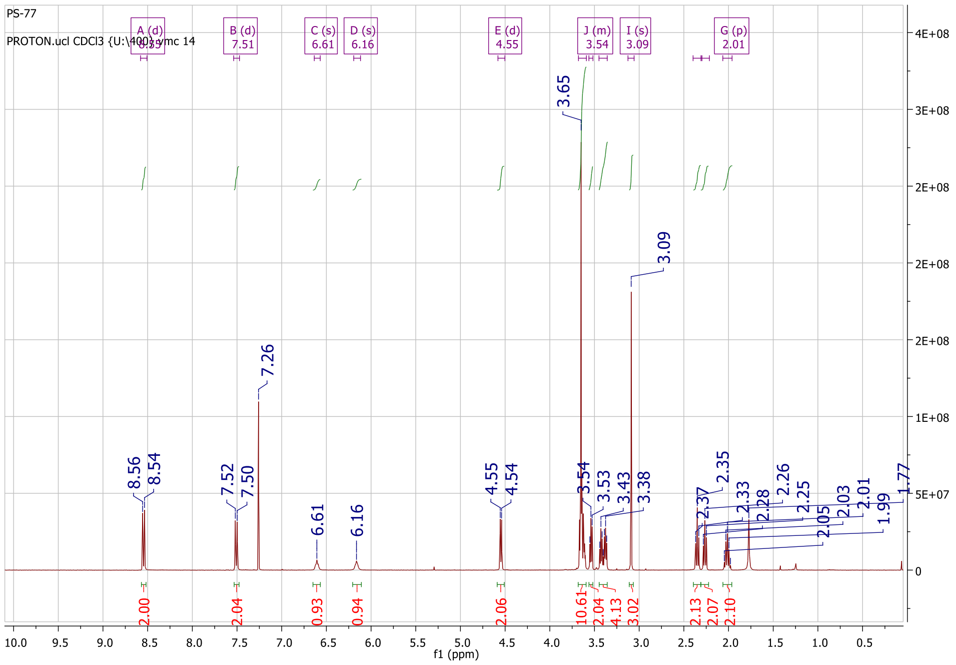

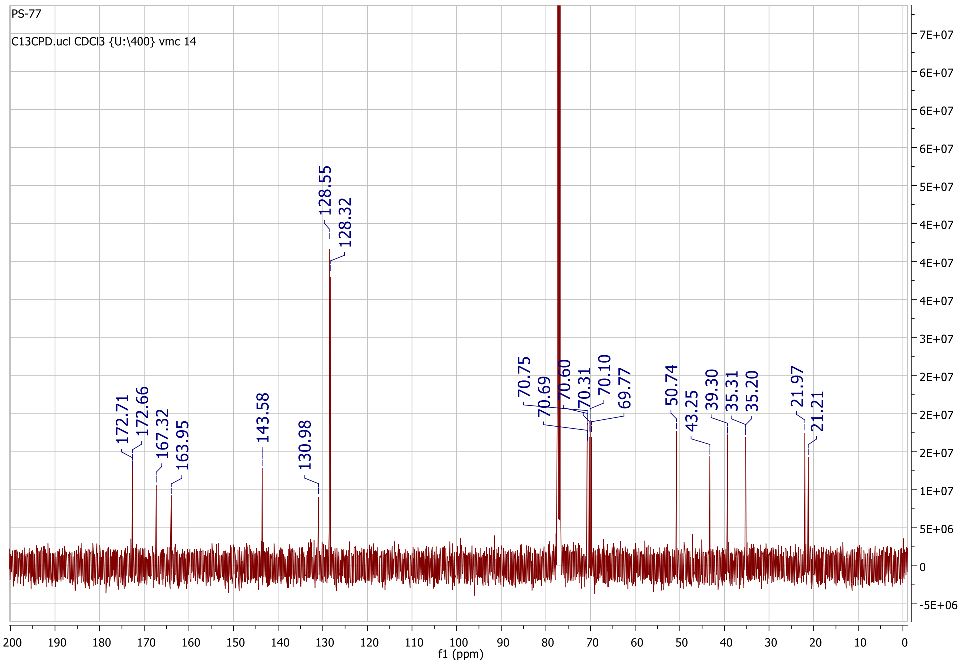

9.7. Compound **S6**, di-tert-butyl 1-methylhydrazine-1,2-dicarboxylate[2]

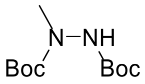

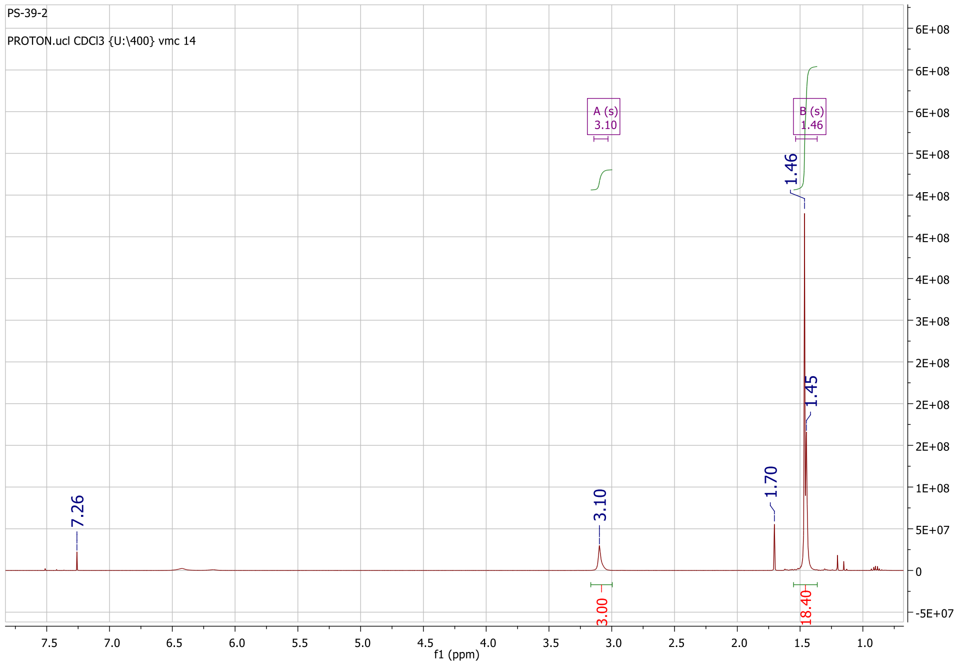

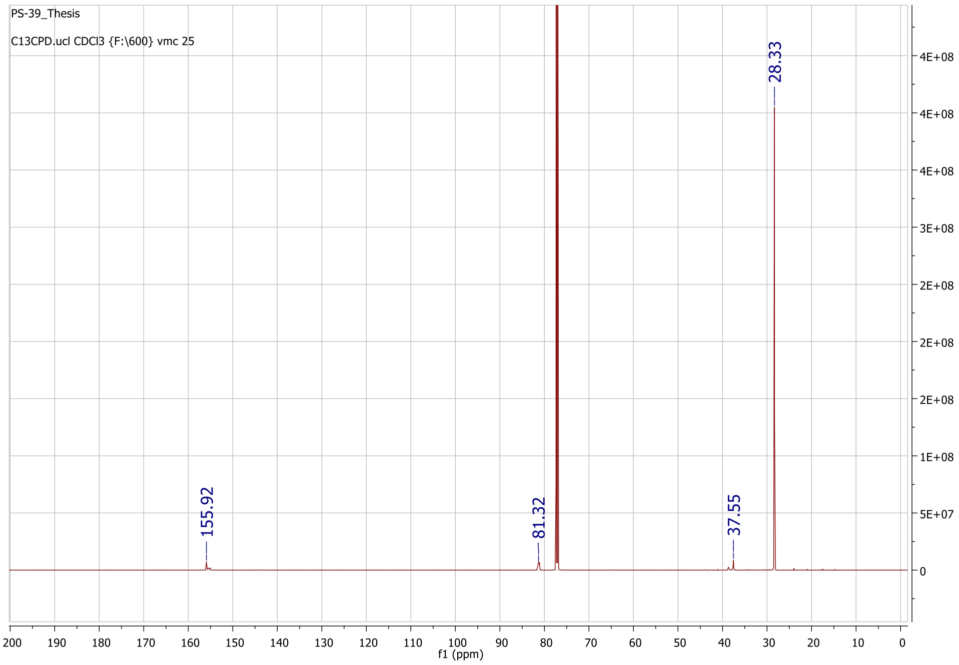

9.8. Compound **S7**, di-*tert*-butyl 1-(3-(tert-butoxy)-3-oxopropyl)-2-methylhydrazine-1,2-dicarboxylate[2]

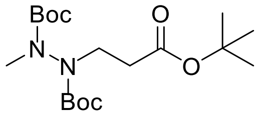

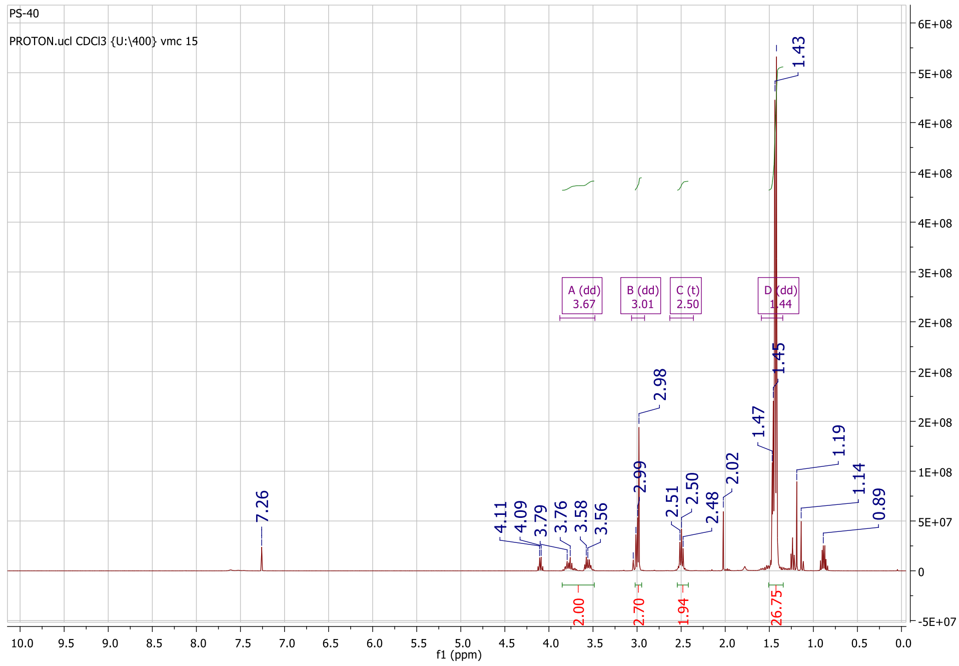

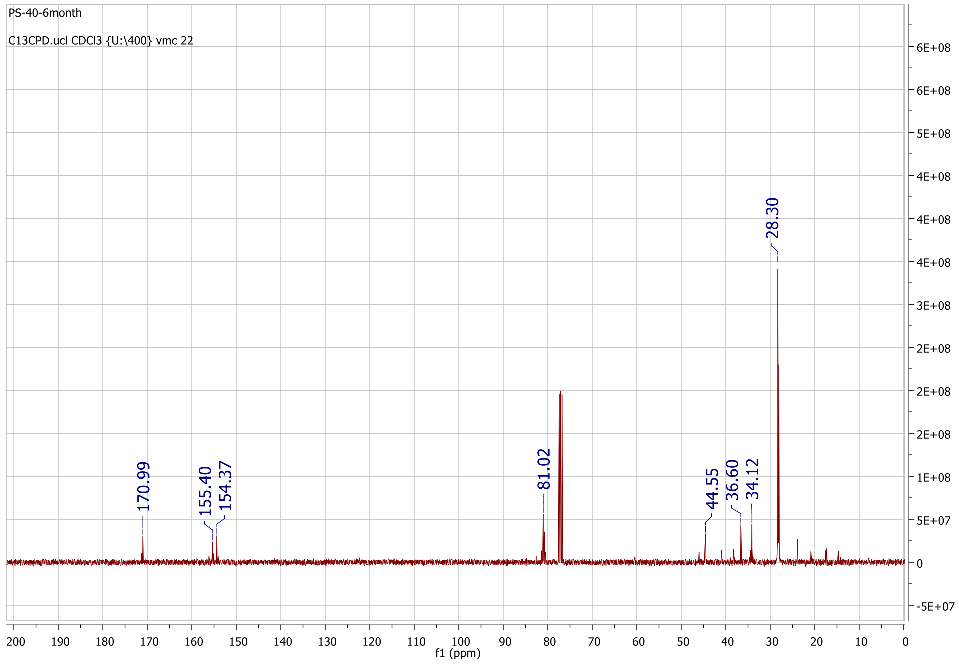

9.9. Compound **S8**, 3-(4,5-dibromo-2-methyl-3,6-dioxo-3,6-dihydropyridazin-1*(2H)-yl*)propanoic acid[2]

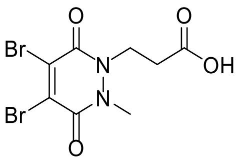

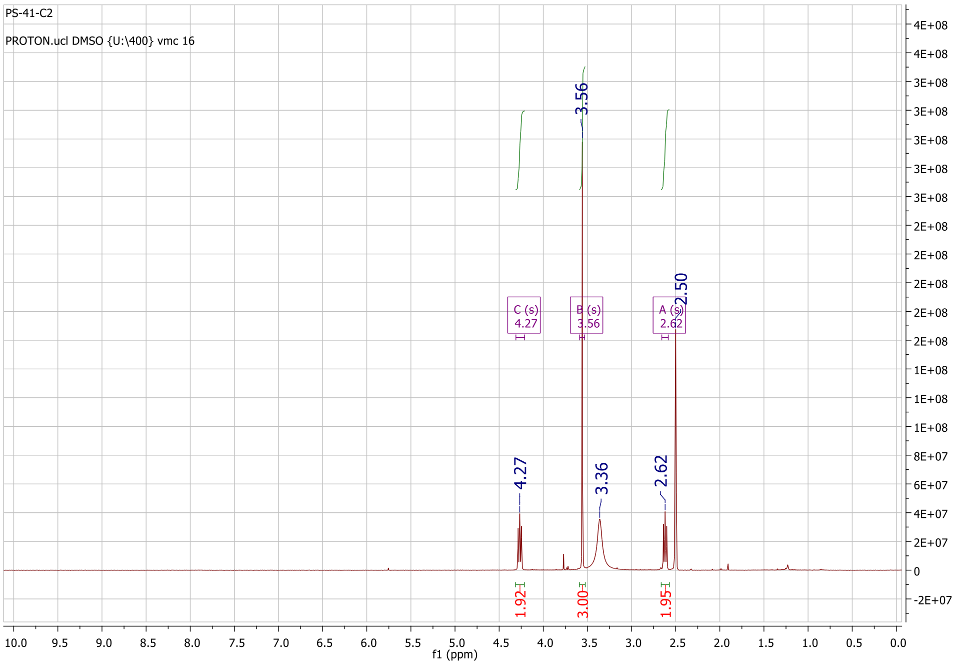

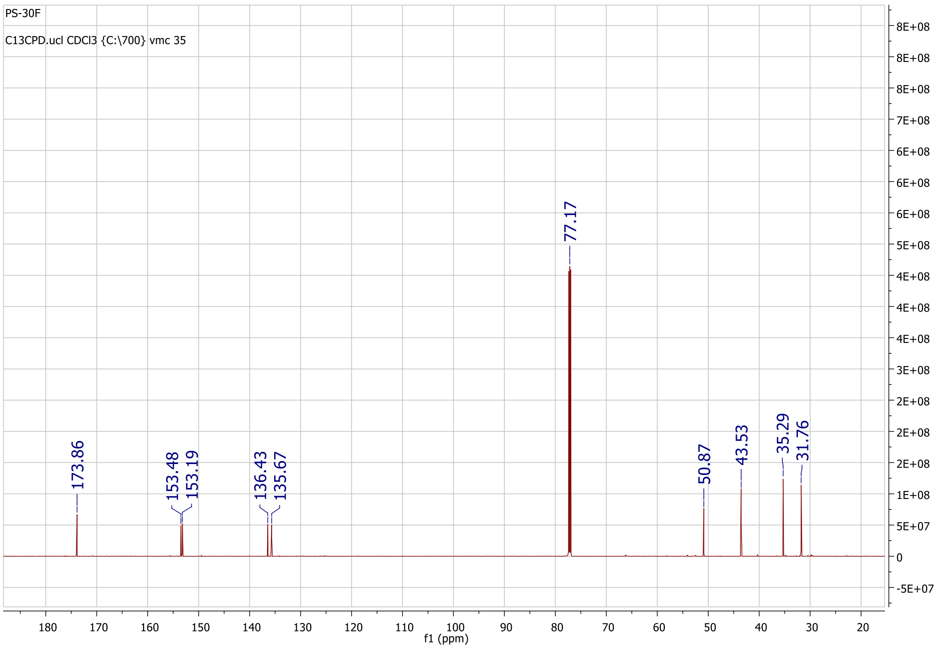

9.10. Compound **S9**, 2,5-dioxopyrrolidin-1-yl 3-(4,5-dibromo-2-methyl-3,6-dioxo-3,6-dihydropyridazin-1*(2H)-yl*)propanoate[2]

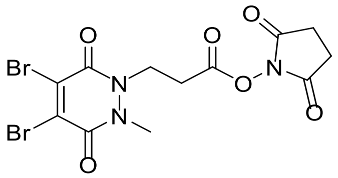

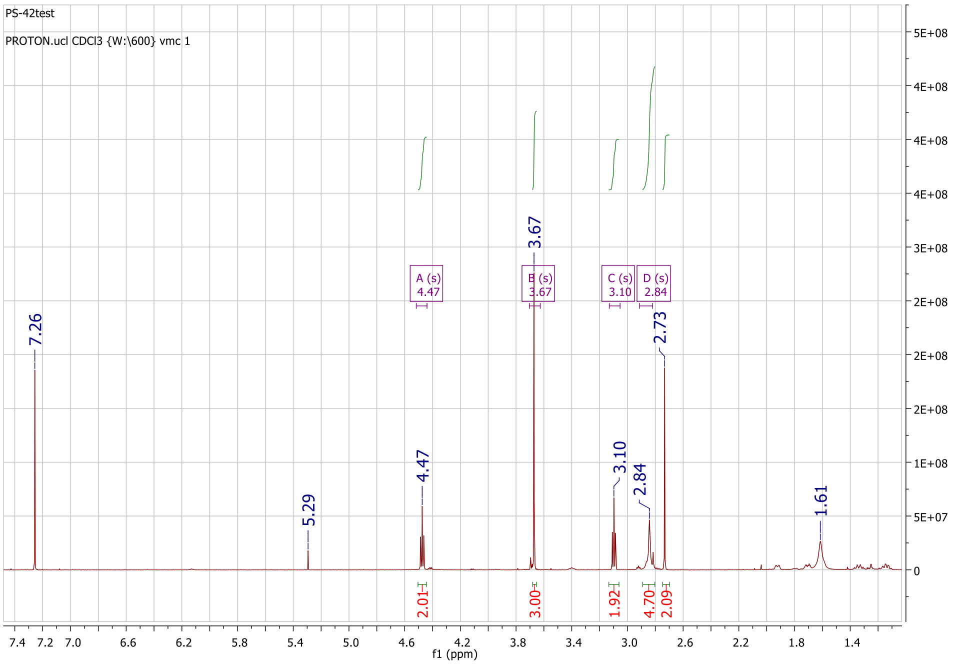

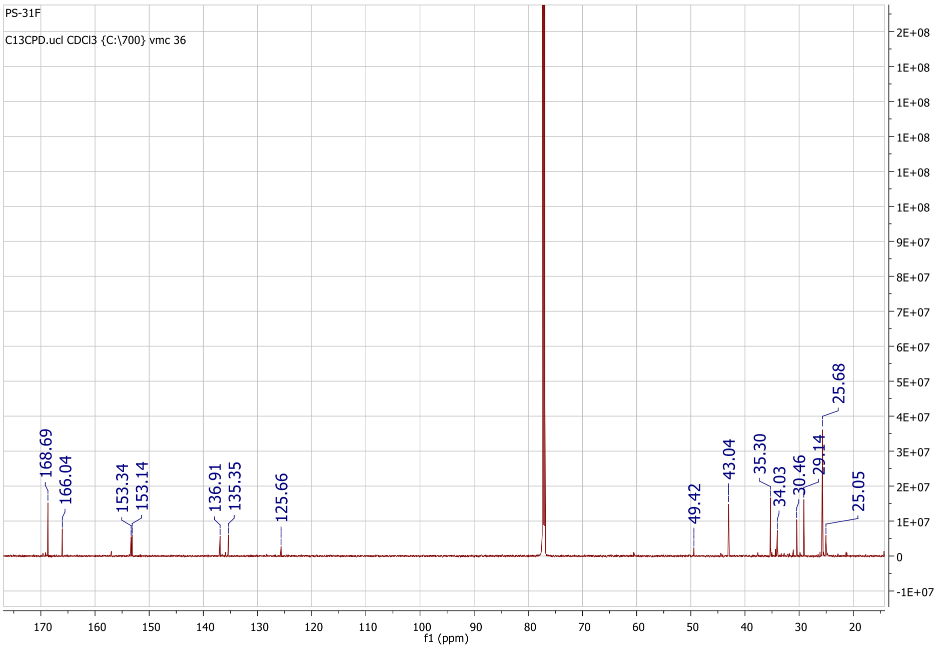

9.11. Compound **4**, *N*[1]-(17-(4,5-dibromo-2-methyl-3,6-dioxo-3,6-dihydropyridazin-1*(2H)-yl*)-15-oxo-4,7,10-trioxa-14-azaheptadecyl)-*N*[5]-(4-(6-methyl-1,2,4,5-tetrazin-3-yl)benzyl)glutaramide[12]

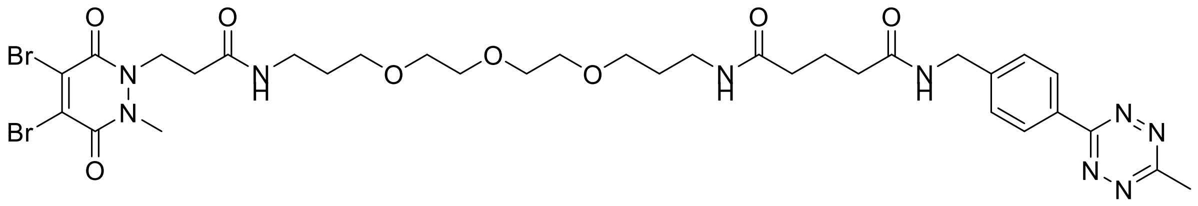

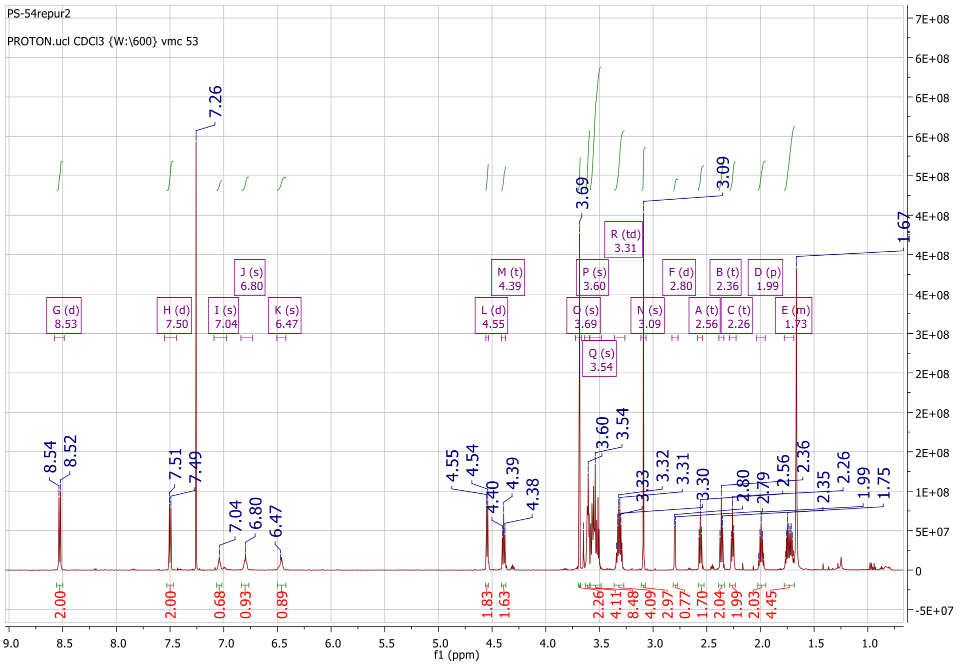

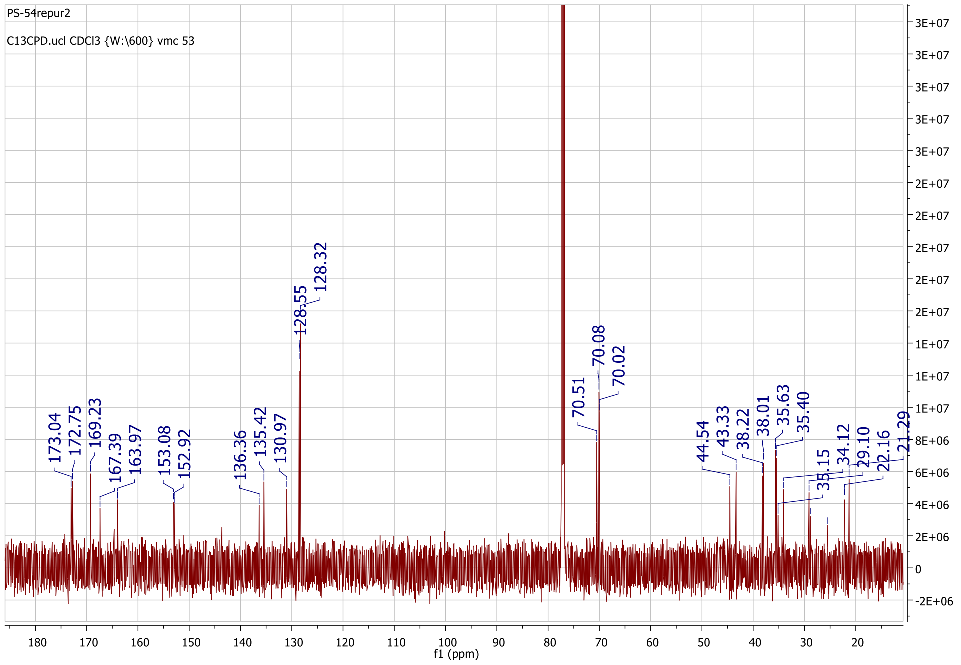

9.12. Compound **S10**, di-*tert*-butyl 1-(prop-2-yn-1-yl)hydrazine-1,2-dicarboxylate[13]

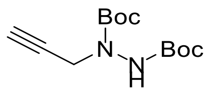

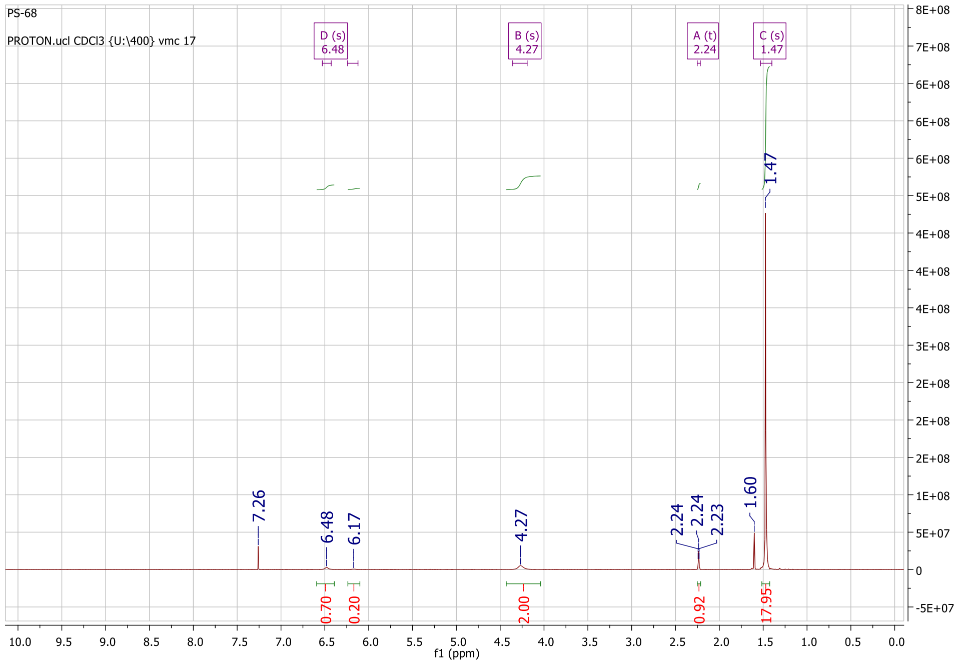

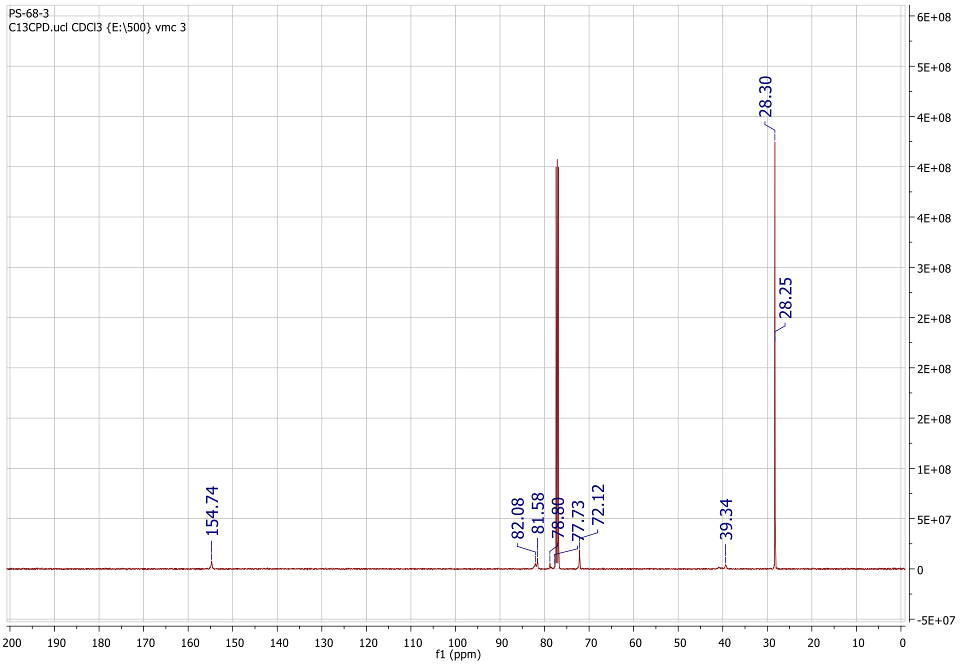

9.13. Compound **S11**, di-tert-butyl 1-(3-(tert-butoxy)-3-oxopropyl)-2-(prop-2-yn-1-yl)hydrazine-1,2-dicarboxylate[3]

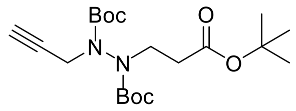

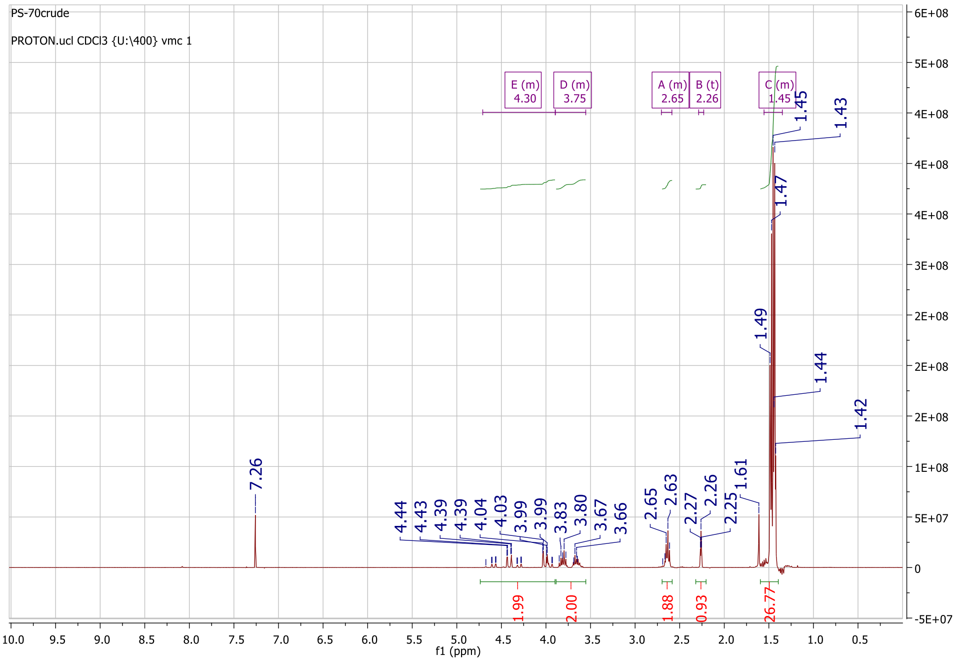

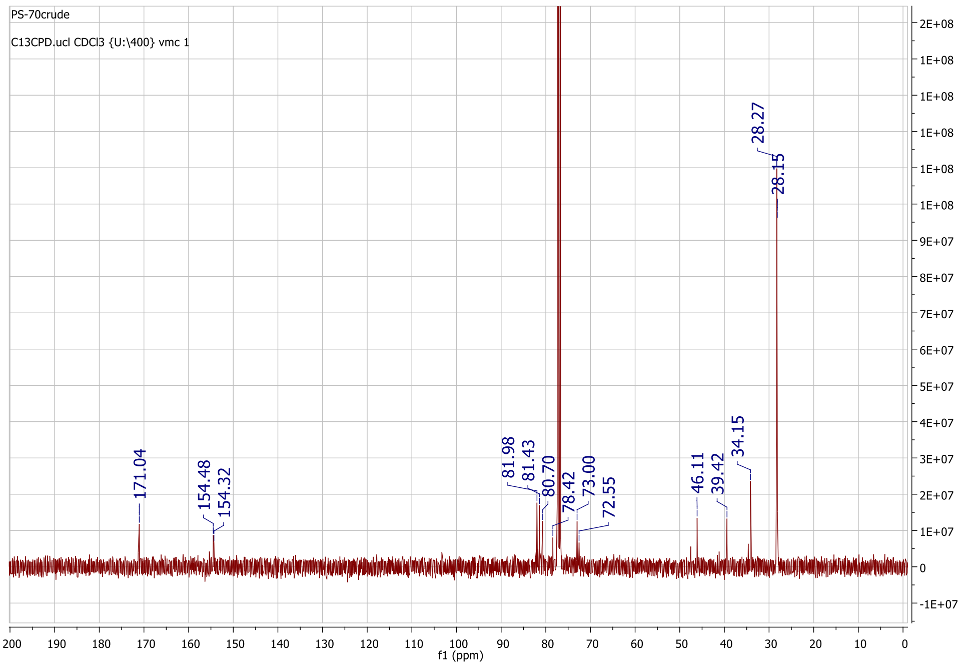

9.14. Compound **S12**, 3-(4,5-dibromo-3,6-dioxo-2-(prop-2-yn-1-yl)-3,6-dihydropyridazin-1(2*H*)-yl)propanoic acid[3]

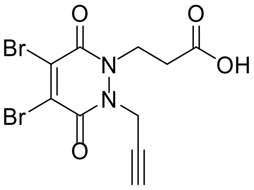

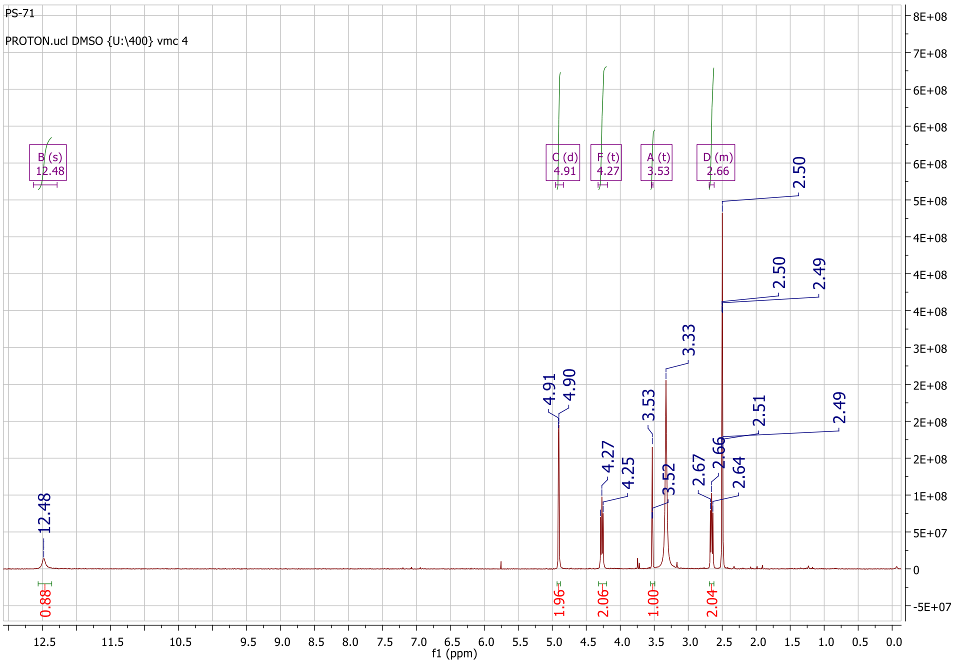

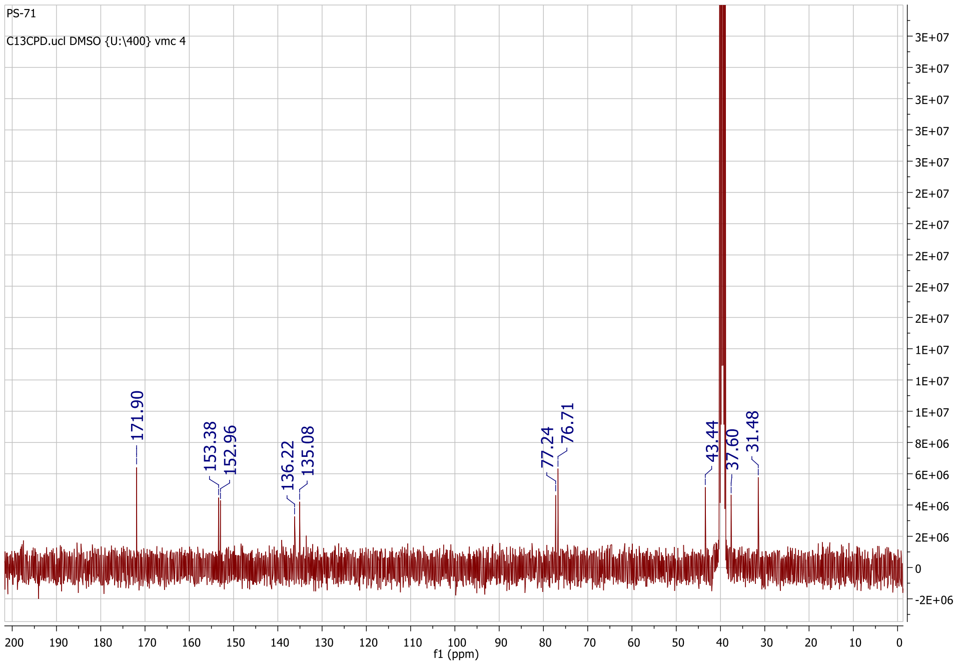

9.15. Compound **S13**, 3-(4,5-dibromo-2-((1-(1-(4-(6-methyl-1,2,4,5-tetrazin-3-yl)phenyl)-3,7-dioxo-11,14,17-trioxa-2,8-diazanonadecan-19-yl)-1*H*-1,2,3-triazol-4-yl)methyl)-3,6-dioxo-3,6-dihydropyridazin-1(2*H*)-yl)propanoic acid[3]

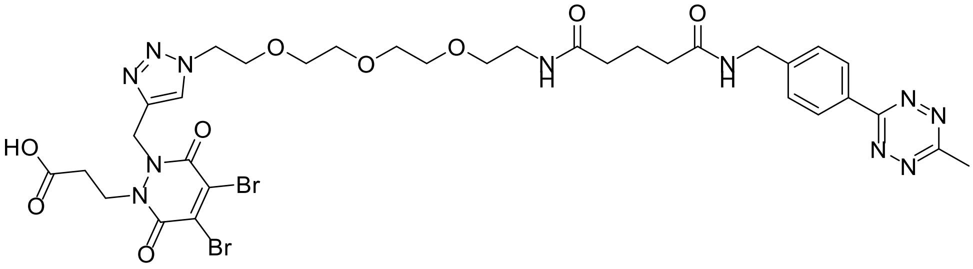

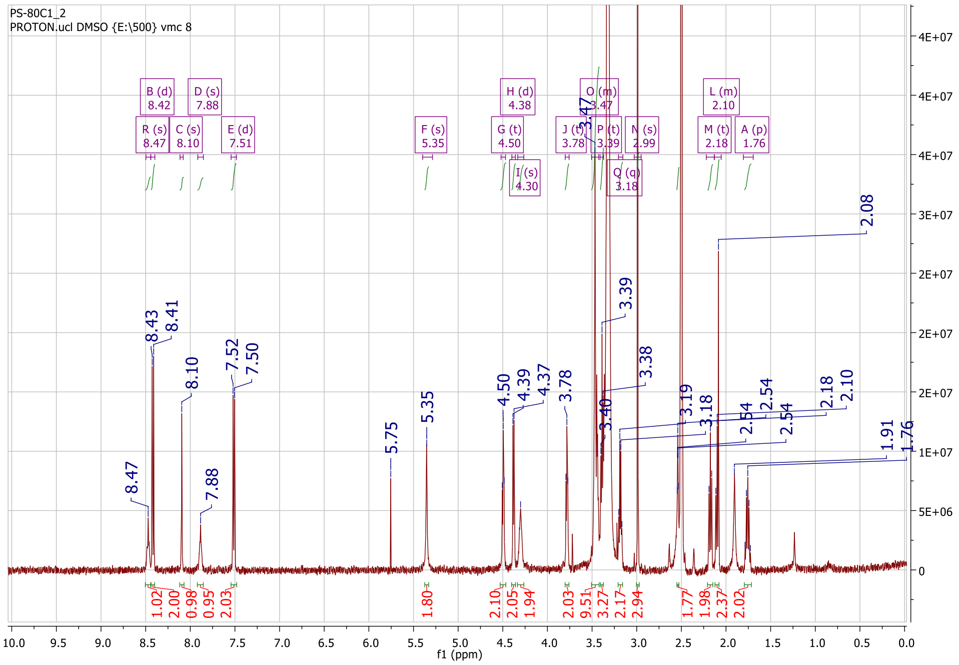

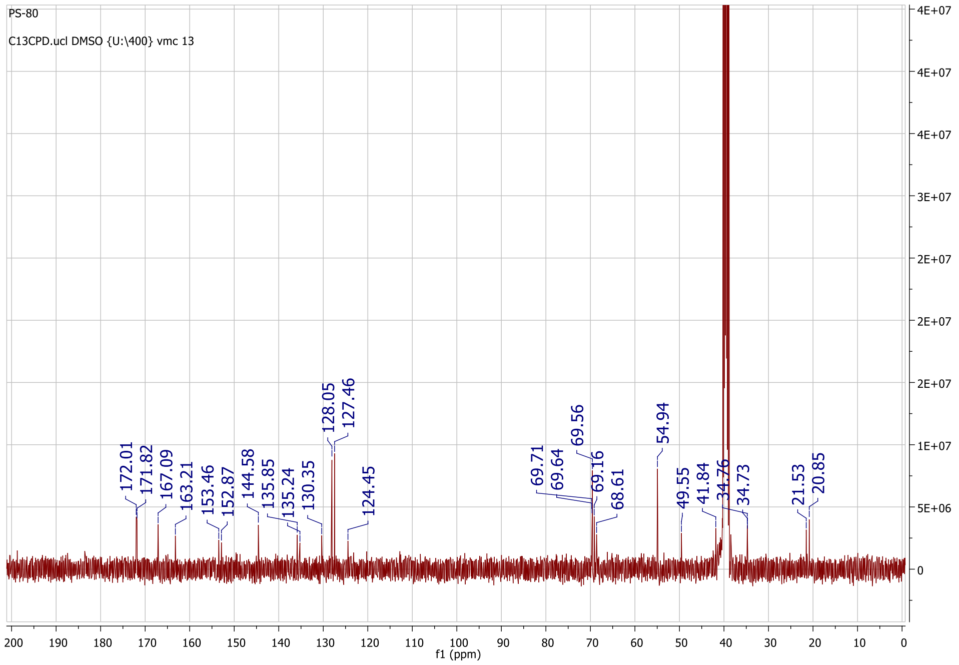

9.16. Compound **1**, N1-(2-(2-(2-(2-(4-((2-(1-azido-13-oxo-3,6,9-trioxa-12-azapentadecan-15-yl)-4,5-dibromo-3,6-dioxo-3,6-dihydropyridazin-1(2H)-yl)methyl)-1H-1,2,3-triazol-1-yl)ethoxy)ethoxy)ethoxy)ethyl)-N5-(4-(6-methyl-1,2,4,5-tetrazin-3-yl)benzyl)glutaramide[3]

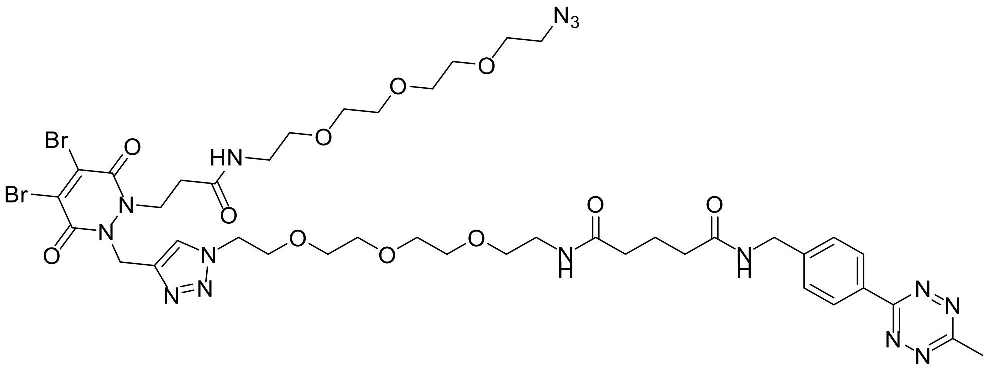

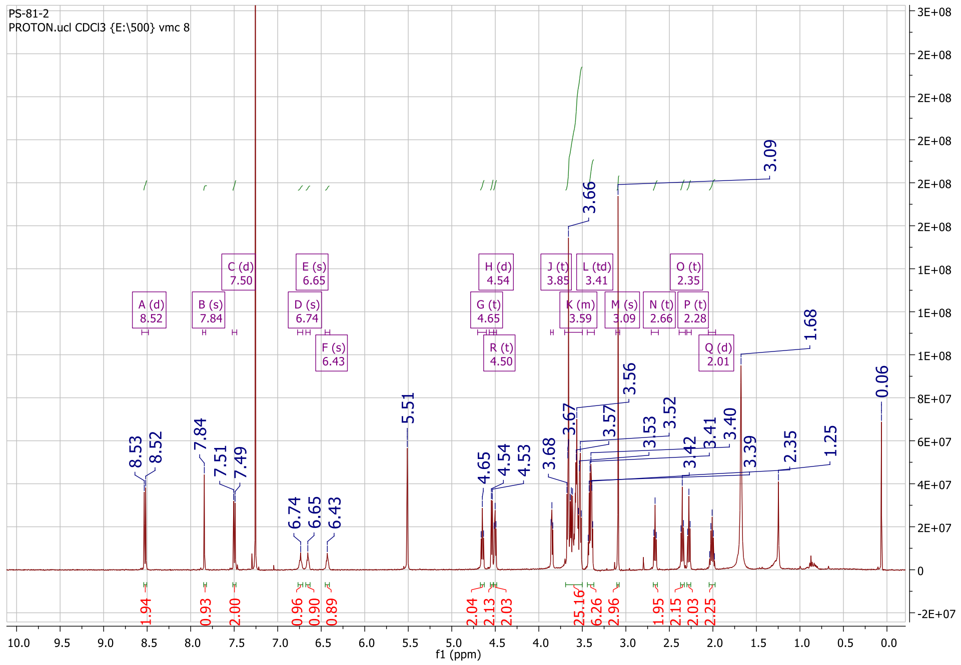

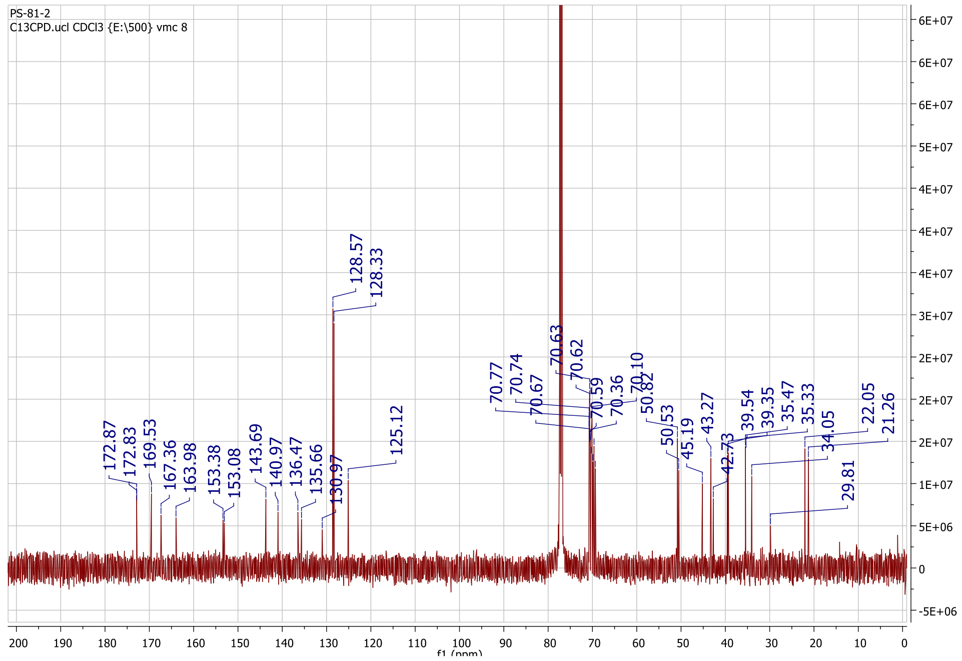

9.17. Compound **2**, bis(((1*R*,8*S*,9s)-bicyclo[6.1.0]non-4-yn-9-yl)methyl) (((oxybis(ethane-2,1-diyl))bis(oxy))bis(propane-3,1-diyl))dicarbamate

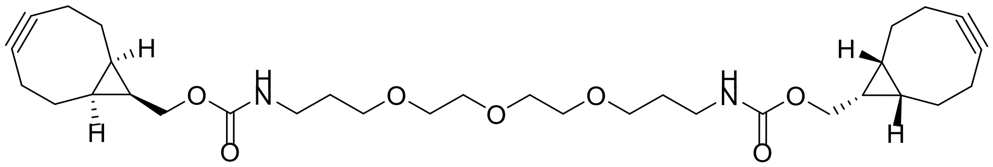

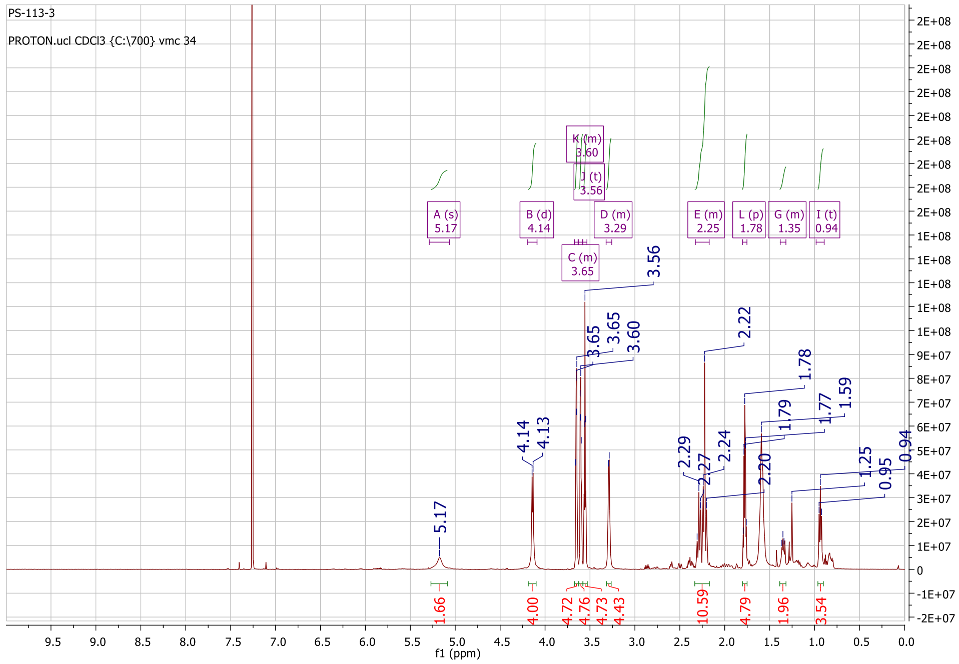

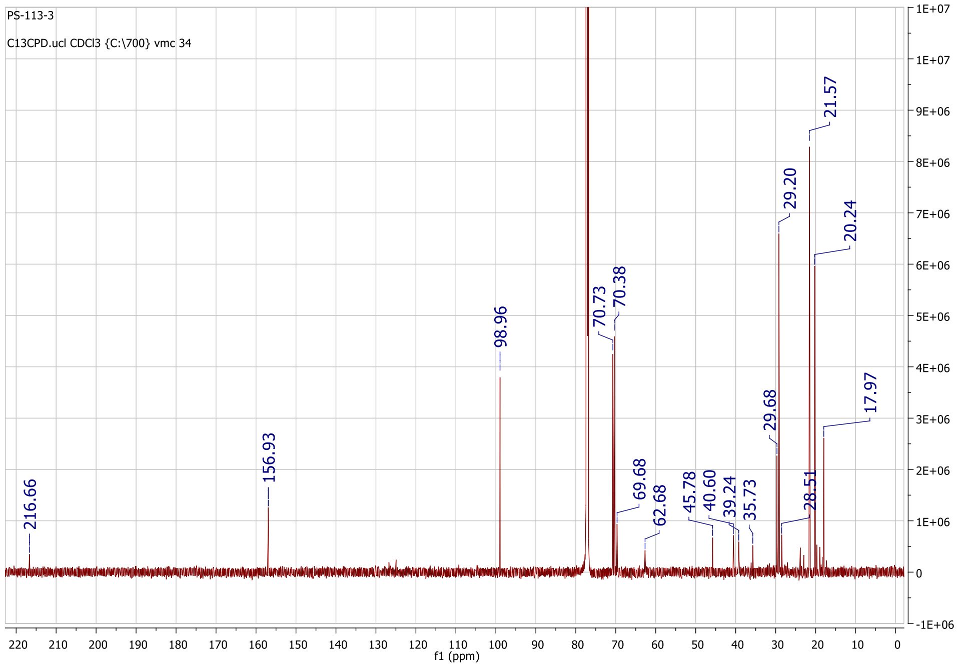

10. LC-MS spectra

N.B.: The region of the LC TIC trace from which the raw data was extracted is shown in the upper left corner of the raw data and deconvoluted spectra.

10.2. Fab$_{PD-1}$ **7**

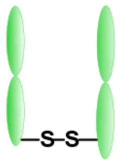

Observed mass: 47884.3 Da, 48023.4 Da.
Mass difference between peak 1 and 2: 139.1 Da (histidine residue (137.1 Da)?).

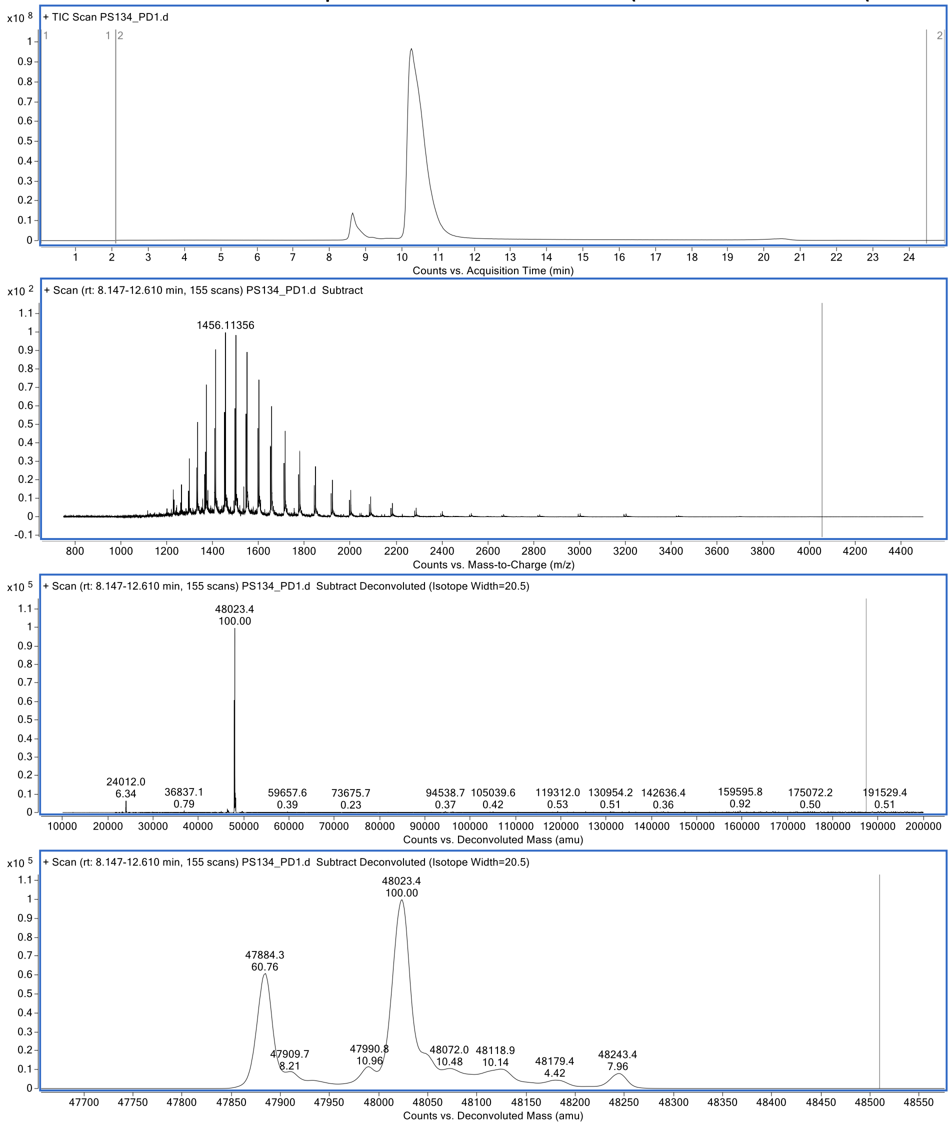

10.3. Sia-Tet-N_3 **8**

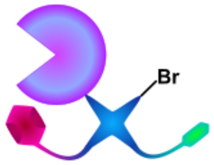

Expected mass: 47011.9 Da.
Observed mass: 47012.4 Da.
1118.26 Da -> Br_2PD-Tet-N_3 1 + Na^+

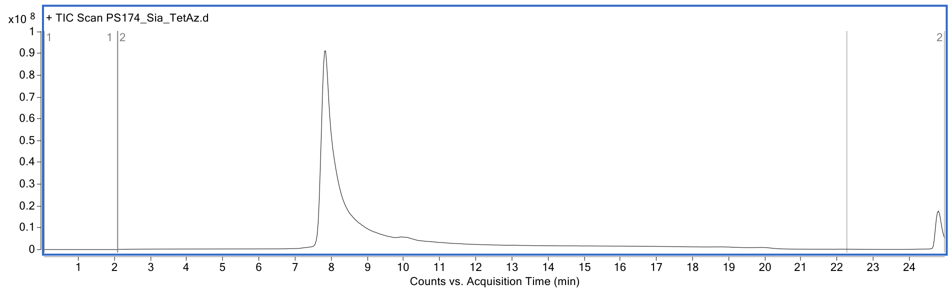

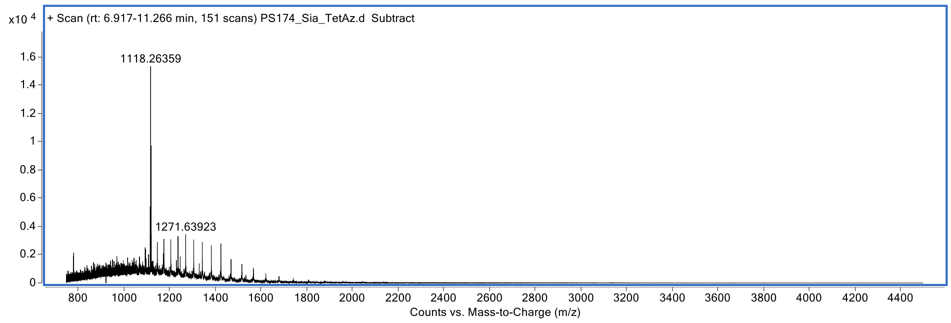

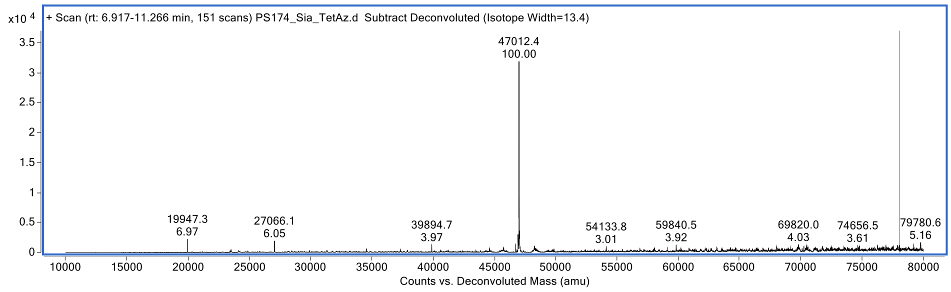

10.4. Fab$_{PD-1}$–Tet-N$_3$ **9**

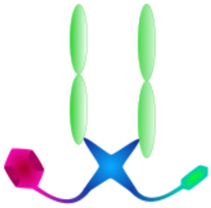

Expected mass: 48819.8 Da, 48958.9 Da.

Observed mass: 48818.0 Da, 48957.3 Da.

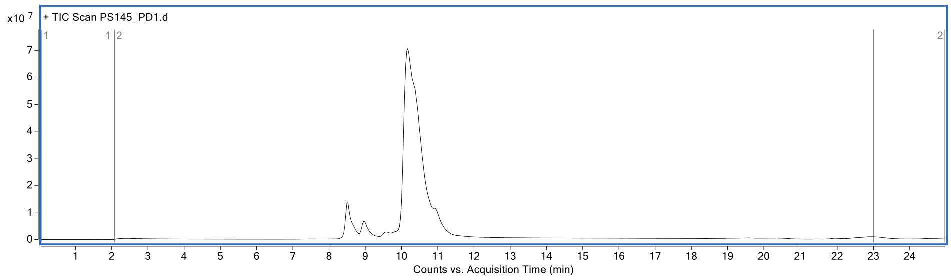

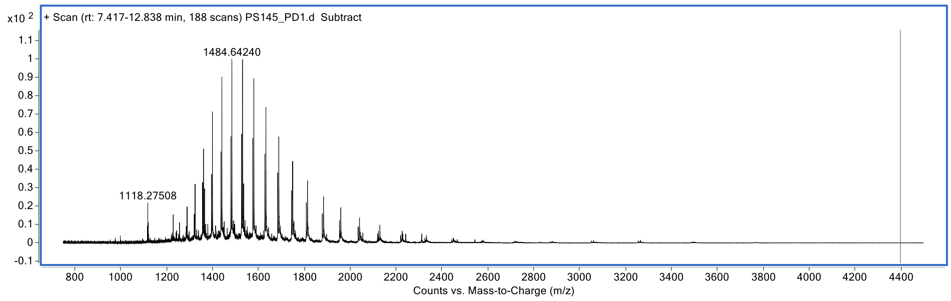

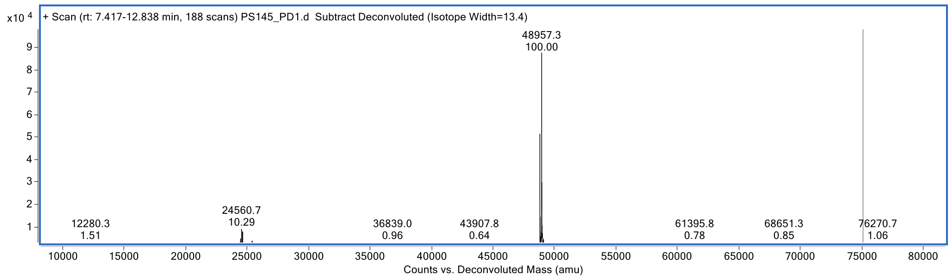

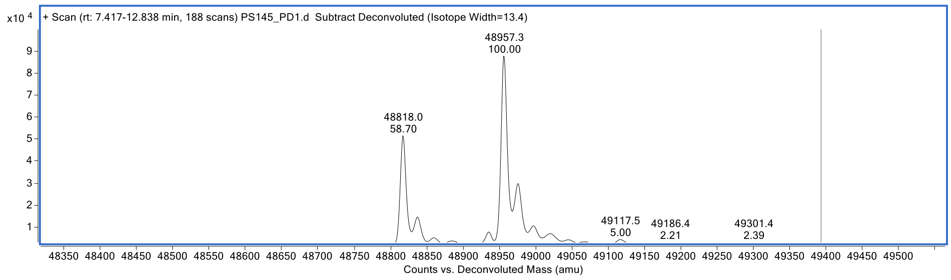

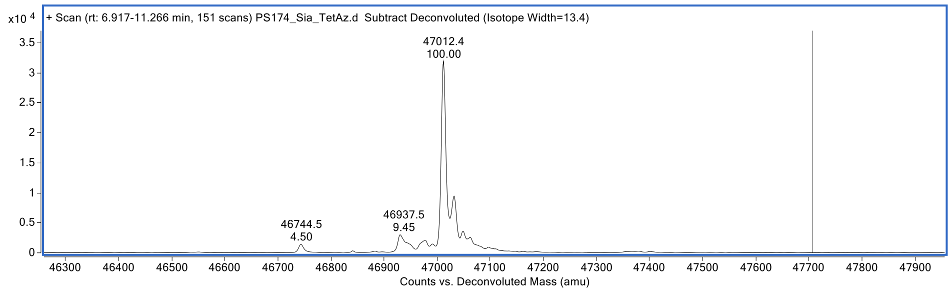

10.5. Fab$_{HER2}$-BCN **10**

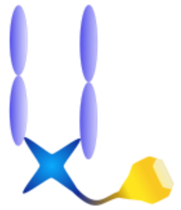

Expected mass: 48141.2 Da. Observed mass: 48140.8 Da.

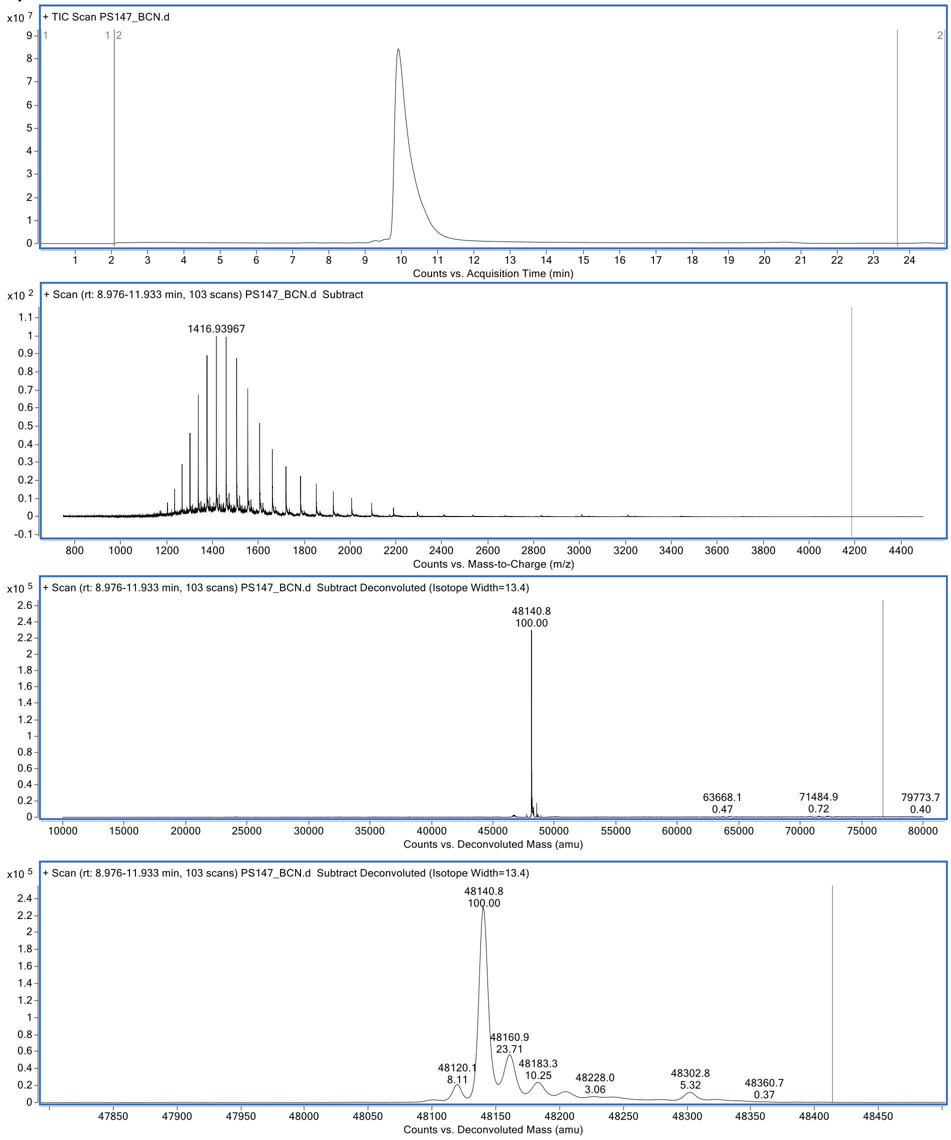

10.6. Fab_{HER2}-Sia-Biotin **11**

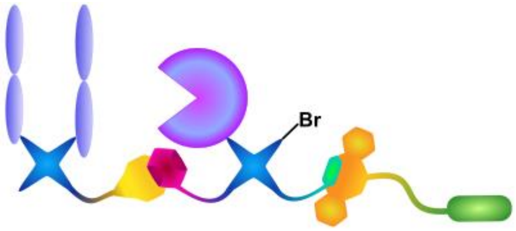

Expected mass: 95873 Da. Observed: 95845 and 95891 Da (HCO_2H adduct).

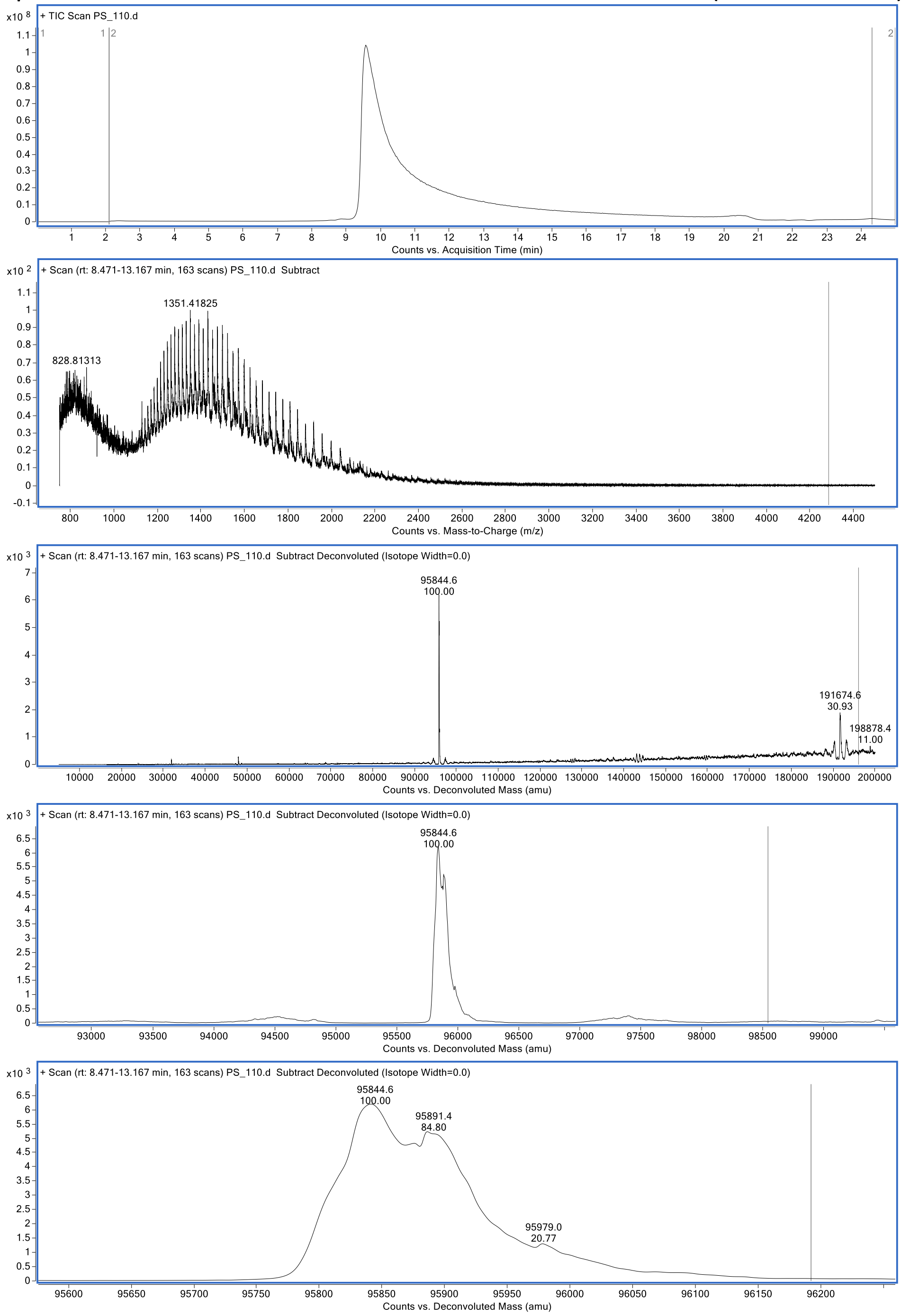

10.7. Fab_{HER2}-Fab_{CD3}-Biotin **12**

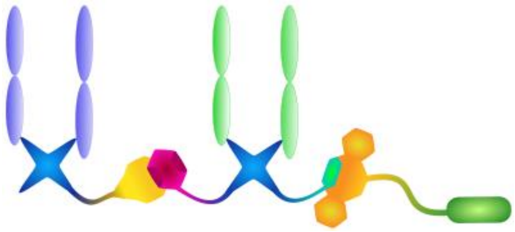

Expected mass: 97243 Da. Observed mass: 97257 Da. (Hump on right derives from Fab_{CD3}.)

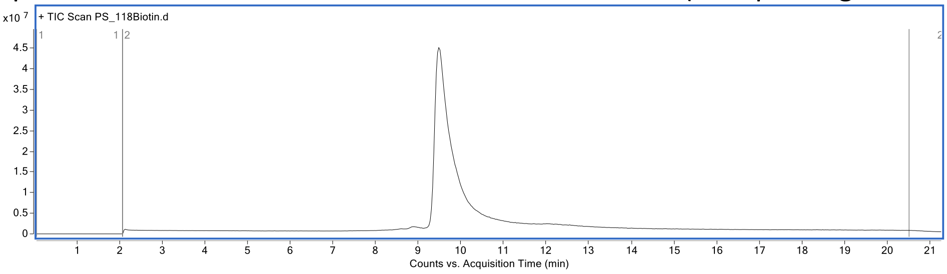

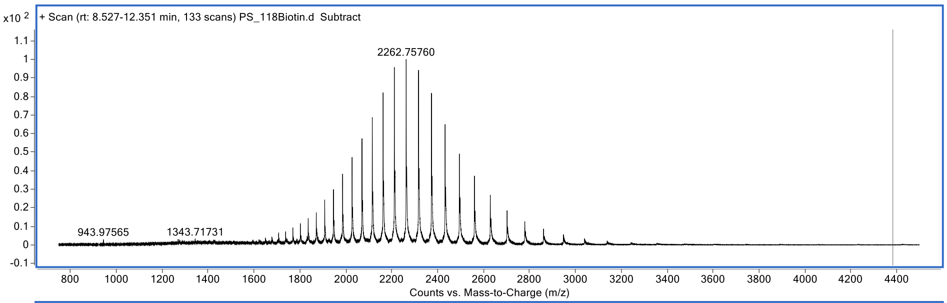

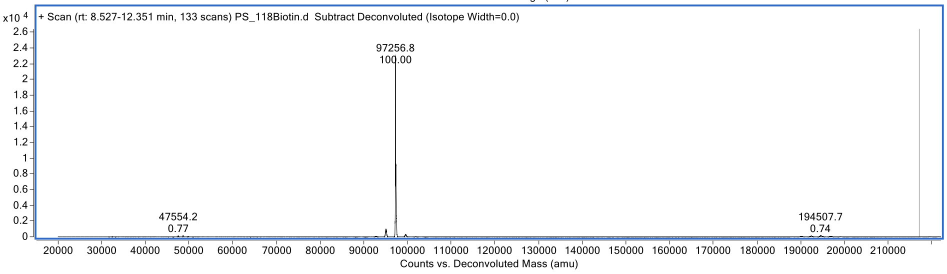

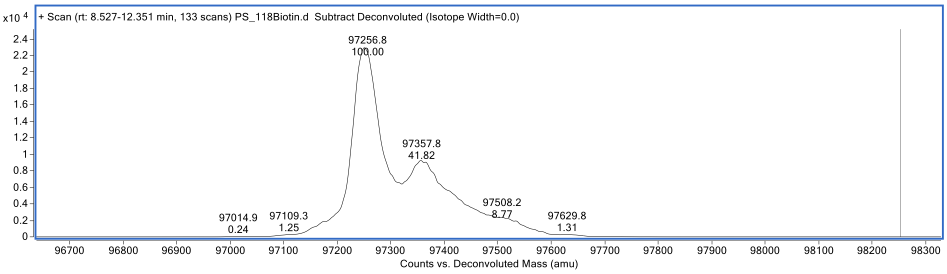

10.8. Fab_{HER2}-Fab_{CD3}-N_3 **13**

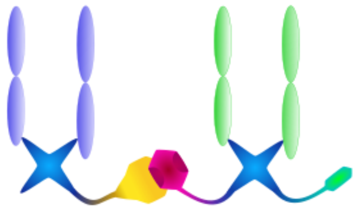

Expected mass: 96493 Da. Observed mass: 96506 Da. (Hump on right derives from Fab_{CD3}.)

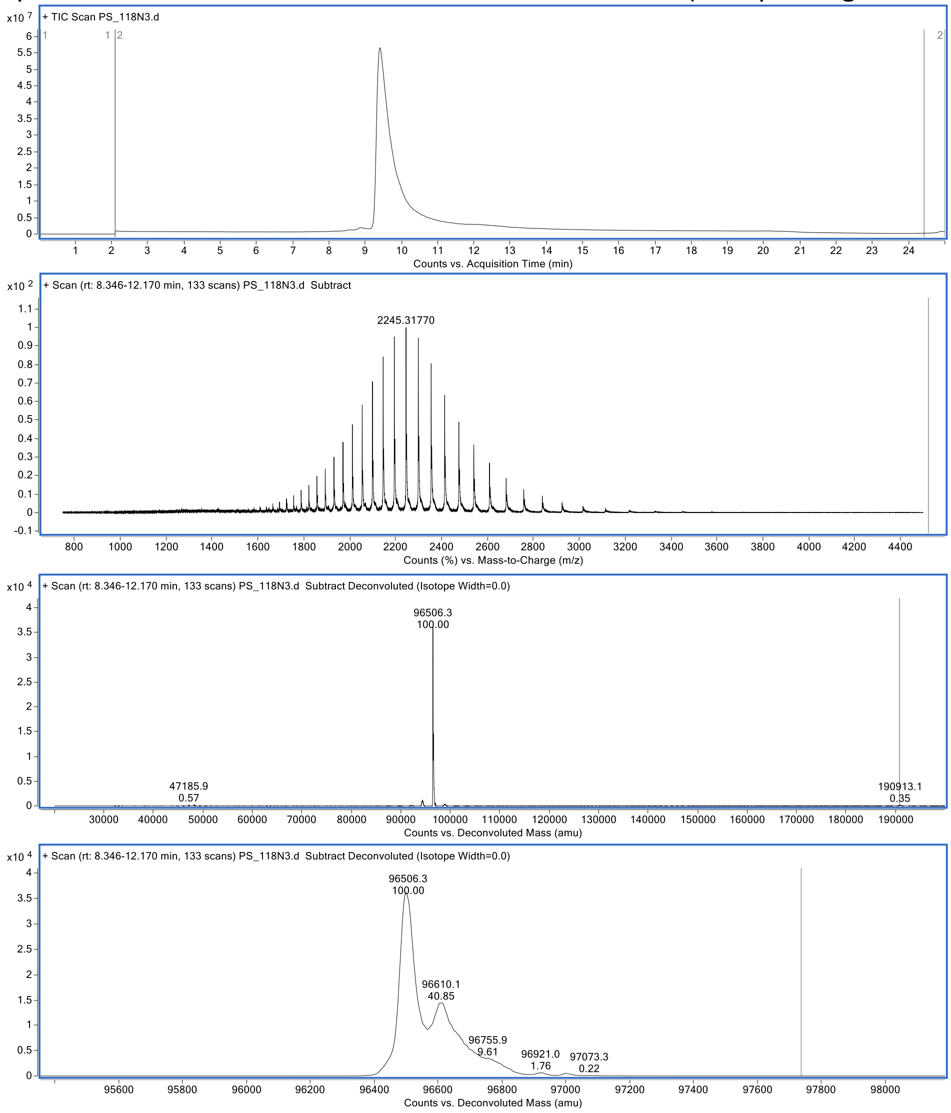

10.9. Fab$_{HER2}$-Tet-N_3 **15**

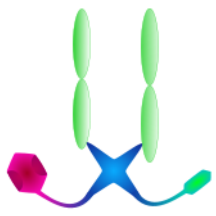

Expected mass: 48574.1 Da.

Observed mass: 48574.1 Da, 48814.0 Da (+240 Da).

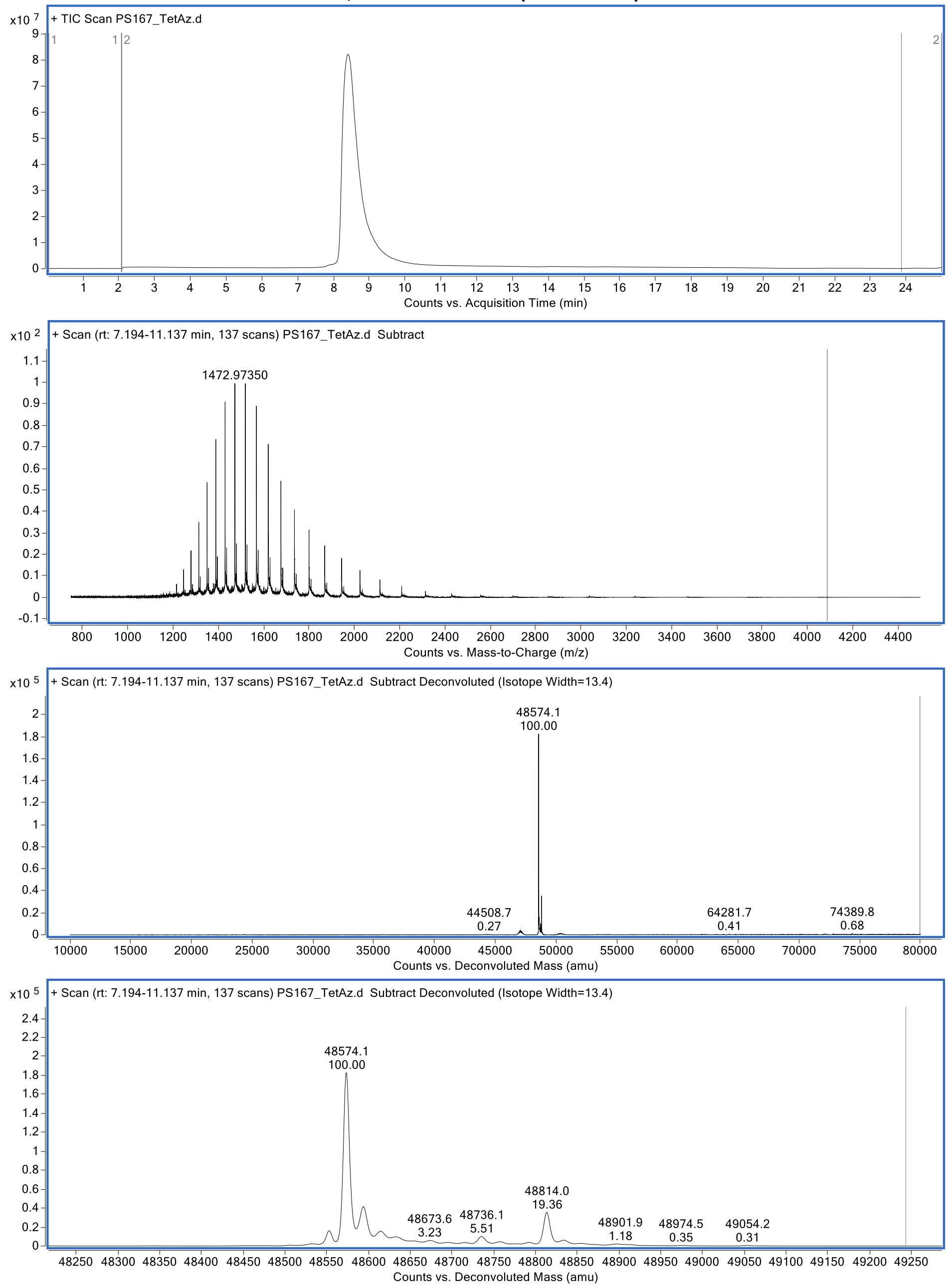

10.10. Fab_{HER2}-(Biotin)-Fab_{CD20}-Biotin **17**

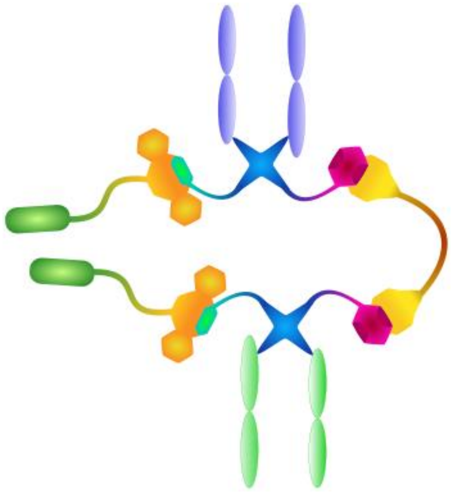

Expected mass: 98734.5 Da. Observed mass: 98722 Da.

Expected for (Fab_{HER2}-biotin)$_2$: 99193 Da, observed: 99181 Da.

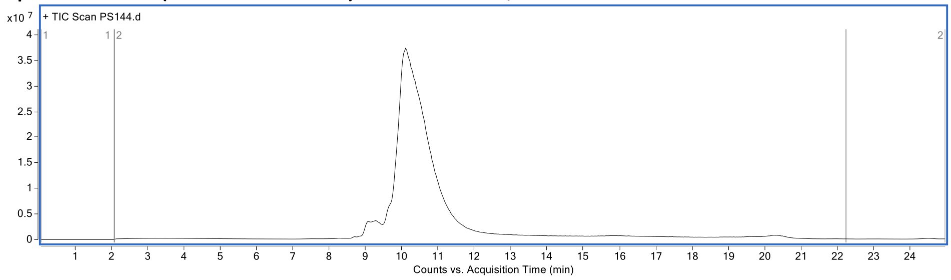

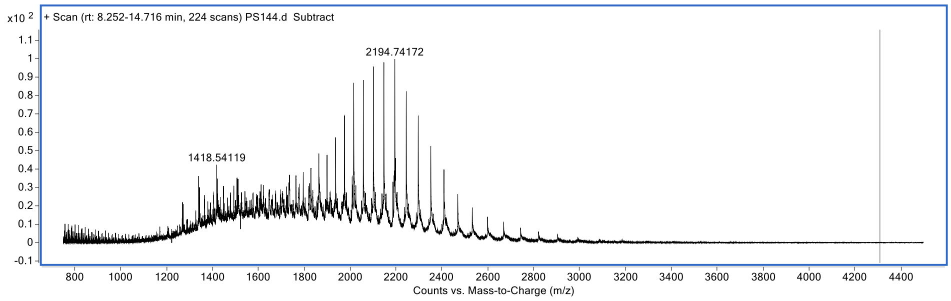

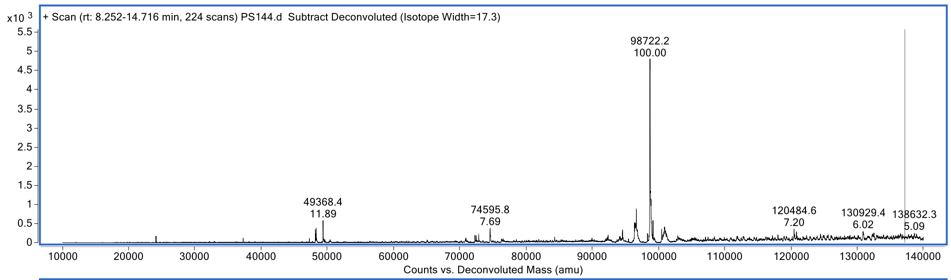

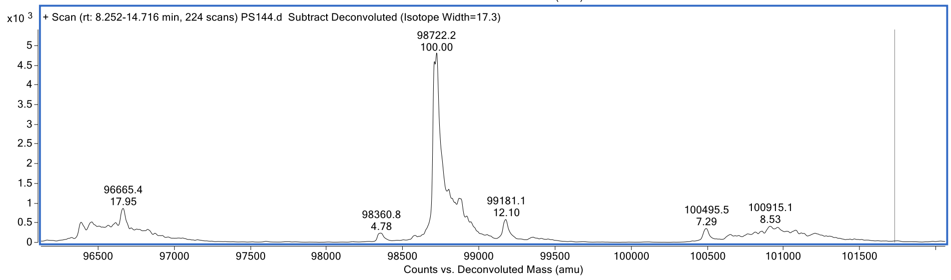

10.11. Fab$_{HER2}$-BCN-Biotin **18**

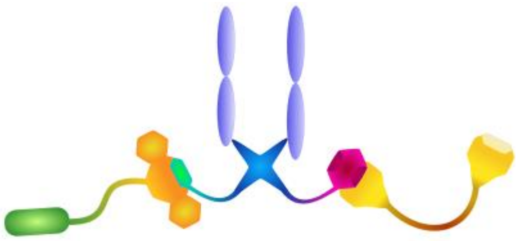

Expected mass: 49869 Da. Expected mass for oxidized version: 49901 Da (+32 Da).
Observed mass: 49886 Da. Difference to expected: 17 Da.

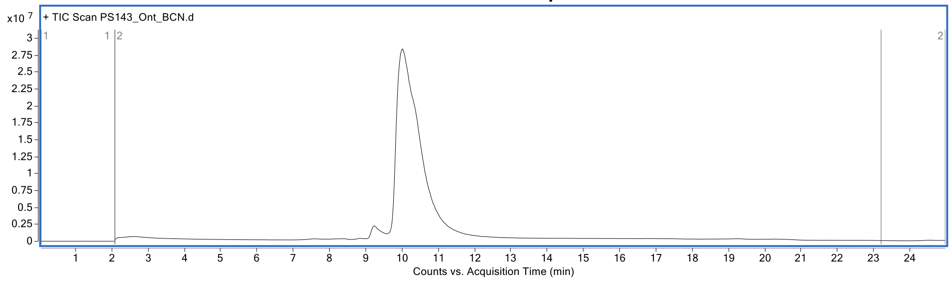

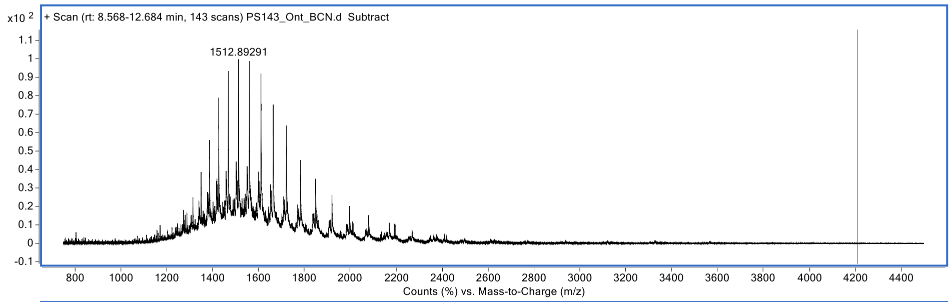

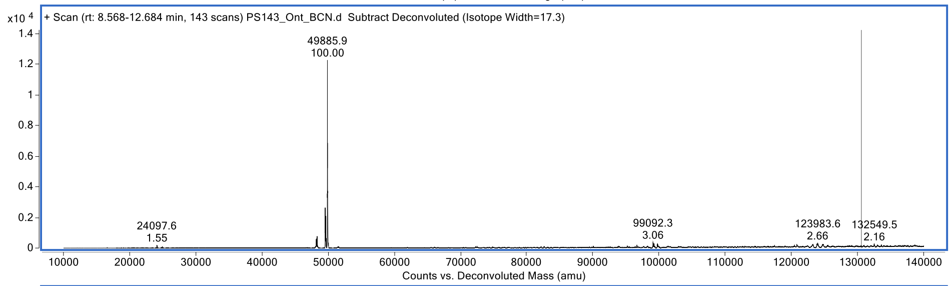

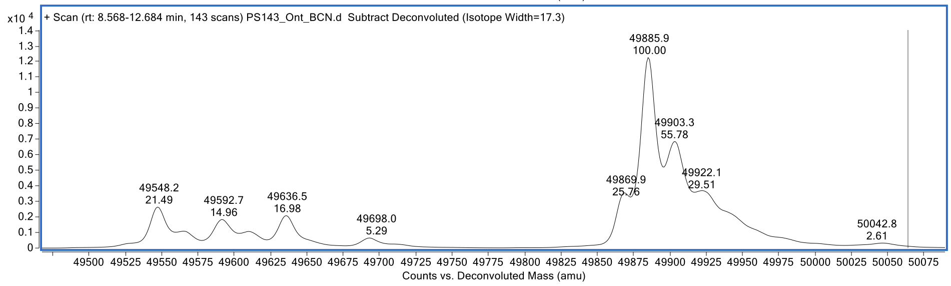

10.12. Fab_{CD20}-Tet-N_3 **19**

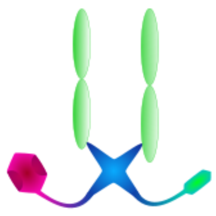

Expected mass: 48114 Da.
Observed mass: 48117 Da.

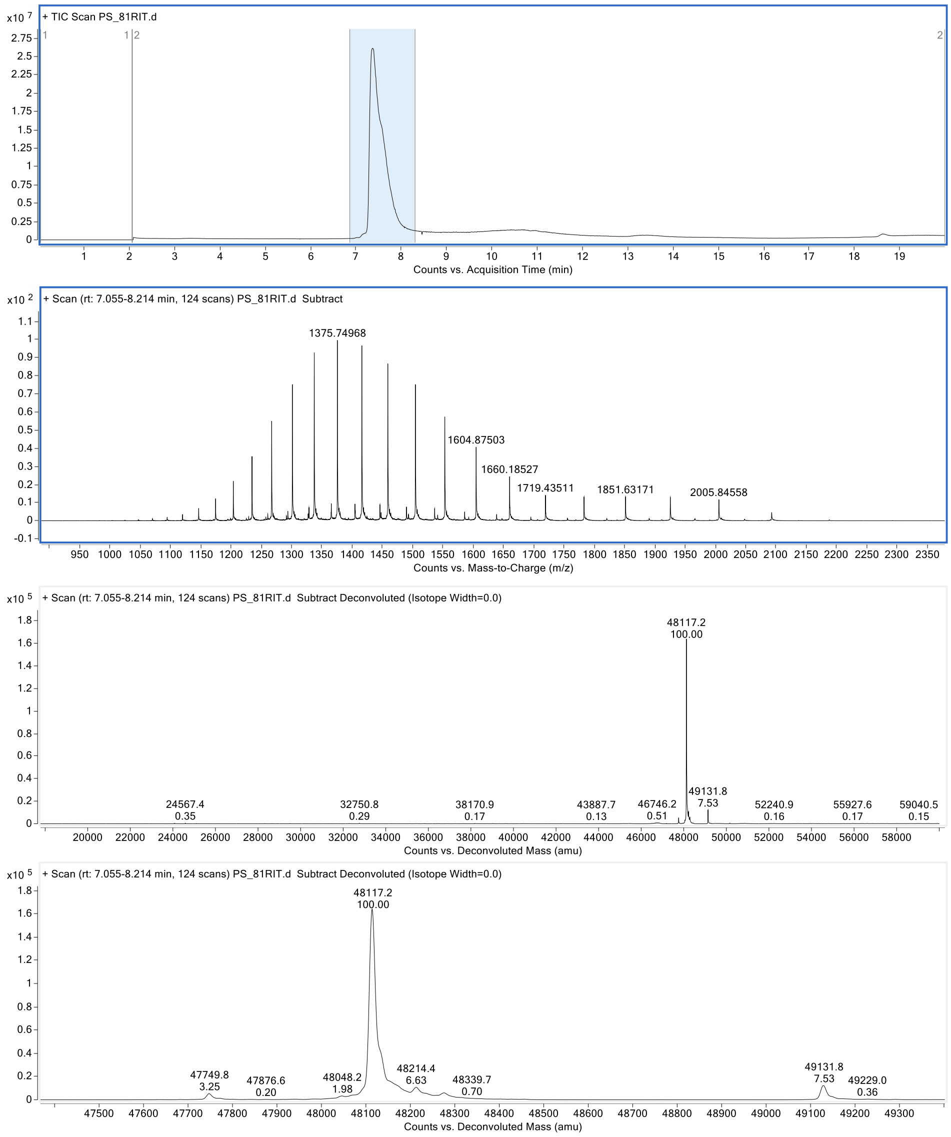

10.13. Fab_{HER2}-Fab_{CD20}-Sia-Biotin **21**

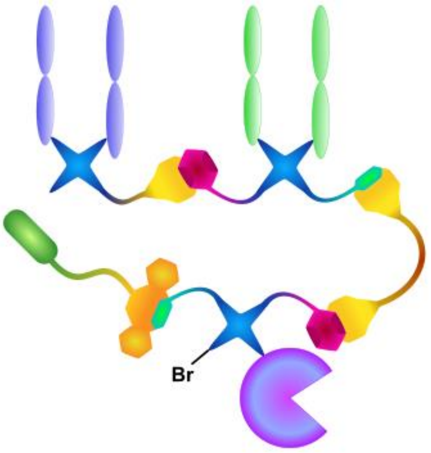

Expected mass: 144532 Da. Observed: 144553 Da.

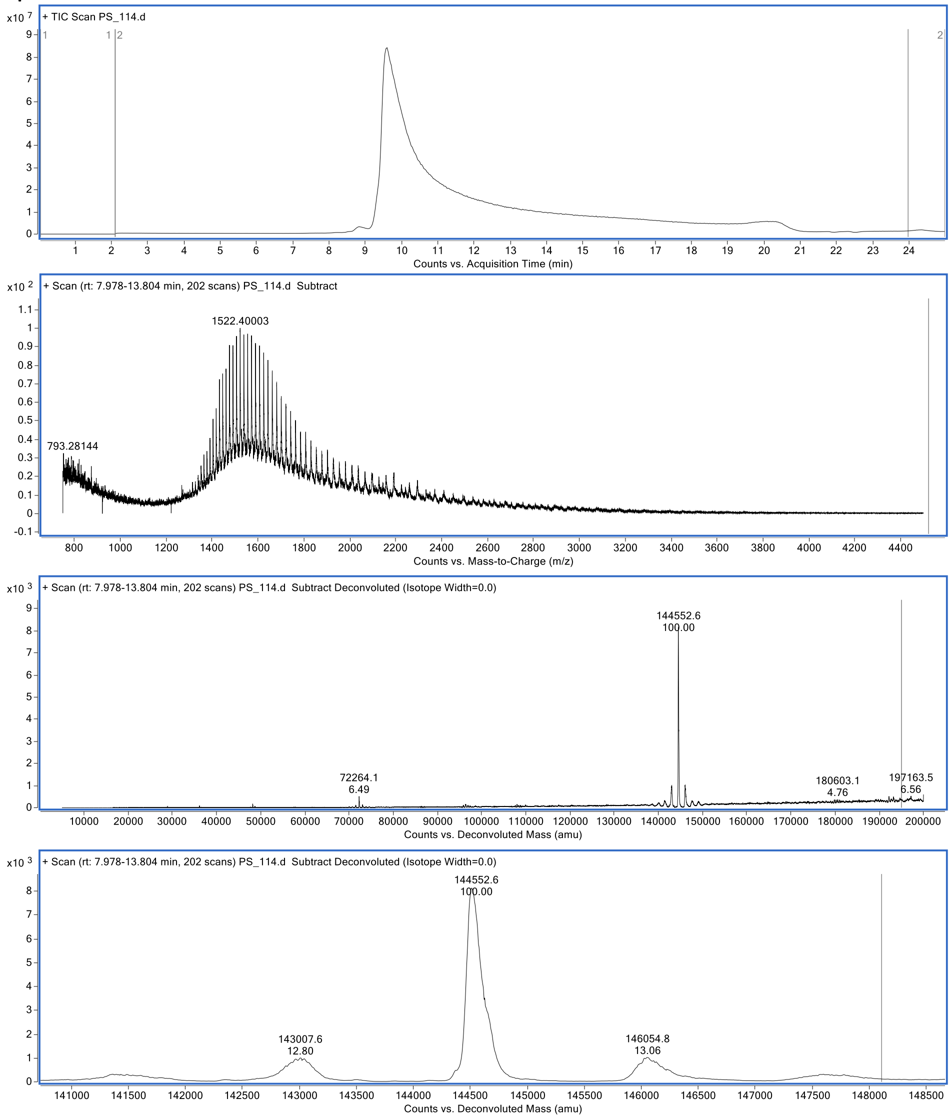

10.14. Fab_{HER2}-Fab_{CD20}-N_3 **22**

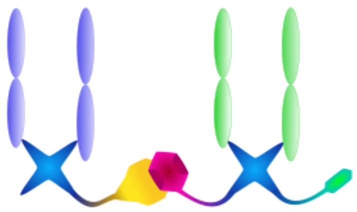

Expected mass for Fab_{HER2}-Fab_{CD20}-N_3: 96226 Da. Observed: 96234 Da.

Expected mass for: N_3-Fab_{CD20}-tet: 48114 Da. Observed: 48117 Da.

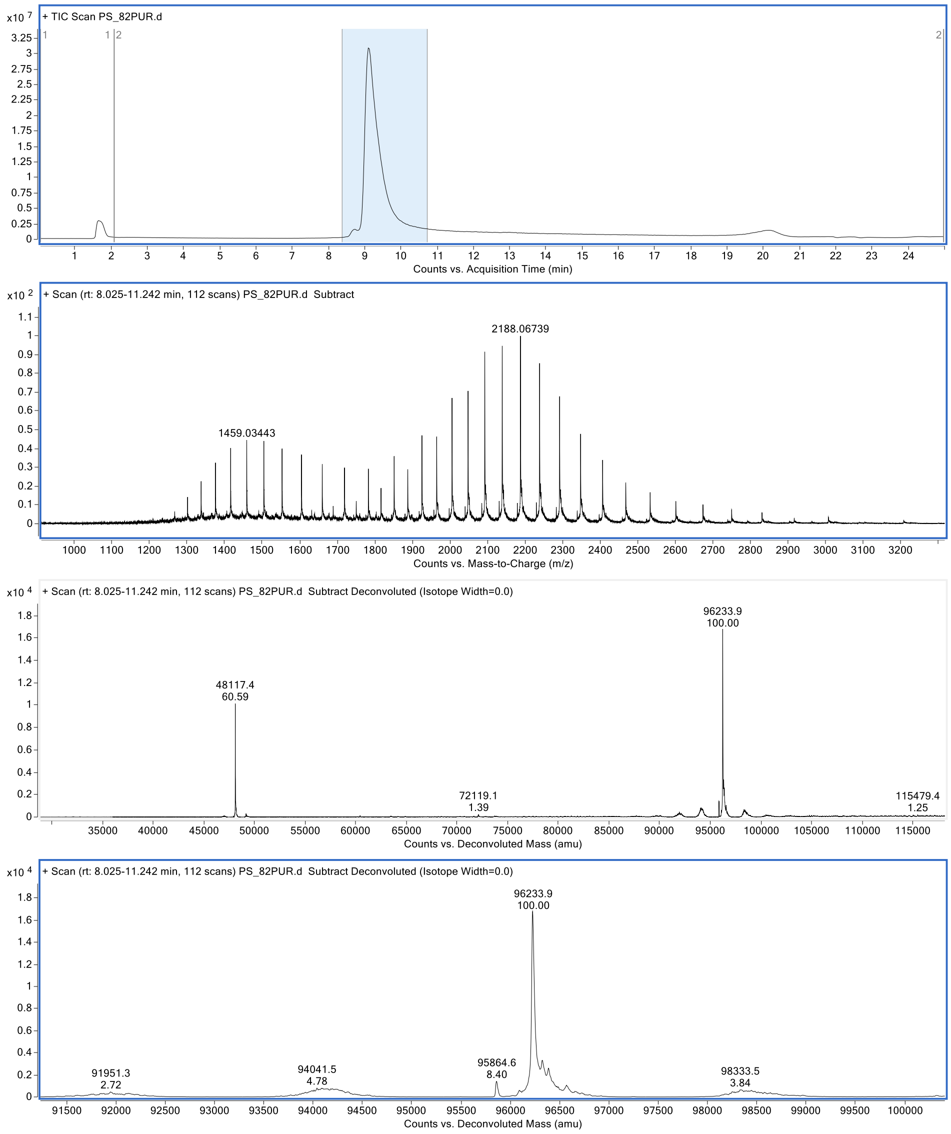

10.15. Fab_{HER2}-Fab_{CD3}-Sia-Biotin **24** - impure

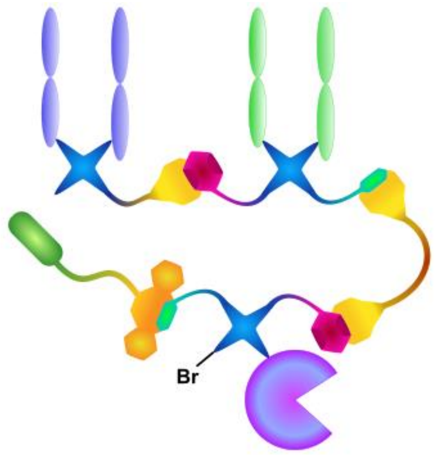

Expected mass: 144799 Da. Observed: 144791 Da.

Expected mass for Fab_{HER2}-Fab_{CD3}-Fab_{HER2}: 144632 Da. Observed: 144644 Da.

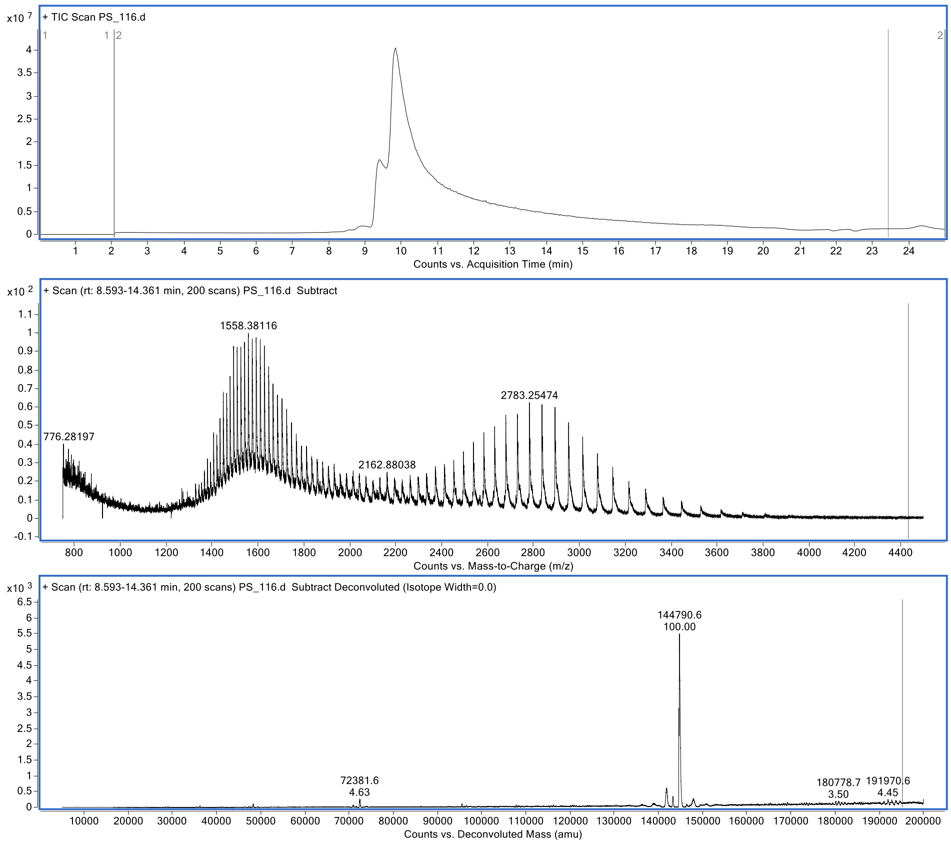

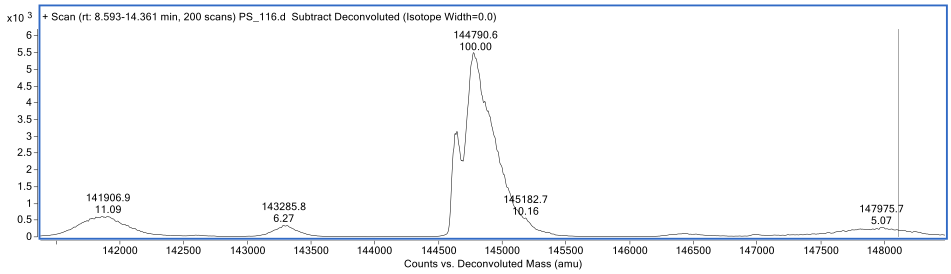
x10 3
+ Scan (rt: 8.593-14.361 min, 200 scans) PS_116.d Subtract Deconvoluted (Isotope Width=0.0)
144790.6
100.00
141906.9
11.09
143285.8
6.27
145182.7
10.16
147975.7
5.07
Counts vs. Deconvoluted Mass (amu)

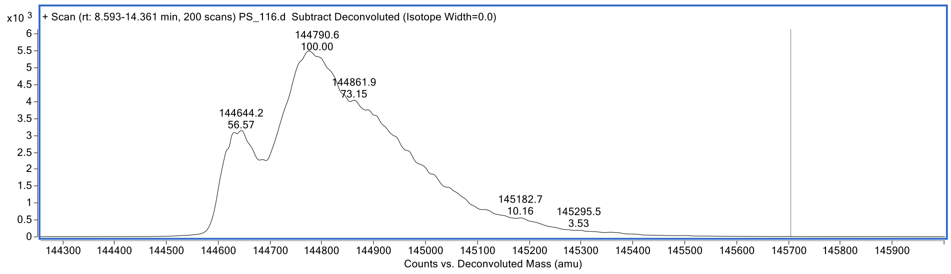
x10 3
+ Scan (rt: 8.593-14.361 min, 200 scans) PS_116.d Subtract Deconvoluted (Isotope Width=0.0)
144790.6
100.00
144861.9
73.15
144644.2
56.57
145182.7
10.16
145295.5
3.53
Counts vs. Deconvoluted Mass (amu)

10.16. Fab_{HER2}-Fab_{CD3}-Sia-Biotin **24** - pure

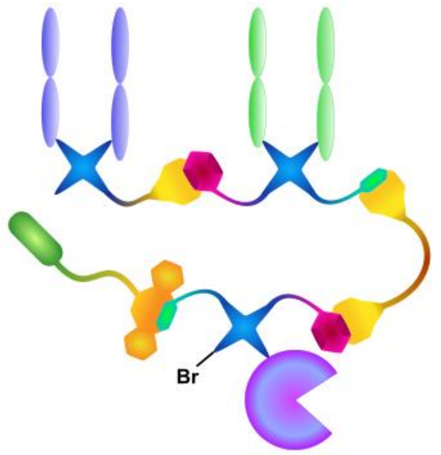

Expected mass: 144799 Da. Observed mass: 144825 Da. (Hump on right derives from Fab_{CD3} **S18**.)

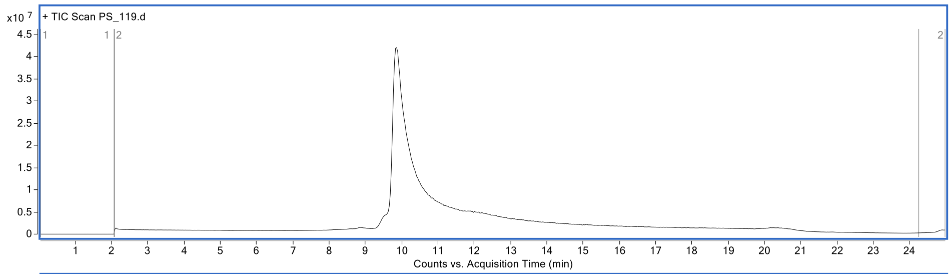

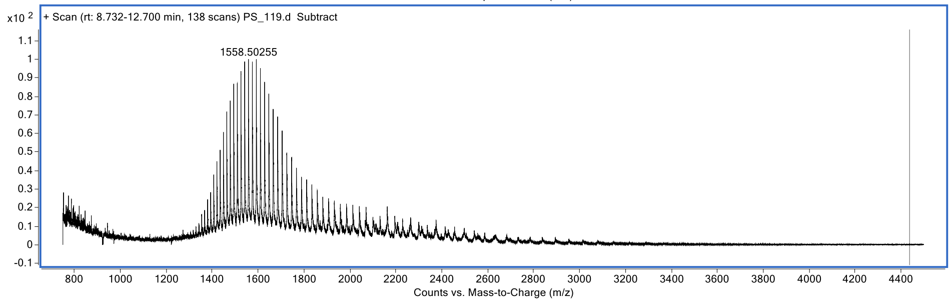

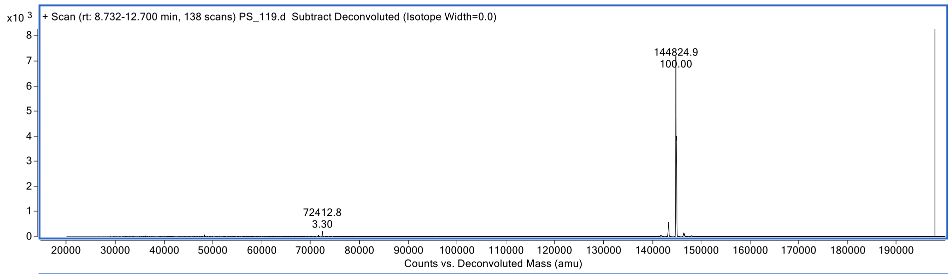

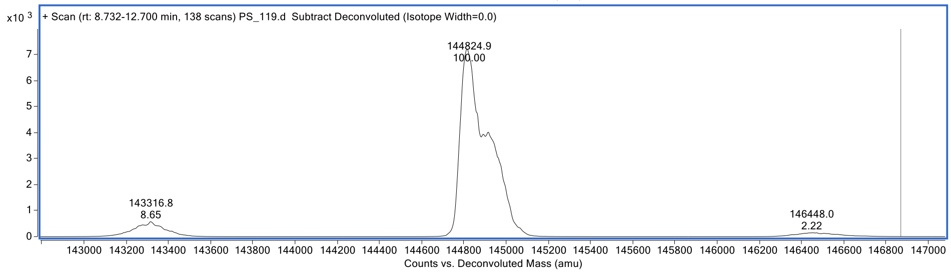

10.17. Fab_{HER2}-Fab_{CD3}-BCN **26**

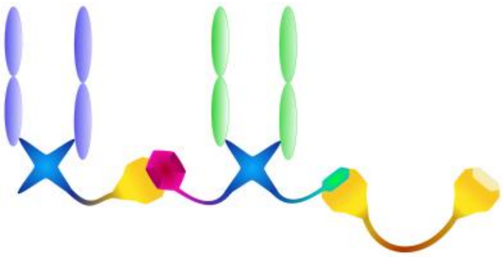

Expected mass: 97065 Da. Observed mass: 97081 Da. (Hump on right derives from Fab_{CD3}.)

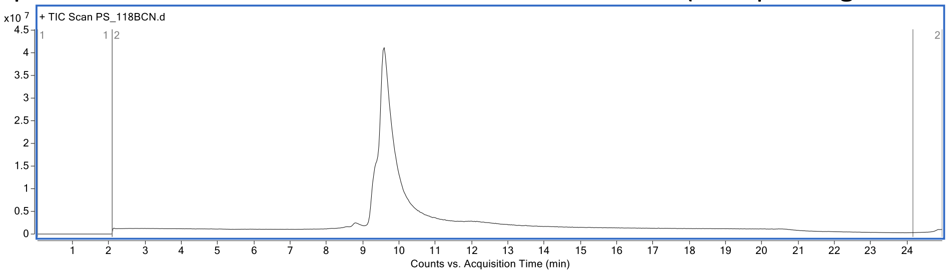

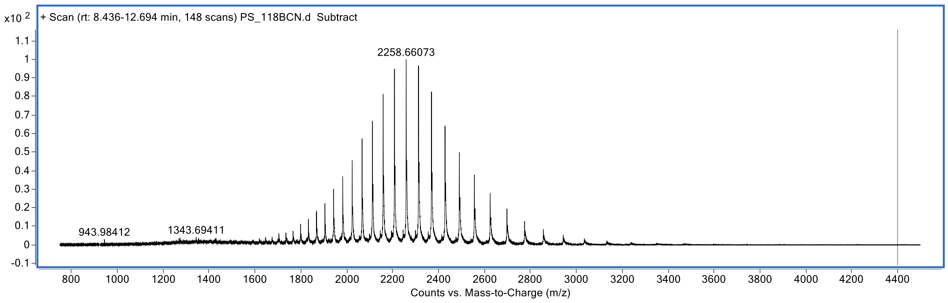

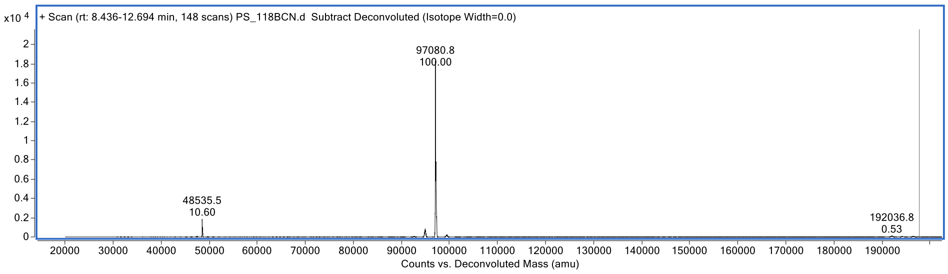

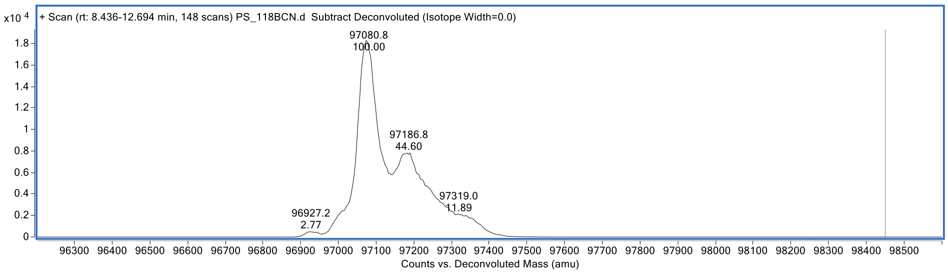

10.18. Fab$_{CD3}$-Fab$_{HER2}$-Fab$_{PD\text{-}1}$-biotin **27**

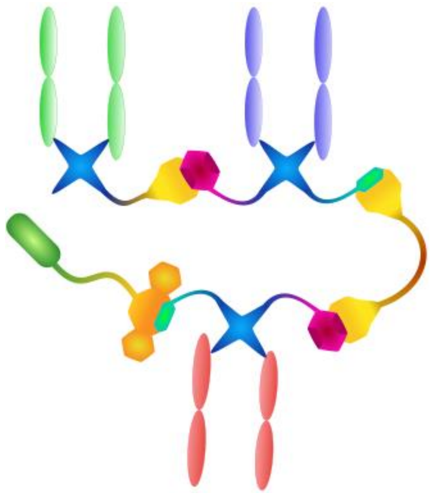

Expected mass: 146610.0 Da, 146749.1 Da, 146857.6 Da. Observed mass: 146618.0 Da, 146758.2 Da, 146861.9 Da.

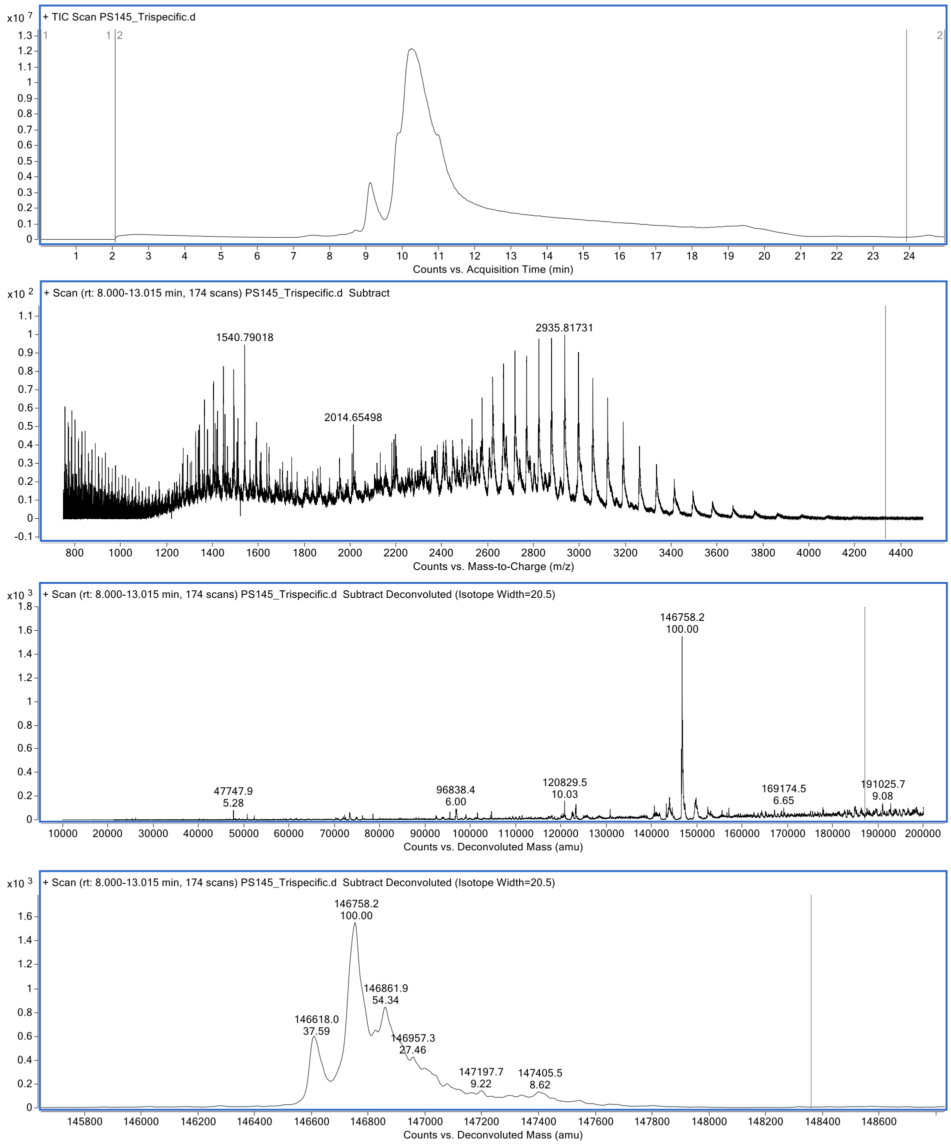

10.19. Fab_{CD3}-Fab_{HER2}–N_3 **28**

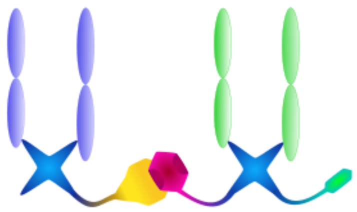

Expected mass: 96495.6 Da. Observed mass: 96505.1 Da.

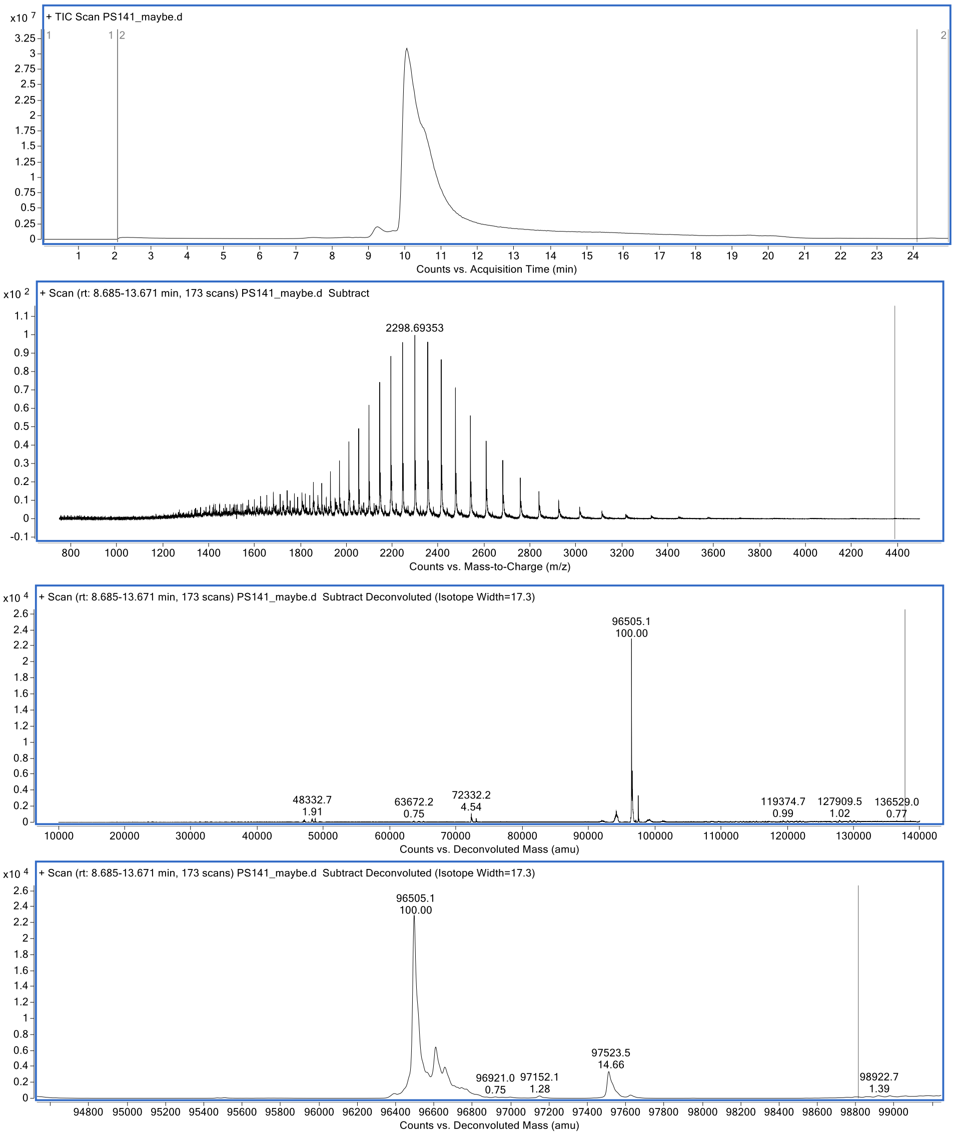

10.20. Fab$_{CD3}$-Fab$_{HER2}$–BCN **29**

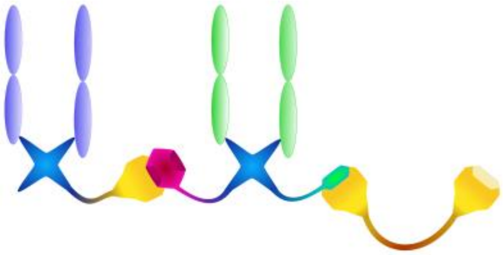

Expected mass: 97068.4 Da. Observed mass: 97086.2 and 97190.2 Da

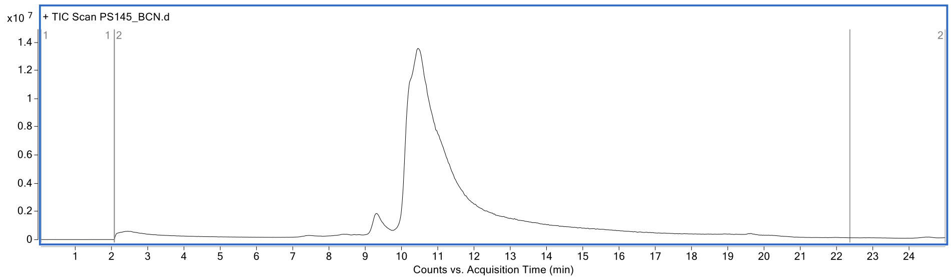

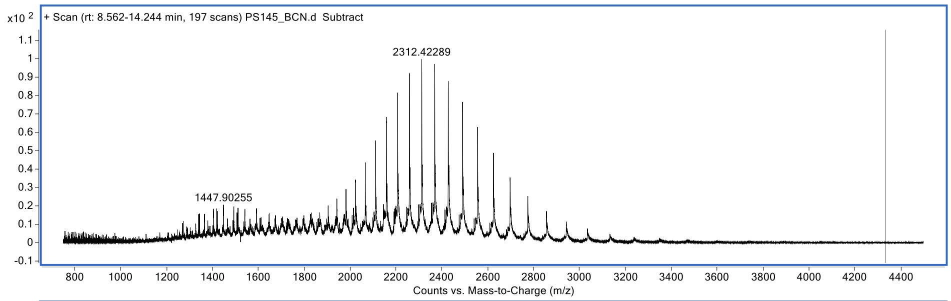

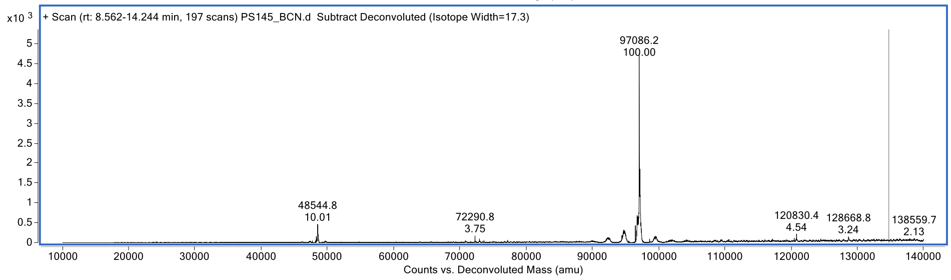

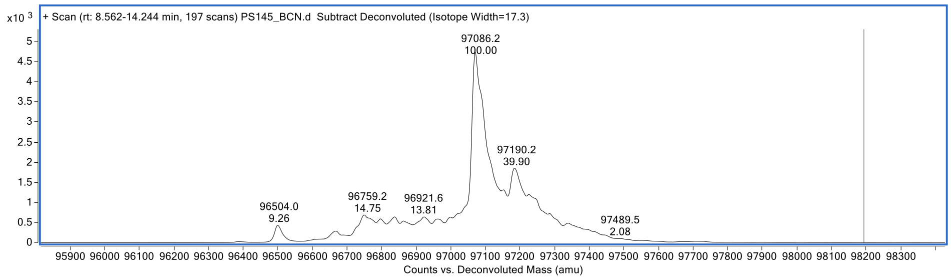

10.21. Fab_{HER2}-Fab_{CD3} **30**

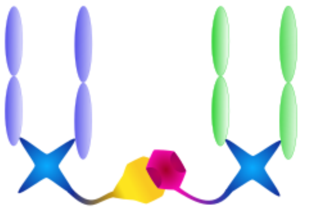

Expected mass: 96256.0 Da. Observed mass: 96261.0 Da.

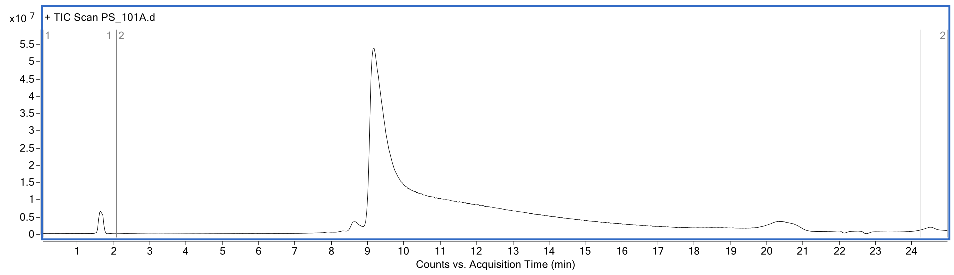

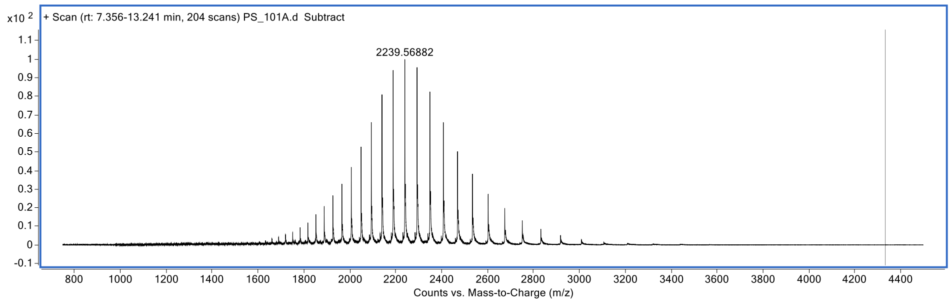

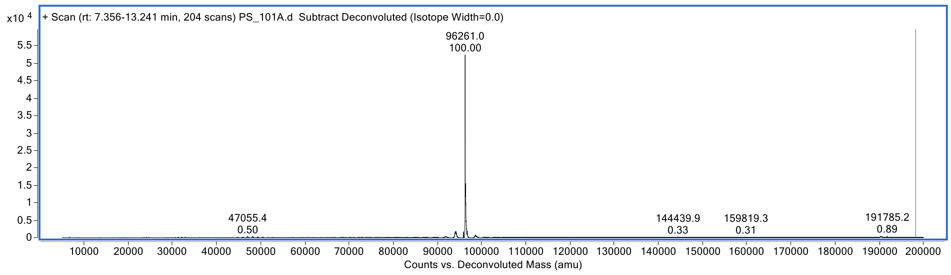

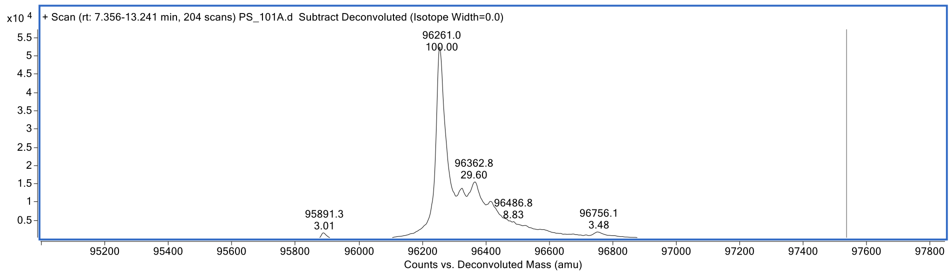

10.22. Fab_{EGFR} **S17**

Expected mass: 49788 and 49933 Da. Observed mass: 49787 and 49932 Da.

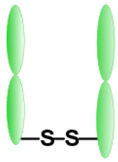

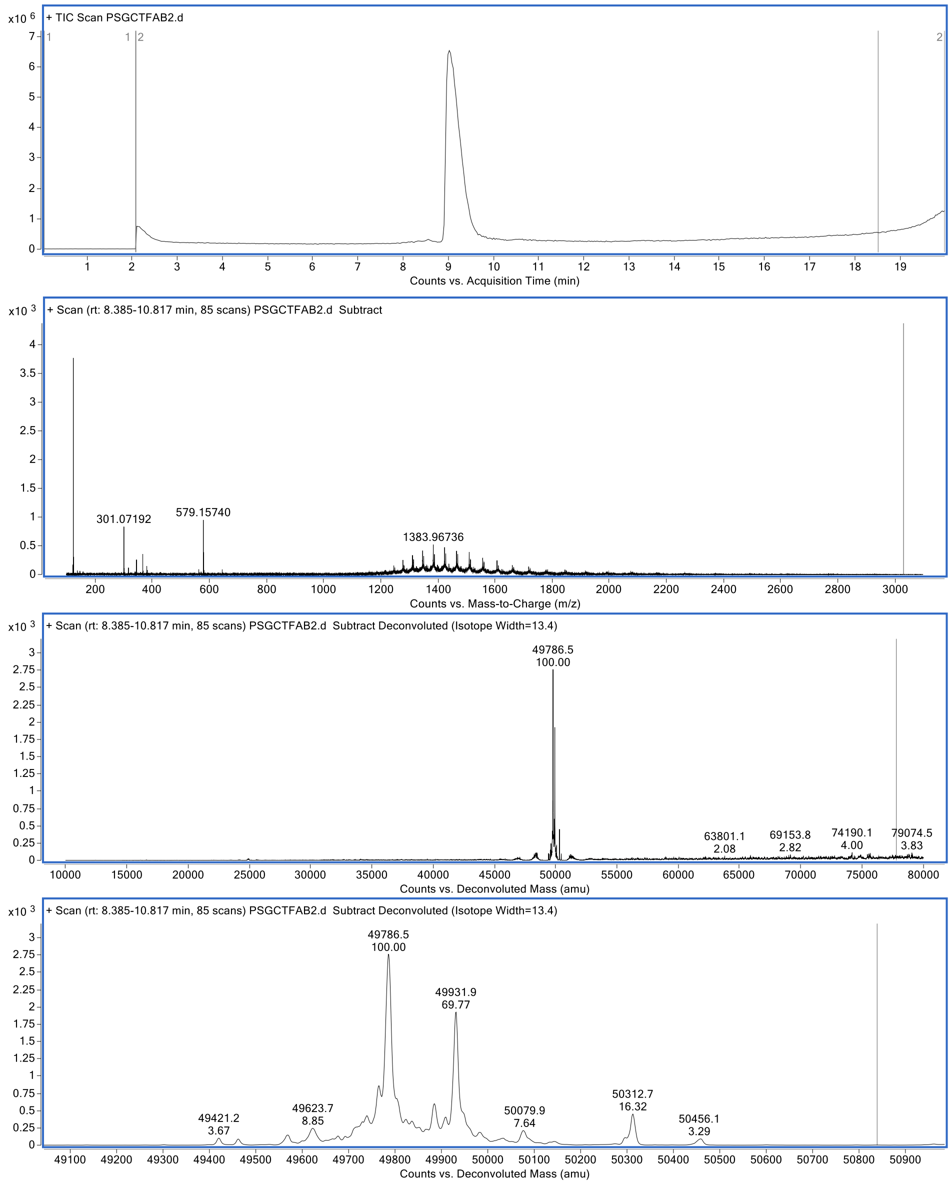

10.23. Fab$_{CD3}$ **S18**

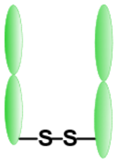

Observed mass: 47448.5 Da, 47562.5 Da.

Mass difference between peak 1 and 2: 114 Da (asparagine residue (114.1 Da)?).

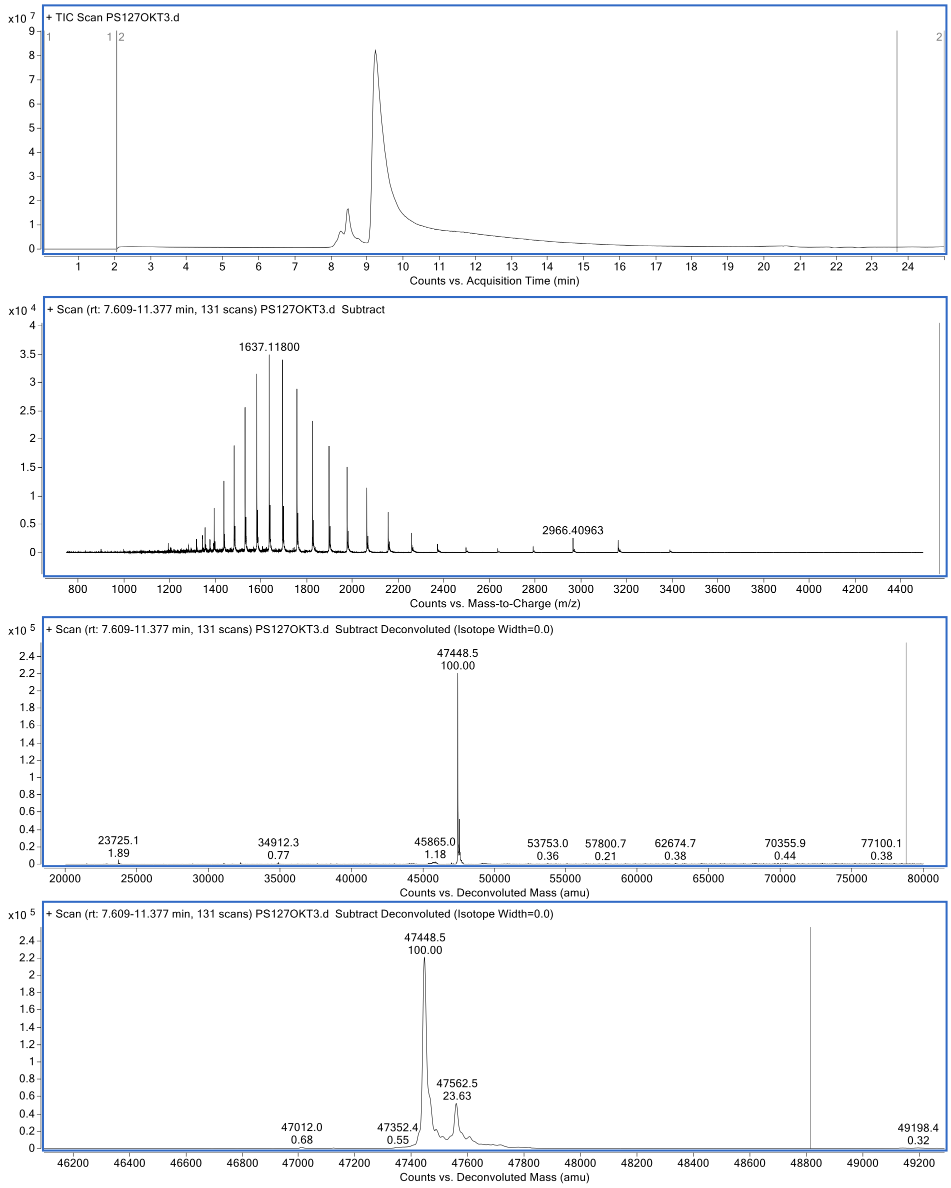

10.24. Fab$_{CTLA\text{-}4}$ **S19**

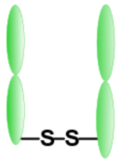

Observed mass: 47628.7 Da, 47741.6 Da.
Mass difference between peak 1 and 2: 113 Da (asparagine (114 Da)/leucine (113 Da)/isoleucine (113 Da)/aspartic acid residue (115 Da)).

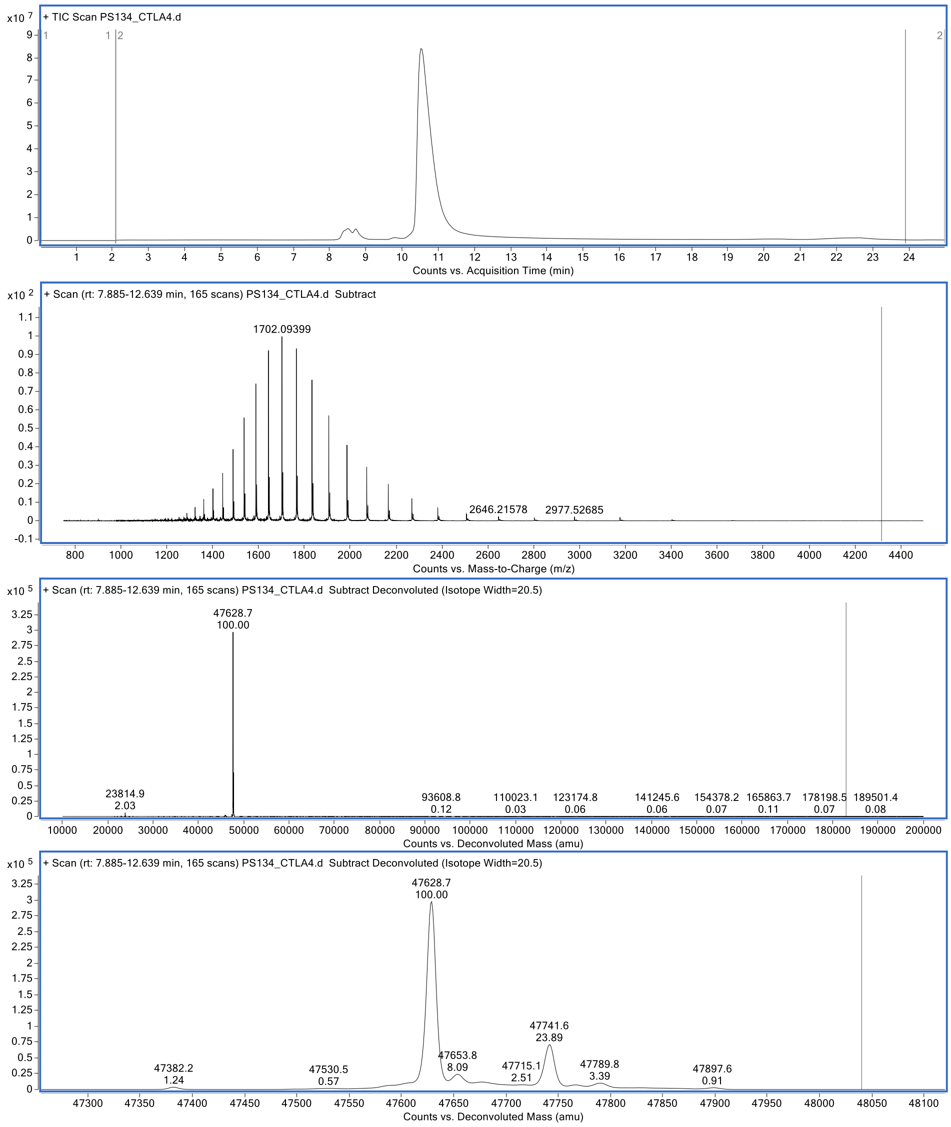

10.25. Fab$_{ICOS}$ **S20**

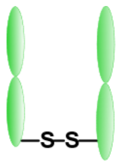

Observed mass: 47880.5 Da.
Some HC and LC also present.

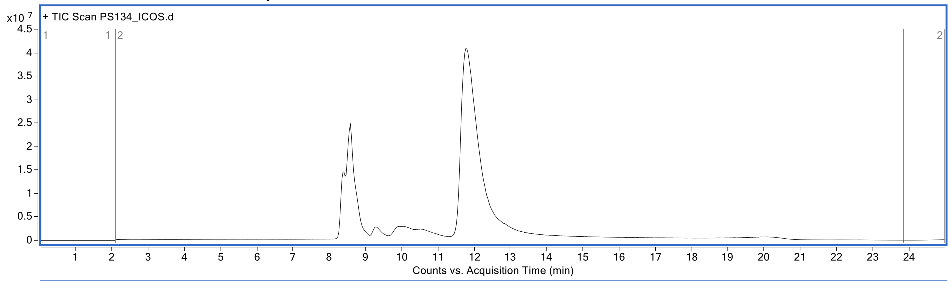

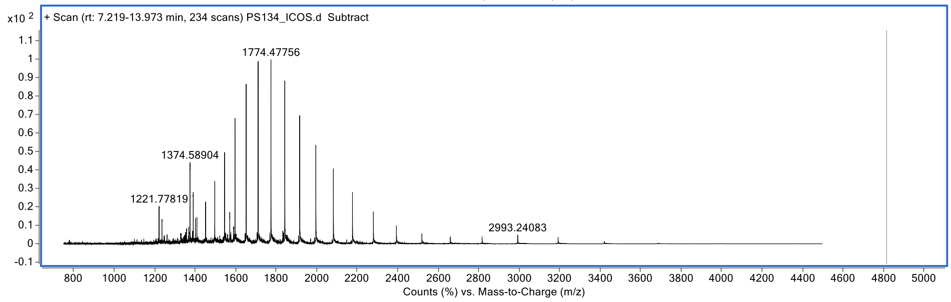

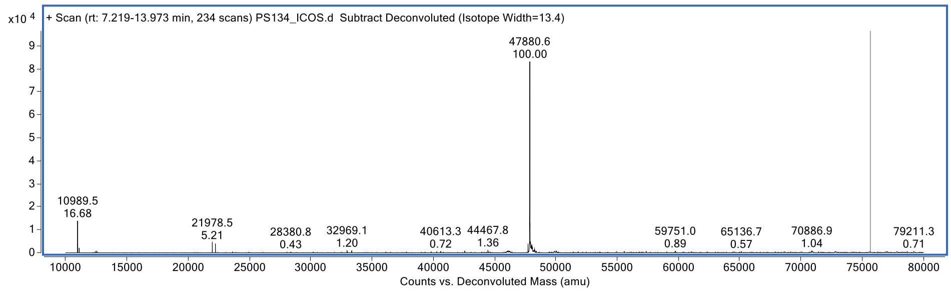

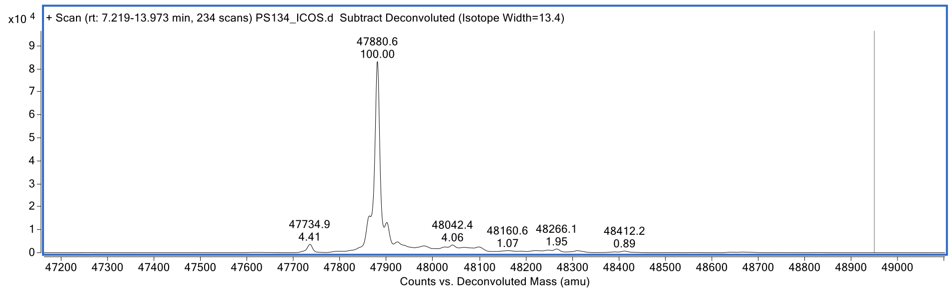

10.26. Fab_{HER2} **S21**

Expected mass: 47638 Da. Observed mass: 47639 Da.

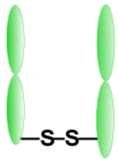

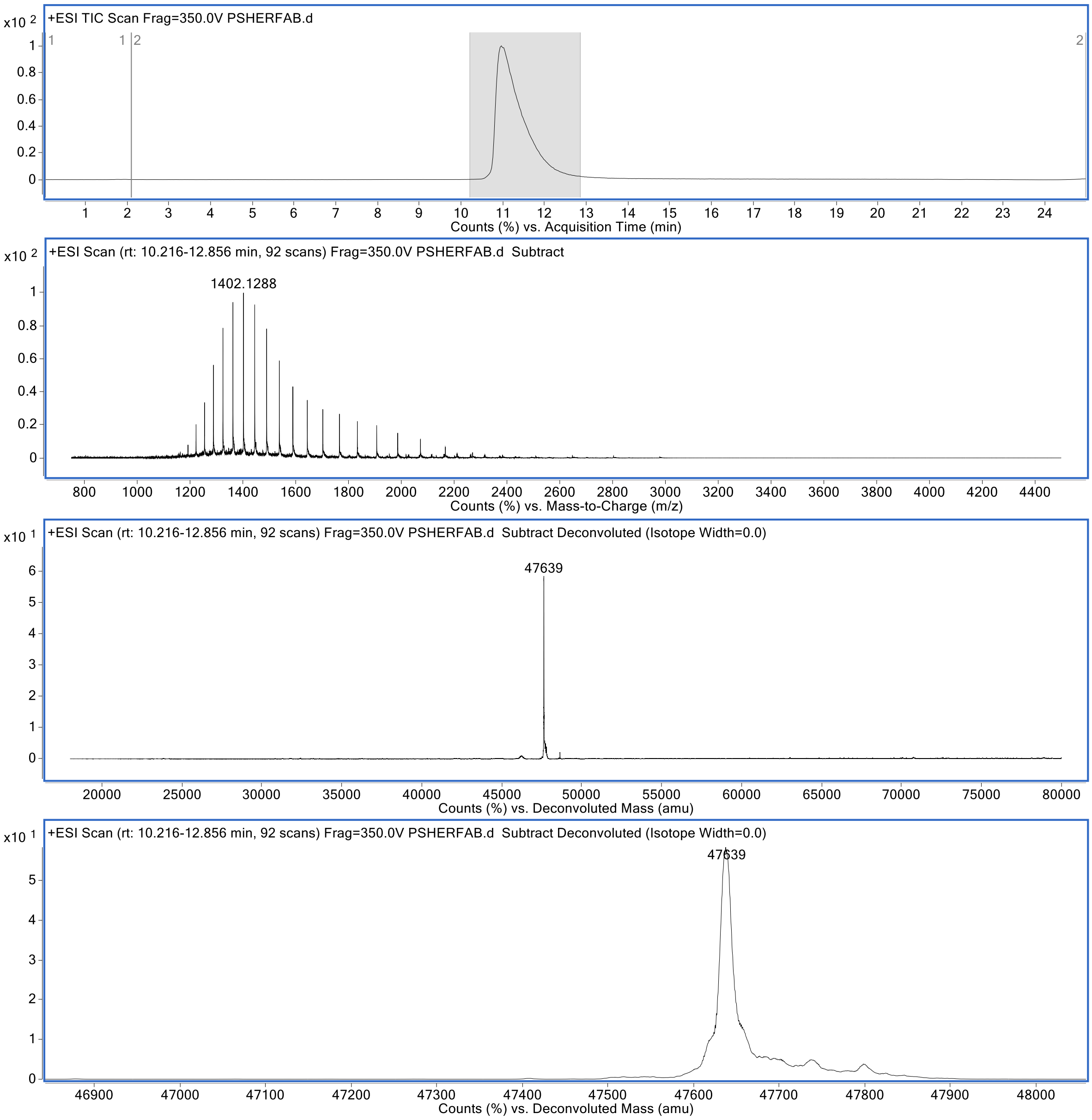

10.27. Fab$_{CD20}$ **S22**

Expected mass: 47181 Da. Observed mass: 47180 Da.

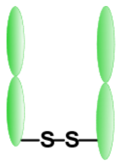

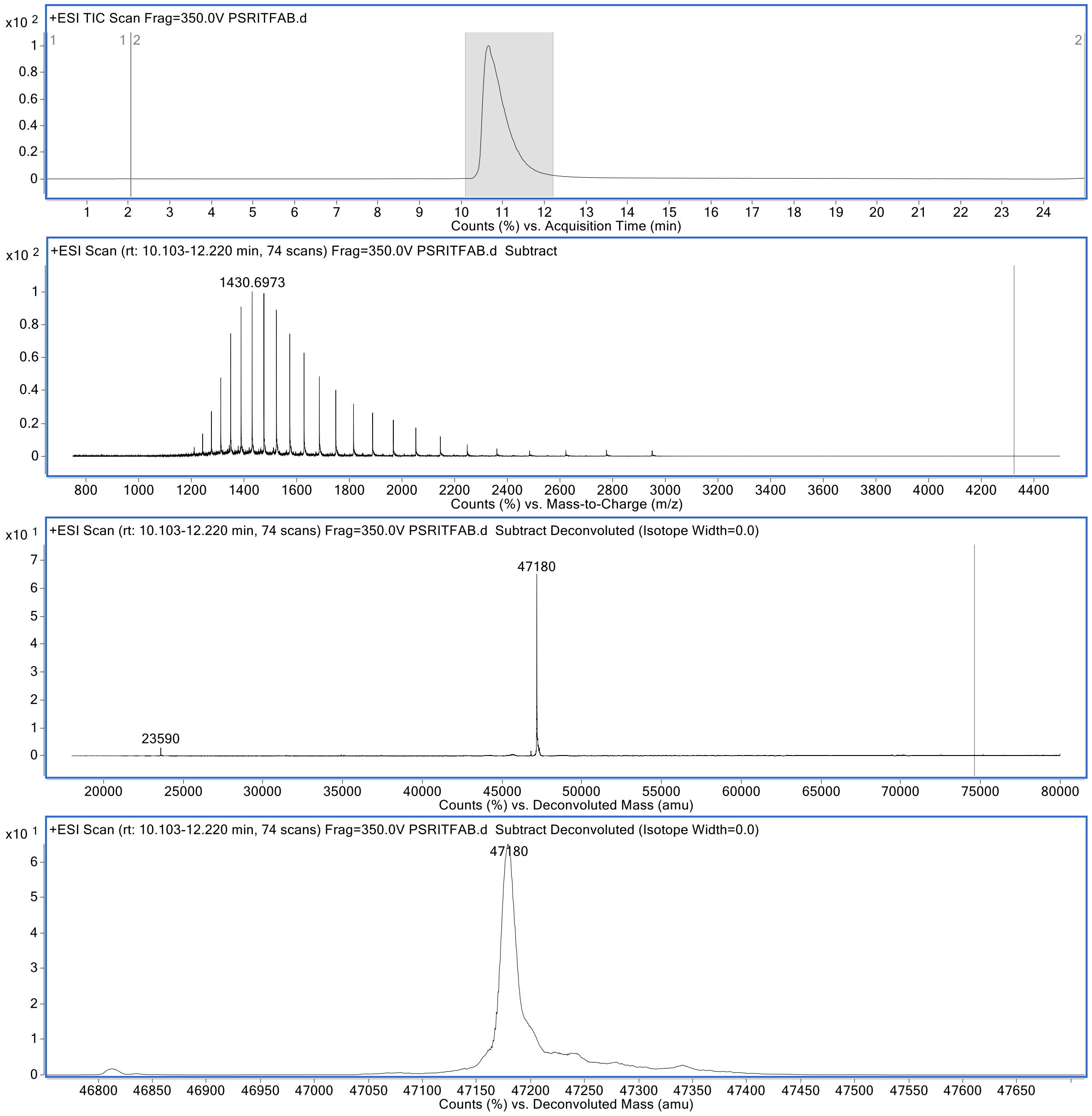

10.28. Fab$_{EGFR}$-Tet **S24**

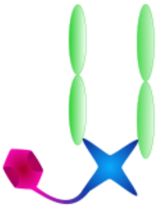

Expected mass: 50481.4 Da, 50628.5 Da.

Observed mass: 50481.9 Da, 50627.2 Da.

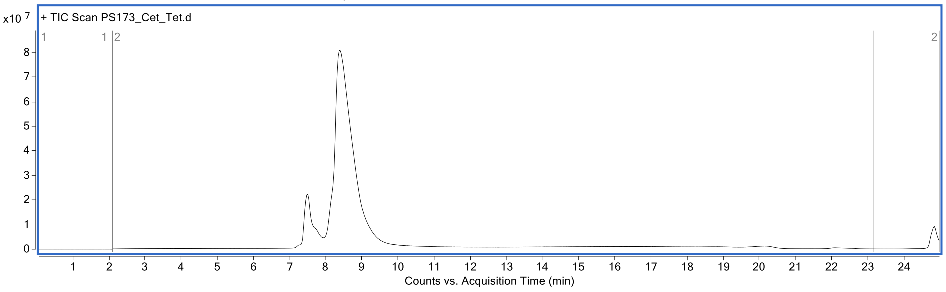

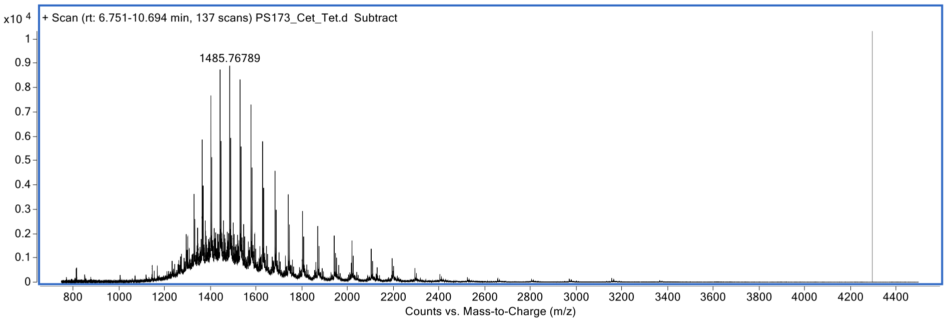

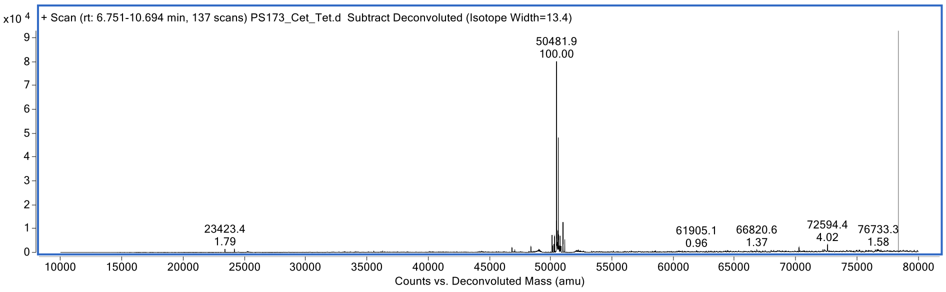

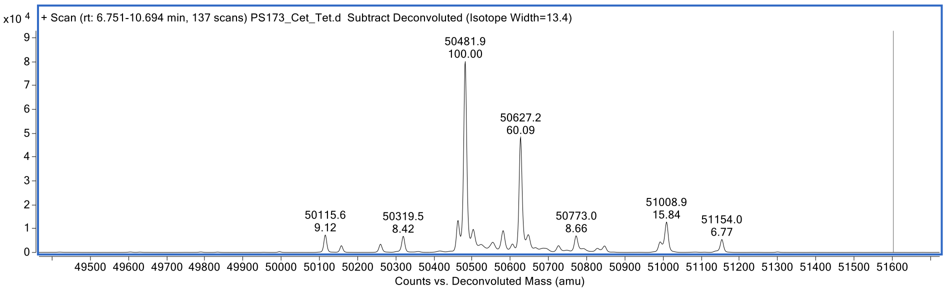

10.29. Fab_{CD20}-BCN **S25**

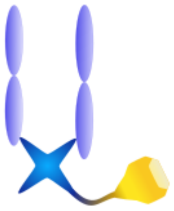

Expected mass: 47683.0 Da.
Observed mass: 47682.0 Da.

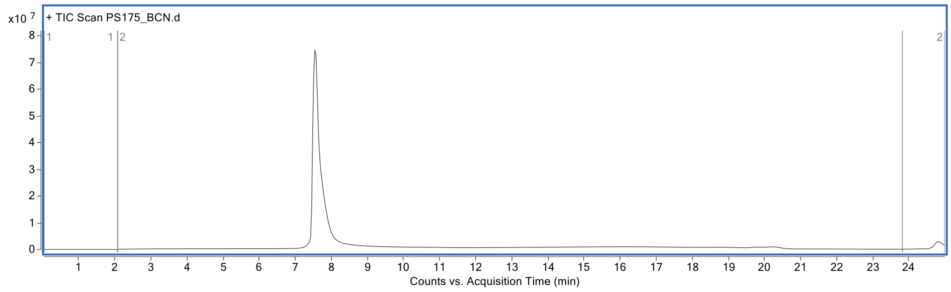

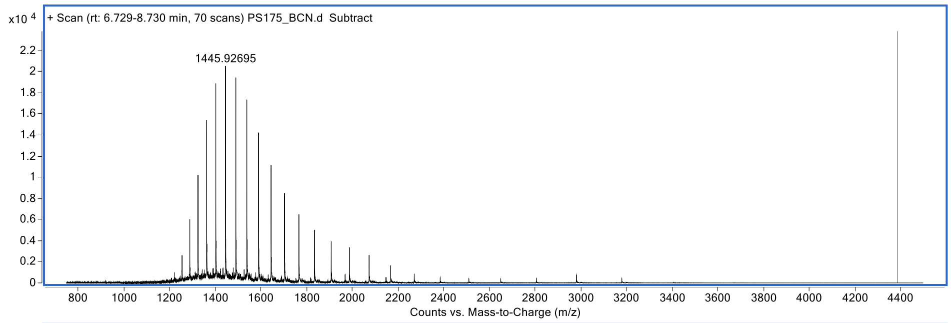

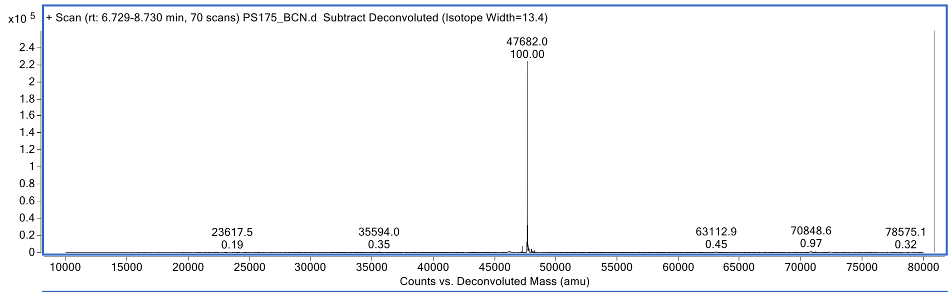

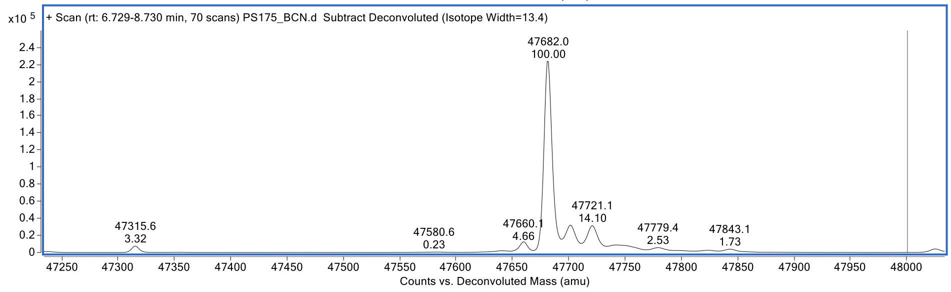

10.30. Fab$_{HER2}$-Tet **S26**

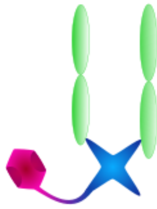

Expected mass: 48334 Da.

Observed mass: 48321 Da (as expected as MS was mis-calibrated by 14 Da, showing native Fab$_{HER2}$ at 47625 Da).

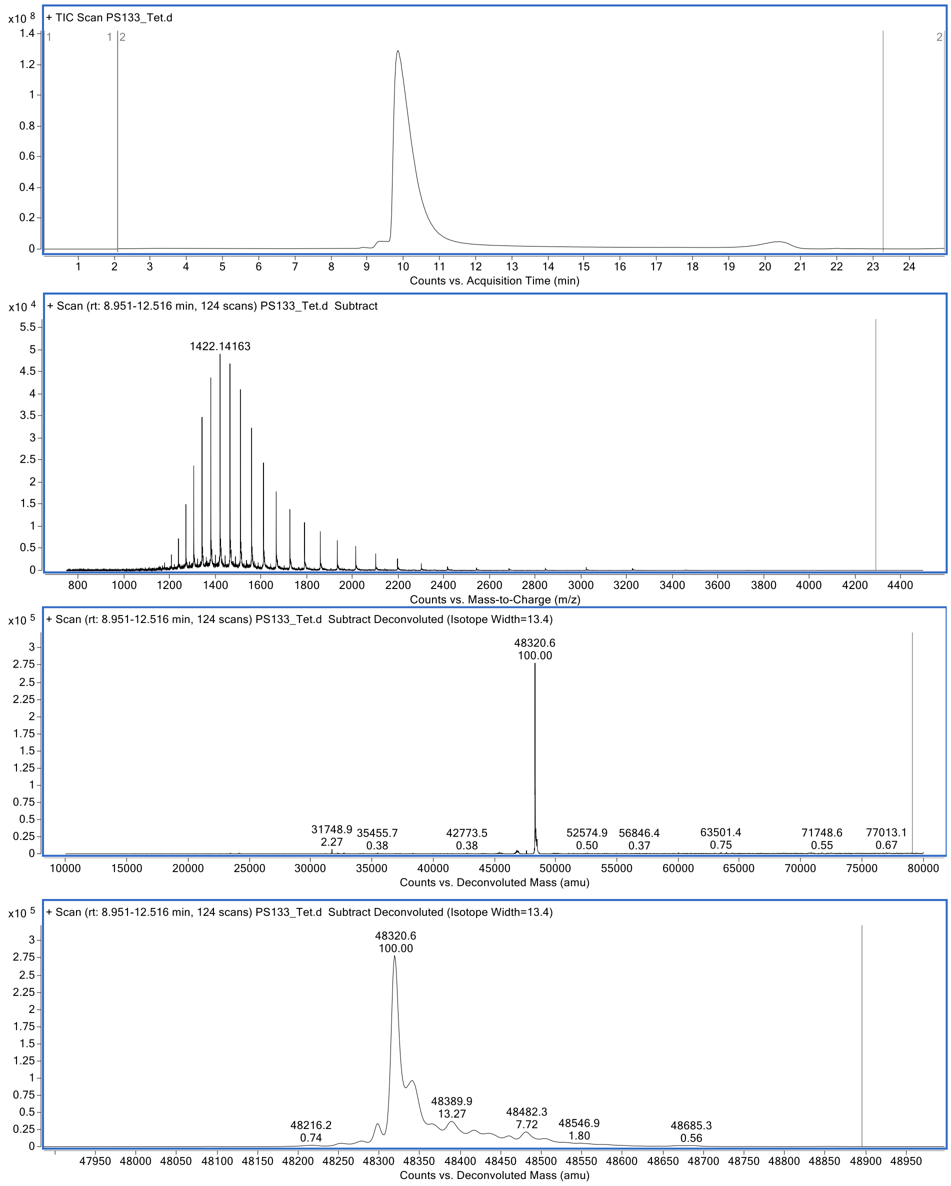

10.31. Sia-BCN **S28**

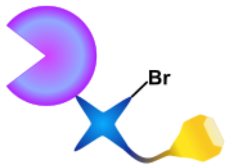

Expected mass: 46578 Da. Observed: 46578 Da.

Other mass envelope (including peak at m/z: 551.4) due to PEG impurity from Zeba spin purification.

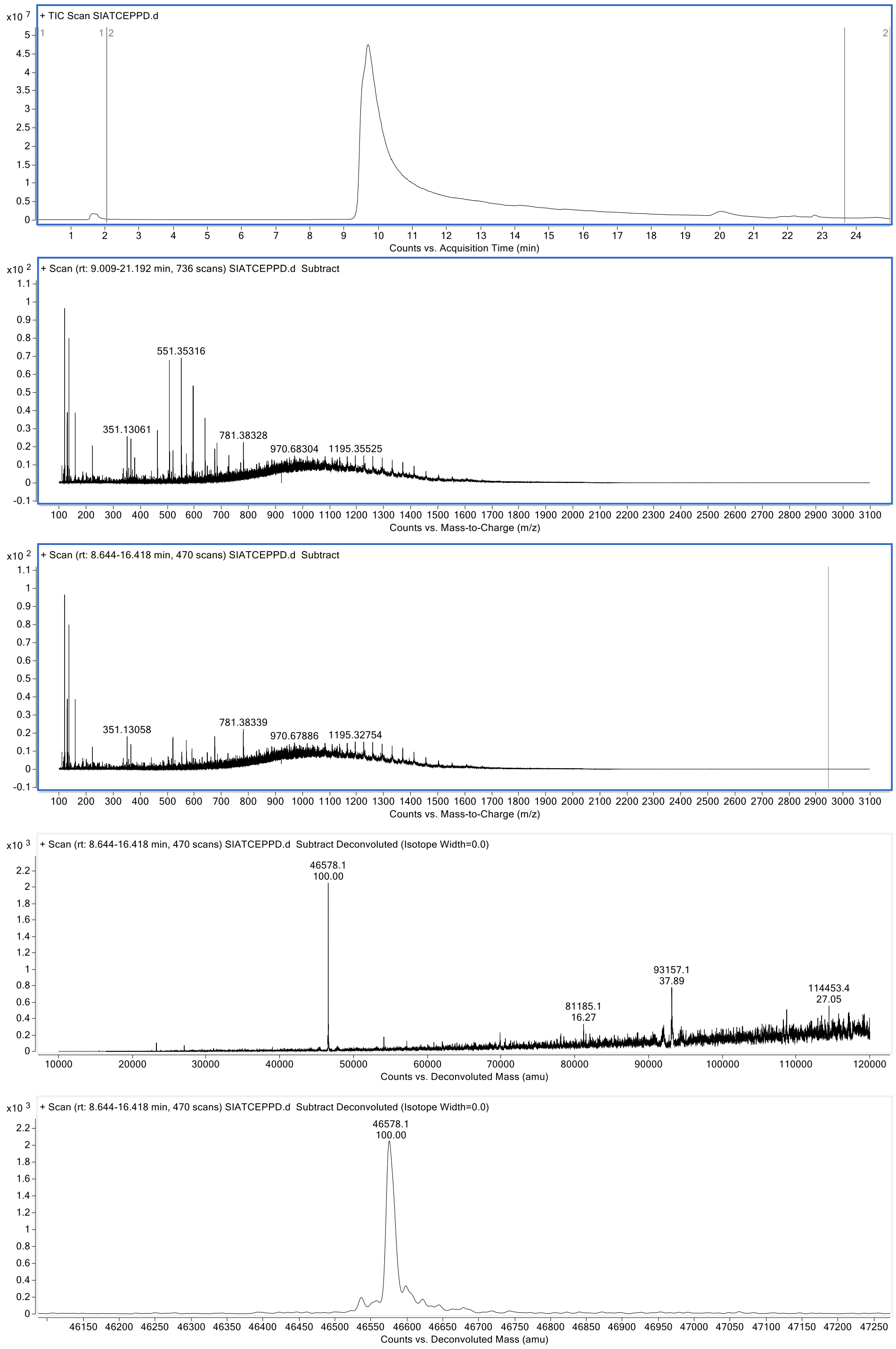

10.32. Fab$_{CD3}$-BCN **S30**

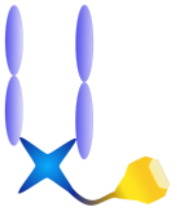

Expected mass: 47948 Da. Observed: 47951 Da.

Mass of 48062 Da probably arising from under digestion of Fab$_{CD3}$. Mass difference of ~111 Da could correspond to one more leucine or isoleucine residue (mass: 113 Da).

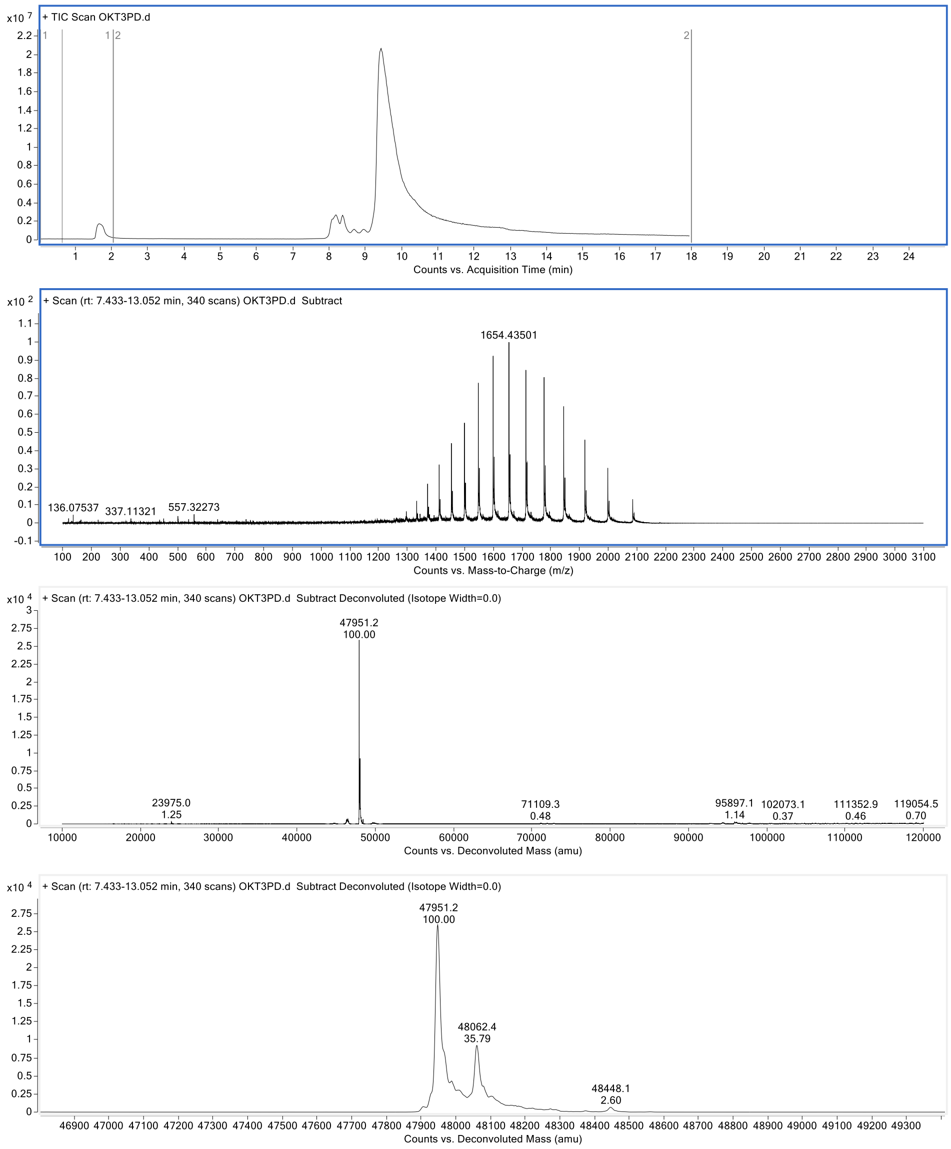

10.33. Fab_{HER2}-Fab_{CD20}-$PDBr_2$ **S32**

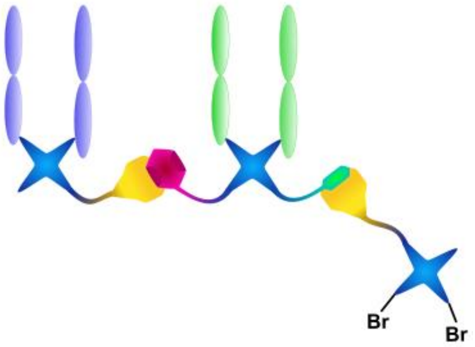

Expected mass: 96888 Da. Observed: 96870 Da.

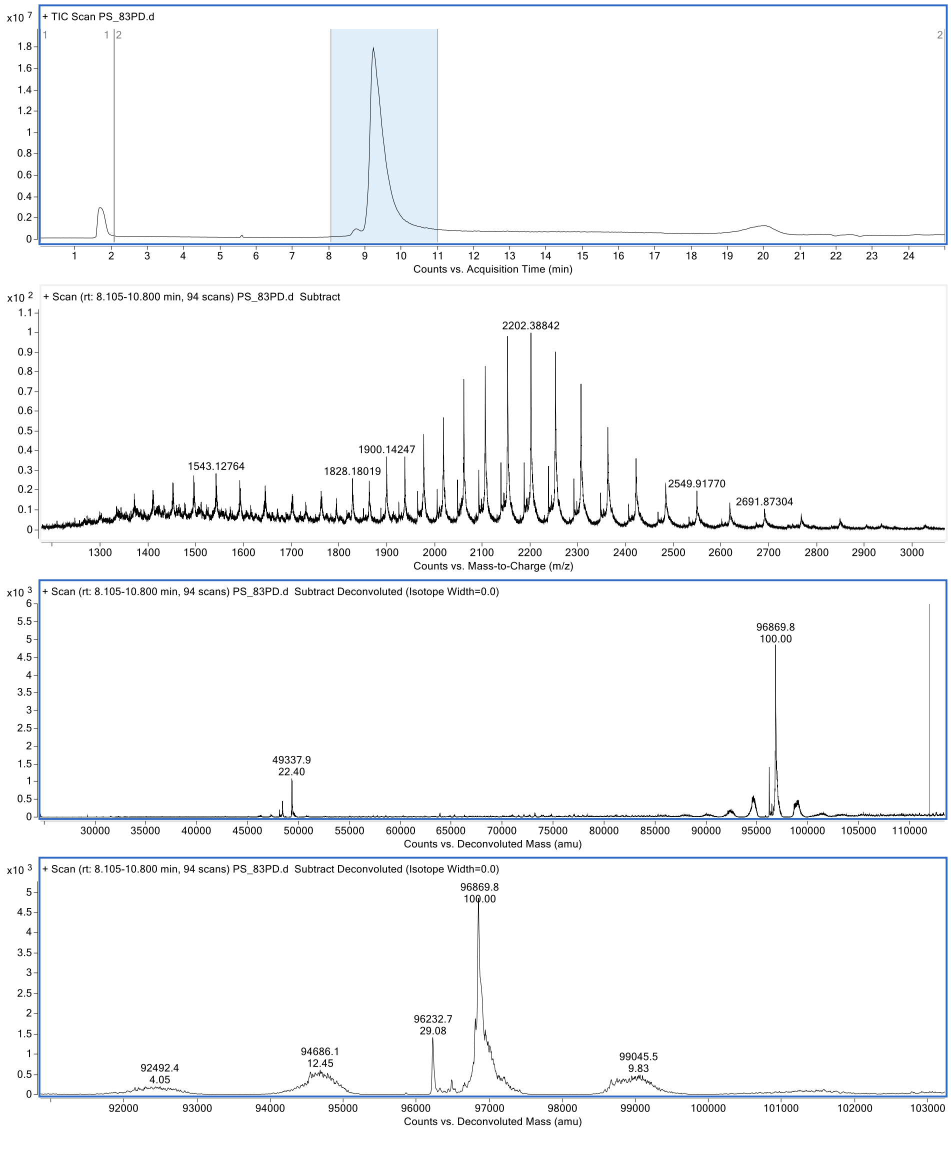

10.34. Fab_{HER2}-Fab_{CD20}-Fab_{EGFR} **S33**

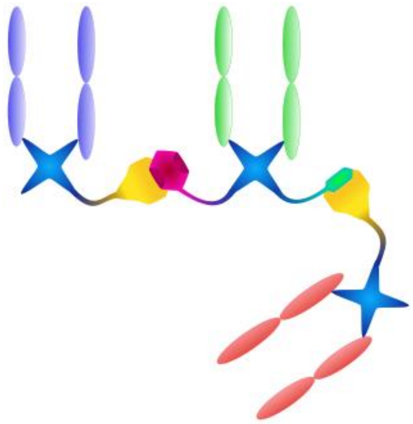

Expected mass: 146512 and 146659 Da. Observed: 146530 and 146677 Da.

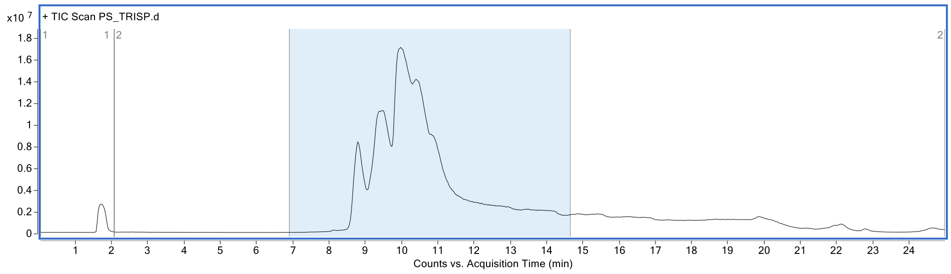

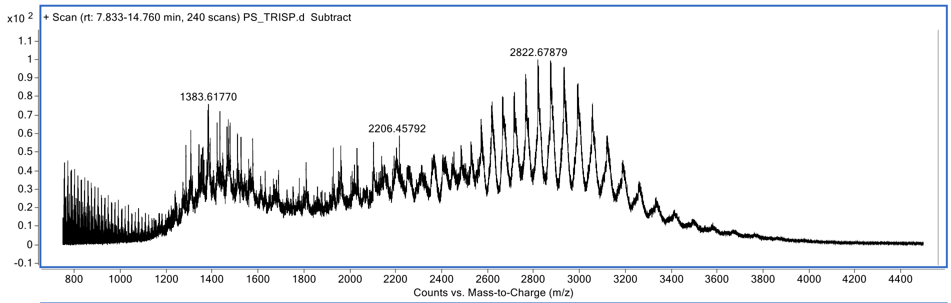

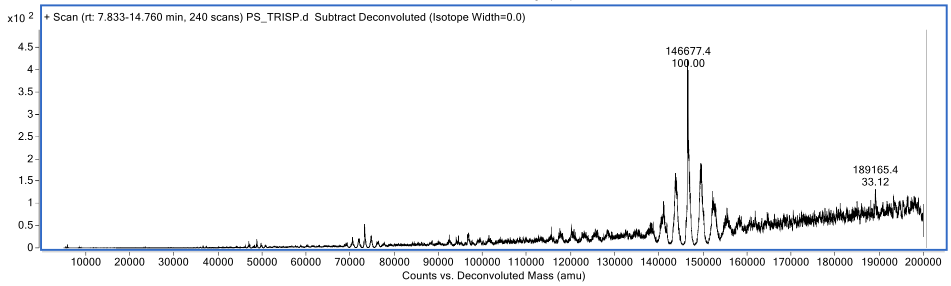

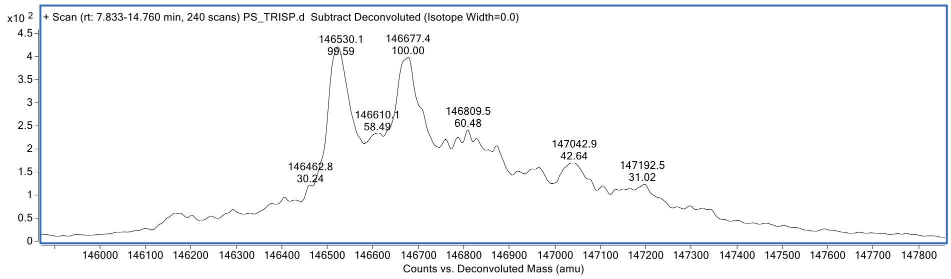

10.35. Fab_{CD20}-Fab_{HER2}-$PDBr_2$ **S34**

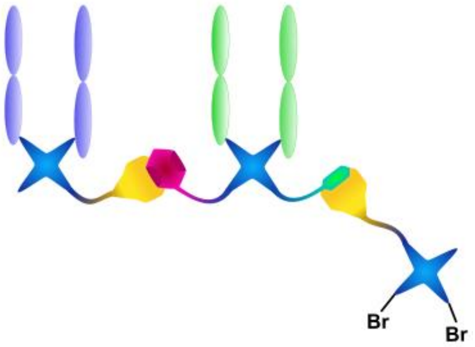

Expected mass: 96891 Da.
Observed mass: 96894 Da.

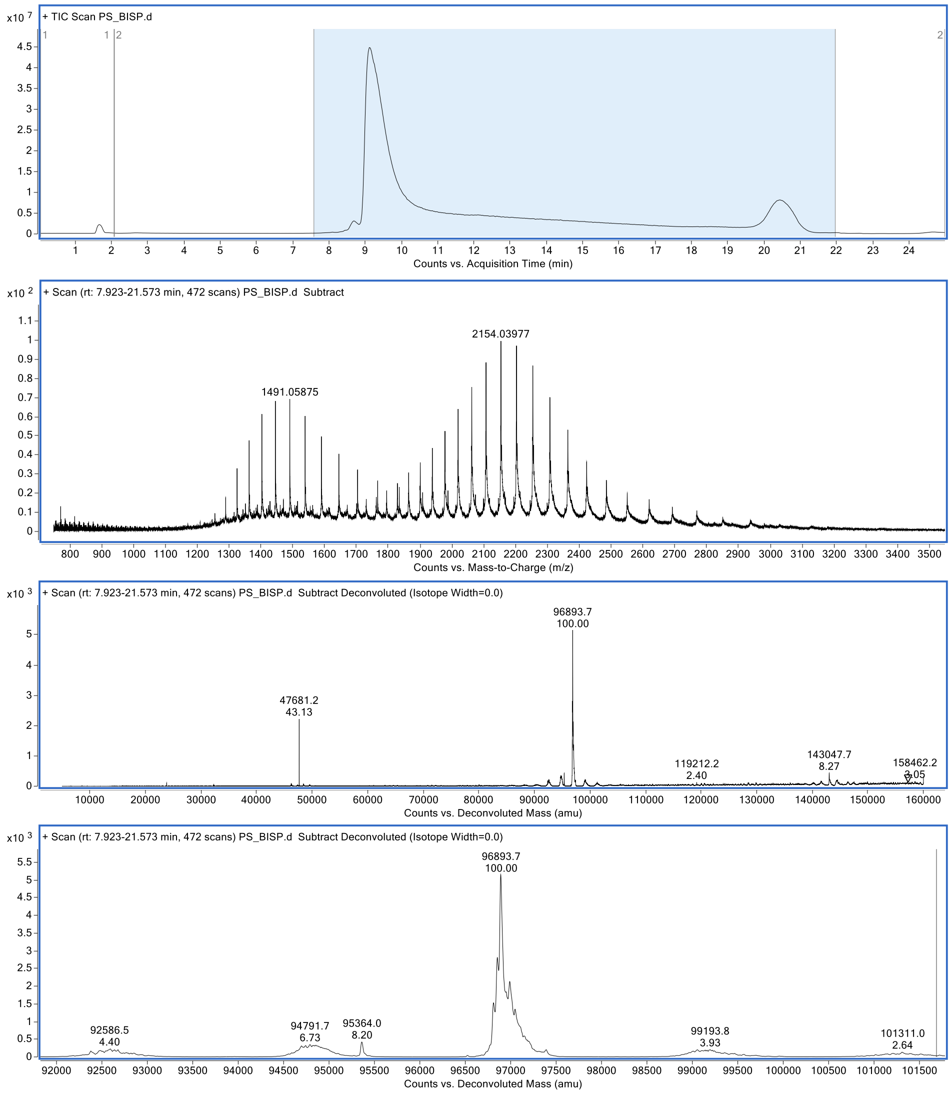

10.36. Fab$_{CD20}$-Fab$_{HER2}$-PDBr **S35**

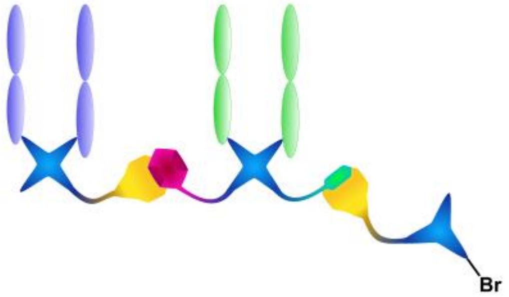

Expected mass: 96812.5 Da.

Observed mass: 96995.7 Da (+183.2 Da, +TCEP oxidized?), 96749.4 Da (-63.1 Da).

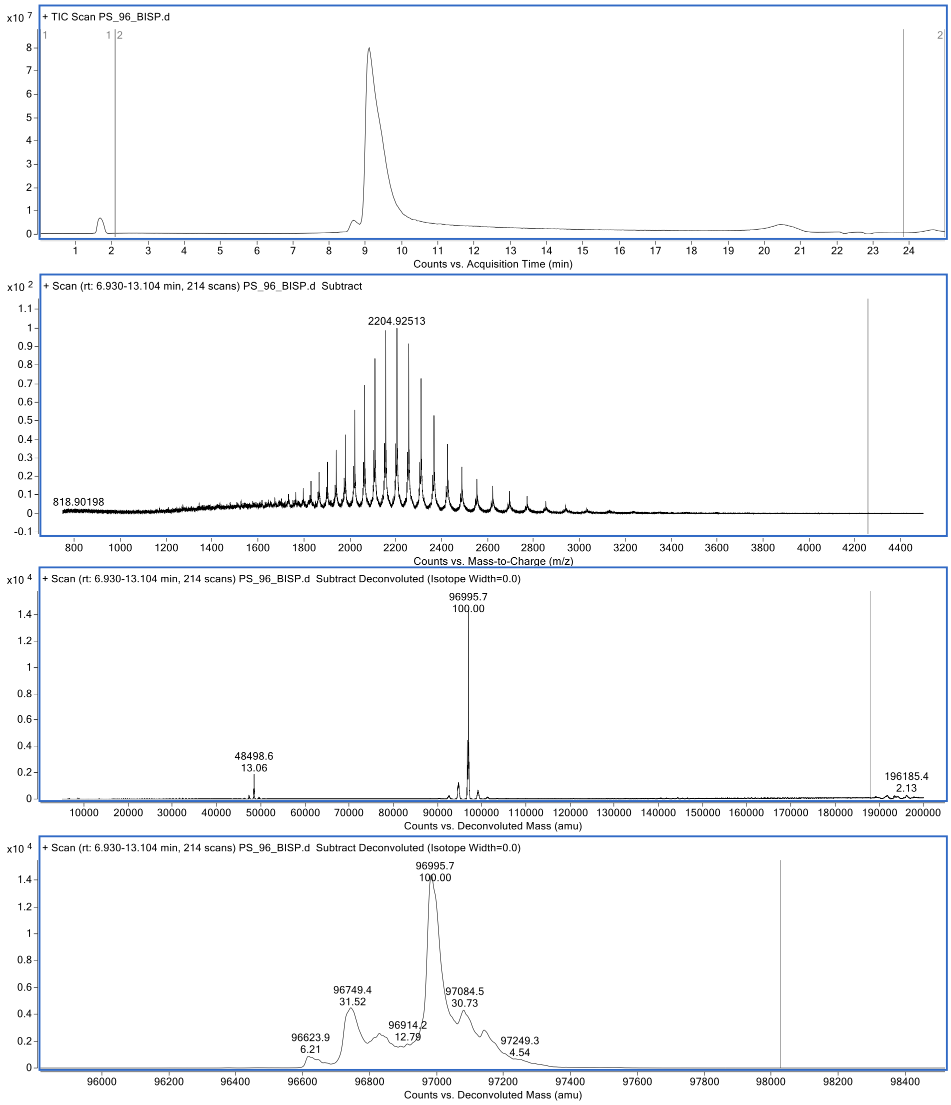

10.37. Fab_{CD20}-Fab_{HER2}-Sia_2 **S36**

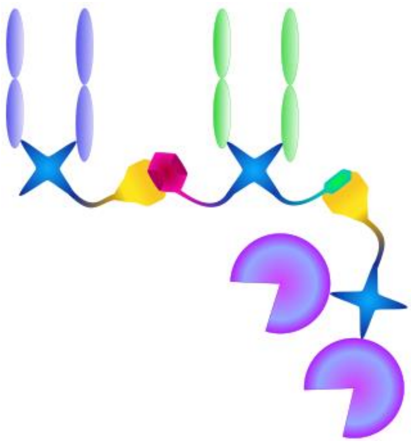

Expected mass: 188720 Da.
Observed mass: 188753 Da.

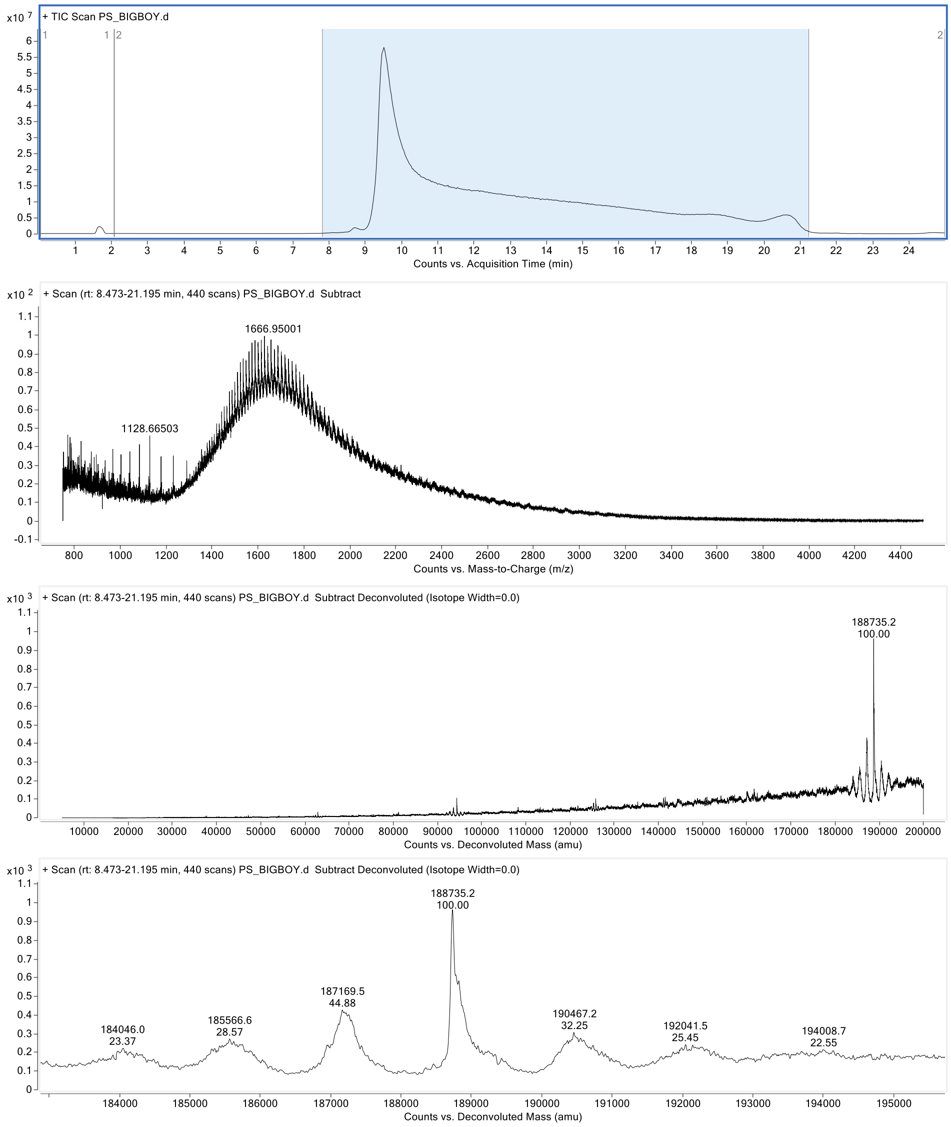

10.38. Fab_{CD20}-Fab_{HER2}-Sia **S37**

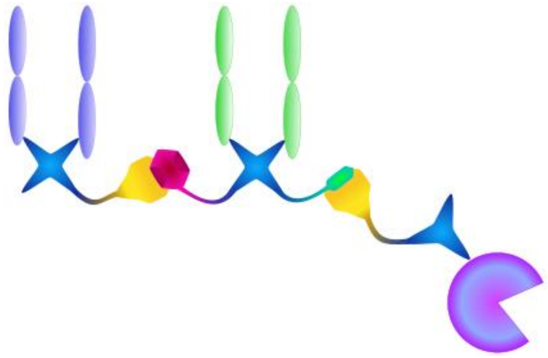

Expected mass: 142725 Da. Observed: 142750 Da.

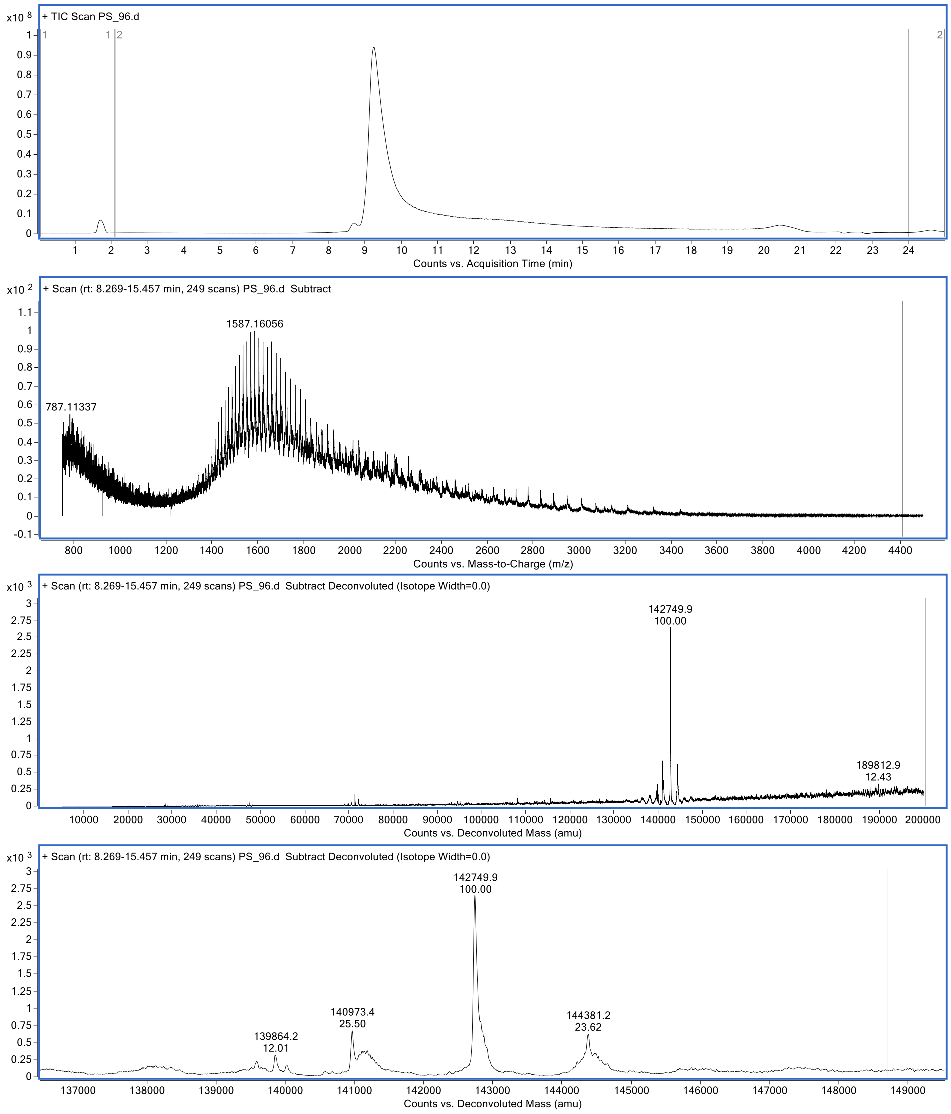

11. References

1. Maruani, A. *et al.* A plug-and-play approach to antibody-based therapeutics via a chemoselective dual click strategy. *Nat. Commun.* **6**, 6645 (2015).

2. Bahou, C. *et al.* Highly homogeneous antibody modification through optimisation of the synthesis and conjugation of functionalised dibromopyridazinediones. *Org. Biomol. Chem.* **16**, 1359–1366 (2018).

3. Bahou, C. *et al.* A Plug-and-Play Platform for the Formation of Trifunctional Cysteine Bioconjugates that also Offers Control over Thiol Cleavability. *Bioconjugate Chem.* **32**, 672–679 (2021).

4. Nogueira, J. C. F. *et al.* Oriented attachment of VNAR proteins,: Via site-selective modification, on PLGA-PEG nanoparticles enhances nanoconjugate performance. *Chem. Commun.* **55**, 7671–7674 (2019).

5. Slaga, D. *et al.* Avidity-based binding to HER2 results in selective killing of HER2-overexpressing cells by anti-HER2/CD3. *Sci. Transl. Med.* **10**, 5775 (2018).

6. Gray, M. A. *et al.* Targeted glycan degradation potentiates the anticancer immune response in vivo. *Nat. Chem. Biol.* **16**, 1376–1384 (2020).

7. Edgar, L. J. *et al.* Sialic Acid Ligands of CD28 Suppress Costimulation of T Cells. *ACS Cent. Sci.* **7**, 1508–1515 (2021).

8. Herrmann, M. *et al.* Bifunctional PD-1 3 aCD3 3 aCD33 fusion protein reverses adaptive immune escape in acute myeloid leukemia. *Blood* **132**, 2484–2494 (2018).

9. Lang, K. *et al.* Genetic encoding of bicyclononynes and trans-cyclooctenes for site-specific protein labeling in vitro and in live mammalian cells via rapid fluorogenic diels-alder reactions. *J. Am. Chem. Soc.* **134**, 10317–10320 (2012).

10. Agramunt, J., Ginesi, R., Pedroso, E. & Grandas, A. Inverse Electron-Demand Diels-Alder Bioconjugation Reactions Using 7-Oxanorbornenes as Dienophiles. *J. Org. Chem.* **85**, 6593–6604 (2020).

11. Hernández-Gil, J. *et al.* Development of 68Ga-labelled ultrasound microbubbles for whole-body PET imaging. *Chem. Sci.* **10**, 5603–5615 (2019).

12. Maruani, A. *et al.* A Plug-and-Play Approach for the de Novo Generation of Dually Functionalized Bispecifics. *Bioconjugate Chem.* **31**, 520–529 (2020).

13. Rasmussen, L. K. Facile synthesis of mono-, di-, and trisubstituted alpha-unbranched hydrazines. *J. Org. Chem.* **71**, 3627–3629 (2006).

14. Zhao, Y. *et al.* Two routes for production and purification of Fab fragments in biopharmaceutical discovery research: Papain digestion of mAb and transient expression in mammalian cells. *Protein Expr. Purif.* **67**, 182–189 (2009).

15. Moore, H. M. *et al.* Biospecimen reporting for improved study quality (BRISQ). *Cancer Cytopathol.* **119**, 92–102 (2011).
