# Supplementary material for: Chemical generation of checkpoint inhibitory T cell engagers for the treatment of cancer
Source: Nat Chem. Author manuscript; Available in PMC 2024 Feb 16. (PMC10624612; doi:10.1038/s41557-023-01280-4)
Supplement: supplementary information 2 [file NIHMS1922194-supplement-supplementary_information_2.pdf]

nature portfolio

Corresponding author(s): Carolyn R. Bertozzi
Vijay Chudasama

Last updated by author(s): Apr 24, 2023

Reporting Summary

Nature Portfolio wishes to improve the reproducibility of the work that we publish. This form provides structure for consistency and transparency in reporting. For further information on Nature Portfolio policies, see our Editorial Policies and the Editorial Policy Checklist.

Statistics

For all statistical analyses, confirm that the following items are present in the figure legend, table legend, main text, or Methods section.

n/a	Confirmed	
☐	☒	The exact sample size (n) for each experimental group/condition, given as a discrete number and unit of measurement
☐	☒	A statement on whether measurements were taken from distinct samples or whether the same sample was measured repeatedly
☐	☒	The statistical test(s) used AND whether they are one- or two-sided<br>*Only common tests should be described solely by name; describe more complex techniques in the Methods section.*
☒	☐	A description of all covariates tested
☐	☒	A description of any assumptions or corrections, such as tests of normality and adjustment for multiple comparisons
☐	☒	A full description of the statistical parameters including central tendency (e.g. means) or other basic estimates (e.g. regression coefficient) AND variation (e.g. standard deviation) or associated estimates of uncertainty (e.g. confidence intervals)
☐	☒	For null hypothesis testing, the test statistic (e.g. F, t, r) with confidence intervals, effect sizes, degrees of freedom and P value noted<br>*Give P values as exact values whenever suitable.*
☒	☐	For Bayesian analysis, information on the choice of priors and Markov chain Monte Carlo settings
☒	☐	For hierarchical and complex designs, identification of the appropriate level for tests and full reporting of outcomes
☒	☐	Estimates of effect sizes (e.g. Cohen's d, Pearson's r), indicating how they were calculated

Our web collection on statistics for biologists contains articles on many of the points above.

Software and code

Policy information about availability of computer code

Data collection	Flow cytometry data acquisition was carried out with a MACSQuant® Analyzer 10 Flow Cytometer (Miltenyi Biotec). For LC-MS an Agilent 6510 QTOF LC-MS system (Agilent, UK) was used. UV-vis spectroscopy was carried out with a NanoDropTM One microvolume UV-Vis spectrophotometer (Thermo Scientific™). All NMR results were obtained using Bruker NMR instruments, the models are as follows: Avance Neo 700, Avance III 600, Avance 500, Avance III 400. Purification by size exclusion chromatography (SEC) was carried out on an Agilent 1100 HPLC system (column: Superdex 200 increase, 10/300 GL) with a MALS system attached (Optilab T-rEX, Dawn8+ Heleos, Wyatt Technology). A SpectraMax i3x Multi-Mode Microplate Reader (Molecular Devices) was used to acquire the results for the cytotoxicity assay.
Data analysis	GraphPad Prism version 9 was used for statistical analysis. FlowJo, version 10.8.1 was used for flow cytometry analysis. MassHunter, version B.07.00 was used for LC-MS analysis. NMR analysis was carried out with MestReNova, version 6. The results of the cytotoxicity assay were analysed with SoftMax Pro 6.4.2. ProtParam (https://web.expasy.org/protparam/) was used to calculate extinction coefficients from known protein sequences where possible.

For manuscripts utilizing custom algorithms or software that are central to the research but not yet described in published literature, software must be made available to editors and reviewers. We strongly encourage code deposition in a community repository (e.g. GitHub). See the Nature Portfolio guidelines for submitting code & software for further information.

Data

Policy information about availability of data

All manuscripts must include a data availability statement. This statement should provide the following information, where applicable:

- Accession codes, unique identifiers, or web links for publicly available datasets
- A description of any restrictions on data availability
- For clinical datasets or third party data, please ensure that the statement adheres to our policy

The cell assay datasets along with the statistical analysis thereof has been made available in Prism format (.pzfx).

Human research participants

Policy information about studies involving human research participants and Sex and Gender in Research.

Reporting on sex and gender	No information available.
Population characteristics	No information available.
Recruitment	Leukoreduction system (LRS) chambers were obtained from healthy anonymous human donors who gave informed consent at the Stanford Blood Center. Tier 1 characteristics of human biospecimens reported according to BRISQ guidelines as follows. Biospecimen type: white blood cell concentrate of TrimaAccel® LRS chamber recovered after Plateletpheresis procedure. Product contains PBMCs, red blood cells, plasma, and negligible amount of anticoagulant (ACD-A). Anatomical or collection site: vein (venipuncture). Biospecimen disease status and clinical characteristics of patients: healthy donors, not routinely tested for infectious disease markers. Vital state: alive. Collection mechanism and parameters: blood draw and LRS filtration for platelet donation. RBCs were returned to the donor. Mechanism of stabilization: none. Type of long-term preservation: peripheral blood mononuclear cells (PBMCs) were separated from the LRS chambers using dentistry gradient separation with Ficoll-Paque (GE Healthcare Life Sciences), biospecimens were frozen in FBS + 10% DMSO in liquid nitrogen. Constitution and concentration of fixative/preservation solution: heat inactivated foetal bovine serum (FBS) with 10% DMSO solution and frozen at -80 °C in an insulated cooler before being transferred to liquid nitrogen for long-term storage. Storage and shipping temperatures: LRS chambers were held at 20-24 °C. Isolated PBMCs were stored in liquid nitrogen vapor (-196 ° C). Storage duration: <1 day in LRS chamber. <2 years for frozen isolated PBMCs. Composition assessment and selection: none.
Ethics oversight	Leukoreduction system (LRS) chambers were obtained from healthy anonymous human donors who gave informed consent at the Stanford Blood Center.

Note that full information on the approval of the study protocol must also be provided in the manuscript.

Field-specific reporting

Please select the one below that is the best fit for your research. If you are not sure, read the appropriate sections before making your selection.

☒ Life sciences ☐ Behavioural & social sciences ☐ Ecological, evolutionary & environmental sciences

For a reference copy of the document with all sections, see nature.com/documents/nr-reporting-summary-flat.pdf

Life sciences study design

All studies must disclose on these points even when the disclosure is negative.

Sample size	No sample size calculations were performed. In most cases three replicates were performed in in vitro cell experiment. Three replicates per experiment were considered sufficient in line with accepted practice in the field. More replication (i.e., biological plus technical, for 9 total experiments) was not performed to conserve the conjugates.<br>Exceptions: Figure 4/F where tumour cell lines were measured with single data points. As in this experiment multiple tumour cell lines were used and furthermore no statistical analysis was performed on this dataset, this was considered sufficient to show the trends in desialylation. Figure 4/I where the binding of BiTE 8 control to T cells was only measured with single data points due to there not being enough BiTE 8 left for three replicates by this point. Since the difference in binding between BiTE 8 and CiTE 27 was clearly marked, and this experiment was not essential for our main conclusions we considered this sufficient. These exceptions are clearly stated in the appropriate figure legends.
Data exclusions	There were no data exclusions.
Replication	In most cases three replicates were performed in in vitro cell experiment as described above.<br>Most Fab and sialidase conjugates were generated multiple times and found to have consistent masses corresponding to calculations via LC-MS analysis. The final three-protein conjugation strategy was carried out to generate three distinct conjugates (FabHER2-FabCD20-Sia-Biotin 20, CiTE 26 and CiTE 27), each of them once, and all three constructs showed the expected masses by LC-MS analysis. Synthetic organic chemistry reactions were performed 1-2 times and products were characterized by NMR, IR and MS as is accepted in the field.

Randomization	No randomization was performed. Randomization was not relevant as samples were from defined groups.
Blinding	No blinding was performed. The groups were defined by the investigators according to standard protocols in the field.

Reporting for specific materials, systems and methods

We require information from authors about some types of materials, experimental systems and methods used in many studies. Here, indicate whether each material, system or method listed is relevant to your study. If you are not sure if a list item applies to your research, read the appropriate section before selecting a response.

Materials & experimental systems

n/a	Involved in the study
☐	☒ Antibodies
☐	☒ Eukaryotic cell lines
☒	☐ Palaeontology and archaeology
☒	☐ Animals and other organisms
☒	☐ Clinical data
☒	☐ Dual use research of concern

Methods

n/a	Involved in the study
☒	☐ ChIP-seq
☐	☒ Flow cytometry
☒	☐ MRI-based neuroimaging

Antibodies

Antibodies used

Cetuximab (anti-EGFR, approved for use in humans, University College London Hospital), https://www.erbitux.com/

Rituximab (anti-CD20, approved for use in humans, University College London Hospital), https://www.rituxan.com/

Ontruzant (anti-HER2, approved for use in humans, University College London Hospital), https://www.ontruzant.com/

Herceptin (anti-HER2, approved for use in humans, University College London Hospital), https://www.herceptin.com/

Anti-PD-1 (J116, BioXCell #BE0188), https://bioxcell.com/invivomab-anti-human-pd-1-cd279-be0188

Anti-ICOS (C398.4A, BioLegend #31350), https://www.biolegend.com/fr-ch/products/purified-anti-human-mouse-rat-cd278-icos-antibody-2477

Anti-CTLA4 (BN13, BioXCell #BE0190), https://bioxcell.com/invivomab-anti-human-ctla-4-cd152-be0190

Anti-CD3 (OKT3, BioLegend #317347 or BioXCell #BE0001-2), https://www.biolegend.com/en-us/products/ultra-leaf-purified-anti-human-cd3-antibody-7745?GroupID=BLG4203, https://bioxcell.com/invivomab-anti-human-cd3-be0001-2

Streptavidin Alexa Fluor™ 647 conjugate (ThermoFisher S21374, 1:2000 dilution), https://www.thermofisher.com/order/catalog/product/S21374

Human Siglec-9 Fc (R&D Systems 1139-SL-050, 2 µg/mL), https://www.rndsystems.com/products/recombinant-human-siglec-9-fc-chimera-protein-cf_1139-sl

Rabbit IgG Alexa Flour 488-conjugated antibody (R&D Systems IC1051G, 1:375 dilution), https://www.rndsystems.com/products/rabbit-igg-alexa-fluor-488-conjugated-antibody_ic1051g

Validation

Validation data is available from the suppliers' websites. No further validation of antibodies was performed in this study. Cetuximab, Rituximab, Ontruzant and Herceptin are approved for use in humans, and have as such been extensively validated.

Anti-PD-1 (J116, BioXCell #BE0188), https://bioxcell.com/invivomab-anti-human-pd-1-cd279-be0188
Production: Purified from tissue culture supernatant in an animal free facility, Purification: Protein G, Purity >95% Determined by SDS-PAGE, Endotoxin <2EU/mg (<0.002EU/µg) Determined by LAL gel clotting assa

Anti-ICOS (C398.4A, BioLegend #313502), https://www.biolegend.com/fr-ch/products/purified-anti-human-mouse-rat-cd278-icos-antibody-2477)
The antibody was purified by affinity chromatography.

Anti-CTLA4 (BN13, BioXCell #BE0190), https://bioxcell.com/invivomab-anti-human-ctla-4-cd152-be0190
Production: Purified from tissue culture supernatant in an animal free facility, Purification: Protein G, Purity >95% Determined by SDS-PAGE, Endotoxin <2EU/mg (<0.002EU/µg) Determined by LAL gel clotting assay.

Anti-CD3 (OKT3, BioLegend #317347), https://www.biolegend.com/en-us/products/ultra-leaf-purified-anti-human-cd3-antibody-7745?GroupID=BLG4203
The Ultra-LEAF™ (Low Endotoxin, Azide-Free) antibody was purified by affinity chromatography. Endotoxin level is <0.01 EU/µg of the protein (<0.001 ng/µg of the protein) as determined by the LAL test.

Anti-CD3 (OKT3, BioXCell #BE0001-2), https://bioxcell.com/invivomab-anti-human-cd3-be0001-2
Production: Purified from tissue culture supernatant in an animal free facility, Purification: Protein G, Purity >95% Determined by SDS-PAGE, Endotoxin <2EU/mg (<0.002EU/μg) Determined by LAL gel clotting assay.

Streptavidin Alexa Fluor™ 647 conjugate (ThermoFisher S21374), https://www.thermofisher.com/order/catalog/product/S21374
Validated by absorption at 652 nm. Emission maximum at 670 nm. Microscopy: Staining of HEp2 cells by human anti-nuclear antibody, DSB-X biotin goat anti-human and this product: good nuclear staining, negligible background. TLC: negligible or no free dye detected.

Human Siglec-9 Fc (R&D Systems 1139-SL-050), https://www.rndsystems.com/products/recombinant-human-siglec-9-fc-chimera-protein-cf_1139-sl
Purity >97%, by SDS-PAGE visualized with Silver Staining and quantitative densitometry by Coomassie® Blue Staining.
Endotoxin Level <0.10 EU per 1 μg of the protein by the LAL method.
Activity Measured by the ability of the immobilized protein to support the adhesion of human red blood cells. Kelm, S. et al. (1994) Current Biology 4:965. The ED50 for this effect is 10.0-100 ng/mL.

Rabbit IgG Alexa Flour 488-conjugated antibody (R&D Systems IC1051G), https://www.rndsystems.com/products/rabbit-igg-alexa-fluor-488-conjugated-antibody_ic1051g
Protein A or G purified from cell culture supernatant

Eukaryotic cell lines

Policy information about cell lines and Sex and Gender in Research

Cell line source(s)	SKBR3, HCC-1954, BT-20, MDA-MB-468 and MDA-MB-231 cell lines were purchased from American Type Culture Collection.
Authentication	Cell lines have not been subjected to additional authentication.
Mycoplasma contamination	All cell lines regularly tested negative for mycoplasma infection by the Lonza Mycoplasma Detection Assay.
Commonly misidentified lines (See ICLAC register)	No commonly misidentified cell lines were used in this study.

Flow Cytometry

Plots

Confirm that:

☒ The axis labels state the marker and fluorochrome used (e.g. CD4-FITC).

☒ The axis scales are clearly visible. Include numbers along axes only for bottom left plot of group (a 'group' is an analysis of identical markers).

☒ All plots are contour plots with outliers or pseudocolor plots.

☒ A numerical value for number of cells or percentage (with statistics) is provided.

Methodology

Sample preparation	SKBR3, HCC-1954, BT-20, MDA-MB-468 and MDA-MB-231 cell lines were purchased from American Type Culture Collection and cultured in filtered Dulbecco's Modified Eagle's Medium/Nutrient Mixture F-12 Ham media with 10% heat-inactivated FBS and no added antibiotics or cultured as suggested. Cultures were grown in T25 and T75 flasks and maintained at 37 °C with 5% CO2. Where required, cells were induced for the expression of human PD-L1 by incubation with interferon gamma (IFN-γ) (PeproTech 300-02) at 100 ng/mL for 48 h and lifted with Enzyme Free Cell Dissociation Solution PBS Based (MilliporeSigma S-014-M) before flow cytometry and cytotoxicity assays.<br>Cells were stained with either Zombie NIR (Biolegend 423106) or Zombie Violet (Biolegend 423113) Fixable Viability Kits according to manufacturer protocols and fixed with 4% Paraformaldehyde (Ted Pella 18505) prior to analysis. Washing and staining were performed in PBS with 0.5% BSA. Binding was determined by incubating the constructs with 100,000 cells for 30 min at 4 °C, followed by incubating with Streptavidin Alexa Fluor™ 647 conjugate (ThermoFisher S21374) for 30 min at 4 °C. Desialylation activity was determined by incubating cells for 30 min at 37 °C with the constructs, then detecting binding with a 1:1 molar mixture of recombinant Human Siglec-9 Fc (R&D Systems 1139-SL-050) and rabbit IgG Alexa Flour 488-conjugated antibody (R&D Systems IC1051G). Data points were normalized to the maximum mean fluorescence intensity.<br>LRS chambers were obtained from healthy human donors from the Stanford Blood Bank. Peripheral blood mononuclear cells (PBMC) were separated from the chambers using dentistry gradient separation with Ficoll-Paque (GE Healthcare Life Sciences). T cells were isolated using immunomagnetic negative selection EasySep™ Human T Cell Isolation Kit StemCell (STEMCELL Technologies, 17951) followed by activation for 5 days with human T-Activator CD3/CD28 Dynabeads™ (ThermoFisher, 11131D) and 30 IU/mL recombinant human Interleukin-2 (IL-2) (PeproTech, 200-02).
Instrument	MACSQuant® Analyzer 10 Flow Cytometer (Miltenyi Biotec)
Software	FlowJo, version 10.8.1
Cell population abundance	No sorting was performed, the entirety of the cell samples were analyzed.

Gating strategy	Gating was performed using FlowJo software to eliminate debris (forward versus side scatter (FSC/SSC)) to analyze single cells (FSC-A/FSC-H), and to analyze live cells with either Zombie NIR (Biolegend 423106) or Zombie Violet (Biolegend 423113) Fixable Viability Kits.

☒ Tick this box to confirm that a figure exemplifying the gating strategy is provided in the Supplementary Information.
